# Effects of Ecosystem Recovery Types on Soil Phosphorus Bioavailability, Roles of Plant and Microbial Diversity: A Meta‐Analysis

**DOI:** 10.1002/ece3.71172

**Published:** 2025-04-25

**Authors:** Jinguo Hua, Wenyue Wang, Jinyu Huo, Lin Wu, Lingfeng Huang, Hongtao Zhong

**Affiliations:** ^1^ Key Laboratory of the Ministry of Education for Coastal and Wetland Ecosystems, College of the Environment and Ecology Xiamen University Xiamen Fujian China; ^2^ College of Horticulture and Forestry Huazhong Agricultural University Wuhan Hubei China; ^3^ School of Biological Sciences The University of Western Australia Perth Western Australia Australia

**Keywords:** ecological rehabilitation, ecological restoration, microbial diversity, plant diversity, soil phosphorus

## Abstract

Strategies for restoring degraded ecosystems vary widely in the levels of human intervention. It has commonly been assumed that recovery with artificial inputs would be quicker and more efficient. However, is this truly the situation? We conducted a meta‐analysis to evaluate the differences and applicability between ecological restoration and ecological rehabilitation. Relationships between soil phosphorus content, plant diversity, and soil microbial diversity were analyzed using 463 valid experimental data points collected from 72 publications. The results indicated that in grassland ecosystems, ecological restoration outperformed rehabilitation by 35%, 68%, 38%, and 48% in belowground biomass, community coverage, plant richness, and Shannon diversity, respectively. In forests, rehabilitation trailed behind restoration by 58%, 26%, and 92% in belowground biomass, Simpson diversity, and bacterial Shannon diversity. Furthermore, there was minimal difference in the recovery mode among different fungal and bacterial phyla. Rehabilitation demonstrated lower stability and efficiency in long‐term phosphorus cycling compared to restoration. Overall, ecological restoration offers more stable and efficient long‐term phosphorus cycling, thereby questioning the effectiveness of ecological rehabilitation for sustainable ecosystem recovery, especially for species diversity and phosphorus cycling.

## Graphical abstract

We did a meta‐analysis intended to explore the correlation between biodiversity and soil phosphorus before and after environmental changes. Our findings provide a practical reference for the recovery of degraded and damaged forests and grasslands, highlighting the significance of ecological restoration in ecosystem recovery efforts as the important role of plant and microbial biodiversity in phosphorus acquisition during the natural recovery process.

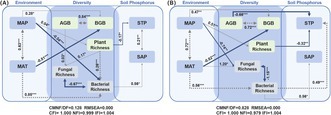

## 1 Introduction

The Anthropocene is characterized by rapid landscape modification and climate change, intensely altering Earth's surface processes and exacerbating various environmental issues (Steffen et al. 2007; Fu et al. 2022). In response to global environmental challenges, including biodiversity loss and ecosystem degradation, ecosystem recovery has emerged as a critical strategy for promoting sustainability (IPBES 2019). The United Nations has designated the Decade of Ecosystem Restoration (2021–2030) to enhance global restoration efforts (United Nations Environment Agency 2019). However, evaluating the efficacy of ecosystem recovery has become increasingly complex, requiring considerations beyond eco‐physical outcomes to encompass ecosystem multifunctionality and human well‐being (Crossman and Bryan 2009; Mastrangelo et al. 2014). Therefore, targeted research is crucial for developing strategies that are aligned with conservation priorities and sustainable development goals (Anderegg et al. 2020; Shi et al. 2024).

Ecosystem recovery can occur naturally when human activities like agriculture, logging, or grazing are ceased (Guariguata et al. 1997; Meli et al. 2017), allowing ecosystems to regenerate through ecological succession—a process referred to here as “ecological restoration” (Corlett 2016; Gerwing et al. 2022). However, natural recovery is often slow and is influenced by factors such as biogeochemical cycles and existing biodiversity (Walther 2010; Rey Benayas et al. 2009; Karssenberg et al. 2017). In cases of severe degradation, active human intervention, including soil improvement, reforestation, or landscape reshaping, is often required to accelerate the recovery trajectory (Holl and Kappelle 1999; Hobbs and Cramer 2008). This active intervention is typically termed “ecological rehabilitation” (Corlett 2016; Gerwing et al. 2022).

Nutrient cycling is a fundamental driver of ecosystem recovery, influencing biodiversity, soil health, and overall ecosystem sustainability (Finzi et al. 2015; Jiao 2018). While previous research has extensively examined carbon and nitrogen cycling (Morrien et al. 2017; Lu et al. 2018), phosphorus (P) dynamics remain less understood despite their critical role in plant growth and ecosystem productivity (Raven et al. 2018). Furthermore, P limitation of terrestrial ecosystem production is more prevalent than previously recognized (Hou et al. 2020). Plants and microorganisms employ diverse strategies to acquire P (Lambers et al. 2008; Richardson and Simpson 2011), including root morphological adjustments (White et al. 2013; Lynch 2019), symbiotic relationships with mycorrhizal fungi (van der Heijden et al. 2015), and modifications in root exudate composition (Shen et al. 2011; Yin et al. 2013). However, the intricate mechanisms governing plant –microbe interactions for P acquisition under different recovery types remain poorly understood.

Biodiversity is closely linked to nutrient cycling, and an increase in biodiversity contributes to the enhancement of ecosystem resilience and stability (Loreau and Behera 1999). Similarly, soil microbial diversity is a central factor influencing ecosystem functioning (Bernhard and Kelly 2016; Tanentzap et al. 2019). Changes in plant diversity and biomass influence soil microbial communities, subsequently affecting P transformations (Proulx et al. 2010). However, the majority of existing studies have been investigating plants, microorganisms, or soils separately, with relatively less attention paid to their collective roles during ecological restoration. Additionally, the effects of different recovery types on plant and microbial diversity and their interaction mechanisms require further investigation. A comprehensive understanding of how plant and microbial diversity interact synergistically to influence P acquisition during ecological recovery is therefore essential.

This present study aims to investigate the rate of responses between different ecosystem recovery types, biodiversity, and soil P dynamics by conducting a global meta‐analysis of field data. Specifically, we address two key questions: (1) how does biodiversity (encompassing plant and microbial diversity) vary under different ecosystem recovery types? (2) what are the trends of synergistic changes among plant diversity, microbial diversity, and soil P (including soil total phosphorus and soil available phosphorus) during the ecosystem recovery process? The findings of this study have the potential to better support the researchers and practitioners in restoring biodiversity under specific environmental conditions using different recovery types, ultimately supporting sustainable ecosystem recovery.

## 2 Materials and Methods

### 2.1 Data Collection

On the basis of the Web of Science (http://isiknowledge.com), Google Scholar (https://scholar.google.com), and the China National Knowledge Infrastructure (http://www.cnki.net), we compiled data on the rate of response in different restoration types, we searched for peer‐reviewed literature published before 30 July 2024. We used the following combinations of keywords: “restor*” OR “recover*” OR “rehabilit*” OR “natural succession” OR “secondary succession” OR “vegetation succession” OR “chronosequence” AND “soil microb*” OR “soil bacteria*” OR “soil fung*” OR “diversity” OR “richness” OR “biomass” OR “coverage” OR “AGB” OR “BGB” AND “soil total phosph*” OR “soil available phosph*” OR “soil phosph*” OR “STP” OR “SAP”.

The matching articles were found using the following criteria: (1) field experiments were conducted in semi‐natural or natural grasslands, forests, or croplands, incorporating both ambient and nutrient addition treatments, while excluding studies focused on mine site rehabilitation and restoration; (2) studies were required to report data on soil total phosphorus (STP) or soil available phosphorus (SAP), and at least one parameter associated with vegetation or soil microbial diversity, such as vegetation coverage, biomass, plant or microbial α‐diversity (e.g., richness, Shannon diversity, Simpson diversity and Chao 1), or microbial biomass phosphorus (MBP); (3) reported data had to include means, standard deviations (SD), or standard errors (SE) and sample sizes for control and treatment groups were reported, allowing SE to be converted to SD using the formula SE $\sqrt{n}$, with missing SD values approximated as 1/10 of the mean (Luo et al. 2006); (4) studies contained multiple field experiments with varying vegetation types, altitudes, or terrains, so each experiment was treated as an independent case for analysis; (5) studies focusing on the control of exotic species were excluded.

Based on these criteria, 72 publications with 463 valid experimental data points were collected (Figure S1). In addition to directly obtained data from tables and texts, we extracted the data by using GetData Graph Digitizer software version 2.26 (http://getdata‐graphdigitizer.com/). In our compiled dataset, the following information was compiled: (1) soil total phosphorus and soil available phosphorus; (2) plant α‐diversity, degree of coverage, and biomass, including plant richness, plant Shannon diversity, plant Simpson diversity, aboveground biomass (AGB), belowground biomass (BGB), and litter biomass (LB); (3) microbial groups, divided into bacteria and fungi, along with microbial biomass phosphorus (MBP) and their respective α‐diversity metrics, including richness, Shannon diversity, and Chao 1; (4) the relative abundance of 12 major microbial phyla, with six for bacteria (Proteobacteria, Actinobacteria, Acidobacteria, Firmicutes, Bacteroidetes, Gemmatimonadetes) and six for fungi (Ascomycota, Basidiomycota, Mortierellomycota, Glomeromycota, Rozellomycota, Chytridiomycota).

Additionally, we collected the coordinates (latitude and longitude) of the study locations, climatic variables (mean annual temperature, MAT; mean annual precipitation, MAP), ecosystem types (forest, shrubland, grassland), pedogenic conditions (initial phosphorus content), and experimental conditions. For studies that did not report MAT and MAP, these values were extracted based on geographic location from WorldClimate (https://www.worldclimate.com). The majority of the study sites (about 97.4%) were located in China (Figure S2). The experiments were categorized into subgroups based on recovery types (ecological restoration vs. ecological rehabilitation), ecosystem types (forest, shrubland, grassland), and restoration duration (0–10 years, 10–20 years, > 20 years). When an ecosystem is damaged and then restored by its own recovery capacity, it is hereinafter referred to as “ecological restoration” The recovery that is artificially assisted is hereinafter referred to as “ecological rehabilitation”

In terms of experimental types, ecological restoration experiments accounted for approximately 71.27% of the data, involving measures such as physical barriers (e.g., fencing to prevent grazing), reduced agricultural activity (e.g., land abandonment), and ecological recovery projects (e.g., reforestation or grassland restoration). Ecological rehabilitation experiments made up about 28.73% of the dataset and included interventions such as soil improvement, inorganic fertilization or organic amendment, and afforestation. In terms of restoration duration, short‐term experiments were more common; about 65.66% of the experimental time series were < 20 years. Long‐term experiments were less frequent, with only about 34.34% of the experimental observations lasting for > 20 years. Grassland and forest ecosystems were the dominant studied ecosystems, accounting for 63.49% and 31.94% of the studies, respectively. Shrubland only made up a smaller portion (4.57%) and was categorized as part of the forest ecosystem.

### 2.2 Data Analysis

We used the natural log‐transformed response ratio (lnRR) to quantify the recovery rates under ecological restoration versus ecological rehabilitation. The lnRR, also known as the “effect size”, is a dimensionless metric used to represent the relative changes between treatment and control groups (Hedges et al. 1999). In this study, the treatment group represents the condition of the sample plots after different ecosystem recovery types, while the control group represents the condition of the sample plots before degradation and damage:

(1) $$\ln\mathrm{RR}=\ln\left(\frac{{\bar{X}}_{t}}{{\bar{X}}_{c}}\right)=\ln\left({\bar{X}}_{t}\right)-\ln\left({\bar{X}}_{c}\right).$$

where ${\bar{X}}_{t}$ and ${\bar{X}}_{c}$ are the mean response values for the treatment and control groups, respectively.

The variance of lnRR(v) was calculated as:

(2) $$v=\frac{s_{t}^{2}}{n_{t}{\bar{X}}_{t}^{2}}+\frac{s_{c}^{2}}{n_{c}{\bar{X}}_{c}^{2}}$$

where $s_{t}$ and $s_{c}^{2}$ are the standard deviations of the treatment and control groups, and $n_{t}$ and $n_{c}$ are the sample sizes of the treatment and control groups, respectively.

The initial weight (w) of each observation was calculated as the inverse of $\left(v\right)$. However, if multiple observations for the same variable were included from a single study, the adjusted weight ($w^{\prime }$) was calculated as follows (Bai et al. 2013):

(3) $$w^{\prime }=\frac{w}{n}$$

where $\left(n\right)$ represents the total number of observations for the variable within the same study.

Finally, the overall mean effect size ($\ln RR^{\prime }$) across all observations was calculated using the following formula:

(4) $$\ln RR^{\prime }=\ln\mathrm{RR}\times w^{\prime }$$

(5) $$\bar{\ln RR^{\prime }}=\frac{\sum_{i}\;\ln RR_{i}^{\prime }}{\sum_{i}\;w_{i}^{\prime }}$$

where ($\ln RR^{\prime }$) is the weighted effect size of each observation and ($\ln RR_{i}^{\prime }$) and ($w_{i}^{\prime }$) are the ($\ln RR^{\prime }$) and ($w^{\prime }$) of the (i) observation, respectively.

To present the effect size more intuitively, $\bar{\ln RR^{\prime }}$ was converted to a percentage change using the following formula:

(6) $$\text{Percentage change}\;\left(\%\right)=\left(e^{\bar{\ln RR^{\prime }}}-1\right)\times 100\%$$

Publication bias is a common issue in meta‐analyses, often arising from small sample effects, therefore can significantly influence the validity of the results. To assess publication bias for each variable, we used funnel plots and Egger's test in the “metafor” package (Figure S3). Variables showing bias were further examined using the trim‐and‐fill method, which estimates and fills in missing data to create a more symmetric funnel plot (Duval and Tweedie 2000), allowing us to evaluate whether the bias affects the meta‐analysis (Peters et al. 2008). After adjusting for missing data, we calculated the 95% confidence interval (CI) for the imputed mean effect size ($\bar{\ln RR^{\prime }}$). If the imputed ($\bar{\ln RR^{\prime }}$) was not significantly different from the original ($\bar{\ln RR^{\prime }}$), we concluded that publication bias did not affect the analysis, with a significant correlation (*p* < 0.05) indicating possible bias (Rosenberg 2005).

Linear regression is used to evaluate the relationship among soil phosphorus, plant diversity, and relative abundance of soil microbial diversity. In the meta‐analysis, we applied a random‐effects model using the “rma.mv” function in the “metafor” package to assess the significance of results based on 95% confidence intervals (95% CI) (Viechtbauer 2005). If the 95% CI of the mean effect size does not overlap with zero, this indicates a statistically significant effect of soil phosphorus content on the variable, with positive or negative effects depending on whether the effect size is greater or less than zero. If the 95% CI includes zero, the results are considered non‐significant.

Structural equation modeling (SEM) was employed to determine the combined effects of climate, plant diversity, and microbial diversity on soil phosphorus under different ecosystem recovery types. Model fit was assessed using the Chi‐squared test (χ^2^). A model was considered acceptable if RMSEA was < 0.08, the CMNF/DF ranged from 0 to 2, and NFI, CFI, or IFI exceeded 0.9. Lower RMSEA values indicate a better fit, with values above 0.1 suggesting a poor model fit, while RMSEA values below 0.05 and indexes close to 1 indicate an excellent model fit (Zhang et al. 2019). All statistical tests were performed using R (version 4.3.2, http://www.datavis.ca/R/).

## 3 Results

### 3.1 Effects of Recovery Types on Plant Diversity, Soil Microbial Diversity, and Soil Phosphorus

**FIGURE 1:**
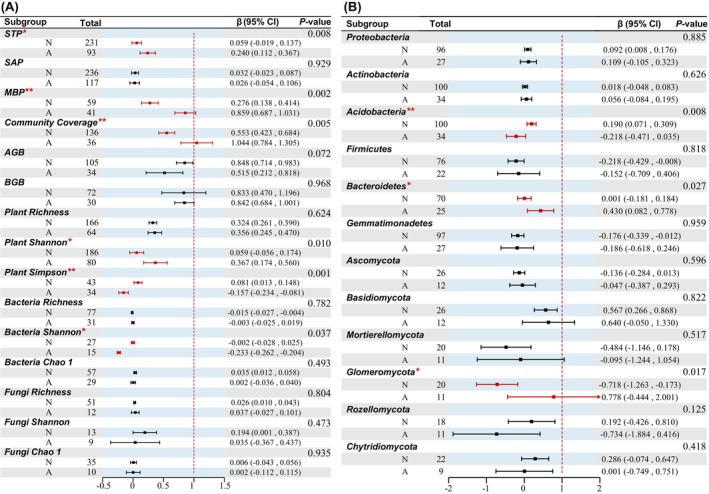
Effects of ecosystem recovery type on plant diversity, soil microbial diversity, soil phosphorus, and microbial phylum: (A) Effects of restoration type on plant diversity, soil microbial diversity, and soil phosphorus; (B) effects of restoration type on the relative abundance of the main microbial phylum. N, Ecological restoration; A, ecological rehabilitation. Values in the total columns indicate the sample size for each variable. Red lines indicate significant differences, while *p < 0.05 and **p < 0.01. AGB, above‐ground biomass; BGB, below‐ground biomass; MBP, microbial biomass phosphorus; SAP, soil available phosphorus; STP, oil total phosphorus.

Ecological restoration significantly increased the overall response ratio (RRs) of plant community coverage, aboveground biomass (AGB), belowground biomass (BGB), and plant species richness by 55.30%, 84.80%, 83.30%, and 32.40%, respectively. In contrast, ecological rehabilitation increased the plant community coverage, AGB, BGB, and species richness of the plant community coverage by 104.40%, 51.50%, 84.20%, and 35.60%, respectively. Except for the RRs of AGB, ecological rehabilitation showed a higher degree than ecological restoration in all other indicators. Comparisons between the different recovery types showed greatly significant differences (*p* < 0.01, Figure 1A) in the RRs of microbial biomass phosphorus (MBP), community coverage, and plant Simpson diversity. The RRs of soil total phosphorus (STP), plant Shannon diversity, and bacterial Shannon diversity were significant differences (*p* < 0.05, Figure 1A) between different ecosystem recovery types, whereas there was no significant difference between the RRs of soil available phosphorus (SAP) (*p* > 0.05, Figure 1A).

Along the restoration trajectory, both ecosystem recovery types resulted in significant increases in the relative abundance of “Basidiomycota” with increases of 56.70% and 64.00% in ecological restoration and ecological rehabilitation, respectively (Figure 1B). The relative abundance of “Firmicutes” “Gemmatimonadetes” “Ascomycota” and “Mortierellomycota” all showed a declining trend as recovery proceeds. In addition, the comparison of different ecosystem recovery types showed significant differences in the relative abundance of “Acidobacteria” “Bacteroidetes” and “Glomeromycota” (*p* < 0.05, Figure 1B).

### 3.2 Effects of Recovery Types on Plant Diversity and Soil Microbial Diversity Under Different Ecosystems and Recovery Durations

**FIGURE 2:**
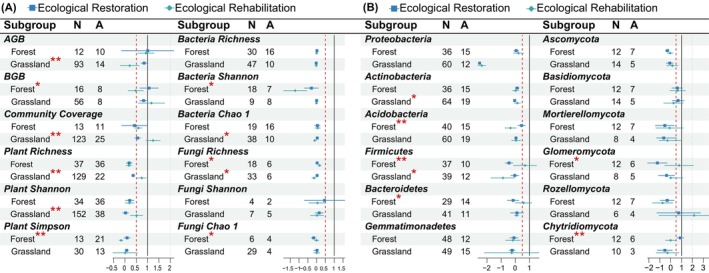
Effects of ecosystem recovery type on plant diversity, soil microbial diversity and microbial phylum between forest and grassland ecosystems: (A) Effects of ecosystem type on plant diversity and soil microbial diversity and (B) effects of ecosystem type on the relative abundance of the main microbial phylum. A, ecological rehabilitation; N, Ecological restoration. The second and third columns in each table are the sample size for the variables. Points with error bars represent weighted means and their 95% RR confidence intervals (CI). Red asterisks indicate that responses between different recovery indicators were considered significant, while *p < 0.05 and **p < 0.01. AGB, bove‐ground biomass; BGB, below‐ground biomass; MBP, microbial biomass phosphorus.

In forest ecosystems, the RRs of BGB, plant Simpson diversity, fungi richness, bacteria Shannon diversity, and fungi Chao 1 showed significant differences among different recovery types (*p* < 0.05, Figure 2A). Among them, the effect of ecosystem recovery on RRs of BGB and plant Simpson diversity was 58.41% and 26.42% higher under ecological restoration than under ecological rehabilitation, respectively. Grassland ecosystems, on the other hand, had significant differences in RRs of above‐ground biomass (AGB), community coverage, plant richness, plant Shannon diversity, bacteria Chao 1, and fungi Chao 1 (*p* < 0.05, Figure 2A). However, ecological rehabilitation showed greater recovery than ecological restoration in most plant tested indicators (Figure 2A), except for AGB and Plant Simpson. In addition, forest ecosystems showed significant differences in RRs of “Acidobacteria”, “Firmicutes”, “Bacteroidetes”, “Glomeromycota”, and “Chytridiomycota” between recovery types. Yet, in grassland ecosystems, only the abundance of RRs of “Actinobacteria” and “Firmicutes” differed significantly among ecosystem recovery types (*p* < 0.05, Figure 2B).

**FIGURE 3:**
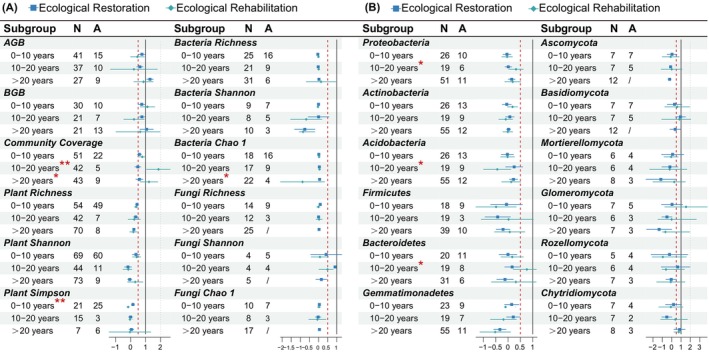
Effects of ecosystem recovery type on plant diversity, soil microbial diversity and microbial phylum between different restoration durations: (A) Effects of restoration durations on plant diversity and soil microbial diversity; (B) Effects of restoration durations on the relative abundance of the main microbial phylum. A, ecological rehabilitation; N, ecological restoration. The second and third columns in each table are the sample size for the variables. Points with error bars represent weighted means and their 95% RR confidence intervals (CI). Red asterisks indicate that responses between different recovery indicators were considered significant, while *p < 0.05 and **p < 0.01. AGB, above‐ground biomass; BGB, below‐ground biomass; MBP, microbial biomass phosphorus.

For the recovery durations, highly significant differences between recovery types only showed in RRs of community coverage between 10 and 20 years, while the RRs of plant Simpson diversity were significant during the first 10 years (*p* < 0.01, Figure 3A). Ecological rehabilitation showed varying degrees of higher recovery rates for community coverage than ecological restoration in the three phases of restoration, which were higher than 19.08%, 137.88%, and 64.65%, respectively (Figure 3A). Community coverage and bacteria Chao 1 recovery rates by recovery type were significantly different at > 20 years (*p* < 0.05, Figure 3A). There was a significant difference in RRs of community coverage and bacteria Chao 1 by type of recovery for > 20 years (*p* < 0.05, Figure 3A). In addition, there was also a significant difference in the recovery rates for “Acidobacteria” and “Bacteroidetes” in the microbial phylum over10–20 years (*p* < 0.05, Figure 3B).

### 3.3 The Relationship Between Soil Phosphorus and Plant or Microbial Diversity Along Recovery Trajectory

**FIGURE 4:**
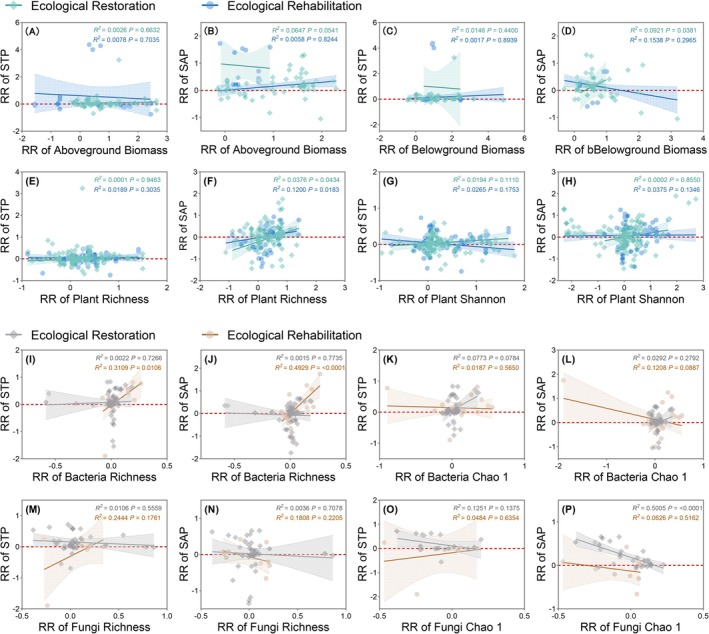
Response rate (RR) of plant biomass (A–D), plant diversity (E–H), bacterial diversity (I–L), and fungal diversity (M–P) in relation to response values of soil total phosphorus (STP) and soil available phosphorus (SAP) for different ecosystem recovery types. Shaded areas represent 95% confidence intervals.

Under ecological restoration conditions, most of the soil total phosphorus (STP) and soil available phosphorus (SAP) response rates (RRs) increased with increasing RRs of biomass and plant diversity. Two exceptions occurred in RRs of SAP to aboveground biomass and STP to belowground biomass, respectively. However, only the RRs of BGB to SAP and plant diversity's RRs to SAP showed significant correlations (*p* < 0.05, Figure 4D,F). Additionally, under ecological restoration, the RRs of STP and SAP showed fluctuations or slight increases as bacterial RRs increased, while they decreased as fungal RRs rose (Figure 4K–N). In contrast, under ecological rehabilitation, no clear correlation was observed between the RRs of STP and SAP and plant or soil microbial biodiversity. However, the overall increase in biodiversity's RRs due to ecological rehabilitation was more pronounced compared to that of ecological restoration (Figure 4).

**FIGURE 5:**
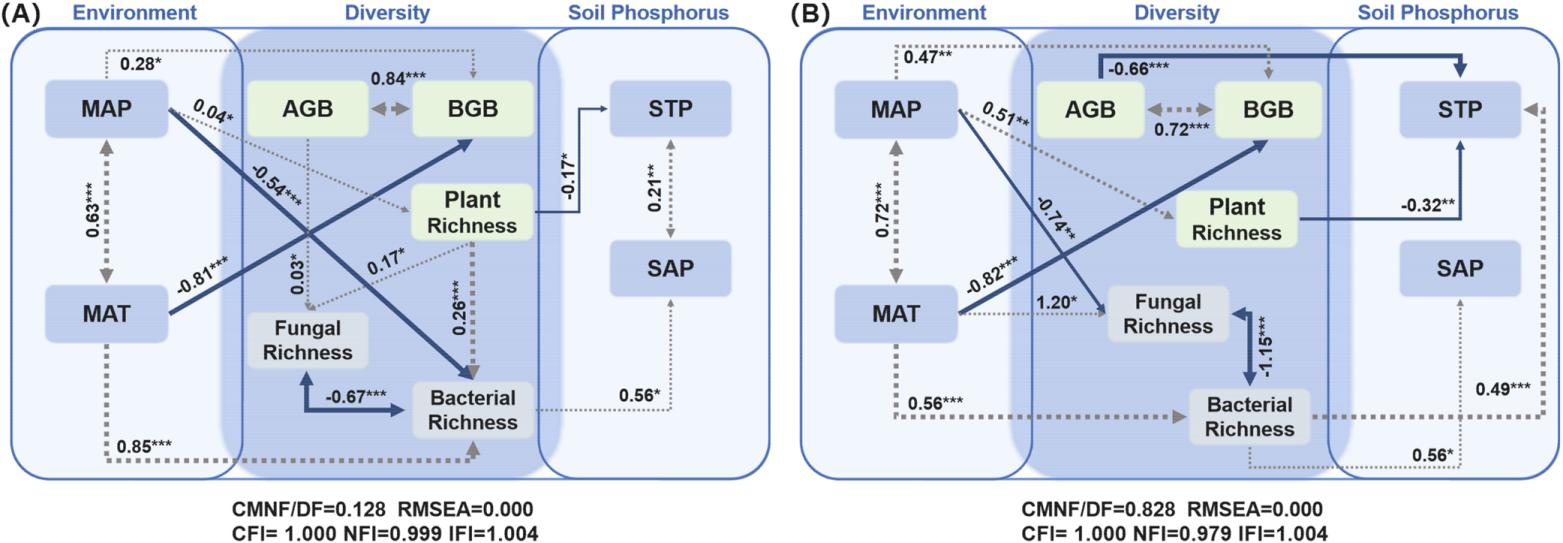
Structural equation modeling (SEM) describing the direct or indirect effects of climate, plant diversity, and microbial diversity on total and available phosphorus in soil under different ecosystem recovery types: (A) Ecological restoration and (B) ecological rehabilitation. Gray dashed and blue solid arrows indicate positive and negative relationships, respectively. Arrow widths are proportional to the strength of the relationship. The numbers on the arrows indicate significant standardized path coefficients, while *p < 0.05 and **p < 0.01 and ***p < 0.001. AGB, above‐ground biomass; BGB, below‐ground biomass; MAP, mean annual precipitation; MAT, mean annual temperature; SAP, soil available phosphorus; STP, soil total phosphorus.

The results of the structural equation model (SEM) indicate that under ecological restoration conditions, mean annual precipitation (MAP) indirectly affects soil P levels by influencing plant and bacterial diversity (Figure 5A). Specifically, the RRs of STP had a significant negative correlation with the increase in plant diversity, while the RRs of SAP were significantly positively correlated with bacterial diversity (*p* < 0.05, Figure 5A). Moreover, the RRs of both bacterial and fungal diversity showed significant positive correlations with plant diversity (*p* < 0.05, Figure 5A). There was also a strong correlation between STP and SAP (*p* < 0.001, Figure 5A). Under ecological rehabilitation conditions, several factors significantly influenced STP, including AGB, plant diversity, and bacterial diversity (*p* < 0.05, Figure 5B). Plant diversity and AGB were significantly negatively correlated with STP, while SAP was significantly and positively correlated only with bacterial diversity (*p* < 0.05, Figure 5B). In contrast to the ecological restoration approach, which possessed a highly significant correlation between STP and SAP (*p* < 0.001, Figure 5B), there was no significant correlation between STP and SAP under ecological rehabilitation conditions (*p* > 0.05, Figure 5B).

## 4 Discussion

### 4.1 Changes of Plant Diversity, Microbial Diversity, and Key Microbial Phyla Along Recovery Trajectory

Vegetation recovery is widely recognized as an effective strategy for ameliorating the ecological conditions of fragile and degraded ecosystems (Rey Benayas et al. 2009; Ghosh et al. 2021). Our results show increased plant biomass and diversity during recovery (Figure 1). Notably, rehabilitation was found to be more effective than ecological restoration in enhancing community coverage and plant diversity, particularly within grassland ecosystems. In comparison, restoration was superior in forests for below‐ground biomass, Simpson diversity, and microbial diversity (Figure 2A). This reflects active interventions' effects on grasslands, potentially leading to competitive exclusion (Crouzeilles et al. 2016; Kralovec et al. 2009). Our results highlight that these effects are context‐dependent, with the benefits of rehabilitation diminishing in severely degraded grasslands. Restored forest microbial diversity responds strongly to long‐term succession, highlighting plant–soil interactions (Liu et al. 2021). These findings highlight the need for restoration strategies that prioritize long‐term resilience and ecosystem‐specific dynamics over short‐term gains. In severely degraded grasslands, rehabilitation can be less effective, even detrimental, compared to restoration (Wolf et al. 2018). Severely degraded grasslands often exhibit compromised soil structure, organic matter loss, and nutrient depletion (Cao et al. 2011), significant declines in soil microbial community abundance and functional diversity (Hallett et al. 2017), and reduced soil moisture retention capacity (Pugnaire et al. 2019). While active interventions can initially enhance vegetation cover, they may not necessarily sustain biodiversity and soil health over time, as natural recovery processes (e.g., seed bank recruitment, plant–soil‐microbe interactions) are essential for ecosystem resilience (De yn et al. 2003; Liu et al. 2021). Moreover, external inputs, such as fertilization, can sometimes induce nutrient imbalances, leading to unintended shifts in microbial composition that further constrain recovery (Carter and Blair 2012).

Regarding differences between recovery types, some studies suggest that ecological rehabilitation can significantly increase plant or microbial diversity in the short term, achieving faster outcomes than ecological restoration (Klopf et al. 2017; Chen et al. 2023). Short‐term recovery often entails significant economic investment in assisted rehabilitation (Meyer et al. 2019; Orrock et al. 2023). Our results indicated that within the first 10 years, ecological restoration and rehabilitation differed significantly only in plant Simpson diversity, with no significant differences observed in other biomass or diversity metrics. At longer timescales (> 10 years), significant differences emerged only in community coverage and bacterial Chao 1 (Figure 3A). The absence of a clear short‐term advantage for rehabilitation in our study contrasts with findings by Xu and Zhang (2021), who reported faster vegetation cover enhancement through rehabilitation. This inconsistency may stem from differing definitions of short‐term recovery. While our study emphasizes medium‐ and long‐term results, previous research often focused on overall community coverage rather than diversity.

Soil bacterial and fungal communities are crucial for ecosystem biogeochemical cycles and overall ecosystem health (van der Heijen 2008). In the present study, bacterial and fungal diversity exhibited minimal change before and after restoration (Figure 1B), consistent with other meta‐analyses (e.g., Watson et al. 2022; Zang et al. 2024). However, the abundance of eutrophic bacteria, such as Proteobacteria and Actinobacteria, increased during recovery, partially supporting the findings in Cheng et al. (2016), who noted that eutrophic bacteria proliferate as soil nutrients increase. Grasslands showed more Actinobacteria variability between recovery types (Figure 2B). The shift from fast‐growing Proteobacteria to slower‐growing Actinobacteria reflects microbial adaptation (Fierer et al. 2007; Zhou et al. 2017), especially in grassland rehabilitation. Yet, recovery types did not differ significantly in phyla over time (Figure 3B). Overall, rehabilitation offered no significant microbial recovery advantage over restoration in forests or long term.

### 4.2 Soil Phosphorus Levels Altered by Ecosystem Recovery

Ecosystems recovery exerts a substantial influence on above‐ground ecosystems and species diversity, while also impacting underground ecosystems by enhancing soil nutrient content and physical structure (Klopf et al. 2017; Wardle et al. 2004). Our study observed a negative correlation between soil total phosphorus (STP), microbial biomass phosphorus (MBP), and below‐ground plant biomass, consistent with findings that increased P availability can reduce microbial and root biomass (Xia et al. 2020; Yao et al. 2018). This suggests potential P competition between microbes and plants during recovery. In P‐saturated soils, root growth may be constrained, limiting belowground biomass (Wissuwa et al. 2005). Elevated P levels can also increase microbial immobilization, restricting plant uptake (Hepher 1958). Conversely, we found a positive correlation between soil available phosphorus (SAP) and below‐ground biomass. Under P‐deficient conditions, plants allocate more biomass to the root to optimize nutrient acquisition (Chapin 1980; Huluka and Evans 1991). However, this increased root growth, while facilitating acquisition, can impede overall plant growth due to respiratory costs (Caradus and Snaydon 1986), potentially explaining a less pronounced increase in plant diversity.

In forest ecosystems, ecological rehabilitation exhibited higher STP, SAP, and MBP recovery rates than ecological restoration during the initial recovery stages (*p* < 0.05, Figures S4 and S5). Grassland ecosystems, however, showed no significant differences between recovery types. This is because rehabilitation methods typically induce rapid increases in STP levels within the initial years of recovery initiation through direct application of fertilizers and soil amendments (Martínez‐Sánchez 2003; Wu and Liu 2013). In acidic forest soils, plant uptake of fertilizer‐derived P can be limited due to forming complexes with iron and aluminum oxides (Martínez‐Sánchez 2003; Shannon‐Firestone et al. 2015). Furthermore, fertilization can disrupt microbial P cycling, decreasing diversity and function, thus hindering plant uptake (Wu and Liu 2013; Trivedi et al. 2020). In grassland soils, droughts can exacerbate P limitation by reducing root exudation and disrupting mycorrhizal networks (Liu et al. 2023), whereas forest moisture fluctuations may alter plant –microbe P competition (Kong et al. 2021). Furthermore, temperature affects microbial enzyme activity and organic matter mineralization, influencing P solubilization (Allison et al. 2010; Steinweg et al. 2013). Elevated temperatures can enhance short‐term P mineralization but may disrupt long‐term C–P balance (Xu et al. 2020). Conversely, soil moisture regulates oxygen diffusion and redox conditions, which influence P release (Ding et al. 2021).

Regarding recovery duration, ecological rehabilitation promoted faster STP recovery within the initial 20 years, whereas ecological restoration exhibited superior recovery thereafter. For SAP, rehabilitation demonstrated slower initial recovery but surpassed restoration after 10 years (Figure S5). Despite fertilizer‐induced STP increases, SAP availability can decline due to P fixation. This occurs as P is immobilized by soil minerals or organic complexes, limiting plant uptake (Litaor et al. 2019). While prolonged fertilization maintains high soil P levels (Fay et al. 2015), over time, this can lead to declining soil health as ecosystems mature and reliance on external inputs becomes unsustainable (Hanisch et al. 2020; Rogers et al. 2022). In contrast, ecological restoration, relying on ecological succession and nutrient cycling, may exhibit slower initial STP recovery (Gunderson 2000; Li 2004). Unlike rehabilitation, restoration enhances P availability by promoting diverse plant communities and robust microbial networks (Huang et al. 2020; Dai et al. 2023). The existence of complex plant communities and symbiotic relationships with mycorrhizal fungi further enhances P uptake (He et al. 2022). However, after 20 years, the recovery rate for SAP in ecological restoration declines, indicating a potential steady state in natural P cycling (Li 2004; Schaerer et al. 2007). Therefore, while artificial amendments applied through rehabilitation may initially augment P levels, their long‐term sustainability remains questionable.

### 4.3 Plant‐Microbe‐Soil Phosphorus Interaction Mechanisms Under Different Recovery Types

Plant‐microbe interactions influence plant P acquisition strategies by shaping microbial community composition (Figure 5) (Paredes and Lebeis 2016). Our study revealed distinct plant‐microbe‐soil P interaction mechanisms between ecological restoration and rehabilitation (Figures 4 and 5). In rehabilitation, soil P content was influenced by plant and bacterial diversity and biomass (Figure 5B). In contrast, the enhanced plant diversity observed in restoration fostered greater microbial diversity, which in turn drove P cycling. Notably, in low‐fertility conditions, diverse plant communities strengthen associations with soil microbes, thereby enhancing P cycling (Schlatter et al. 2015). Below‐ground biomass positively correlates with soil P, as roots release organic acids, mobilizing P (Margalef et al. 2017; Yao et al. 2018). Roots also provide habitats for P‐solubilizing microbes, enhancing P cycling (Bardgett and van der Putten 2014; Rillig and Mummey 2006). Diverse plant communities foster microbial diversity (Xue et al. 2022), with bacterial communities further impacting the P cycle through processes like phosphatase (Acuña et al. 2016; Li et al. 2021).

An increase in STP does not necessarily translate to a proportional increase in plant‐available P, particularly in rehabilitation. Biomass allocation patterns in rehabilitation differ from natural systems, leading to discrepancies between total and available P (Figure 5B). While STP may increase, available P often remains low due to altered soil conditions that hinder solubilization and plant uptake (Flanagan and Richardson 2010). This disparity arises from the selection of economical or native species in rehabilitation, which may not support the comparable biodiversity or plant‐microbe‐soil interactions as restoration (Bucharova et al. 2017; Budiharta et al. 2014). However, certain plant species, like *Digitaria sanguinalis* (Poaceae) and *Trifolium repens* (Fabaceae), can improve soil nutrient status by increasing cation exchange capacity, reducing P fixation, and improving P availability for plant uptake (Wang et al. 2016).

Consequently, rehabilitation can reduce microbial diversity, alter nutrient dynamics, and hinder the establishment of beneficial microbial communities that are critical for P cycling (Dupre la Tour et al. 2020). Conversely, restoration enhances biodiversity, fostering resilient ecosystems capable of adapting to environmental changes (Elmqvist et al. 2003; Hong et al. 2022). Rehabilitation's focus on rapid soil fertility can lead to ecosystem instability, degraded soil health, and a feedback loop of reduced microbial diversity and further soil degradation (Fierer and Jackson 2006; Hong et al. 2022).

### 4.4 Implications for Ecosystem Recovery Practices

Ecological rehabilitation, while capable of enhancing soil nutrient levels in the short term, is often less effective than ecological restoration over extended periods. However, this observation is not applicable across all ecosystems. We argue that immediate implementation of ecological rehabilitation at damaged or degraded sites, without prior site surveys and research, should be discontinued. Rehabilitation efforts should be implemented only after thorough assessment, controlling for relevant biotic and abiotic factors. We recommend that (1) in forest ecosystems with relatively less degradation or damage, ecological rehabilitation should be applied with caution, but rather rely on the resilience of nature ecological restoration; (2) the application of artificial fertilizers or amendments needs to be carefully assessed, as these practices do not consistently result in sustainable long‐term phosphorus (P) utilization or the establishment of healthy soil quality within restored ecosystems; and (3) recovery practices should emphasize strategies that increase below‐ground biomass and microbial diversity to improve soil P bioavailability.

## 5 Conclusion

Our meta‐analysis reveals that ecological rehabilitation is significantly less effective than ecological restoration in maintaining long‐term phosphorus cycling stability. While ecosystem‐based ecological restoration enhances biodiversity and promotes sustainable nutrient cycling, ecological rehabilitation, which often focuses on short‐term recovery, progresses more slowly than commonly assumed and may compromise soil quality and ecosystem stability. In forest ecosystems, recovery efforts should prioritize natural processes, with human interventions carefully evaluated. Furthermore, enhancing belowground biomass and microbial diversity is crucial for optimizing phosphorus bioavailability. Since this study only analyzed STP and SAP data, future research should focus on organic P pools and their interactions with plant and microbial diversity. A more holistic approach, integrating plant–soil–microbe interactions, will improve recovery effectiveness and enhance ecosystem resilience.

## Author Contributions

**Jinguo Hua:** data curation (equal), formal analysis (equal), methodology (equal), software (equal), visualization (equal), writing – original draft (equal). **Wenyue Wang:** data curation (equal), visualization (equal), writing – original draft (equal). **Jinyu Huo:** formal analysis (equal), methodology (equal), software (equal). **Lin Wu:** investigation (equal), methodology (equal). **Lingfeng Huang:** conceptualization (equal), funding acquisition (equal), project administration (equal), validation (equal), writing – review and editing (equal). **Hongtao Zhong:** conceptualization (equal), funding acquisition (equal), project administration (equal), supervision (equal), writing – original draft (equal), writing – review and editing (equal).

## Conflicts of Interest

The authors declare no conflicts of interest.

## Supporting information


Appendix S1.

Appendix S2.

Appendix S3.

Appendix S4.

## Data Availability

The datasets for this study are accessible in Supporting Information.
