# Supplementary material for: Effects of Ecosystem Recovery Types on Soil Phosphorus Bioavailability, Roles of Plant and Microbial Diversity: A Meta‐Analysis
Source: Ecol Evol. 2025 Apr 25;15(4):e71172. doi: 10.1002/ece3.71172 (PMC12032196; doi:10.1002/ece3.71172)
Supplement: Supplementary file 1 — Appendix S1. [file ECE3-15-e71172-s001.docx]

**Supplementary information1**

**Effects of ecosystem recovery types on soil phosphorus bioavailability, roles of plant and microbial diversity: a meta-analysis**

Jinguo Hua ^1^, Wenyue Wang ^2^, Jinyu Huo ^1^, Lin Wu ^1^, Lingfeng Huang ^1*^, Hongtao Zhong ^1,3*^

^1^ Key Laboratory of the Ministry of Education for Coastal and Wetland Ecosystems, College of the Environment and Ecology, Xiamen University, Xiamen, Fujian 361102, China

^2^ College of Horticulture and Forestry, Huazhong Agricultural University, Wuhan, Hubei 430070, China

^3^ School of Biological Sciences, The University of Western Australia, Perth, WA 6009, Australia

*Authors for correspondence:

Hongtao Zhong (zhonght@xmu.edu.cn), Lingfeng Huang (huanglf@xmu.edu.cn)

**List of Contents**

**Fig. S1.** The PRISMA flow diagram.

**Fig. S2.** Distribution of experiments included in this meta-analysis in relation to effect of restoration types on soil phosphorus bioavailability, plant and microbial diversity.

**Fig. S3.** Funnel charts of data sensitivity analysis results.

**Fig. S4.** Effects of recovery type on phosphorus levels between forest and grassland ecosystems.

**Fig. S5.** Effects of recovery types on phosphorus levels between different restoration durations.

**Notes S1.** Studies included in the meta-analysis.


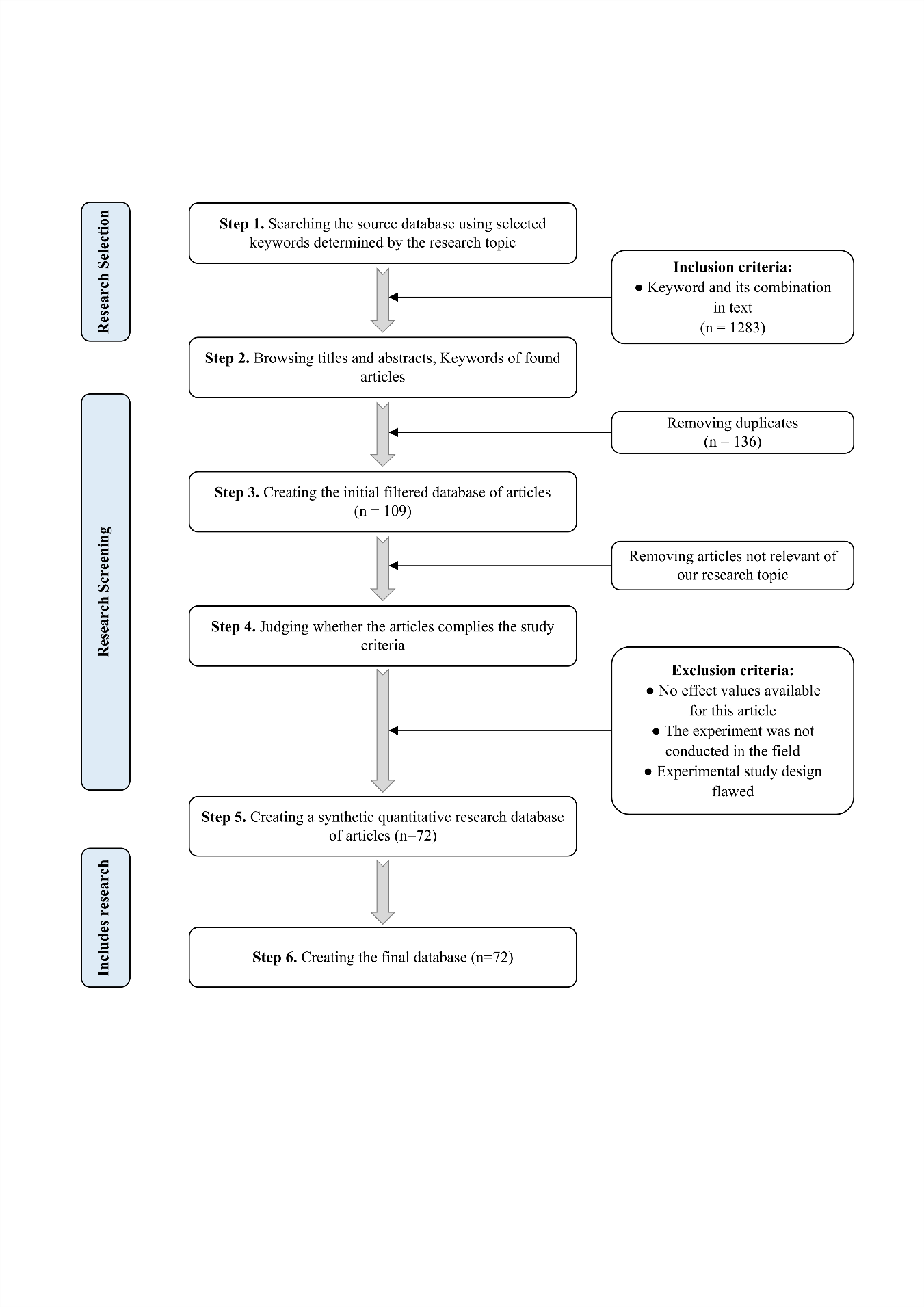


**Fig. S1.** The PRISMA flow diagram.

**
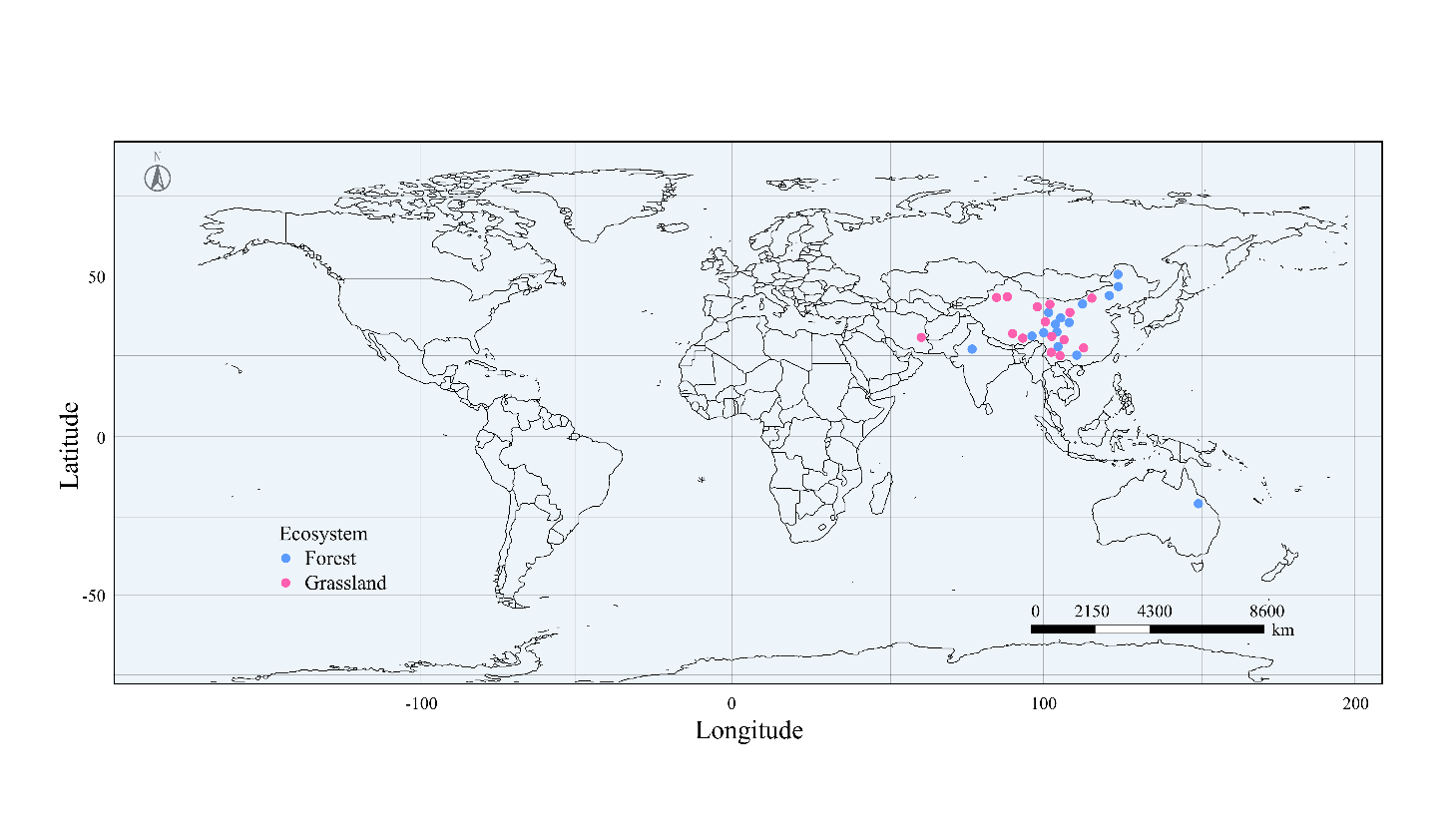
**

**Fig. S2.** Distribution of experiments included in this meta-analysis in relation to effect of recovery types on soil phosphorus bioavailability, plant and microbial diversity. Note that in some cases there are overlapped data points.


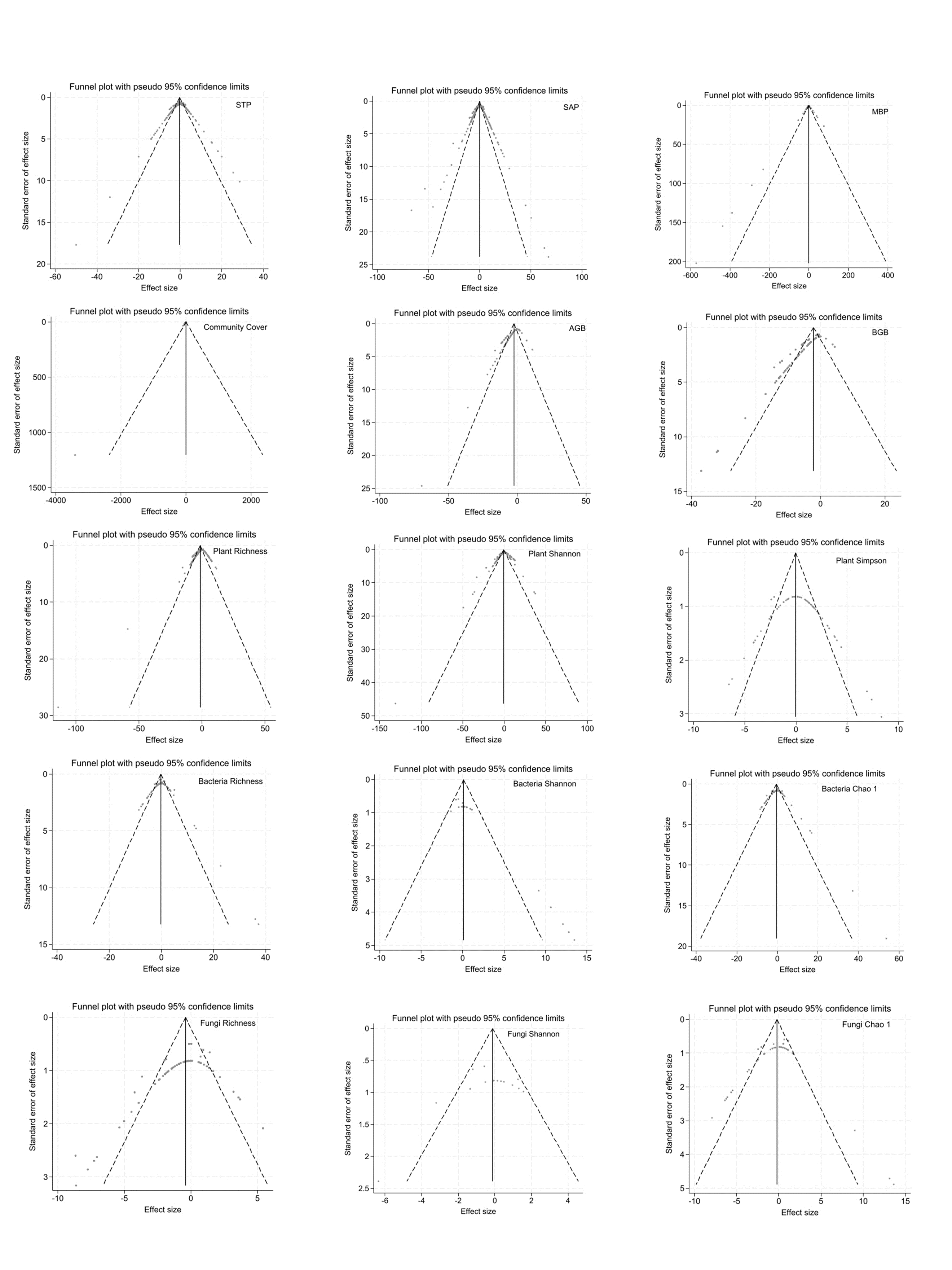


**Fig. S3.** Funnel charts of data sensitivity analysis results. STP, soil total phosphorus; SAP, soil available phosphorus; MBP, microbial biomass phosphorus; AGB, aboveground biomass; BGB, belowground biomass.


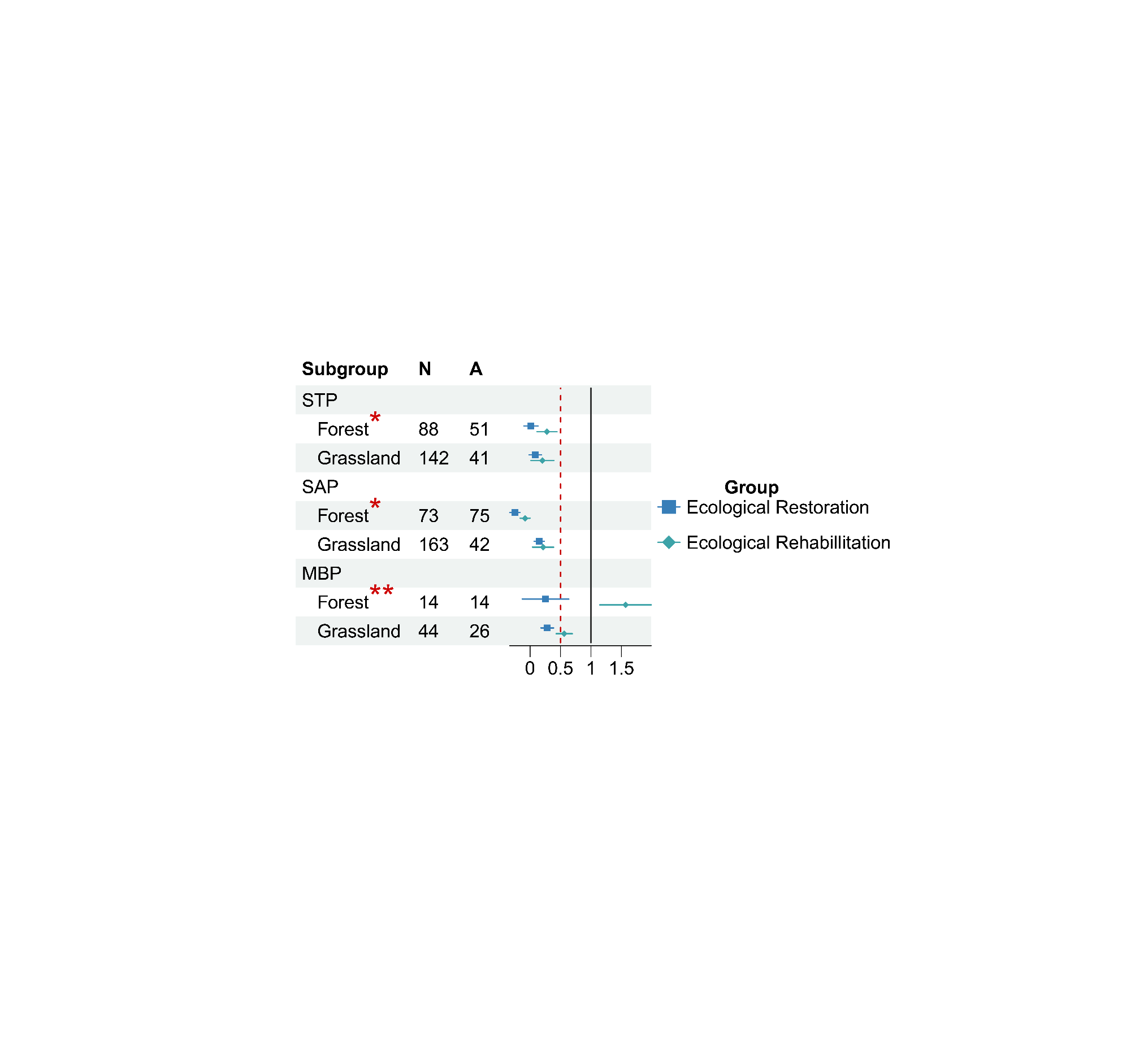


**Fig. S4.** Effects of recovery types on phosphorus levels between forest and grassland ecosystems. N, ecological restoration; A, ecological rehabilitation. Second and third columns are the sample size for the variables. Points with error bars represent weighted means and their 95% RR confidence intervals (CI). Red asterisks indicate that responses between different recovery indicators were considered significant, while * indicates *P* < 0.05 and ** indicate *P* < 0.01. STP, soil total phosphorus; SAP, soil available phosphorus; MBP, microbial biomass phosphorus.


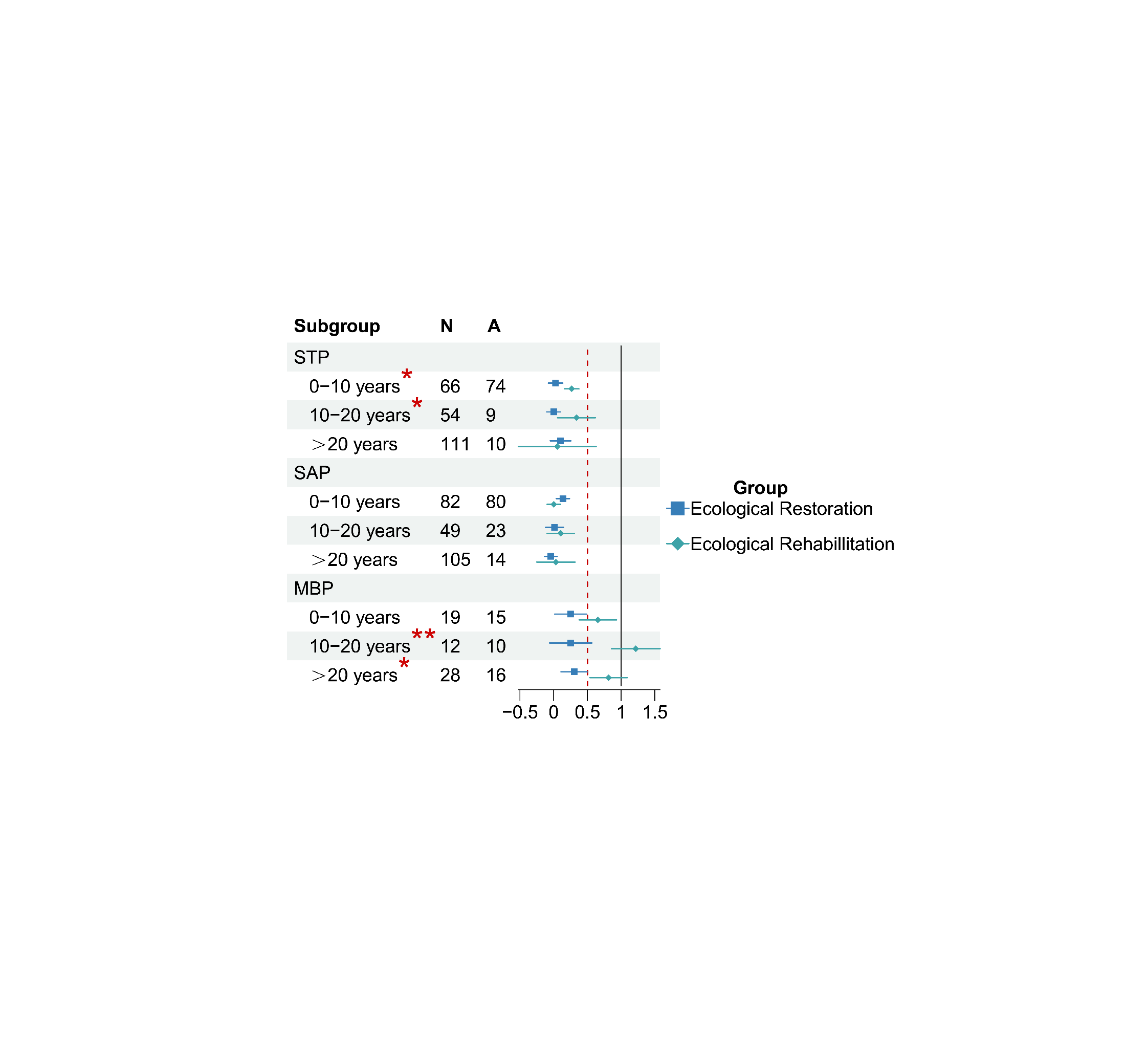


**Fig. S5.** Effects of recovery types on phosphorus levels between different restoration durations. N, ecological restoration; A, ecological rehabilitation. Second and third columns are the sample size for the variables. Points with error bars represent weighted means and their 95% RR confidence intervals (CI). Red asterisks indicate that responses between different recovery indicators were considered significant, while * indicates *P* < 0.05 and ** indicate *P* < 0.01. STP, soil total phosphorus; SAP, soil available phosphorus; MBP, microbial biomass phosphorus.

**Notes S1.** Studies included in the meta-analysis.

Amarasinghe, A., Knox, O.G.G., Fyfe, C., Lobry de Bruyn, L.A, Wilson, B.R., 2021. Response of soil microbial functionality and soil properties to environmental plantings across a chronosequence in south eastern Australia. Appl. Soil Ecol. 168, 104100. https://doi.org/10.1016/j.apsoil.2021.104100.

Chen, X., Zhang, Z., Han, X., Hao, X., Lu, X., Yan, J., Biswas, A., Dunfield, K., Zou, W., 2021. Impacts of land-use changes on the variability of microbiomes in soil profiles. J. Sci. Food Agric. 101, 5056–5066. https://doi.org/10.1002/jsfa.11150.

Chen, X.X, Li, H.L., Dong, Z., Chen, P., Qiu, S.Q., Gao, Y., 2019. Effects of soil remediation and changes of plant diversity in burned areas under different afforestation models (in China). Journal of Soil and Water Conservation 03, 332-339. https://doi.org/10.13870/j.cnki.stbcxb.2019.03.049.

Cheng, J., Jing, G., Wei, L., Jing, Z., 2016. Long-term grazing exclusion effects on vegetation characteristics, soil properties and bacterial communities in the semi-arid grasslands of China. Ecological engineering: The Journal of Ecotechnology 97, 170–178. https://doi.org/10.1016/j.ecoleng.2016.09.003.

Cheng, Y., An, S. S., Li, G.H., Li, D.H., 2010. Effects of vegetation restoration on soil nutrients and microbial biomass in loess hilly region of Ningxia (in China). Journal of China Ecological Agriculture 18(02), 261-266. <https://doi.org/10.3724/SP.J.1011.2010.00261>.

Cui, Y., Fang, L., Guo, X., Wang, X., Wang, Y., Zhang, Y., Zhang, X., 2018. Responses of soil bacterial communities, enzyme activities, and nutrients to agricultural-to-natural ecosystem conversion in the Loess Plateau, China. Journal of Soils & Sediments 19, 1–14. https://doi.org/10.1007/s11368-018-2110-4.

Deng, L., Peng, C., Huang, C., Wang, K., Shangguan, Z., 2019. Drivers of soil microbial metabolic limitation changes along a vegetation restoration gradient on the Loess Plateau, China. Geoderma 353, 188–200. https://doi.org/10.1016/j.geoderma.2019.06.037.

Deng, L., Zhang, Z.N., Shangguan, Z.P., 2014. Long-term fencing effects on plant diversity and soil properties in China. Soil Till Res 137, 7–15. https://doi.org/10.1016/j.still.2013.11.002.

Duan, C.W., Li, X.L., Chai, Y., Xu, W.Y., Su, L.L., Yang, X.G., Ma, P.P., 2022. Eco-stoichiometric characteristics of carbon, nitrogen and phosphorus in degraded alpine meadow under artificial restoration (in China). Journal of Grassland of China 07, 23-32. https://doi.org/10.16742/j.zgcdxb.20210298.

Ebrahimi, M., Khosravi, H., Rigi, M., 2016. Short-term grazing exclusion from heavy livestock rangelands affects vegetation cover and soil properties in natural ecosystems of southeastern Iran. Ecological Engineering 95, 10–18. https://doi.org/10.1016/j.ecoleng.2016.06.069.

Gao, X., Dong, S., Xu, Y., Li, Y., Li, S., Wu, S., Shen, H., Liu, S., Fry, E.L., 2021. Revegetation significantly increased the bacterial-fungal interactions in different successional stages of alpine grasslands on the Qinghai-Tibetan Plateau. Catena 205, 105385. https://doi.org/10.1016/j.catena.2021.105385.

Ge, P., Cai, T., Da. L.J., Cui, Y.Y., Wang, Y.H., Yu, F.D., 2014. Evolution of soil nutrients and microorganisms in *Pinus massoniana* secondary forest at different restoration stages (in China). Chinese agricultural science bulletin 22, 22-27. http://www.casb.org.cn.

Guo, J., Jiang, H., Bian, H., Sheng, L., He, C., Gao, Y., 2017. Natural succession is a feasible approach for cultivated peatland restoration in Northeast China. Ecological Engineering 104, 39–44. https://doi.org/10.1016/j.ecoleng.2017.04.001.

Guo, N., Degen, A.A., Deng, B., Shi, F., Shang, Z., 2019. Changes in vegetation parameters and soil nutrients along degradation and recovery successions on alpine grasslands of the Tibetan plateau. Agriculture, Ecosystems & Environment 284, 106593. https://doi.org/10.1016/j.agee.2019.106593.

Han, C., Liang, D., Zhou, W., Xu, Q., Xiang, M., Gu, Y., Siddique, K.H.M., 2024. Soil, Plant, and Microorganism Interactions Drive Secondary Succession in Alpine Grassland Restoration. Plants-Basel 13, 780. https://doi.org/10.3390/plants13060780.

Hou, X., Han, H., Tigabu, M., Cai, L., Ma, X., 2019. Changes in soil physico-chemical properties following vegetation restoration mediate bacterial community composition and diversity in Changting, China. Ecological Engineering 138, 171–179. https://doi.org/10.1016/j.ecoleng.2019.07.031.

Hu, F., Du, H., Zeng, F.P., Song, T.Q., Peng, W.X., Zhang, F., 2018. Effects of different vegetation restoration on soil nutrient content and microbial diversity in typical karst peak-cluster depressions (in China). Acta Ecologica Sinica 06, 2170-2179. https://doi.org/10.5846 /stxb201703180458.

Hu, H.Y., Zhang, Y.L., Chu, Z.P., Huang, C.F., Wang, S.Z., Zhou, C.F., 2021. Changes of soil enzyme activities and microbial diversity in different vegetation restoration stages in red soil erosion areas (in China). Journal of Applied and Environmental Biology 03, 734-741. https://doi.org/10.19675/j.cnki.1006-687x.2020.

Hu, J.J., Zhou, Q., Cao, Q., Hu, J, 2022. Effects of ecological restoration measures on vegetation and soil properties in semi-humid sandy land on the southeast Qinghai-Tibetan Plateau, China. Glob. Ecol. Conserv. 33, e02000. https://doi.org/10.1016/j.gecco.2022.e02000.

Hu, M., Sardans, J., Sun, D., Yan, R., Wu, H., Ni, R., Peuelas, J., 2024. Microbial diversity and keystone species drive soil nutrient cycling and multifunctionality following mangrove restoration. Environmental Research 251, 118715. https://doi.org/10.1016/j.envres.2024.118715.

Jing, Z., Cheng, J., Su, J., Bai, Y., Jin, J., 2014. Changes in plant community composition and soil properties under 3-decade grazing exclusion in semiarid grassland. Ecological Engineering 64, 171–178. https://doi.org/10.1016/j.ecoleng.2013.12.023.

Li, Q., Zhou, D., Jin, Y., Wang, M., Song, Y., Li, G., 2014. Effects of fencing on vegetation and soil restoration in a degraded alkaline grassland in northeast China. Journal of Arid Land 6, 478-487. https://doi.org/10.1007/s40333-013-0207-6.

Li, W., Liu, Y., Wang, J., Shi, S., Cao, W., 2018. Six years of grazing exclusion is the optimum duration in the alpine meadow-steppe of the north-eastern Qinghai-Tibetan Plateau. Sci Rep 8, 17269. https://doi.org/10.1038/s41598-018-35273-y.

Li, X.N., Wang, C., Zhang, W.W., Zhao, C.Q., Shi, R.S., Xue, R.B., Fan, R., 2019. Effects of short-term vegetation restoration on soil physical and chemical properties and microbial community structure in wasteland of Beijing suburbs (in China). Journal of Soil and Water Conservation 05, 343-348+357. https://doi.org/10.13870/j.cnki.stbcxb.2019.05.050.

Li, Y., Shi, C., Wei, D., Ding, J., Xu, N., Jin, L., Wang, L., 2023. Associations of soil bacterial diversity and function with plant diversity in *Carex* tussock wetland. Front. Microbiol. 14, 1142052. https://doi.org/10.3389/fmicb.2023.1142052.

Li, Y.R., Hu, S., Lang, S., Pu, Y., Zhang, S., Li, T., Xu, X., Jia, Y., Wang, G., Yuan, D., Li, Yun, 2023. Soil quality and ecological benefits assessment of alpine desertified grassland following different ecological restoration measures. Front. Plant Sci. 14, 1283457. https://doi.org/10.3389/fpls.2023.1283457.

Liao, H., Zheng, C., Li, J., Long, J., 2018. Dynamics of soil microbial recovery from cropland to orchard along a 20-year chronosequence in a degraded karst ecosystem. Sci. Total Environ. 639, 1051–1059. https://doi.org/10.1016/j.scitotenv.2018.05.246.

Liu, D., Huang, Y.M., An, S.S., 2012. Changes of soil nitrogen and microbial activity during the restoration of artificial *Robinia pseudoacacia* forest in loess hilly region (in China). Journal of China Ecological Agriculture 20 (03), 322-329. https://doi.org/10.3724/SP.J.1011.2012.00322.

Liu, G., Bai, Z., Shah, F., Cui, G., Xiao, Z., Gong, H., Li, D., Lin, Y., Li, B., Ji, G., Shah, S., 2021. Compositional and structural changes in soil microbial communities in response to straw mulching and plant revegetation in an abandoned artificial pasture in Northeast China. Glob. Ecol. Conserv. 31, 01871. https://doi.org/10.1016/j.gecco.2021.e01871.

Liu, H.F., Liang C.T., Ai, Z., Zhang, J.Y., Wu, Y., Xue, S., Liu, G.B., 2019. Plant‐mycorrhizae association affects plant diversity, biomass, and soil nutrients along temporal gradients of natural restoration after farmland abandonment in the Loess Plateau, China. Land Degradation and Development 30, 1677-1690. https://doi.org/10.1002/ldr.3372.

Liu, J., Bian, Z., Zhang, K., Ahmad, B., Khan, A., 2019. Effects of different fencing regimes on community structure of degraded desert grasslands on Mu Us desert, China. Ecol. Evol. 9, 3367–3377. https://doi.org/10.1002/ece3.4958.

Liu, W., Fu, S., Yan, S., Ren, C., Wu, S., Deng, J., Li, B., Han, X., Yang, G., 2020. Responses of plant community to the linkages in plant-soil C: N: P stoichiometry during secondary succession of abandoned farmlands, China. J. Arid Land 12, 215–226. https://doi.org/10.1007/s40333-020-0009-6.

Liu, Y., Hou, Z.S., Zhao, S., Qi, G.H., Zhang, X.M., Guo, S.P., 2018. Evolution of plant diversity and soil characteristics in the process of vegetation restoration in gneiss mountain area of Taihang Mountain (in China). Journal of Ecology 38 (15), 5331-5339. https://doi.org/10.5846 /stxb201709271749.

Miao, R., Jiang, D., Musa, A., Zhou, Q., Guo, M., Wang, Y., 2015. Effectiveness of shrub planting and grazing exclusion on degraded sandy grassland restoration in Horqin sandy land in Inner Mongolia. Ecol. Eng. 74, 164–173. https://doi.org/10.1016/j.ecoleng.2014.10.004.

Mir, A.H., Dad, J.M., Singh, B., Kamili, A.N., 2022. Passive restoration considerably improved the community structure, soil health and carbon stock in the Pine forests of Kashmir Himalaya, India. Ecol. Eng. 176, 106535. https://doi.org/10.1016/j.ecoleng.2021.106535.

Pan, Y., Kang, P., Qu, X., Ran, Y., Li, X., 2024. Effects of long-term fencing on soil microbial community structure and function in the desert steppe, China. Journal of Arid LandIssue 16 (3), 431-446. https://doi.org/10.1007/s40333-024-0009-z.

Rong, Y., Yuan, F., Ma, L., 2014. Effectiveness of exclosures for restoring soils and vegetation degraded by overgrazing in the Junggar Basin, China. Grassland Science 60, 118–124. https://doi.org/10.1111/grs.12048.

Song, D.C., Wu, H., Wang, L.D., He, F.L., Zhao, X.R., Han, S.H., Xu, B.Y., 2021. Variation characteristics of soil microorganisms and soil enzyme activities in secondary grassland in Minqin area (in China). Journal of Grassland of China 43 (06), 85-93. https://doi.org/06,85-93.10.16742/j.zgcdxb.20200224.

Song, X.C., Wang, H.L., Qin, W.D., Deng, X.J., Tian, H.D., Tan, Y.B., Cao, J.Z., 2019. Effects of different restoration types of degraded plantations on functional diversity of soil microbial communities (in China). Acta Applied Ecology 03, 841-848. <https://doi.org/10.13287/j.1001-9332.201903.036>.

Su, J., Jing, G., Jin, J., Wei, L., Liu, J., Cheng, J., 2017. Identifying drivers of root community compositional changes in semiarid grassland on the Loess plateau after long-term grazing exclusion. Ecological engineering: The Journal of Ecotechnology 99, 13–21. https://doi.org/10.1016/j.ecoleng.2016.11.050.

Sun, D., Huang, Y., Wang, Z., Tang, X., Ye, W., Cao, H., Shen, H., 2024. Soil microbial community structure, function and network along a mangrove forest restoration chronosequence. Sci. Total Environ. 913, 169704. https://doi.org/10.1016/j.scitotenv.2023.169704.

Tao, J.Y., Tan, J.L., Zheng, F.L., Wang, X.N., Zhang, W.J., 2022. Effects of vegetation restoration model on soil main enzyme activities, microbial diversity and soil nutrients in southern Ningxia (in China). Agricultural Research in Arid Areas 03, 207-217. https://doi.org/10.7606/j.issn.1000-7601.2022.03.25.

Wang, A.N., Huang, Q.X., Li, X.G., Xu, X.H., Li, Y.L., 2019. Bacterial community structure and diversity in rhizosphere soil of different vegetation restoration types in northern Hebei Province (in China). Forestry Science 09, 130-141. https://doi.org/10.11707/j.1001-7488.20190914.

Wang, J., Wang, X., Liu, G., Wang, G., Zhang, C., 2020. Fencing as an effective approach for restoration of alpine meadows: Evidence from nutrient limitation of soil microbes. Geoderma 363, 114148. https://doi.org/10.1016/j.geoderma.2019.114148.

Wang, J., Wang, X., Liu, G., Wang, G., Zhang, C., 2021. Grazing-to-fencing conversion affects soil microbial composition, functional profiles by altering plant functional groups in a Tibetan alpine meadow. Applied Soil Ecology 166, 104008. https://doi.org/10.1016/j.apsoil.2021.104008.

Wang, X., Song, N.P., Yang, X.G., Yang, M.X., Xiao, X.P., 2013. Response of grassland plant diversity to soil factors under grazing disturbance (in China). Acta prataculture 05, 27-36. https://doi.org/10. 11686/ cyxb20130504.

Wang, Z., Li, X., Ji, B., Struik, P.C., Tang, S., 2021. Coupling Between the Responses of Plants, Soil, and Microorganisms Following Grazing Exclusion in an Overgrazed Grassland. Frontiers in Plant Science 12, 640789. https://doi.org/10.3389/fpls.2021.640789.

Wang, Z., Zhang, Q., Staley, C., Gao, H., Ishii, S., Wei, X., Liu, J., Cheng, J., Hao, M., Sadowsky, M.J., 2019. Impact of long-term grazing exclusion on soil microbial community composition and nutrient availability. Biology and Fertility of Soils 55, 121–134. https://doi.org/10.1007/s00374-018-01336-5.

Wu, C.D., Meng, Z.J., Liu, M.H., Meng, X.Z., Zhou, C.L., 2024. Plant diversity and its relationship with soil chemical factors in the wetland of Naoli River Nature Reserve (in China). Journal of Jiangxi Agricultural University 04, 969-979. https://doi.org/10.3724/aauj.2024086.

Wu, X.Y., Lu, H., Wang, X.J., 2007. Changes of plant diversity, biomass and soil nutrient content after natural restoration of cut-off land of sand-fixing forest (in China). Journal of Ecology 07, 978-982. https://doi.org/10.13292/j.1000-4890.2007.0176.

Xiang, K.X., Zhang, X., Liu, J.M., Xue, J.H., Cui, Y.C., Wu, Y.B., 2019. Correlation between plant diversity index of cypress forest and soil physical and chemical indexes in Shimo District (in China). Journal of south China agriculture 08,1771-1778. https://doi.org/10.3969/j.issn.2095-1191.2019.08.17.

Xu, X., Cui, F., Fu, L., Fan, P., Wang, M., Cai, Z., 2024. A pioneer tree species rapidly facilitating ecosystem restoration in coastal regions depends on soil traits. Catena 238, 107825. https://doi.org/10.1016/j.catena.2024.107825.

Yan, B., Sun, L., Li, J., Liang, C., Wei, F., Xue, S., Wang, G., 2020. Change in composition and potential functional genes of soil bacterial and fungal communities with secondary succession in Quercus liaotungensis forests of the Loess Plateau, western China. Geoderma 364, 114199. https://doi.org/10.1016/j.geoderma.2020.114199.

Yan, C.L., Xue, Y., Wang, Y.F., Kang, H.B., Wang, D.X., 2024. Changes of plant diversity and its driving factors in abandoned farmland at different restoration stages in the middle Qinling Mountains (in China). Environmental Science 02, 992-1003. <https://doi.org/10.13227/j.hjkx.202303002>.

Yang, F., Huang, M., Li, C., Wu, X., Fang, L., 2022. Vegetation restoration increases the diversity of bacterial communities in deep soils. Appl. Soil Ecol. 180, 104631. https://doi.org/10.1016/j.apsoil.2022.104631.

Yang, S., Feng, C., Ma, Y., Wang, W., Huang, C., Qi, C., Fu, S., Chen, H.Y.H., 2021. Transition from N to P limited soil nutrients over time since restoration in degraded subtropical broadleaved mixed forests. For. Ecol. Manage. 494, 119298. https://doi.org/10.1016/j.foreco.2021.119298.

Yang, Y., Liu, H., Yang, X., Yao, H., Deng, X., Wang, Y., An, S., Kuzyakov, Y., Chang, S.X., 2022. Plant and soil elemental C: N: P ratios are linked to soil microbial diversity during grassland restoration on the Loess Plateau, China. Sci. Total Environ. 806, 150557. https://doi.org/10.1016/j.scitotenv.2021.150557.

Yang, Z., Xiong, W., Xu, Y., Jiang, L., Chen, H., 2016. Soil properties and species composition under different grazing intensity in an alpine meadow on the eastern Tibetan Plateau, China. Environmental Monitoring and Assessment 188, 678. https://doi.org/10.1007/s10661-016-5663-y.

Yin, Y.L., Wang, Y.Q., Li, S.X., Liu, Y., Zhao, W., Ma, Y.S., Bao, G.S., 2019. Effects of enclosure on soil microbial community diversity and soil stoichiometry characteristics in degraded alpine meadow (in China). Journal of Applied Ecology 01, 127-136. https://doi.org/10.13287/j.1001-9332.201901.009.

Yuan, J., Ouyang, Z., Zheng, H., Xu, W., 2012. Effects of different grassland restoration approaches on soil properties in the southeastern Horqin sandy land, northern China. Applied Soil Ecology 61, 34–39. https://doi.org/10.1016/j.apsoil.2012.04.003.

Yuan, Z., Epstein, H., Li, G., 2020. Grazing exclusion did not affect soil properties in alpine meadows in the Tibetan permafrost region. Ecological Engineering 147, 105657. https://doi.org/10.1016/j.ecoleng.2019.105657.

Zhang, C., Li, J., Wang, J., Liu, G., Wang, G., Guo, L., Peng, S., 2019. Decreased temporary turnover of bacterial communities along soil depth gradient during a 35-year grazing exclusion period in a semiarid grassland. Geoderma 351, 49–58. https://doi.org/10.1016/j.geoderma.2019.05.010.

Zhang, C., Liu, G., Xue, S., Wang, G., 2016. Soil bacterial community dynamics reflect changes in plant community and soil properties during the secondary succession of abandoned farmland in the Loess Plateau. Soil Biol. Biochem. 97, 40–49. https://doi.org/10.1016/j.soilbio.2016.02.013.

Zhang, P.J., Xu, J.M., Lu, W.H., Pan, S.H., Chen, M.X., Li, K.S., Shang, X.H., 2021. Analysis of Plant Diversity and Soil Physical and Chemical Properties of *Eucalyptus urophylla* Plantation in Leizhou Peninsula (in China). Journal of Central South University of Forestry and Technology 09, 96-105. https://doi.org/10.14067/j.cnki.1673-923x.2021.09.011.

Zhang, W., Xu, Y., Gao, D., Wang, X., Liu, W., Deng, J., Han, X., Yang, G., Feng, Y., Ren, G., 2019. Ecoenzymatic stoichiometry and nutrient dynamics along a revegetation chronosequence in the soils of abandoned land and Robinia pseudoacacia plantation on the Loess Plateau, China. Soil Biology and Biochemistry 134, 1–14. https://doi.org/10.1016/j.soilbio.2019.03.017.

Zhang, X.Y., Du, W.B., Zhang, X.P., Wang, J.X., Li, D.Y., Cai, X.H., 2007. Evaluation of soil microorganisms in the process of vegetation restoration in western Sichuan and its relationship with soil factors (in China). Ecological environment 05, 1470-1474. https://doi.org/10.16258/j.cnki.1674-5906.2007.05.

Zhang, Y., Wang, G., Gou, Q., Zhang, Y., Liu, J., Gao, M., 2023. Succession of a natural desert vegetation community after long-term fencing at the edge of a desert oasis in northwest China. Front. Plant Sci. 14, 1091446. https://doi.org/10.3389/fpls.2023.1091446.

Zhang, Y., Zhao, W., 2015. Vegetation and soil property response of short-time fencing in temperate desert of the Hexi Corridor, northwestern China. Catena 133, 43–51. https://doi.org/10.1016/j.catena.2015.04.019.

Zhang, Z., Han, X., Yan, J., Zou, W., Wang, E., Lu, X., Chen, X., 2020. Keystone Microbiomes Revealed by 14 Years of Field Restoration of the Degraded Agricultural Soil Under Distinct Vegetation Scenarios. Front. Microbiol. 11, 1915. https://doi.org/10.3389/fmicb.2020.01915.

Zheng, X., Yan, M., Lin, C., Guo, B., Ding, H., Yu, J., Peng, S., Sveen, T.R., Zhang, Y., 2022. Vegetation restoration types affect soil bacterial community composition and diversity in degraded lands in subtropical of China. Restor. Ecol. 30, e13494. https://doi.org/10.1111/rec.13494.

Zhong, Z., Zhang, X., Wang, X., Fu, S., Wu, S., Lu, X., Ren, C., Han, X., Yang, G., 2020. Soil bacteria and fungi respond differently to plant diversity and plant family composition during the secondary succession of abandoned farmland on the Loess Plateau, China. Plant and Soil 448, 183–200. https://doi.org/10.1007/s11104-019-04415-0.

Zhu, G.Y., Deng, L., Zhang, X.B., Shangguan, Z.P., 2016. Effects of grazing exclusion on plant community and soil physicochemical properties in a desert steppe on the Loess Plateau, China. Ecological engineering: The Journal of Ecotechnology 90, 372–381. https://doi.org/10.1016/j.ecoleng.2016.02.001.

| **ID** | **Study** | **Recovery type** | **System type** | **Recovery period** | **Longitude** | **Latitude** | **TP** | **Bias** | **TP** | **Bias** | **AP** | **Bias** | **AP** | **Bias** | **MBP** | **Bias** | **MBP** | **Bias** | **AGB** | **Bias** | **AGB** | **Bias** | **BGB** | **Bias** | **BGB** | **Bias** | **LI** | **Bias** | **LI** | **Bias** | **PR** | **Bias** | **PR** | **Bias** | **PS** | **Bias** | **PS** | **Bias** | **PSI** | **Bias** | **PSI** | **Bias** |
| --- | --- | --- | --- | --- | --- | --- | --- | --- | --- | --- | --- | --- | --- | --- | --- | --- | --- | --- | --- | --- | --- | --- | --- | --- | --- | --- | --- | --- | --- | --- | --- | --- | --- | --- | --- | --- | --- | --- | --- | --- | --- | --- |
| 1 | 1 | Ecological restoration | Forest | 0-10 | 30.04 | 117.53 | 0.170 | 0.037 | 0.215 | #### | 6.310 | 0.020 | 5.570 | 0.030 |  |  |  |  |  |  |  |  |  |  |  |  | ##### | 50.39 | 978.10 | 34.83 |  |  |  |  |  |  |  |  |  |  |  |  |
| 2 | 1 | Ecological restoration | Forest | 0-10 | 30.04 | 117.53 | 0.130 | 0.034 | 0.181 | #### | 4.030 | 0.030 | 2.010 | 0.030 |  |  |  |  |  |  |  |  |  |  |  |  | ##### | 50.39 | 978.10 | 34.83 |  |  |  |  |  |  |  |  |  |  |  |  |
| 3 | 1 | Ecological restoration | Forest | ＞20 | 30.04 | 117.53 | 0.140 | 0.031 | 0.137 | #### | 8.390 | 0.180 | 6.260 | 0.120 |  |  |  |  |  |  |  |  |  |  |  |  | ##### | 59.61 | ##### | 78.13 |  |  |  |  |  |  |  |  |  |  |  |  |
| 4 | 1 | Ecological restoration | Forest | ＞20 | 30.04 | 117.53 | 0.120 | 0.039 | 0.114 | #### | 4.390 | 0.260 | 3.230 | 0.190 |  |  |  |  |  |  |  |  |  |  |  |  | ##### | 59.61 | ##### | 78.13 |  |  |  |  |  |  |  |  |  |  |  |  |
| 5 | 1 | Ecological restoration | Forest | ＞20 | 30.04 | 117.53 | 0.145 | 0.037 | 0.158 | #### | 10.580 | 0.270 | 8.140 | 0.240 |  |  |  |  |  |  |  |  |  |  |  |  | ##### | 68.37 | ##### | 69.49 |  |  |  |  |  |  |  |  |  |  |  |  |
| 6 | 1 | Ecological restoration | Forest | ＞20 | 30.04 | 117.53 | 0.129 | 0.030 | 0.121 | #### | 7.230 | 0.160 | 5.110 | 0.020 |  |  |  |  |  |  |  |  |  |  |  |  | ##### | 68.37 | ##### | 69.49 |  |  |  |  |  |  |  |  |  |  |  |  |
| 7 | 2 | Ecological restoration | Forest | 0-10 | 30.64 | 103.68 | 0.131 | 0.030 | 0.114 | #### | 8.370 | 0.240 | 8.310 | 0.340 |  |  |  |  |  |  |  |  |  |  |  |  |  |  |  |  |  |  |  |  | 1.680 | 0.170 | 6.630 | 0.670 |  |  |  |  |
| 8 | 2 | Ecological restoration | Forest | 0-10 | 30.64 | 103.68 | 0.131 | 0.030 | 0.114 | #### | 10.310 | 0.360 | 8.310 | 0.340 |  |  |  |  |  |  |  |  |  |  |  |  |  |  |  |  |  |  |  |  | 2.880 | 0.290 | 3.340 | 0.330 |  |  |  |  |
| 9 | 2 | Ecological restoration | Forest | 10-20 | 30.64 | 103.68 | 0.131 | 0.030 | 0.114 | #### | 8.370 | 0.240 | 8.310 | 0.340 |  |  |  |  |  |  |  |  |  |  |  |  |  |  |  |  |  |  |  |  | 1.680 | 0.170 | 2.940 | 0.290 |  |  |  |  |
| 10 | 2 | Ecological restoration | Forest | 10-20 | 30.64 | 103.68 | 0.131 | 0.030 | 0.104 | #### | 10.310 | 0.360 | 6.370 | 0.640 |  |  |  |  |  |  |  |  |  |  |  |  |  |  |  |  |  |  |  |  | 2.880 | 0.290 | 1.350 | 0.140 |  |  |  |  |
| 11 | 2 | Ecological restoration | Forest | 10-20 | 30.64 | 103.68 | 0.131 | 0.030 | 0.104 | #### | 8.370 | 0.240 | 6.370 | 0.640 |  |  |  |  |  |  |  |  |  |  |  |  |  |  |  |  |  |  |  |  | 1.680 | 0.170 | 4.040 | 0.400 |  |  |  |  |
| 12 | 2 | Ecological restoration | Forest | 10-20 | 30.64 | 103.68 | 0.131 | 0.030 | 0.104 | #### | 10.310 | 0.360 | 6.370 | 0.640 |  |  |  |  |  |  |  |  |  |  |  |  |  |  |  |  |  |  |  |  | 2.880 | 0.290 | 2.840 | 0.280 |  |  |  |  |
| 13 | 2 | Ecological restoration | Forest | ＞20 | 30.64 | 103.68 | 0.131 | 0.030 | 0.110 | #### | 8.370 | 0.240 | 9.570 | 0.340 |  |  |  |  |  |  |  |  |  |  |  |  |  |  |  |  |  |  |  |  | 1.680 | 0.170 | 7.290 | 0.730 |  |  |  |  |
| 14 | 2 | Ecological restoration | Forest | ＞20 | 30.64 | 103.68 | 0.131 | 0.030 | 0.110 | #### | 10.310 | 0.360 | 9.570 | 0.340 |  |  |  |  |  |  |  |  |  |  |  |  |  |  |  |  |  |  |  |  | 2.880 | 0.290 | 2.110 | 0.210 |  |  |  |  |
| 15 | 2 | Ecological restoration | Forest | ＞20 | 30.64 | 103.68 | 0.131 | 0.030 | 0.110 | #### | 8.370 | 0.240 | 9.570 | 0.340 |  |  |  |  |  |  |  |  |  |  |  |  |  |  |  |  |  |  |  |  | 1.680 | 0.170 | 2.890 | 0.290 |  |  |  |  |
| 16 | 2 | Ecological restoration | Forest | ＞20 | 30.64 | 103.68 | 0.131 | 0.030 | 0.103 | #### | 10.310 | 0.360 | 4.390 | 0.310 |  |  |  |  |  |  |  |  |  |  |  |  |  |  |  |  |  |  |  |  | 2.880 | 0.290 | 9.550 | 0.960 |  |  |  |  |
| 17 | 2 | Ecological restoration | Forest | ＞20 | 30.64 | 103.68 | 0.131 | 0.030 | 0.103 | #### | 8.370 | 0.240 | 4.390 | 0.310 |  |  |  |  |  |  |  |  |  |  |  |  |  |  |  |  |  |  |  |  | 1.680 | 0.170 | 3.680 | 0.370 |  |  |  |  |
| 18 | 2 | Ecological restoration | Forest | ＞20 | 30.64 | 103.68 | 0.131 | 0.030 | 0.103 | #### | 10.310 | 0.360 | 4.390 | 0.310 |  |  |  |  |  |  |  |  |  |  |  |  |  |  |  |  |  |  |  |  | 2.880 | 0.290 | 1.110 | 0.110 |  |  |  |  |
| 19 | 2 | Ecological restoration | Forest | ＞20 | 30.64 | 103.68 | 0.131 | 0.030 | 0.096 | 0.031 | 8.370 | 0.240 | 5.380 | 0.610 |  |  |  |  |  |  |  |  |  |  |  |  |  |  |  |  |  |  |  |  | 1.680 | 0.170 | 10.090 | 1.010 |  |  |  |  |
| 20 | 2 | Ecological restoration | Forest | ＞20 | 30.64 | 103.68 | 0.131 | 0.030 | 0.096 | 0.031 | 10.310 | 0.360 | 5.380 | 0.610 |  |  |  |  |  |  |  |  |  |  |  |  |  |  |  |  |  |  |  |  | 2.880 | 0.290 | 3.650 | 0.370 |  |  |  |  |
| 21 | 2 | Ecological restoration | Forest | ＞20 | 30.64 | 103.68 | 0.131 | 0.030 | 0.096 | 0.031 | 8.370 | 0.240 | 5.380 | 0.610 |  |  |  |  |  |  |  |  |  |  |  |  |  |  |  |  |  |  |  |  | 1.680 | 0.170 | 2.980 | 0.300 |  |  |  |  |
| 22 | 3 | Ecological restoration | Forest | 0-10 | 25.21 | 108.10 | 2.990 | 1.005 | 4.110 | 1.230 | ##### | 36.280 | 31.920 | #### | 10.39 | 0.360 | 13.64 | 0.220 |  |  |  |  |  |  |  |  |  |  |  |  |  |  |  |  |  |  |  |  |  |  |  |  |
| 23 | 3 | Ecological restoration | Forest | 0-10 | 25.21 | 108.10 | 2.990 | 1.005 | 3.910 | #### | ##### | 36.280 | 34.480 | #### | 19.37 | 0.190 | 21.07 | 0.780 |  |  |  |  |  |  |  |  |  |  |  |  |  |  |  |  |  |  |  |  |  |  |  |  |
| 24 | 3 | Ecological restoration | Forest | 10-20 | 25.21 | 108.10 | 2.990 | 1.005 | 3.420 | 1.440 | ##### | 36.280 | 20.760 | #### | 18.36 | 0.570 | 24.55 | 0.260 |  |  |  |  |  |  |  |  |  |  |  |  |  |  |  |  |  |  |  |  |  |  |  |  |
| 25 | 3 | Ecological restoration | Forest | 10-20 | 25.21 | 108.10 | 2.990 | 1.005 | 3.610 | 1.570 | ##### | 36.280 | 19.740 | #### | 20.36 | 0.370 | 10.01 | 0.450 |  |  |  |  |  |  |  |  |  |  |  |  |  |  |  |  |  |  |  |  |  |  |  |  |
| 26 | 3 | Ecological restoration | Forest | ＞20 | 25.21 | 108.10 | 2.990 | 1.005 | 3.330 | 1.710 | ##### | 36.280 | 30.230 | #### | 19.36 | 0.690 | 13.21 | 0.380 |  |  |  |  |  |  |  |  |  |  |  |  |  |  |  |  |  |  |  |  |  |  |  |  |
| 27 | 3 | Ecological restoration | Forest | ＞20 | 25.21 | 108.10 | 2.990 | 1.005 | 3.610 | 1.970 | ##### | 36.280 | 13.320 | 12.160 | 15.37 | 0.670 | 20.16 | 0.290 |  |  |  |  |  |  |  |  |  |  |  |  |  |  |  |  |  |  |  |  |  |  |  |  |
| 28 | 4 | Ecological restoration | Forest | 0-10 | 25.55 | 116.52 |  |  |  |  | 7.020 | 0.610 | 11.760 | 4.031 |  |  |  |  |  |  |  |  |  |  |  |  |  |  |  |  |  |  |  |  |  |  |  |  |  |  |  |  |
| 29 | 4 | Ecological restoration | Forest | 10-20 | 25.55 | 116.52 |  |  |  |  | 7.020 | 0.610 | 8.800 | 0.277 |  |  |  |  |  |  |  |  |  |  |  |  |  |  |  |  |  |  |  |  |  |  |  |  |  |  |  |  |
| 30 | 4 | Ecological restoration | Forest | ＞20 | 25.55 | 116.52 |  |  |  |  | 7.020 | 0.610 | 9.390 | 1.228 |  |  |  |  |  |  |  |  |  |  |  |  |  |  |  |  |  |  |  |  |  |  |  |  |  |  |  |  |
| 31 | 4 | Ecological restoration | Forest | ＞20 | 25.55 | 116.52 |  |  |  |  | 7.020 | 0.610 | 8.850 | 0.865 |  |  |  |  |  |  |  |  |  |  |  |  |  |  |  |  |  |  |  |  |  |  |  |  |  |  |  |  |
| 44 | 6 | Ecological restoration | Forest | 10-20 | 41.47 | 116.21 |  |  |  |  | 7.460 | 0.069 | 5.580 | 0.104 | 4.10 | 2.509 | 5.91 | 0.813 |  |  |  |  |  |  |  |  |  |  |  |  |  |  |  |  |  |  |  |  |  |  |  |  |
| 45 | 6 | Ecological restoration | Forest | 10-20 | 41.47 | 116.21 |  |  |  |  | 7.460 | 0.069 | 9.000 | 0.554 | 4.10 | 2.509 | 7.84 | 2.076 |  |  |  |  |  |  |  |  |  |  |  |  |  |  |  |  |  |  |  |  |  |  |  |  |
| 46 | 6 | Ecological restoration | Forest | 10-20 | 41.47 | 116.21 |  |  |  |  | 7.460 | 0.069 | 5.870 | 0.761 | 4.10 | 2.509 | 11.54 | 2.111 |  |  |  |  |  |  |  |  |  |  |  |  |  |  |  |  |  |  |  |  |  |  |  |  |
| 47 | 6 | Ecological restoration | Forest | 10-20 | 41.47 | 116.21 |  |  |  |  | 7.460 | 0.069 | 14.540 | 0.208 | 4.10 | 2.509 | 27.49 | 0.363 |  |  |  |  |  |  |  |  |  |  |  |  |  |  |  |  |  |  |  |  |  |  |  |  |
| 48 | 6 | Ecological restoration | Forest | 10-20 | 41.47 | 116.21 |  |  |  |  | 6.850 | 0.225 | 5.410 | 0.035 | 1.11 | 0.017 | 2.71 | 0.813 |  |  |  |  |  |  |  |  |  |  |  |  |  |  |  |  |  |  |  |  |  |  |  |  |
| 49 | 6 | Ecological restoration | Forest | 10-20 | 41.47 | 116.21 |  |  |  |  | 6.850 | 0.225 | 7.460 | 0.796 | 1.11 | 0.017 | 4.68 | 1.142 |  |  |  |  |  |  |  |  |  |  |  |  |  |  |  |  |  |  |  |  |  |  |  |  |
| 50 | 6 | Ecological restoration | Forest | ＞20 | 41.47 | 116.21 |  |  |  |  | 6.850 | 0.225 | 5.510 | 0.173 | 1.11 | 0.017 | 3.81 | 0.813 |  |  |  |  |  |  |  |  |  |  |  |  |  |  |  |  |  |  |  |  |  |  |  |  |
| 51 | 6 | Ecological restoration | Forest | ＞20 | 41.47 | 116.21 |  |  |  |  | 6.850 | 0.225 | 12.170 | 0.069 | 1.11 | 0.017 | 21.17 | 0.121 |  |  |  |  |  |  |  |  |  |  |  |  |  |  |  |  |  |  |  |  |  |  |  |  |
| 52 | 6 | Ecological restoration | Forest | ＞20 | 41.47 | 116.21 |  |  |  |  | 6.580 | 0.260 | 4.300 | 0.104 | 0.22 | 0.225 | 1.28 | 0.433 |  |  |  |  |  |  |  |  |  |  |  |  |  |  |  |  |  |  |  |  |  |  |  |  |
| 55 | 8 | Ecological restoration | Grassland | 0-10 | 39.03 | 104.58 |  |  |  |  | 6.580 | 0.260 | 7.350 | 1.055 | 0.22 | 0.225 | 7.13 | 0.727 |  |  |  |  |  |  |  |  |  |  |  |  |  |  |  |  |  |  |  |  |  |  |  |  |
| 56 | 8 | Ecological restoration | Grassland | 0-10 | 39.03 | 104.58 |  |  |  |  | 6.580 | 0.260 | 4.550 | 0.017 | 0.22 | 0.225 | 1.69 | 0.017 |  |  |  |  |  |  |  |  |  |  |  |  |  |  |  |  |  |  |  |  |  |  |  |  |
| 57 | 8 | Ecological restoration | Grassland | 0-10 | 39.03 | 104.58 |  |  |  |  | 6.580 | 0.260 | 9.080 | 0.156 | 0.22 | 0.225 | 11.42 | 0.190 |  |  |  |  |  |  |  |  |  |  |  |  |  |  |  |  |  |  |  |  |  |  |  |  |
| 58 | 8 | Ecological restoration | Grassland | 0-10 | 39.03 | 104.58 | 1.310 | 0.050 | 0.280 | 0.010 |  |  |  |  |  |  |  |  |  |  |  |  |  |  |  |  |  |  |  |  | 5.09 | 0.17 | 5.33 | 0.11 |  |  |  |  |  |  |  |  |
| 59 | 8 | Ecological restoration | Grassland | 10-20 | 39.03 | 104.58 | 1.310 | 0.050 | 0.340 | #### |  |  |  |  |  |  |  |  |  |  |  |  |  |  |  |  |  |  |  |  | 5.09 | 0.17 | ##### | 0.1100 |  |  |  |  |  |  |  |  |
| 60 | 8 | Ecological restoration | Grassland | 10-20 | 39.03 | 104.58 | 1.310 | 0.050 | 0.620 | #### |  |  |  |  |  |  |  |  |  |  |  |  |  |  |  |  |  |  |  |  | 5.09 | 0.17 | ##### | 0.1400 |  |  |  |  |  |  |  |  |
| 61 | 8 | Ecological restoration | Grassland | ＞20 | 39.03 | 104.58 | 1.310 | 0.050 | 1.290 | #### |  |  |  |  |  |  |  |  |  |  |  |  |  |  |  |  |  |  |  |  | 5.09 | 0.17 | 4.4100 | ##### |  |  |  |  |  |  |  |  |
| 62 | 8 | Ecological restoration | Grassland | ＞20 | 39.03 | 104.58 | 1.310 | 0.050 | 1.380 | #### |  |  |  |  |  |  |  |  |  |  |  |  |  |  |  |  |  |  |  |  | 7.290 | 0.006 | 7.290 | 0.021 |  |  |  |  |  |  |  |  |
| 63 | 9 | Ecological restoration | Forest | ＞20 | 35.61 | 106.24 | 1.310 | 0.050 | 2.060 | #### |  |  |  |  |  |  |  |  |  |  |  |  |  |  |  |  |  |  |  |  | 7.290 | 0.006 | 7.210 | 0.006 |  |  |  |  |  |  |  |  |
| 64 | 9 | Ecological restoration | Forest | ＞20 | 35.61 | 106.24 | 1.310 | 0.050 | 2.980 | 0.610 |  |  |  |  |  |  |  |  |  |  |  |  |  |  |  |  |  |  |  |  | 7.290 | 0.006 | 7.240 | 0.021 |  |  |  |  |  |  |  |  |
| 65 | 9 | Ecological restoration | Forest | ＞20 | 35.61 | 106.24 | 1.310 | 0.050 | 3.010 | #### |  |  |  |  |  |  |  |  |  |  |  |  |  |  |  |  |  |  |  |  | 7.290 | 0.006 | 7.300 | 0.015 |  |  |  |  |  |  |  |  |
| 66 | 9 | Ecological restoration | Forest | ＞20 | 35.61 | 106.24 | 1.310 | 0.050 | 2.180 | #### |  |  |  |  |  |  |  |  |  |  |  |  |  |  |  |  |  |  |  |  | 5.680 | 0.040 | 5.640 | 0.150 |  |  |  |  |  |  |  |  |
| 67 | 9 | Ecological restoration | Forest | ＞20 | 35.61 | 106.24 | 0.400 | 0.240 | 0.510 | #### | 10.410 | 16.020 | 11.340 | #### |  |  |  |  |  |  |  |  |  |  |  |  |  |  |  |  | 5.800 | 0.200 | 5.600 | 0.060 |  |  |  |  |  |  |  |  |
| 68 | 9 | Ecological restoration | Forest | ＞20 | 35.61 | 106.24 | 0.400 | 0.240 | 0.060 | #### | 10.410 | 16.020 | 9.520 | 6.660 |  |  |  |  |  |  |  |  |  |  |  |  |  |  |  |  | 5.730 | 0.030 | 5.740 | 0.060 |  |  |  |  |  |  |  |  |
| 69 | 10 | Ecological restoration | Grassland | 0-10 | 35.98 | 106.43 |  |  |  |  | 1.397 | 0.210 | 0.962 | 0.096 | 15.50 | 1.550 | 21.08 | 2.108 |  |  |  |  |  |  |  |  |  |  |  |  |  |  |  |  |  |  |  |  |  |  |  |  |
| 70 | 10 | Ecological restoration | Grassland | 0-10 | 35.98 | 106.43 |  |  |  |  | 1.397 | 0.210 | 1.036 | 0.104 | 15.50 | 1.550 | 25.32 | 2.532 |  |  |  |  |  |  |  |  |  |  |  |  |  |  |  |  |  |  |  |  |  |  |  |  |
| 71 | 10 | Ecological restoration | Grassland | 0-10 | 35.98 | 106.43 |  |  |  |  | 1.397 | 0.210 | 1.100 | 0.110 | 15.50 | 1.550 | 14.86 | 1.486 |  |  |  |  |  |  |  |  |  |  |  |  |  |  |  |  |  |  |  |  |  |  |  |  |
| 72 | 10 | Ecological restoration | Grassland | ＞20 | 35.98 | 106.43 |  |  |  |  | 1.397 | 0.210 | 1.480 | 0.148 | 15.50 | 1.550 | 6.58 | 0.658 |  |  |  |  |  |  |  |  |  |  |  |  |  |  |  |  |  |  |  |  |  |  |  |  |
| 73 | 10 | Ecological restoration | Grassland | ＞20 | 35.98 | 106.43 |  |  |  |  | 1.397 | 0.210 | 0.760 | 0.076 | 15.50 | 1.550 | 27.41 | 2.741 |  |  |  |  |  |  |  |  |  |  |  |  |  |  |  |  |  |  |  |  |  |  |  |  |
| 74 | 10 | Ecological restoration | Grassland | ＞20 | 35.98 | 106.43 |  |  |  |  | 1.397 | 0.210 | 0.506 | 0.051 | 15.50 | 1.550 | 7.52 | 0.752 |  |  |  |  |  |  |  |  |  |  |  |  |  |  |  |  |  |  |  |  |  |  |  |  |
| 84 | 11 | Ecological restoration | Forest | 0-10 | 33.30 | 108.35 |  |  |  |  | 1.397 | 0.210 | 0.898 | 0.090 | 15.50 | 1.550 | 14.37 | 1.437 |  |  |  |  |  |  |  |  |  |  |  |  |  |  |  |  |  |  |  |  |  |  |  |  |
| 85 | 11 | Ecological restoration | Forest | 10-20 | 33.30 | 108.35 |  |  |  |  | 1.397 | 0.210 | 3.879 | 0.388 | 15.50 | 1.550 | 19.24 | 1.924 |  |  |  |  |  |  |  |  |  |  |  |  |  |  |  |  |  |  |  |  |  |  |  |  |
| 86 | 11 | Ecological restoration | Forest | ＞20 | 33.30 | 108.35 | 0.332 | 0.033 | 0.568 | #### | 7.524 | 0.752 | 14.052 | 1.405 |  |  |  |  |  |  |  |  |  |  |  |  |  |  |  |  |  |  |  |  |  |  |  |  |  |  |  |  |
| 87 | 11 | Ecological restoration | Forest | ＞20 | 33.30 | 108.35 | 0.332 | 0.033 | 0.587 | #### | 7.524 | 0.752 | 14.485 | 1.449 |  |  |  |  |  |  |  |  |  |  |  |  |  |  |  |  |  |  |  |  |  |  |  |  |  |  |  |  |
| 91 | 13 | Ecological restoration | Forest | ＞20 | 25.88 | 110.48 | 0.332 | 0.033 | 0.591 | #### | 7.524 | 0.752 | 14.537 | 1.454 |  |  |  |  |  |  |  |  |  |  |  |  |  |  |  |  |  |  |  |  |  |  |  |  |  |  |  |  |
| 92 | 13 | Ecological restoration | Forest | ＞20 | 25.88 | 110.48 | 0.299 | 0.030 | 0.559 | #### | 7.648 | 0.765 | 12.978 | 1.298 |  |  |  |  |  |  |  |  |  |  |  |  |  |  |  |  |  |  |  |  |  |  |  |  |  |  |  |  |
| 93 | 14 | Ecological restoration | Grassland | 0-10 | 34.46 | 100.21 | 0.299 | 0.030 | 0.560 | #### | 7.648 | 0.765 | 13.357 | 1.336 |  |  |  |  |  |  |  |  |  |  |  |  |  |  |  |  |  |  |  |  |  |  |  |  |  |  |  |  |
| 94 | 14 | Ecological restoration | Grassland | 0-10 | 34.46 | 100.21 | 0.299 | 0.030 | 0.611 | 0.061 | 7.648 | 0.765 | 13.018 | 1.302 |  |  |  |  |  |  |  |  |  |  |  |  |  |  |  |  |  |  |  |  |  |  |  |  |  |  |  |  |
| 112 | 17 | Ecological restoration | Grassland | 0-10 | 46.50 | 132.95 |  |  |  |  | ##### | 2.570 | 14.190 | 1.419 | 18.23 | 1.823 | 24.37 | 2.437 |  |  |  |  |  |  |  |  |  |  |  |  |  |  |  |  |  |  |  |  |  |  |  |  |
| 113 | 17 | Ecological restoration | Grassland | 0-10 | 46.50 | 132.95 |  |  |  |  | 20.190 | 4.100 | 10.900 | 1.090 | 10.04 | 1.004 | 21.14 | 2.114 |  |  |  |  |  |  |  |  |  |  |  |  |  |  |  |  |  |  |  |  |  |  |  |  |
| 114 | 17 | Ecological restoration | Grassland | 10-20 | 46.50 | 132.95 |  |  |  |  | 8.680 | 4.350 | 6.240 | 0.624 | 9.87 | 0.987 | 18.31 | 1.831 |  |  |  |  |  |  |  |  |  |  |  |  |  |  |  |  |  |  |  |  |  |  |  |  |
| 115 | 17 | Ecological restoration | Grassland | 10-20 | 46.50 | 132.95 |  |  |  |  | ##### | 2.570 | 8.550 | 0.855 | 18.23 | 1.823 | 39.37 | 3.937 |  |  |  |  |  |  |  |  |  |  |  |  |  |  |  |  |  |  |  |  |  |  |  |  |
| 142 | 21 | Ecological restoration | Forest | 0-10 | 42.72 | 122.36 |  |  |  |  | 20.190 | 4.100 | 6.370 | 0.637 | 10.04 | 1.004 | 20.13 | 2.013 |  |  |  |  |  |  |  |  |  |  |  |  |  |  |  |  |  |  |  |  |  |  |  |  |
| 143 | 21 | Ecological restoration | Forest | 0-10 | 42.72 | 122.36 |  |  |  |  | 8.680 | 4.350 | 7.230 | 0.723 | 9.87 | 0.987 | 17.34 | 1.734 |  |  |  |  |  |  |  |  |  |  |  |  |  |  |  |  |  |  |  |  |  |  |  |  |
| 144 | 21 | Ecological restoration | Forest | 0-10 | 42.72 | 122.36 |  |  |  |  | ##### | 2.570 | 8.450 | 0.845 | 18.23 | 1.823 | 32.17 | 3.217 |  |  |  |  |  |  |  |  |  |  |  |  |  |  |  |  |  |  |  |  |  |  |  |  |
| 145 | 21 | Ecological restoration | Forest | 0-10 | 42.72 | 122.36 |  |  |  |  | 20.190 | 4.100 | 6.450 | 0.645 | 10.04 | 1.004 | 23.02 | 2.302 |  |  |  |  |  |  |  |  |  |  |  |  |  |  |  |  |  |  |  |  |  |  |  |  |
| 146 | 21 | Ecological restoration | Forest | 0-10 | 42.72 | 122.36 |  |  |  |  | 8.680 | 4.350 | 5.010 | 0.501 | 9.87 | 0.987 | 12.60 | 1.260 |  |  |  |  |  |  |  |  |  |  |  |  |  |  |  |  |  |  |  |  |  |  |  |  |
| 147 | 21 | Ecological restoration | Forest | 0-10 | 42.72 | 122.36 |  |  |  |  | ##### | 2.570 | 8.880 | 0.888 | 18.23 | 1.823 | 23.48 | 2.348 |  |  |  |  |  |  |  |  |  |  |  |  |  |  |  |  |  |  |  |  |  |  |  |  |
| 148 | 22 | Ecological restoration | Forest | ＞20 | 36.07 | 109.13 |  |  |  |  | 20.190 | 4.100 | 6.710 | 0.671 | 10.04 | 1.004 | 20.31 | 2.031 |  |  |  |  |  |  |  |  |  |  |  |  |  |  |  |  |  |  |  |  |  |  |  |  |
| 149 | 22 | Ecological restoration | Forest | ＞20 | 36.07 | 109.13 |  |  |  |  | 8.680 | 4.350 | 3.980 | 0.398 | 9.87 | 0.987 | 8.97 | 0.897 |  |  |  |  |  |  |  |  |  |  |  |  |  |  |  |  |  |  |  |  |  |  |  |  |
| 150 | 22 | Ecological restoration | Forest | ＞20 | 36.07 | 109.13 |  |  |  |  | ##### | 2.570 | 20.190 | 2.019 | 18.23 | 1.823 | 23.04 | 2.304 |  |  |  |  |  |  |  |  |  |  |  |  |  |  |  |  |  |  |  |  |  |  |  |  |
| 151 | 23 | Ecological restoration | Grassland | 0-10 | 33.67 | 105.85 |  |  |  |  | 20.190 | 4.100 | 9.370 | 0.937 | 10.04 | 1.004 | 20.37 | 2.037 |  |  |  |  |  |  |  |  |  |  |  |  |  |  |  |  |  |  |  |  |  |  |  |  |
| 152 | 23 | Ecological restoration | Grassland | 0-10 | 33.67 | 105.85 |  |  |  |  | 8.680 | 4.350 | 7.150 | 0.715 | 9.87 | 0.987 | 17.08 | 1.708 |  |  |  |  |  |  |  |  |  |  |  |  |  |  |  |  |  |  |  |  |  |  |  |  |
| 153 | 23 | Ecological restoration | Grassland | 0-10 | 33.67 | 105.85 | 1.84 | 0.184 | 1.721 | 0.17 | 10.800 | 1.080 | 6.394 | 0.639 |  |  |  |  |  |  |  |  |  |  |  |  |  |  |  |  | 8.21 | 0.82 | 5.90 | 0.590 | 1.562 | 0.156 | 1.324 | 0.132 | 0.713 | 0.071 | 0.601 | 0.060 |
| 154 | 23 | Ecological restoration | Grassland | 0-10 | 33.67 | 105.85 | 1.84 | 0.184 | 2.546 | 0.25 | 10.800 | 1.080 | 14.025 | 1.403 |  |  |  |  |  |  |  |  |  |  |  |  |  |  |  |  | 8.21 | 0.82 | 3.42 | 0.342 | 1.562 | 0.156 | 0.650 | 0.065 | 0.713 | 0.071 | 0.328 | 0.033 |
| 155 | 23 | Ecological restoration | Grassland | 0-10 | 33.67 | 105.85 | 1.84 | 0.184 | 2.417 | 0.24 | 10.800 | 1.080 | 11.453 | 1.145 |  |  |  |  |  |  |  |  |  |  |  |  |  |  |  |  | 8.21 | 0.82 | 7.05 | 0.705 | 1.562 | 0.156 | 1.542 | 0.154 | 0.713 | 0.071 | 0.617 | 0.062 |
| 156 | 23 | Ecological restoration | Grassland | 0-10 | 33.67 | 105.85 | 1.84 | 0.184 | 1.832 | 0.18 | 10.800 | 1.080 | 6.211 | 0.621 |  |  |  |  |  |  |  |  |  |  |  |  |  |  |  |  | 8.21 | 0.82 | 6.18 | 0.618 | 1.562 | 0.156 | 1.349 | 0.135 | 0.713 | 0.071 | 0.596 | 0.060 |
| 157 | 24 | Ecological restoration | Grassland | ＞20 | 43.59 | 116.71 | 0.750 | 0.075 | 0.530 | #### |  |  |  |  | 31.20 | 3.120 | 40.03 | 4.003 |  |  |  |  |  |  |  |  |  |  |  |  |  |  |  |  |  |  |  |  |  |  |  |  |
| 158 | 25 | Ecological restoration | Grassland | 0-10 | 36.17 | 106.35 | 0.750 | 0.075 | 0.560 | #### |  |  |  |  | 31.20 | 3.120 | 8.34 | 0.834 |  |  |  |  |  |  |  |  |  |  |  |  |  |  |  |  |  |  |  |  |  |  |  |  |
| 159 | 25 | Ecological restoration | Grassland | 0-10 | 36.17 | 106.35 | 0.750 | 0.075 | 0.580 | #### |  |  |  |  | 31.20 | 3.120 | 38.16 | 3.816 |  |  |  |  |  |  |  |  |  |  |  |  |  |  |  |  |  |  |  |  |  |  |  |  |
| 160 | 25 | Ecological restoration | Grassland | 0-10 | 36.17 | 106.35 |  |  |  |  | 3.100 | 0.310 | 1.530 | 0.153 |  |  |  |  |  |  |  |  |  |  |  |  |  |  |  |  | 17.30 | 5.02 | 5.700 | 0.570 | 1.980 | 0.415 | 1.530 | 0.153 |  |  |  |  |
| 161 | 25 | Ecological restoration | Grassland | 0-10 | 36.17 | 106.35 |  |  |  |  | 3.100 | 0.310 | 2.330 | 0.233 |  |  |  |  |  |  |  |  |  |  |  |  |  |  |  |  | 17.30 | 5.02 | 18.100 | 1.810 | 1.980 | 0.415 | 0.820 | 0.082 |  |  |  |  |
| 162 | 25 | Ecological restoration | Grassland | 10-20 | 36.17 | 106.35 | 0.790 | 0.060 | 0.780 | #### | 15.920 | 0.060 | 16.340 | 0.040 |  |  |  |  |  |  |  |  |  |  |  |  |  |  |  |  |  |  |  |  |  |  |  |  |  |  |  |  |
| 163 | 25 | Ecological restoration | Grassland | 10-20 | 36.17 | 106.35 | 0.610 | 0.760 | 0.600 | #### | 3.700 | 0.500 | 3.590 | 0.960 |  |  |  |  |  |  |  |  |  |  |  |  |  |  |  |  |  |  |  |  |  |  |  |  |  |  |  |  |
| 164 | 25 | Ecological restoration | Grassland | 10-20 | 36.17 | 106.35 | 0.360 | 0.156 | 0.340 | #### | 1.890 | 0.813 | 2.300 | 0.230 |  |  |  |  | 67.52 | 19.70 | 271.26 | 36.99 |  |  |  |  |  |  |  |  | 9.67 | 1.00 | 14.67 | 1.467 | 0.372 | 3.340 | 0.551 | 0.055 |  |  |  |  |
| 165 | 25 | Ecological restoration | Grassland | 10-20 | 36.17 | 106.35 | 0.360 | 0.156 | 0.300 | #### | 1.890 | 0.813 | 2.900 | 0.290 |  |  |  |  | 67.52 | 19.70 | ##### | 16.88 |  |  |  |  |  |  |  |  | 9.67 | 1.00 | 16.67 | 1.667 | 0.372 | 3.340 | 0.280 | 0.028 |  |  |  |  |
| 166 | 25 | Ecological restoration | Grassland | ＞20 | 36.17 | 106.35 | 0.360 | 0.156 | 0.290 | #### | 1.890 | 0.813 | 2.710 | 0.271 |  |  |  |  | 67.52 | 19.70 | 313.84 | 24.64 |  |  |  |  |  |  |  |  | 9.67 | 1.00 | 13.67 | 1.367 | 0.372 | 3.340 | 0.640 | 0.064 |  |  |  |  |
| 167 | 25 | Ecological restoration | Grassland | ＞20 | 36.17 | 106.35 | 0.360 | 0.156 | 0.320 | 0.121 | 1.890 | 0.813 | 4.800 | 0.480 |  |  |  |  | 67.52 | 19.70 | 330.61 | 51.35 |  |  |  |  |  |  |  |  | 9.67 | 1.00 | 16.67 | 1.667 | 0.372 | 3.340 | 0.380 | 0.038 |  |  |  |  |
| 168 | 25 | Ecological restoration | Grassland | ＞20 | 36.17 | 106.35 | 0.360 | 0.156 | 0.300 | #### | 1.890 | 0.813 | 1.700 | 0.170 |  |  |  |  | 67.52 | 19.70 | 379.91 | 22.20 |  |  |  |  |  |  |  |  | 9.67 | 1.00 | 19.33 | 1.933 | 0.372 | 3.340 | 0.660 | 0.066 |  |  |  |  |
| 169 | 25 | Ecological restoration | Grassland | ＞20 | 36.17 | 106.35 | 0.490 | 0.049 | 0.610 | 0.061 | 2.370 | 0.237 | 2.640 | 0.264 |  |  |  |  |  |  |  |  |  |  |  |  |  |  |  |  | 2.28 | 0.23 | 0.94 | 0.094 | 0.530 | 0.053 | 0.940 | 0.094 | 1.01 | 0.10 | 0.890 | 0.089 |
| 170 | 25 | Ecological restoration | Grassland | ＞20 | 36.17 | 106.35 | 0.380 | 0.038 | 0.500 | #### | 1.300 | 0.130 | 1.420 | 0.142 |  |  |  |  |  |  |  |  |  |  |  |  |  |  |  |  | 2.28 | 0.23 | 3.28 | 0.328 | 0.530 | 0.053 | 1.270 | 0.127 | 1.01 | 0.10 | 0.740 | 0.074 |
| 171 | 25 | Ecological restoration | Grassland | ＞20 | 36.17 | 106.35 | 0.490 | 0.049 | 0.640 | #### | 2.370 | 0.237 | 1.960 | 0.196 |  |  |  |  |  |  |  |  |  |  |  |  |  |  |  |  | 2.28 | 0.23 | 1.47 | 0.147 | 0.530 | 0.053 | 1.830 | 0.183 | 1.01 | 0.10 | 1.290 | 0.129 |
| 172 | 25 | Ecological restoration | Grassland | ＞20 | 36.17 | 106.35 | 0.380 | 0.038 | 0.420 | #### | 1.300 | 0.130 | 0.870 | 0.087 |  |  |  |  |  |  |  |  |  |  |  |  |  |  |  |  | 2.28 | 0.23 | 2.51 | 0.251 | 0.530 | 0.053 | 1.200 | 0.120 | 1.01 | 0.10 | 0.910 | 0.091 |
| 173 | 25 | Ecological restoration | Grassland | ＞20 | 36.17 | 106.35 | 0.490 | 0.049 | 0.530 | #### | 2.370 | 0.237 | 2.980 | 0.298 |  |  |  |  |  |  |  |  |  |  |  |  |  |  |  |  | 2.28 | 0.23 | 3.79 | 0.379 | 0.530 | 0.053 | 1.830 | 0.183 | 1.01 | 0.10 | 0.860 | 0.086 |
| 174 | 26 | Ecological restoration | Grassland | 0-10 | 36.77 | 109.23 | 0.380 | 0.038 | 0.430 | #### | 1.300 | 0.130 | 1.500 | 0.150 |  |  |  |  |  |  |  |  |  |  |  |  |  |  |  |  | 2.28 | 0.23 | 3.42 | 0.342 | 0.530 | 0.053 | 1.360 | 0.136 | 1.01 | 0.10 | 0.740 | 0.074 |
| 175 | 26 | Ecological restoration | Grassland | 0-10 | 36.77 | 109.23 | 0.490 | 0.049 | 0.430 | #### | 2.370 | 0.237 | 1.140 | 0.114 |  |  |  |  |  |  |  |  |  |  |  |  |  |  |  |  | 2.28 | 0.23 | 3.60 | 0.360 | 0.530 | 0.053 | 1.020 | 0.102 | 1.01 | 0.10 | 0.640 | 0.064 |
| 176 | 26 | Ecological restoration | Grassland | 10-20 | 36.77 | 109.23 | 0.380 | 0.038 | 0.360 | #### | 1.300 | 0.130 | 0.900 | 0.090 |  |  |  |  |  |  |  |  |  |  |  |  |  |  |  |  | 2.28 | 0.23 | 4.72 | 0.472 | 0.530 | 0.053 | 0.990 | 0.099 | 1.01 | 0.10 | 0.870 | 0.087 |
| 177 | 26 | Ecological restoration | Grassland | 10-20 | 36.77 | 109.23 | 0.490 | 0.049 | 0.590 | #### | 2.370 | 0.237 | 1.310 | 0.131 |  |  |  |  |  |  |  |  |  |  |  |  |  |  |  |  | 2.28 | 0.23 | 3.43 | 0.343 | 0.530 | 0.053 | 0.870 | 0.087 | 1.01 | 0.10 | 0.670 | 0.067 |
| 178 | 26 | Ecological restoration | Grassland | ＞20 | 36.77 | 109.23 | 0.380 | 0.038 | 0.440 | #### | 1.300 | 0.130 | 0.670 | 0.067 |  |  |  |  |  |  |  |  |  |  |  |  |  |  |  |  | 2.28 | 0.23 | 3.11 | 0.311 | 0.530 | 0.053 | 0.690 | 0.069 | 1.01 | 0.10 | 0.440 | 0.044 |
| 179 | 26 | Ecological restoration | Grassland | ＞20 | 36.77 | 109.23 | 0.490 | 0.049 | 0.480 | #### | 2.370 | 0.237 | 2.700 | 0.270 |  |  |  |  |  |  |  |  |  |  |  |  |  |  |  |  | 2.28 | 0.23 | 3.57 | 0.357 | 0.530 | 0.053 | 0.250 | 0.025 | 1.01 | 0.10 | 0.780 | 0.078 |
| 180 | 27 | Ecological restoration | Forest | 10-20 | 36.86 | 109.33 | 0.380 | 0.038 | 0.370 | #### | 1.300 | 0.130 | 1.730 | 0.173 |  |  |  |  |  |  |  |  |  |  |  |  |  |  |  |  | 2.28 | 0.23 | 1.22 | 0.122 | 0.530 | 0.053 | 0.520 | 0.052 | 1.01 | 0.10 | 0.380 | 0.038 |
| 181 | 27 | Ecological restoration | Forest | ＞20 | 36.86 | 109.33 |  |  |  |  | 12.500 | 1.250 | 18.030 | 1.803 |  |  |  |  |  |  |  |  |  |  |  |  |  |  |  |  | 0.75 | 0.08 | 2.28 | 0.228 | 1.230 | 0.123 | 1.960 | 0.196 | 0.54 | 0.05 | 0.820 | 0.082 |
| 182 | 27 | Ecological restoration | Forest | ＞20 | 36.86 | 109.33 |  |  |  |  | 12.500 | 1.250 | 11.680 | 1.168 |  |  |  |  |  |  |  |  |  |  |  |  |  |  |  |  | 0.75 | 0.08 | 1.55 | 0.155 | 1.230 | 0.123 | 1.680 | 0.168 | 0.54 | 0.054 | 0.630 | 0.063 |
| 186 | 28 | Ecological restoration | Grassland | 0-10 | 46.11 | 87.98 |  |  |  |  | 12.500 | 1.250 | 12.440 | 1.244 |  |  |  |  |  |  |  |  |  |  |  |  |  |  |  |  | 0.75 | 0.08 | 1.04 | 0.104 | 1.230 | 0.123 | 1.430 | 0.143 | 0.54 | 0.054 | 0.650 | 0.065 |
| 187 | 28 | Ecological restoration | Grassland | 0-10 | 46.11 | 87.98 |  |  |  |  | 12.500 | 1.250 | 12.100 | 1.210 |  |  |  |  |  |  |  |  |  |  |  |  |  |  |  |  | 0.75 | 0.08 | 0.74 | 0.074 | 1.230 | 0.123 | 1.270 | 0.127 | 0.54 | 0.054 | 0.640 | 0.064 |
| 188 | 28 | Ecological restoration | Grassland | 0-10 | 46.11 | 87.98 | 0.310 | 0.554 | 0.290 | #### | 3.310 | 0.294 | 2.120 | 0.212 |  |  |  |  |  |  |  |  |  |  |  |  |  |  |  |  | 9.75 | 0.98 | 8.50 | 0.850 | 0.693 | 0.069 | 0.550 | 0.055 | 0.482 | 0.048 | 0.328 | 0.033 |
| 192 | 29 | Ecological restoration | Grassland | 0-10 | 43.03 | 119.65 | 0.310 | 0.554 | 0.210 | 0.021 | 3.310 | 0.294 | 1.230 | 0.123 |  |  |  |  |  |  |  |  |  |  |  |  |  |  |  |  | 9.75 | 0.98 | 10.00 | 1.000 | 0.693 | 0.069 | 0.642 | 0.064 | 0.482 | 0.048 | 0.441 | 0.044 |
| 193 | 29 | Ecological restoration | Grassland | 10-20 | 43.03 | 119.65 | 0.310 | 0.554 | 0.210 | 0.021 | 3.310 | 0.294 | 1.000 | 0.100 |  |  |  |  |  |  |  |  |  |  |  |  |  |  |  |  | 9.75 | 0.98 | 11.50 | 1.150 | 0.693 | 0.069 | 0.636 | 0.064 | 0.482 | 0.048 | 0.384 | 0.038 |
| 194 | 29 | Ecological restoration | Grassland | ＞20 | 43.03 | 119.65 | 0.310 | 0.554 | 0.260 | #### | 3.310 | 0.294 | 1.520 | 0.152 |  |  |  |  |  |  |  |  |  |  |  |  |  |  |  |  | 9.75 | 0.98 | 12.00 | 1.200 | 0.693 | 0.069 | 0.637 | 0.064 | 0.482 | 0.048 | 0.398 | 0.040 |
| 195 | 30 | Ecological restoration | Grassland | 0-10 | 37.07 | 106.50 | 0.310 | 0.554 | 0.340 | #### | 3.310 | 0.294 | 1.220 | 0.122 |  |  |  |  |  |  |  |  |  |  |  |  |  |  |  |  | 9.75 | 0.98 | 11.00 | 1.100 | 0.693 | 0.069 | 0.658 | 0.066 | 0.482 | 0.048 | 0.423 | 0.042 |
| 196 | 30 | Ecological restoration | Grassland | 10-20 | 37.07 | 106.50 | 0.310 | 0.554 | 0.240 | #### | 3.310 | 0.294 | 2.600 | 0.260 |  |  |  |  |  |  |  |  |  |  |  |  |  |  |  |  | 9.75 | 0.98 | 16.50 | 1.650 | 0.693 | 0.069 | 0.707 | 0.071 | 0.482 | 0.048 | 0.392 | 0.039 |
| 197 | 30 | Ecological restoration | Grassland | ＞20 | 37.07 | 106.50 | 0.310 | 0.554 | 0.280 | #### | 3.310 | 0.294 | 0.840 | 0.084 |  |  |  |  |  |  |  |  |  |  |  |  |  |  |  |  | 9.75 | 0.98 | 11.00 | 1.100 | 0.693 | 0.069 | 1.031 | 0.103 | 0.482 | 0.048 | 0.543 | 0.054 |
| 198 | 30 | Ecological restoration | Grassland | 0-10 | 37.07 | 106.50 | 0.310 | 0.554 | 0.160 | 0.016 | 3.310 | 0.294 | 2.390 | 0.239 |  |  |  |  |  |  |  |  |  |  |  |  |  |  |  |  | 9.75 | 0.98 | 5.50 | 0.550 | 0.693 | 0.069 | 0.686 | 0.069 | 0.482 | 0.048 | 0.408 | 0.041 |
| 199 | 30 | Ecological restoration | Grassland | 10-20 | 37.07 | 106.50 | 1.870 | 0.187 | 1.640 | 0.164 | 40.140 | 4.014 | 44.180 | 4.418 |  |  |  |  |  |  |  |  |  |  |  |  |  |  |  |  | 0.64 | 0.22 | 1.12 | 0.087 | 0.350 | 0.170 | 1.160 | 0.116 |  |  |  |  |
| 200 | 30 | Ecological restoration | Grassland | ＞20 | 37.07 | 106.50 | 1.940 | 0.194 | 1.540 | 0.154 | 44.160 | 4.416 | 47.790 | 4.779 |  |  |  |  |  |  |  |  |  |  |  |  |  |  |  |  | 0.64 | 0.22 | 1.12 | 0.087 | 0.350 | 0.170 | 1.160 | 0.116 |  |  |  |  |
| 201 | 30 | Ecological restoration | Grassland | 0-10 | 37.07 | 106.50 | 1.870 | 0.187 | 2.280 | #### | 40.140 | 4.014 | 78.710 | 7.871 |  |  |  |  |  |  |  |  |  |  |  |  |  |  |  |  | 0.64 | 0.22 | 1.05 | 0.138 | 0.350 | 0.170 | 1.030 | 0.103 |  |  |  |  |
| 202 | 30 | Ecological restoration | Grassland | 10-20 | 37.07 | 106.50 | 1.940 | 0.194 | 2.280 | #### | 44.160 | 4.416 | 61.730 | 6.173 |  |  |  |  |  |  |  |  |  |  |  |  |  |  |  |  | 0.64 | 0.22 | 1.05 | 0.138 | 0.350 | 0.170 | 1.030 | 0.103 |  |  |  |  |
| 203 | 30 | Ecological restoration | Grassland | ＞20 | 37.07 | 106.50 | 1.870 | 0.187 | 2.270 | #### | 40.140 | 4.014 | 52.390 | 5.239 |  |  |  |  |  |  |  |  |  |  |  |  |  |  |  |  | 0.64 | 0.22 | 1.07 | 0.727 | 0.350 | 0.170 | 0.880 | 0.088 |  |  |  |  |
| 204 | 31 | Ecological restoration | Grassland | ＞20 | 33.13 | 102.62 | 1.940 | 0.194 | 2.210 | 0.221 | 44.160 | 4.416 | 52.120 | 5.212 |  |  |  |  |  |  |  |  |  |  |  |  |  |  |  |  | 0.64 | 0.22 | 1.07 | 0.727 | 0.350 | 0.170 | 0.880 | 0.088 |  |  |  |  |
| 205 | 31 | Ecological restoration | Grassland | ＞20 | 33.13 | 102.62 | 1.870 | 0.187 | 2.490 | #### | 40.140 | 4.014 | 87.780 | 8.778 |  |  |  |  |  |  |  |  |  |  |  |  |  |  |  |  | 0.64 | 0.22 | 1.35 | 0.882 | 0.350 | 0.170 | 1.170 | 0.117 |  |  |  |  |
| 206 | 31 | Ecological restoration | Grassland | ＞20 | 33.13 | 102.62 | 1.940 | 0.194 | 2.410 | 0.241 | 44.160 | 4.416 | 65.100 | 6.510 |  |  |  |  |  |  |  |  |  |  |  |  |  |  |  |  | 0.64 | 0.22 | 1.35 | 0.882 | 0.350 | 0.170 | 1.170 | 0.117 |  |  |  |  |
| 213 | 32 | Ecological restoration | Grassland | 0-10 | 44.25 | 123.57 | 1.870 | 0.187 | 2.910 | 0.291 | 40.140 | 4.014 | 88.230 | 8.823 |  |  |  |  |  |  |  |  |  |  |  |  |  |  |  |  | 0.64 | 0.22 | 2.62 | 0.692 | 0.350 | 0.170 | 1.860 | 0.186 |  |  |  |  |
| 214 | 32 | Ecological restoration | Grassland | 0-10 | 44.25 | 123.57 | 1.940 | 0.194 | 2.720 | #### | 44.160 | 4.416 | 49.230 | 4.923 |  |  |  |  |  |  |  |  |  |  |  |  |  |  |  |  | 0.64 | 0.22 | 2.62 | 0.692 | 0.350 | 0.170 | 1.860 | 0.186 |  |  |  |  |
| 215 | 32 | Ecological restoration | Grassland | 0-10 | 44.25 | 123.57 | 0.262 | 0.026 | 0.209 | 0.021 | 1.539 | 0.154 | 0.931 | 0.093 |  |  |  |  |  |  |  |  |  |  |  |  |  |  |  |  | 2.35 | 0.23 | 2.59 | 0.259 | 1.882 | 0.188 | 1.777 | 0.178 | 0.831 | 0.083 | 0.816 | 0.082 |
| 219 | 32 | Ecological restoration | Grassland | 0-10 | 44.25 | 123.57 | 0.186 | 0.019 | 0.223 | #### | 0.470 | 0.047 | 0.675 | 0.067 |  |  |  |  |  |  |  |  |  |  |  |  |  |  |  |  | 2.35 | 0.23 | 2.59 | 0.259 | 1.882 | 0.188 | 1.777 | 0.178 | 0.831 | 0.083 | 0.816 | 0.082 |
| 220 | 32 | Ecological restoration | Grassland | 0-10 | 44.25 | 123.57 | 0.262 | 0.026 | 0.249 | #### | 1.539 | 0.154 | 0.666 | 0.067 |  |  |  |  |  |  |  |  |  |  |  |  |  |  |  |  | 2.35 | 0.23 | 3.23 | 0.323 | 1.882 | 0.188 | 1.803 | 0.180 | 0.831 | 0.083 | 0.817 | 0.082 |
| 221 | 32 | Ecological restoration | Grassland | 0-10 | 44.25 | 123.57 | 0.186 | 0.019 | 0.193 | 0.019 | 0.470 | 0.047 | 0.877 | 0.088 |  |  |  |  |  |  |  |  |  |  |  |  |  |  |  |  | 2.35 | 0.23 | 3.23 | 0.323 | 1.882 | 0.188 | 1.803 | 0.180 | 0.831 | 0.083 | 0.817 | 0.082 |
| 225 | 33 | Ecological restoration | Grassland | 10-20 | 36.14 | 106.84 | 0.262 | 0.026 | 0.238 | #### | 1.539 | 0.154 | 1.080 | 0.108 |  |  |  |  |  |  |  |  |  |  |  |  |  |  |  |  | 2.35 | 0.23 | 3.06 | 0.306 | 1.882 | 0.188 | 1.999 | 0.200 | 0.831 | 0.083 | 0.856 | 0.086 |
| 226 | 33 | Ecological restoration | Grassland | 10-20 | 36.14 | 106.84 | 0.186 | 0.019 | 0.290 | #### | 0.470 | 0.047 | 0.801 | 0.080 |  |  |  |  |  |  |  |  |  |  |  |  |  |  |  |  | 2.35 | 0.23 | 3.06 | 0.306 | 1.882 | 0.188 | 1.999 | 0.200 | 0.831 | 0.083 | 0.856 | 0.086 |
| 227 | 33 | Ecological restoration | Grassland | 10-20 | 36.14 | 106.84 | 0.262 | 0.026 | 0.316 | #### | 1.539 | 0.154 | 1.774 | 0.177 |  |  |  |  |  |  |  |  |  |  |  |  |  |  |  |  | 2.35 | 0.23 | 3.02 | 0.302 | 1.882 | 0.188 | 1.899 | 0.190 | 0.831 | 0.083 | 0.838 | 0.084 |
| 228 | 33 | Ecological restoration | Grassland | 10-20 | 36.14 | 106.84 | 0.186 | 0.019 | 0.202 | #### | 0.470 | 0.047 | 0.646 | 0.065 |  |  |  |  |  |  |  |  |  |  |  |  |  |  |  |  | 2.35 | 0.23 | 3.02 | 0.302 | 1.882 | 0.188 | 1.899 | 0.190 | 0.831 | 0.083 | 0.838 | 0.084 |
| 229 | 33 | Ecological restoration | Grassland | 10-20 | 36.14 | 106.84 | 0.130 | 0.013 | 0.100 | 0.010 | 2.322 | 0.232 | 1.830 | 0.183 |  |  |  |  | 1.13 | 0.11 | 2.45 | 0.25 | 0.23 | 0.023 | 0.53 | 0.05 |  |  |  |  | 2.05 | 0.21 | 2.27 | 0.227 |  |  |  |  | 0.68 | 0.07 | 0.792 | 0.079 |
| 230 | 33 | Ecological restoration | Grassland | 10-20 | 36.14 | 106.84 | 0.110 | 0.011 | 0.120 | 0.012 | 2.147 | 0.215 | 2.210 | 0.221 |  |  |  |  | 0.00 | 0.00 | 0.82 | 0.08 | 0.00 | 0.000 | 0.31 | 0.03 |  |  |  |  | 2.05 | 0.21 | 2.27 | 0.227 |  |  |  |  | 0.68 | 0.07 | 0.792 | 0.079 |
| 231 | 33 | Ecological restoration | Grassland | 10-20 | 36.14 | 106.84 | 0.130 | 0.013 | 0.130 | 0.013 | 2.322 | 0.232 | 2.063 | 0.206 |  |  |  |  | 1.13 | 0.11 | 3.15 | 0.32 | 0.23 | 0.023 | 0.72 | 0.07 |  |  |  |  | 2.05 | 0.21 | 2.92 | 0.292 |  |  |  |  | 0.68 | 0.07 | 0.845 | 0.085 |
| 232 | 34 | Ecological restoration | Grassland | 0-10 | 32.51 | 91.67 | 0.110 | 0.011 | 0.120 | 0.012 | 2.147 | 0.215 | 2.238 | 0.224 |  |  |  |  | 0.00 | 0.00 | 1.09 | 0.11 | 0.00 | 0.000 | 0.61 | 0.06 |  |  |  |  | 2.05 | 0.21 | 2.92 | 0.292 |  |  |  |  | 0.68 | 0.07 | 0.845 | 0.085 |
| 233 | 34 | Ecological restoration | Grassland | 0-10 | 32.51 | 91.67 | 0.130 | 0.013 | 0.130 | 0.013 | 2.322 | 0.232 | 2.195 | 0.220 |  |  |  |  | 1.13 | 0.11 | 3.82 | 0.38 | 0.23 | 0.023 | 1.68 | 0.17 |  |  |  |  | 2.05 | 0.21 | 3.14 | 0.314 |  |  |  |  | 0.68 | 0.07 | 0.870 | 0.087 |
| 234 | 34 | Ecological restoration | Grassland | ＞20 | 32.51 | 91.67 | 0.110 | 0.011 | 0.190 | 0.019 | 2.147 | 0.215 | 2.316 | 0.232 |  |  |  |  | 0.00 | 0.00 | 1.84 | 0.18 | 0.00 | 0.000 | 1.00 | 0.10 |  |  |  |  | 2.05 | 0.21 | 3.14 | 0.314 |  |  |  |  | 0.68 | 0.07 | 0.870 | 0.087 |
| 235 | 35 | Ecological restoration | Grassland | 0-10 | 32.52 | 91.67 | 0.580 | 0.028 | 0.620 | #### | 16.620 | 4.186 | 4.310 | 1.895 | 38.67 | 3.867 | #### | 5.633 |  |  |  |  |  |  |  |  |  |  |  |  |  |  |  |  |  |  |  |  |  |  |  |  |
| 237 | 35 | Ecological restoration | Grassland | ＞20 | 32.52 | 91.67 | 0.580 | 0.028 | 0.600 | #### | 16.620 | 4.186 | 5.110 | 1.357 | 38.67 | 3.867 | #### | 7.084 |  |  |  |  |  |  |  |  |  |  |  |  |  |  |  |  |  |  |  |  |  |  |  |  |
| 238 | 36 | Ecological restoration | Grassland | 0-10 | 34.90 | 102.1 | 0.580 | 0.028 | 0.610 | #### | 16.620 | 4.186 | 4.680 | 1.782 | 38.67 | 3.867 | #### | 7.561 |  |  |  |  |  |  |  |  |  |  |  |  |  |  |  |  |  |  |  |  |  |  |  |  |
| 239 | 36 | Ecological restoration | Grassland | 0-10 | 34.90 | 102.1 | 0.740 | 0.090 | 0.621 | #### |  |  |  |  |  |  |  |  | 110.23 | 20.54 | 128.44 | 18.35 |  |  |  |  |  |  |  |  | 27.90 | 0.84 | 29.10 | 2.100 | 3.450 | 0.240 | 3.710 | 0.330 |  |  |  |  |
| 240 | 36 | Ecological restoration | Grassland | 0-10 | 34.90 | 102.1 | 0.670 | 0.030 | 0.588 | 0.010 |  |  |  |  |  |  |  |  | 110.23 | 20.54 | 128.44 | 18.35 |  |  |  |  |  |  |  |  | 27.90 | 0.84 | 29.10 | 2.100 | 3.450 | 0.240 | 3.710 | 0.330 |  |  |  |  |
| 241 | 37 | Ecological restoration | Forest | 0-10 | 30.32 | 117.67 | 0.812 | 0.020 | 0.650 | #### |  |  |  |  |  |  |  |  | 110.23 | 20.54 | 128.44 | 18.35 |  |  |  |  |  |  |  |  | 27.90 | 0.84 | 29.10 | 2.100 | 3.450 | 0.240 | 3.710 | 0.330 |  |  |  |  |
| 242 | 37 | Ecological restoration | Forest | 10-20 | 30.32 | 117.67 | 0.740 | 0.090 | 0.510 | #### |  |  |  |  |  |  |  |  | 110.23 | 20.54 | 221.32 | 22.57 |  |  |  |  |  |  |  |  | 27.90 | 0.84 | 28.97 | 0.770 | 3.450 | 0.240 | 3.580 | 0.070 |  |  |  |  |
| 243 | 37 | Ecological restoration | Forest | ＞20 | 30.32 | 117.67 | 0.670 | 0.030 | 0.500 | #### |  |  |  |  |  |  |  |  | 110.23 | 20.54 | 221.32 | 22.57 |  |  |  |  |  |  |  |  | 27.90 | 0.84 | 28.97 | 0.770 | 3.450 | 0.240 | 3.580 | 0.070 |  |  |  |  |
| 244 | 37 | Ecological restoration | Forest | ＞20 | 30.32 | 117.67 | 0.812 | 0.020 | 0.570 | 0.010 |  |  |  |  |  |  |  |  | 110.23 | 20.54 | 221.32 | 22.57 |  |  |  |  |  |  |  |  | 27.90 | 0.84 | 28.97 | 0.770 | 3.450 | 0.240 | 3.580 | 0.070 |  |  |  |  |
| 245 | 37 | Ecological restoration | Forest | 0-10 | 30.32 | 117.67 | 3.210 | 1.200 | 1.840 | 1.530 | 4.750 | 1.040 | 5.780 | 2.880 |  |  |  |  |  |  |  |  |  |  |  |  | 2.54 | 0.21 | 64.77 | 2.58 | 6.01 | 0.45 | 7.68 | 0.310 | 0.990 | 0.070 | 1.420 | 0.010 |  |  |  |  |
| 246 | 37 | Ecological restoration | Forest | 10-20 | 30.32 | 117.67 |  |  |  |  | 5.337 | 0.534 | 6.325 | 0.633 |  |  |  |  |  |  |  |  |  |  |  |  |  |  |  |  |  |  |  |  |  |  |  |  |  |  |  |  |
| 247 | 37 | Ecological restoration | Forest | ＞20 | 30.32 | 117.67 |  |  |  |  | 4.698 | 0.470 | 5.324 | 0.532 |  |  |  |  |  |  |  |  |  |  |  |  |  |  |  |  |  |  |  |  |  |  |  |  |  |  |  |  |
| 248 | 37 | Ecological restoration | Forest | ＞20 | 30.32 | 117.67 |  |  |  |  | 4.787 | 0.479 | 4.625 | 0.463 |  |  |  |  |  |  |  |  |  |  |  |  |  |  |  |  |  |  |  |  |  |  |  |  |  |  |  |  |
| 249 | 37 | Ecological restoration | Forest | 0-10 | 30.32 | 117.67 |  |  |  |  | 2.351 | 0.235 | 3.022 | 0.302 |  |  |  |  |  |  |  |  |  |  |  |  |  |  |  |  |  |  |  |  |  |  |  |  |  |  |  |  |
| 250 | 37 | Ecological restoration | Forest | 10-20 | 30.32 | 117.67 |  |  |  |  | 5.337 | 0.534 | 6.355 | 0.636 |  |  |  |  |  |  |  |  |  |  |  |  |  |  |  |  |  |  |  |  |  |  |  |  |  |  |  |  |
| 251 | 37 | Ecological restoration | Forest | ＞20 | 30.32 | 117.67 |  |  |  |  | 4.698 | 0.470 | 5.021 | 0.502 |  |  |  |  |  |  |  |  |  |  |  |  |  |  |  |  |  |  |  |  |  |  |  |  |  |  |  |  |
| 252 | 37 | Ecological restoration | Forest | ＞20 | 30.32 | 117.67 |  |  |  |  | 4.787 | 0.479 | 4.856 | 0.486 |  |  |  |  |  |  |  |  |  |  |  |  |  |  |  |  |  |  |  |  |  |  |  |  |  |  |  |  |
| 253 | 38 | Ecological restoration | Grassland | 0-10 | 39.40 | 100.17 |  |  |  |  | 2.351 | 0.235 | 3.253 | 0.325 |  |  |  |  |  |  |  |  |  |  |  |  |  |  |  |  |  |  |  |  |  |  |  |  |  |  |  |  |
| 254 | 38 | Ecological restoration | Grassland | 0-10 | 39.40 | 100.17 |  |  |  |  | 5.337 | 0.534 | 5.324 | 0.532 |  |  |  |  |  |  |  |  |  |  |  |  |  |  |  |  |  |  |  |  |  |  |  |  |  |  |  |  |
| 255 | 39 | Ecological restoration | Grassland | 0-10 | 36.22 | 106.38 |  |  |  |  | 4.698 | 0.470 | 4.685 | 0.469 |  |  |  |  |  |  |  |  |  |  |  |  |  |  |  |  |  |  |  |  |  |  |  |  |  |  |  |  |
| 256 | 39 | Ecological restoration | Grassland | 0-10 | 36.22 | 106.38 |  |  |  |  | 4.787 | 0.479 | 4.858 | 0.486 |  |  |  |  |  |  |  |  |  |  |  |  |  |  |  |  |  |  |  |  |  |  |  |  |  |  |  |  |
| 257 | 39 | Ecological restoration | Grassland | ＞20 | 36.22 | 106.38 |  |  |  |  | 2.351 | 0.235 | 2.421 | 0.242 |  |  |  |  |  |  |  |  |  |  |  |  |  |  |  |  |  |  |  |  |  |  |  |  |  |  |  |  |
| 258 | 39 | Ecological restoration | Grassland | ＞20 | 36.22 | 106.38 |  |  |  |  | 5.337 | 0.534 | 5.024 | 0.502 |  |  |  |  |  |  |  |  |  |  |  |  |  |  |  |  |  |  |  |  |  |  |  |  |  |  |  |  |
| 259 | 40 | Ecological restoration | Grassland | ＞20 | 36.22 | 106.40 |  |  |  |  | 4.698 | 0.470 | 4.598 | 0.460 |  |  |  |  |  |  |  |  |  |  |  |  |  |  |  |  |  |  |  |  |  |  |  |  |  |  |  |  |
| 260 | 40 | Ecological restoration | Grassland | ＞20 | 36.22 | 106.40 |  |  |  |  | 4.787 | 0.479 | 5.342 | 0.534 |  |  |  |  |  |  |  |  |  |  |  |  |  |  |  |  |  |  |  |  |  |  |  |  |  |  |  |  |
| 261 | 40 | Ecological restoration | Grassland | ＞20 | 36.22 | 106.40 |  |  |  |  | 2.351 | 0.235 | 3.250 | 0.325 |  |  |  |  |  |  |  |  |  |  |  |  |  |  |  |  |  |  |  |  |  |  |  |  |  |  |  |  |
| 262 | 40 | Ecological restoration | Grassland | ＞20 | 36.22 | 106.40 | 0.500 | 0.040 | 0.480 | #### | ##### | 6.130 | 22.530 | 1.790 | 2.16 | 0.210 | 1.7 | 0.220 |  |  |  |  |  |  |  |  |  |  |  |  |  |  |  |  |  |  |  |  |  |  |  |  |
| 263 | 40 | Ecological restoration | Grassland | ＞20 | 36.22 | 106.40 | 0.500 | 0.040 | 0.490 | #### | ##### | 6.130 | 25.270 | 3.630 | 2.16 | 0.210 | 1.63 | 0.230 |  |  |  |  |  |  |  |  |  |  |  |  |  |  |  |  |  |  |  |  |  |  |  |  |
| 264 | 40 | Ecological restoration | Grassland | ＞20 | 36.22 | 106.40 | 0.500 | 0.040 | 0.500 | 0.010 | ##### | 6.130 | 25.530 | 3.680 | 2.16 | 0.210 | 1.96 | 0.330 |  |  |  |  |  |  |  |  |  |  |  |  |  |  |  |  |  |  |  |  |  |  |  |  |
| 265 | 40 | Ecological restoration | Grassland | ＞20 | 36.22 | 106.40 | 0.500 | 0.040 | 0.510 | #### | ##### | 6.130 | 33.240 | 2.780 | 2.16 | 0.210 | 1.89 | 0.360 |  |  |  |  |  |  |  |  |  |  |  |  |  |  |  |  |  |  |  |  |  |  |  |  |
| 266 | 40 | Ecological restoration | Grassland | ＞20 | 36.22 | 106.40 | 0.500 | 0.040 | 0.510 | #### | ##### | 6.130 | 49.860 | 6.670 | 2.16 | 0.210 | 2.25 | 0.410 |  |  |  |  |  |  |  |  |  |  |  |  |  |  |  |  |  |  |  |  |  |  |  |  |
| 267 | 40 | Ecological restoration | Grassland | ＞20 | 36.22 | 106.40 | 0.500 | 0.040 | 0.530 | 0.010 | ##### | 6.130 | 57.920 | 5.930 | 2.16 | 0.210 | 2.46 | 0.280 |  |  |  |  |  |  |  |  |  |  |  |  |  |  |  |  |  |  |  |  |  |  |  |  |
| 268 | 40 | Ecological restoration | Grassland | ＞20 | 36.22 | 106.40 | 0.570 | 0.010 | 0.541 | 0.010 | 3.845 | 0.040 | 3.212 | 0.030 | 6.05 | 0.020 | 7.13 | 0.020 |  |  |  |  | 58.49 | 8.820 | 87.42 | 4.45 | 46.55 | 5.57 | 76.17 | 3.87 | 1.90 | 0.17 | 2.76 | 0.130 | 1.300 | 0.190 | 1.790 | 0.040 | 0.53 | 0.09 | 0.770 | 0.010 |
| 269 | 41 | Ecological restoration | Grassland | ＞20 | 47.45 | 126.98 | 0.570 | 0.010 | 0.554 | #### | 3.644 | 0.036 | 2.989 | 0.020 | 3.24 | 0.020 | 11.03 | 0.020 |  |  |  |  | 58.49 | 8.820 | 132.85 | 5.02 | 46.55 | 5.57 | 112.86 | 7.19 | 1.90 | 0.17 | 3.82 | 0.100 | 1.300 | 0.190 | 2.470 | 0.060 | 0.53 | 0.09 | 0.800 | 0.020 |
| 270 | 41 | Ecological restoration | Grassland | ＞20 | 47.45 | 126.98 | 0.570 | 0.010 | 0.497 | 0.010 | 3.845 | 0.040 | 3.001 | 0.030 | 6.05 | 0.020 | 5.44 | 0.040 |  |  |  |  | 58.49 | 8.820 | 184.66 | 5.63 | 46.55 | 5.57 | 157.4 | 6.70 | 1.90 | 0.17 | 3.25 | 0.130 | 1.300 | 0.190 | 2.300 | 0.060 | 0.53 | 0.09 | 0.860 | 0.020 |
| 271 | 41 | Ecological restoration | Grassland | ＞20 | 47.45 | 126.98 | 0.522 | 0.020 | 0.503 | 0.010 | 3.644 | 0.036 | 2.014 | 0.005 | 3.24 | 0.020 | 14.39 | 0.030 |  |  |  |  | 106.29 | 7.810 | 158.46 | 5.43 | 206.9 | 15.72 | 352.5 | 14.03 | 3.76 | 0.11 | 3.45 | 0.070 | 2.600 | 0.040 | 2.010 | 0.120 | 0.89 | 0.02 | 0.790 | 0.080 |
| 272 | 41 | Ecological restoration | Grassland | ＞20 | 47.45 | 126.98 | 0.522 | 0.020 | 0.514 | #### | 3.845 | 0.040 | 2.421 | 0.020 | 6.05 | 0.020 | 10.27 | 0.005 |  |  |  |  | 106.29 | 7.810 | 186.56 | 7.06 | 206.9 | 15.72 | 508.6 | 12.46 | 3.76 | 0.11 | 3.13 | 0.100 | 2.600 | 0.040 | 2.190 | 0.090 | 0.89 | 0.02 | 0.830 | 0.010 |
| 273 | 41 | Ecological restoration | Forest | ＞20 | 47.45 | 126.98 | 0.522 | 0.020 | 0.526 | 0.010 | 3.644 | 0.036 | 2.312 | 0.010 | 3.24 | 0.020 | 12.28 | 0.010 |  |  |  |  | 106.29 | 7.810 | 218.16 | 8.53 | 206.9 | 15.72 | 571.62 | 18.77 | 3.76 | 0.11 | 3.36 | 0.050 | 2.600 | 0.040 | 2.420 | 0.200 | 0.89 | 0.02 | 0.870 | 0.050 |
| 274 | 41 | Ecological restoration | Forest | ＞20 | 47.45 | 126.98 | 9.430 | 0.500 | 7.110 | #### |  |  |  |  |  |  |  |  |  |  |  |  |  |  |  |  |  |  |  |  | 17.00 | 0.00 | 15.00 | 0.000 | 1.000 | 0.080 | 1.000 | 0.070 | 0.580 | 0.050 | 0.610 | 0.030 |
| 275 | 41 | Ecological restoration | Forest | ＞20 | 47.45 | 126.98 | 9.620 | 0.500 | 7.550 | #### |  |  |  |  |  |  |  |  |  |  |  |  |  |  |  |  |  |  |  |  | 17.00 | 0.00 | 15.00 | 0.000 | 1.000 | 0.080 | 1.000 | 0.070 | 0.580 | 0.050 | 0.610 | 0.030 |
| 276 | 41 | Ecological restoration | Forest | ＞20 | 47.45 | 126.98 | 9.090 | 0.580 | 7.510 | #### |  |  |  |  |  |  |  |  |  |  |  |  |  |  |  |  |  |  |  |  | 17.00 | 0.00 | 15.00 | 0.000 | 1.000 | 0.080 | 1.000 | 0.070 | 0.580 | 0.050 | 0.610 | 0.030 |
| 277 | 42 | Ecological restoration | Grassland | ＞20 | 36.22 | 106.26 |  |  |  |  | 0.591 | 0.010 | 0.832 | 0.080 |  |  |  |  |  |  |  |  |  |  |  |  |  |  |  |  |  |  |  |  | 0.540 | 0.020 | 1.210 | 0.015 |  |  |  |  |
| 278 | 42 | Ecological restoration | Grassland | ＞20 | 36.22 | 106.26 |  |  |  |  | 0.591 | 0.010 | 1.070 | 1.010 |  |  |  |  |  |  |  |  |  |  |  |  |  |  |  |  |  |  |  |  | 0.540 | 0.020 | 1.180 | 0.200 |  |  |  |  |
| 279 | 42 | Ecological restoration | Grassland | ＞20 | 36.22 | 106.26 |  |  |  |  | 0.591 | 0.010 | 0.940 | 0.010 |  |  |  |  |  |  |  |  |  |  |  |  |  |  |  |  |  |  |  |  | 0.540 | 0.020 | 1.430 | 0.350 |  |  |  |  |
| 280 | 42 | Ecological restoration | Grassland | ＞20 | 36.22 | 106.26 |  |  |  |  | 0.181 | 0.010 | 0.192 | 0.020 |  |  |  |  |  |  |  |  |  |  |  |  |  |  |  |  |  |  |  |  | 0.340 | 0.010 | 1.120 | 0.020 |  |  |  |  |
| 281 | 42 | Ecological restoration | Grassland | ＞20 | 36.22 | 106.26 |  |  |  |  | 0.181 | 0.010 | 0.220 | 0.030 |  |  |  |  |  |  |  |  |  |  |  |  |  |  |  |  |  |  |  |  | 0.340 | 0.010 | 0.700 | 0.010 |  |  |  |  |
| 282 | 42 | Ecological restoration | Grassland | ＞20 | 36.22 | 106.26 |  |  |  |  | 0.181 | 0.010 | 1.200 | 0.230 |  |  |  |  |  |  |  |  |  |  |  |  |  |  |  |  |  |  |  |  | 0.340 | 0.010 | 2.410 | 0.020 |  |  |  |  |
| 283 | 42 | Ecological restoration | Grassland | ＞20 | 36.22 | 106.26 | 4.290 | 0.120 | 4.830 | #### |  |  |  |  |  |  |  |  | 64.23 | 2.82 | 47.36 | 6.60 |  |  |  |  |  |  |  |  | 3.19 | 0.09 | 2.17 | 0.100 | 1.830 | 0.180 | 2.24 | 0.15 | 7.46 | 0.43 | 9.150 | 0.550 |
| 284 | 43 | Ecological restoration | Grassland | ＞20 | 36.17 | 106.35 | 4.290 | 0.120 | 5.170 | #### |  |  |  |  |  |  |  |  | 64.23 | 2.82 | 108.54 | 7.91 |  |  |  |  |  |  |  |  | 3.19 | 0.09 | 2.32 | 0.060 | 1.830 | 0.180 | 2.200 | 0.090 | 7.46 | 0.43 | 9.060 | 0.370 |
| 285 | 43 | Ecological restoration | Grassland | ＞20 | 36.17 | 106.35 | 4.290 | 0.120 | 4.540 | 0.100 |  |  |  |  |  |  |  |  | 64.23 | 2.82 | 114.31 | 5.13 |  |  |  |  |  |  |  |  | 3.19 | 0.09 | 3.04 | 0.130 | 1.830 | 0.180 | 2.020 | 0.090 | 7.46 | 0.43 | 6.550 | 0.410 |
| 286 | 43 | Ecological restoration | Grassland | ＞20 | 36.17 | 106.35 | 3.860 | 0.130 | 4.940 | #### |  |  |  |  |  |  |  |  | 83.10 | 3.60 | 73.32 | 7.06 |  |  |  |  |  |  |  |  | 2.75 | 0.12 | 2.17 | 0.120 | 2.260 | 0.130 | 2.650 | 0.100 | 4.99 | 0.54 | 9.320 | 0.820 |
| 287 | 43 | Ecological restoration | Grassland | ＞20 | 36.17 | 106.35 | 3.860 | 0.130 | 5.370 | 0.110 |  |  |  |  |  |  |  |  | 83.10 | 3.60 | 104.71 | 8.13 |  |  |  |  |  |  |  |  | 2.75 | 0.12 | 2.61 | 0.180 | 2.260 | 0.130 | 2.460 | 0.120 | 4.99 | 0.54 | 8.360 | 0.490 |
| 288 | 44 | Ecological restoration | Grassland | 0-10 | 36.77 | 109.27 | 3.860 | 0.130 | 4.420 | #### |  |  |  |  |  |  |  |  | 83.10 | 3.60 | 112.01 | 7.57 |  |  |  |  |  |  |  |  | 2.75 | 0.12 | 3.01 | 0.130 | 2.260 | 0.130 | 2.440 | 0.100 | 4.99 | 0.54 | 5.820 | 0.340 |
| 289 | 44 | Ecological restoration | Grassland | 0-10 | 36.77 | 109.27 | 3.480 | 0.040 | 5.010 | #### |  |  |  |  |  |  |  |  | 52.81 | 2.39 | 49.54 | 3.84 |  |  |  |  |  |  |  |  | 1.85 | 0.11 | 1.88 | 0.080 | 2.010 | 0.150 | 2.480 | 0.140 | 5.83 | 0.45 | 7.140 | 0.550 |
| 290 | 44 | Ecological restoration | Grassland | 10-20 | 36.77 | 109.27 | 3.480 | 0.040 | 5.480 | #### |  |  |  |  |  |  |  |  | 52.81 | 2.39 | 79.39 | 8.42 |  |  |  |  |  |  |  |  | 1.85 | 0.11 | 2.46 | 0.110 | 2.010 | 0.150 | 2.260 | 0.140 | 5.83 | 0.45 | 6.990 | 0.490 |
| 291 | 44 | Ecological restoration | Grassland | 10-20 | 36.77 | 109.27 | 3.480 | 0.040 | 4.330 | #### |  |  |  |  |  |  |  |  | 52.81 | 2.39 | 97.48 | 4.43 |  |  |  |  |  |  |  |  | 1.85 | 0.11 | 2.75 | 0.120 | 2.010 | 0.150 | 2.240 | 0.120 | 5.83 | 0.45 | 5.300 | 0.410 |
| 292 | 44 | Ecological restoration | Grassland | ＞20 | 36.77 | 109.27 | 0.410 | 0.010 | 0.520 | #### |  |  |  |  | 11.870 | 0.080 | 14.05 | 0.070 | 0.02 | 0.01 | 0.28 | 0.04 |  |  |  |  |  |  |  |  | 0.72 | 0.32 | 3.22 | 0.400 | 0.780 | 0.510 | 2.410 | 0.180 |  |  |  |  |
| 293 | 45 | Ecological restoration | Grassland | 0-10 | 37.43 | 109.55 | 0.410 | 0.010 | 0.260 | 0.010 |  |  |  |  | 11.870 | 0.080 | 12.75 | 0.010 | 0.02 | 0.01 | 0.28 | 0.04 |  |  |  |  |  |  |  |  | 0.72 | 0.32 | 3.22 | 0.400 | 0.780 | 0.510 | 2.410 | 0.180 |  |  |  |  |
| 294 | 45 | Ecological restoration | Grassland | 10-20 | 37.43 | 109.55 | 0.410 | 0.010 | 0.430 | 0.010 |  |  |  |  | 11.870 | 0.080 | 12.34 | 0.000 | 0.02 | 0.01 | 0.28 | 0.04 |  |  |  |  |  |  |  |  | 0.72 | 0.32 | 3.22 | 0.400 | 0.780 | 0.510 | 2.410 | 0.180 |  |  |  |  |
| 295 | 45 | Ecological restoration | Grassland | ＞20 | 37.43 | 109.55 | 0.290 | 0.010 | 0.490 | 0.010 |  |  |  |  | 12.140 | 0.077 | 10.02 | 0.010 | 0.02 | 0.01 | 0.01 | 0.00 |  |  |  |  |  |  |  |  | 0.72 | 0.32 | 1.04 | 0.510 | 0.780 | 0.510 | 0.820 | 0.231 |  |  |  |  |
| 296 | 45 | Ecological restoration | Grassland | ＞20 | 37.43 | 109.55 | 0.290 | 0.010 | 0.200 | 0.010 |  |  |  |  | 12.140 | 0.077 | 9.69 | 0.020 | 0.02 | 0.01 | 0.01 | 0.00 |  |  |  |  |  |  |  |  | 0.72 | 0.32 | 1.04 | 0.510 | 0.780 | 0.510 | 0.820 | 0.231 |  |  |  |  |
| 297 | 45 | Ecological restoration | Grassland | ＞20 | 37.43 | 109.55 | 0.290 | 0.010 | 0.410 | 0.010 |  |  |  |  | 12.140 | 0.077 | 7.89 | 0.020 | 0.02 | 0.01 | 0.01 | 0.00 |  |  |  |  |  |  |  |  | 0.72 | 0.32 | 1.04 | 0.510 | 0.780 | 0.510 | 0.820 | 0.231 |  |  |  |  |
| 298 | 46 | Ecological restoration | Grassland | 0-10 | 37.43 | 108.07 | 0.380 | 0.010 | 0.420 | 0.010 |  |  |  |  | 9.210 | 0.000 | 12.13 | 0.000 | 0.02 | 0.01 | 0.23 | 0.03 |  |  |  |  |  |  |  |  | 0.72 | 0.32 | 1.64 | 0.452 | 0.780 | 0.510 | 1.560 | 0.530 |  |  |  |  |
| 299 | 46 | Ecological restoration | Forest | 0-10 | 34.30 | 108.07 | 0.380 | 0.010 | 0.180 | 0.010 |  |  |  |  | 9.210 | 0.000 | 12.04 | 0.010 | 0.02 | 0.01 | 0.23 | 0.03 |  |  |  |  |  |  |  |  | 0.72 | 0.32 | 1.64 | 0.452 | 0.780 | 0.510 | 1.560 | 0.530 |  |  |  |  |
| 300 | 47 | Ecological restoration | Grassland | 0-10 | 34.78 | 100.35 | 0.380 | 0.010 | 0.390 | 0.010 |  |  |  |  | 9.210 | 0.000 | 11.07 | 0.080 | 0.02 | 0.01 | 0.23 | 0.03 |  |  |  |  |  |  |  |  | 0.72 | 0.32 | 1.64 | 0.452 | 0.780 | 0.510 | 1.560 | 0.530 |  |  |  |  |
| 301 | 47 | Ecological restoration | Grassland | 10-20 | 34.78 | 100.35 | 0.200 | 0.020 | 0.210 | #### |  |  |  |  |  |  |  |  | 192.20 | 19.22 | 260.15 | 26.02 | 108.90 | 10.89 | ##### | 94.20 |  |  |  |  | 1.96 | 0.20 | 6.47 | 0.647 | 0.170 | 0.017 | 0.620 | 0.062 |  |  |  |  |
| 302 | 47 | Ecological restoration | Grassland | 10-20 | 34.78 | 100.35 | 0.200 | 0.020 | 0.230 | #### |  |  |  |  |  |  |  |  | 192.20 | 19.22 | 157.83 | 15.78 | 108.90 | 10.89 | ##### | 32.06 |  |  |  |  | 1.96 | 0.20 | 5.41 | 0.541 | 0.170 | 0.017 | 0.690 | 0.069 |  |  |  |  |
| 303 | 47 | Ecological restoration | Grassland | ＞20 | 34.78 | 100.35 | 0.200 | 0.020 | 0.250 | #### |  |  |  |  |  |  |  |  | 192.20 | 19.22 | ##### | 32.05 | 108.90 | 10.89 | ##### | 125.4 |  |  |  |  | 1.96 | 0.20 | 4.87 | 0.487 | 0.170 | 0.017 | 0.410 | 0.041 |  |  |  |  |
| 304 | 48 | Ecological restoration | Grassland | 0-10 | 28.51 | 60.86 | 0.200 | 0.020 | 0.210 | #### |  |  |  |  |  |  |  |  | 192.20 | 19.22 | 89.67 | 8.97 | 108.90 | 10.89 | ##### | 20.05 |  |  |  |  | 1.96 | 0.20 | 3.26 | 0.326 | 0.170 | 0.017 | 0.254 | 0.025 |  |  |  |  |
| 305 | 48 | Ecological restoration | Grassland | 0-10 | 28.51 | 60.86 | 0.200 | 0.020 | 0.200 | #### |  |  |  |  |  |  |  |  | 192.20 | 19.22 | ##### | 36.02 | 108.90 | 10.89 | ##### | 128.9 |  |  |  |  | 1.96 | 0.20 | 5.01 | 0.501 | 0.170 | 0.017 | 0.171 | 0.017 |  |  |  |  |
| 306 | 48 | Ecological restoration | Grassland | 0-10 | 28.51 | 60.86 | 0.200 | 0.020 | 0.200 | 0.010 |  |  |  |  |  |  |  |  | 192.20 | 19.22 | 40.28 | 4.03 | 108.90 | 10.89 | 189.67 | 18.97 |  |  |  |  | 1.96 | 0.20 | 5.03 | 0.503 | 0.170 | 0.017 | 0.230 | 0.023 |  |  |  |  |
| 307 | 49 | Ecological restoration | Grassland | 0-10 | 37.18 | 102.77 | 0.200 | 0.020 | 0.200 | #### |  |  |  |  |  |  |  |  | 192.20 | 19.22 | 260.15 | 26.02 | 108.90 | 10.89 | ##### | 94.20 |  |  |  |  | 1.96 | 0.20 | 6.47 | 0.647 | 0.170 | 0.017 | 0.620 | 0.062 |  |  |  |  |
| 308 | 49 | Ecological restoration | Grassland | 0-10 | 37.18 | 102.77 | 0.200 | 0.020 | 0.220 | 0.010 |  |  |  |  |  |  |  |  | 192.20 | 19.22 | 157.83 | 15.78 | 108.90 | 10.89 | ##### | 32.06 |  |  |  |  | 1.96 | 0.20 | 5.41 | 0.541 | 0.170 | 0.017 | 0.690 | 0.069 |  |  |  |  |
| 309 | 49 | Ecological restoration | Grassland | 0-10 | 37.18 | 102.77 | 0.200 | 0.020 | 0.220 | #### |  |  |  |  |  |  |  |  | 192.20 | 19.22 | ##### | 32.05 | 108.90 | 10.89 | ##### | 125.4 |  |  |  |  | 1.96 | 0.20 | 4.87 | 0.487 | 0.170 | 0.017 | 0.410 | 0.041 |  |  |  |  |
| 310 | 49 | Ecological restoration | Grassland | 10-20 | 37.18 | 102.77 | 0.200 | 0.020 | 0.190 | #### |  |  |  |  |  |  |  |  | 192.20 | 19.22 | 89.67 | 8.97 | 108.90 | 10.89 | ##### | 20.05 |  |  |  |  | 1.96 | 0.20 | 3.26 | 0.326 | 0.170 | 0.017 | 0.254 | 0.025 |  |  |  |  |
| 311 | 50 | Ecological restoration | Grassland | 0-10 | 36.85 | 109.32 | 0.200 | 0.020 | 0.180 | 0.010 |  |  |  |  |  |  |  |  | 192.20 | 19.22 | ##### | 36.02 | 108.90 | 10.89 | ##### | 128.9 |  |  |  |  | 1.96 | 0.20 | 5.01 | 0.501 | 0.170 | 0.017 | 0.171 | 0.017 |  |  |  |  |
| 312 | 50 | Ecological restoration | Grassland | 10-20 | 36.85 | 109.32 | 0.200 | 0.020 | 0.180 | 0.010 |  |  |  |  |  |  |  |  | 192.20 | 19.22 | 40.28 | 4.03 | 108.90 | 10.89 | 189.67 | 18.97 |  |  |  |  | 1.96 | 0.20 | 5.03 | 0.503 | 0.170 | 0.017 | 0.230 | 0.023 |  |  |  |  |
| 313 | 50 | Ecological restoration | Grassland | ＞20 | 36.85 | 109.32 | 0.250 | 0.025 | 0.320 | #### |  |  |  |  |  |  |  |  | 175.08 | 17.51 | ##### | 23.06 | 20.31 | 2.031 | 217.69 | 21.77 | ##### | 20.87 | 60.39 | 6.04 | 5.87 | 0.59 | 8.79 | 0.879 | 1.420 | 0.142 | 1.580 | 0.158 |  |  |  |  |
| 314 | 50 | Ecological restoration | Grassland | ＞20 | 36.85 | 109.32 | 0.220 | 0.022 | 0.290 | #### |  |  |  |  |  |  |  |  | 175.08 | 17.51 | ##### | 23.06 | 50.06 | 5.006 | 170.48 | 17.05 | ##### | 20.87 | 60.39 | 6.04 | 5.87 | 0.59 | 8.79 | 0.879 | 1.420 | 0.142 | 1.580 | 0.158 |  |  |  |  |
| 315 | 51 | Ecological restoration | Grassland | 0-10 | 36.77 | 109.27 | 0.210 | 0.021 | 0.300 | #### |  |  |  |  |  |  |  |  | 175.08 | 17.51 | ##### | 23.06 | 48.32 | 4.832 | 100.24 | 10.02 | ##### | 20.87 | 60.39 | 6.04 | 5.87 | 0.59 | 8.79 | 0.879 | 1.420 | 0.142 | 1.580 | 0.158 |  |  |  |  |
| 316 | 51 | Ecological restoration | Grassland | 0-10 | 36.77 | 109.27 | 0.230 | 0.023 | 0.310 | 0.031 |  |  |  |  |  |  |  |  | 175.08 | 17.51 | ##### | 23.06 | 40.71 | 4.071 | 50.23 | 5.02 | ##### | 20.87 | 60.39 | 6.04 | 5.87 | 0.59 | 8.79 | 0.879 | 1.420 | 0.142 | 1.580 | 0.158 |  |  |  |  |
| 317 | 51 | Ecological restoration | Grassland | 10-20 | 36.77 | 109.27 | 0.270 | 0.027 | 0.380 | #### |  |  |  |  |  |  |  |  | 175.08 | 17.51 | ##### | 23.06 | 50.87 | 5.087 | 52.34 | 5.23 | ##### | 20.87 | 60.39 | 6.04 | 5.87 | 0.59 | 8.79 | 0.879 | 1.420 | 0.142 | 1.580 | 0.158 |  |  |  |  |
| 318 | 51 | Ecological restoration | Grassland | 10-20 | 36.77 | 109.27 | 0.320 | 0.032 | 0.470 | #### |  |  |  |  |  |  |  |  | 175.08 | 17.51 | ##### | 23.06 | 47.39 | 4.739 | 48.97 | 4.90 | ##### | 20.87 | 60.39 | 6.04 | 5.87 | 0.59 | 8.79 | 0.879 | 1.420 | 0.142 | 1.580 | 0.158 |  |  |  |  |
| 319 | 51 | Ecological restoration | Grassland | ＞20 | 36.77 | 109.27 | 0.420 | 0.042 | 0.540 | #### |  |  |  |  |  |  |  |  | 175.08 | 17.51 | ##### | 23.06 | 45.11 | 4.511 | 45.37 | 4.54 | ##### | 20.87 | 60.39 | 6.04 | 5.87 | 0.59 | 8.79 | 0.879 | 1.420 | 0.142 | 1.580 | 0.158 |  |  |  |  |
| 320 | 52 | Ecological restoration | Grassland | 10-20 | 47.45 | 126.92 | 0.200 | 0.000 | 0.210 | #### | 0.630 | 0.020 | 1.330 | 0.030 | 4.06 | 0.810 | 8.76 | 0.140 | 4.33 | 0.67 | 16.31 | 1.69 | 24.50 | 4.390 | 129.18 | 10.95 |  |  |  |  | 7.00 | 1.00 | 10.00 | 2.000 | 1.270 | 0.120 | 1.700 | 0.090 |  |  |  |  |
| 321 | 52 | Ecological restoration | Grassland | 10-20 | 47.45 | 126.92 | 0.200 | 0.000 | 0.200 | 0.010 | 0.630 | 0.020 | 0.950 | 0.010 | 4.06 | 0.810 | 5.89 | 0.220 | 4.33 | 0.67 | 15.29 | 1.99 | 24.50 | 4.390 | 72.33 | 12.35 |  |  |  |  | 7.00 | 1.00 | 10.00 | 2.000 | 1.270 | 0.120 | 1.650 | 0.070 |  |  |  |  |
| 326 | 53 | Ecological restoration | Grassland | 0-10 | 44.07 | 125.70 | 0.200 | 0.000 | 5.150 | 0.120 | 0.630 | 0.020 | 1.600 | 0.030 | 4.06 | 0.810 | 9.63 | 0.580 | 4.33 | 0.67 | 16.58 | 0.84 | 24.50 | 4.390 | 204.01 | 21.73 |  |  |  |  | 7.00 | 1.00 | 9.00 | 3.000 | 1.270 | 0.120 | 0.770 | 0.120 |  |  |  |  |
| 327 | 53 | Ecological restoration | Grassland | 0-10 | 44.07 | 125.70 | 0.200 | 0.000 | 0.210 | #### |  |  |  |  |  |  |  |  | 4.33 | 0.67 | 16.31 | 1.69 | 24.50 | 4.390 | 129.18 | 10.95 |  |  |  |  |  |  |  |  | ##### | 1.770 | ##### | 3.330 |  |  |  |  |
| 328 | 53 | Ecological restoration | Grassland | 0-10 | 44.07 | 125.70 | 0.200 | 0.000 | 0.200 | 0.010 |  |  |  |  |  |  |  |  | 4.33 | 0.67 | 15.29 | 1.990 | 24.50 | 4.390 | 72.36 | 12.35 |  |  |  |  |  |  |  |  | ##### | 1.770 | ##### | 3.430 |  |  |  |  |
| 351 | 59 | Ecological restoration | Grassland | 0-10 | 37.72 | 107.35 | 0.200 | 0.000 | 0.290 | 0.010 |  |  |  |  |  |  |  |  | 4.33 | 0.67 | 16.58 | 0.84 | 24.50 | 4.390 | 250.01 | 21.73 |  |  |  |  |  |  |  |  | ##### | 1.770 | ##### | 2.110 |  |  |  |  |
| 358 | 61 | Ecological restoration | Forest | 0-10 | 24.79 | 118.63 | 1.198 | 0.120 | 1.124 | 0.112 |  |  |  |  |  |  |  |  | ##### | 14.64 | 310.03 | 31.00 | ##### | 159.94 | ##### | 100.73 | 38.37 | 3.837 | 142.6 | 14.26 | 18.04 | 1.80 | 23.21 | 2.321 |  |  |  |  |  |  |  |  |
| 359 | 61 | Ecological restoration | Forest | 10-20 | 24.79 | 118.63 | 1.198 | 0.120 | 1.207 | 0.121 |  |  |  |  |  |  |  |  | ##### | 14.64 | ##### | 23.43 | ##### | 159.94 | ##### | 108.73 | 38.37 | 3.837 | 98.68 | 9.87 | 18.04 | 1.80 | 25.34 | 2.534 |  |  |  |  |  |  |  |  |
| 360 | 61 | Ecological restoration | Forest | ＞20 | 24.79 | 118.63 | 1.198 | 0.120 | 1.060 | 0.106 |  |  |  |  |  |  |  |  | ##### | 14.64 | 210.01 | 21.00 | ##### | 159.94 | ##### | 158.72 | 38.37 | 3.837 | 60.07 | 6.01 | 18.04 | 1.80 | 23.29 | 2.329 |  |  |  |  |  |  |  |  |
| 361 | 62 | Ecological restoration | Grassland | 0-10 | 36.22 | 106.43 | 0.470 | 0.047 | 0.580 | #### |  |  |  |  |  |  |  |  |  |  |  |  |  |  |  |  |  |  |  |  | 4.50 | 0.60 | 8.50 | 1.300 |  |  |  |  |  |  |  |  |
| 362 | 62 | Ecological restoration | Grassland | 0-10 | 36.22 | 106.43 | 0.470 | 0.047 | 0.451 | #### |  |  |  |  |  |  |  |  |  |  |  |  |  |  |  |  |  |  |  |  | 4.50 | 0.60 | 6.50 | 0.600 |  |  |  |  |  |  |  |  |
| 363 | 62 | Ecological restoration | Grassland | 10-20 | 36.22 | 106.43 | 0.470 | 0.047 | 0.568 | #### |  |  |  |  |  |  |  |  |  |  |  |  |  |  |  |  |  |  |  |  | 4.50 | 0.60 | 10.00 | 1.200 |  |  |  |  |  |  |  |  |
| 364 | 62 | Ecological restoration | Grassland | ＞20 | 36.22 | 106.43 | 0.470 | 0.047 | 0.321 | #### |  |  |  |  |  |  |  |  |  |  |  |  |  |  |  |  |  |  |  |  | 4.50 | 0.60 | 7.80 | 1.000 |  |  |  |  |  |  |  |  |
| 365 | 62 | Ecological restoration | Grassland | ＞20 | 36.22 | 106.43 | 0.512 | 0.051 | 0.551 | #### |  |  |  |  |  |  |  |  |  |  |  |  |  |  |  |  |  |  |  |  | 4.50 | 0.60 | 8.50 | 1.300 |  |  |  |  |  |  |  |  |
| 366 | 63 | Ecological restoration | Grassland | 0-10 | 36.17 | 106.35 | 0.512 | 0.051 | 0.401 | #### |  |  |  |  |  |  |  |  |  |  |  |  |  |  |  |  |  |  |  |  | 4.50 | 0.60 | 6.50 | 0.600 |  |  |  |  |  |  |  |  |
| 367 | 63 | Ecological restoration | Grassland | 0-10 | 36.17 | 106.35 | 0.512 | 0.051 | 0.543 | #### |  |  |  |  |  |  |  |  |  |  |  |  |  |  |  |  |  |  |  |  | 4.50 | 0.60 | 10.00 | 1.200 |  |  |  |  |  |  |  |  |
| 368 | 63 | Ecological restoration | Grassland | 0-10 | 36.17 | 106.35 | 0.512 | 0.051 | 0.362 | #### |  |  |  |  |  |  |  |  |  |  |  |  |  |  |  |  |  |  |  |  | 4.50 | 0.60 | 7.80 | 1.000 |  |  |  |  |  |  |  |  |
| 369 | 63 | Ecological restoration | Grassland | 0-10 | 36.17 | 106.35 | 0.421 | 0.042 | 0.536 | #### |  |  |  |  |  |  |  |  |  |  |  |  |  |  |  |  |  |  |  |  | 4.50 | 0.60 | 8.50 | 1.300 |  |  |  |  |  |  |  |  |
| 370 | 63 | Ecological restoration | Grassland | 0-10 | 36.17 | 106.35 | 0.421 | 0.042 | 0.404 | #### |  |  |  |  |  |  |  |  |  |  |  |  |  |  |  |  |  |  |  |  | 4.50 | 0.60 | 6.50 | 0.600 |  |  |  |  |  |  |  |  |
| 371 | 63 | Ecological restoration | Grassland | 0-10 | 36.17 | 106.35 | 0.421 | 0.042 | 0.510 | 0.051 |  |  |  |  |  |  |  |  |  |  |  |  |  |  |  |  |  |  |  |  | 4.50 | 0.60 | 10.00 | 1.200 |  |  |  |  |  |  |  |  |
| 372 | 63 | Ecological restoration | Grassland | 0-10 | 36.17 | 106.35 | 0.421 | 0.042 | 0.253 | #### |  |  |  |  |  |  |  |  |  |  |  |  |  |  |  |  |  |  |  |  | 4.50 | 0.60 | 7.80 | 1.000 |  |  |  |  |  |  |  |  |
| 373 | 63 | Ecological restoration | Grassland | 0-10 | 36.17 | 106.35 | 0.32 | 0.032 | 0.356 | #### | 4.810 | 0.481 | 4.070 | 0.407 |  |  |  |  |  |  |  |  |  |  |  |  |  |  |  |  | 2.24 | 0.22 | 3.25 | 0.325 | 0.78 | 0.08 | 1.240 | 0.124 |  |  |  |  |
| 374 | 63 | Ecological restoration | Grassland | 10-20 | 36.17 | 106.35 | 0.32 | 0.032 | 0.367 | #### | 4.810 | 0.481 | 2.790 | 0.279 |  |  |  |  |  |  |  |  |  |  |  |  |  |  |  |  | 2.24 | 0.22 | 5.12 | 0.512 | 0.78 | 0.08 | 1.247 | 0.125 |  |  |  |  |
| 375 | 63 | Ecological restoration | Grassland | 10-20 | 36.17 | 106.35 | 1.120 | 0.050 | 1.250 | 0.125 | 2.120 | 0.050 | 2.600 | 0.060 |  |  |  |  |  |  |  |  |  |  |  |  |  |  |  |  | 10.78 | 0.74 | 13.00 | 0.620 |  |  |  |  |  |  |  |  |
| 376 | 63 | Ecological restoration | Grassland | 10-20 | 36.17 | 106.35 | 1.120 | 0.050 | 1.180 | 0.170 | 2.120 | 0.050 | 3.160 | 0.240 |  |  |  |  |  |  |  |  |  |  |  |  |  |  |  |  | 10.78 | 0.74 | 8.56 | 0.760 |  |  |  |  |  |  |  |  |
| 377 | 63 | Ecological restoration | Grassland | 10-20 | 36.17 | 106.35 | 1.120 | 0.050 | 2.600 | #### | 2.120 | 0.050 | 3.270 | 0.130 |  |  |  |  |  |  |  |  |  |  |  |  |  |  |  |  | 10.78 | 0.74 | 6.78 | 0.740 |  |  |  |  |  |  |  |  |
| 378 | 63 | Ecological restoration | Grassland | 10-20 | 36.17 | 106.35 | 1.120 | 0.050 | 2.240 | #### | 2.120 | 0.050 | 3.840 | 0.500 |  |  |  |  |  |  |  |  |  |  |  |  |  |  |  |  | 10.78 | 0.74 | 7.44 | 0.900 |  |  |  |  |  |  |  |  |
| 379 | 63 | Ecological restoration | Grassland | 10-20 | 36.17 | 106.35 | 0.640 | 0.020 | 0.650 | 0.010 | 6.200 | 0.970 | 5.370 | 1.050 |  |  |  |  |  |  |  |  |  |  |  |  |  |  |  |  | 6.85 | 0.20 | 6.99 | 0.080 | 6.780 | 0.210 | 6.930 | 0.080 |  |  |  |  |
| 380 | 63 | Ecological restoration | Grassland | 10-20 | 36.17 | 106.35 | 0.640 | 0.020 | 0.590 | #### | 6.200 | 0.970 | 3.740 | 0.700 |  |  |  |  |  |  |  |  |  |  |  |  |  |  |  |  | 6.85 | 0.20 | 7.06 | 0.040 | 6.780 | 0.210 | 6.990 | 0.040 |  |  |  |  |
| 381 | 63 | Ecological restoration | Grassland | 10-20 | 36.17 | 106.35 | 0.640 | 0.020 | 0.600 | #### | 6.200 | 0.970 | 4.670 | 1.280 |  |  |  |  |  |  |  |  |  |  |  |  |  |  |  |  | 6.85 | 0.20 | 7.06 | 0.110 | 6.780 | 0.210 | 6.990 | 0.110 |  |  |  |  |
| 382 | 63 | Ecological restoration | Grassland | ＞20 | 36.17 | 106.35 | 0.640 | 0.020 | 0.660 | 0.010 | 6.200 | 0.970 | 6.050 | 0.590 |  |  |  |  |  |  |  |  |  |  |  |  |  |  |  |  | 6.85 | 0.20 | 7.13 | 0.100 | 6.780 | 0.210 | 7.050 | 0.100 |  |  |  |  |
| 383 | 63 | Ecological restoration | Grassland | ＞20 | 36.17 | 106.35 | 0.640 | 0.020 | 0.760 | #### | 6.200 | 0.970 | 6.300 | 0.390 |  |  |  |  |  |  |  |  |  |  |  |  |  |  |  |  | 6.85 | 0.20 | 7.06 | 0.170 | 6.780 | 0.210 | 6.980 | 0.160 |  |  |  |  |
| 384 | 63 | Ecological restoration | Grassland | ＞20 | 36.17 | 106.35 | 0.590 | 0.020 | 0.610 | #### | 4.440 | 0.310 | 3.810 | 0.250 |  |  |  |  |  |  |  |  |  |  |  |  |  |  |  |  | 6.76 | 0.12 | 6.63 | 0.180 | 6.690 | 0.120 | 6.570 | 0.170 |  |  |  |  |
| 385 | 63 | Ecological restoration | Grassland | ＞20 | 36.17 | 106.35 | 0.590 | 0.020 | 0.580 | 0.010 | 4.440 | 0.310 | 3.280 | 0.240 |  |  |  |  |  |  |  |  |  |  |  |  |  |  |  |  | 6.76 | 0.12 | 6.97 | 0.180 | 6.690 | 0.120 | 6.900 | 0.190 |  |  |  |  |
| 386 | 63 | Ecological restoration | Grassland | ＞20 | 36.17 | 106.35 | 0.590 | 0.020 | 0.590 | #### | 4.440 | 0.310 | 2.160 | 0.140 |  |  |  |  |  |  |  |  |  |  |  |  |  |  |  |  | 6.76 | 0.12 | 6.86 | 0.080 | 6.690 | 0.120 | 6.790 | 0.080 |  |  |  |  |
| 387 | 63 | Ecological restoration | Grassland | ＞20 | 36.17 | 106.35 | 0.590 | 0.020 | 0.640 | 0.010 | 4.440 | 0.310 | 5.030 | 0.200 |  |  |  |  |  |  |  |  |  |  |  |  |  |  |  |  | 6.76 | 0.12 | 6.83 | 0.090 | 6.690 | 0.120 | 6.760 | 0.090 |  |  |  |  |
| 388 | 63 | Ecological restoration | Grassland | ＞20 | 36.17 | 106.35 | 0.590 | 0.020 | 0.740 | #### | 4.440 | 0.310 | 6.550 | 0.380 |  |  |  |  |  |  |  |  |  |  |  |  |  |  |  |  | 6.76 | 0.12 | 6.96 | 0.150 | 6.690 | 0.120 | 6.890 | 0.140 |  |  |  |  |
| 389 | 63 | Ecological restoration | Grassland | ＞20 | 36.17 | 106.35 | 0.820 | 0.082 | 0.852 | #### | 7.360 | 0.736 | 9.680 | 0.968 |  |  |  |  |  |  |  |  |  |  |  |  |  |  |  |  |  |  |  |  |  |  |  |  |  |  |  |  |
| 397 | 66 | Ecological restoration | Forest | ＞20 | 35.21 | 107.70 | 0.820 | 0.082 | 0.895 | #### | 7.360 | 0.736 | 4.580 | 0.458 |  |  |  |  |  |  |  |  |  |  |  |  |  |  |  |  |  |  |  |  |  |  |  |  |  |  |  |  |
| 398 | 66 | Ecological restoration | Forest | ＞20 | 35.21 | 107.70 | 0.670 | 0.067 | 0.690 | #### | 9.670 | 0.967 | 10.040 | 1.004 |  |  |  |  |  |  |  |  |  |  |  |  |  |  |  |  |  |  |  |  |  |  |  |  |  |  |  |  |
| 399 | 66 | Ecological restoration | Forest | ＞20 | 35.21 | 107.70 | 0.670 | 0.067 | 0.681 | #### | 9.670 | 0.967 | 5.370 | 0.537 |  |  |  |  |  |  |  |  |  |  |  |  |  |  |  |  |  |  |  |  |  |  |  |  |  |  |  |  |
| 400 | 66 | Ecological restoration | Forest | ＞20 | 35.21 | 107.70 | 0.560 | 0.056 | 0.582 | #### | 17.690 | 1.769 | 18.350 | 1.835 |  |  |  |  |  |  |  |  |  |  |  |  |  |  |  |  |  |  |  |  |  |  |  |  |  |  |  |  |
| 401 | 66 | Ecological restoration | Forest | ＞20 | 35.21 | 107.70 | 0.560 | 0.056 | 0.572 | #### | 17.690 | 1.769 | 15.290 | 1.529 |  |  |  |  |  |  |  |  |  |  |  |  |  |  |  |  |  |  |  |  |  |  |  |  |  |  |  |  |
| 402 | 66 | Ecological restoration | Forest | ＞20 | 35.21 | 107.70 | 0.520 | 0.052 | 0.493 | #### | ##### | 2.007 | 18.370 | 1.837 |  |  |  |  |  |  |  |  |  |  |  |  |  |  |  |  |  |  |  |  |  |  |  |  |  |  |  |  |
| 403 | 66 | Ecological restoration | Forest | ＞20 | 35.21 | 107.70 | 0.520 | 0.052 | 0.502 | #### | ##### | 2.007 | 20.340 | 2.034 |  |  |  |  |  |  |  |  |  |  |  |  |  |  |  |  |  |  |  |  |  |  |  |  |  |  |  |  |
| 404 | 66 | Ecological restoration | Forest | ＞20 | 35.21 | 107.70 | 0.610 | 0.061 | 0.642 | #### |  |  |  |  |  |  |  |  | ##### | 21.04 | 602.31 | 60.23 | 360.21 | 36.021 | 371.25 | 37.13 | 43.27 | 4.33 | 78.42 | 7.84 | 7.86 | 0.786 | 9.24 | 0.92 | 1.57 | 0.157 | 1.61 | 0.161 |  |  |  |  |
| 405 | 66 | Ecological restoration | Forest | ＞20 | 35.21 | 107.70 | 0.600 | 0.060 | 0.621 | #### |  |  |  |  |  |  |  |  | ##### | 21.04 | 602.31 | 60.23 | 64.20 | 6.420 | 150.37 | 15.04 | 43.27 | 4.33 | 78.42 | 7.84 | 7.86 | 0.786 | 9.24 | 0.92 | 1.57 | 0.157 | 1.61 | 0.161 |  |  |  |  |
| 406 | 66 | Ecological restoration | Forest | ＞20 | 35.21 | 107.70 | 0.590 | 0.059 | 0.623 | #### |  |  |  |  |  |  |  |  | ##### | 21.04 | 602.31 | 60.23 | 47.31 | 4.731 | 50.30 | 5.03 | 43.27 | 4.33 | 78.42 | 7.84 | 7.86 | 0.786 | 9.24 | 0.92 | 1.57 | 0.157 | 1.61 | 0.161 |  |  |  |  |
| 407 | 66 | Ecological restoration | Grassland | ＞20 | 35.21 | 107.70 | 0.610 | 0.061 | 0.610 | 0.061 |  |  |  |  |  |  |  |  | ##### | 21.04 | 602.31 | 60.23 | 10.24 | 1.024 | 15.04 | 1.50 | 43.27 | 4.33 | 78.42 | 7.84 | 7.86 | 0.786 | 9.24 | 0.92 | 1.57 | 0.157 | 1.61 | 0.161 |  |  |  |  |
| 408 | 66 | Ecological restoration | Grassland | ＞20 | 35.21 | 107.70 | 0.620 | 0.062 | 0.600 | #### |  |  |  |  |  |  |  |  | ##### | 21.04 | 602.31 | 60.23 | 15.27 | 1.527 | 20.61 | 2.06 | 43.27 | 4.33 | 78.42 | 7.84 | 7.86 | 0.786 | 9.24 | 0.92 | 1.57 | 0.157 | 1.61 | 0.161 |  |  |  |  |
| 409 | 66 | Ecological restoration | Grassland | ＞20 | 35.21 | 107.70 | 0.618 | 0.062 | 0.575 | #### |  |  |  |  |  |  |  |  | ##### | 21.04 | 602.31 | 60.23 | 0.04 | 0.004 | 5.13 | 0.51 | 43.27 | 4.33 | 78.42 | 7.84 | 7.86 | 0.786 | 9.24 | 0.92 | 1.57 | 0.157 | 1.61 | 0.161 |  |  |  |  |
| 410 | 66 | Ecological restoration | Grassland | ＞20 | 35.21 | 107.70 | 0.615 | 0.062 | 0.560 | #### |  |  |  |  |  |  |  |  | ##### | 21.04 | 602.31 | 60.23 | 0.02 | 0.002 | 0.10 | 0.01 | 43.27 | 4.33 | 78.42 | 7.84 | 7.86 | 0.786 | 9.24 | 0.92 | 1.57 | 0.157 | 1.61 | 0.161 |  |  |  |  |
| 411 | 66 | Ecological restoration | Grassland | ＞20 | 35.21 | 107.70 | 0.450 | 0.045 | 0.670 | #### | 14.270 | 1.427 | 38.250 | 3.825 |  |  |  |  |  |  |  |  |  |  |  |  |  |  |  |  |  |  |  |  |  |  |  |  |  |  |  |  |
| 412 | 66 | Ecological restoration | Grassland | ＞20 | 35.21 | 107.70 | 0.410 | 0.041 | 0.650 | #### | 11.420 | 1.142 | 39.580 | 3.958 |  |  |  |  |  |  |  |  |  |  |  |  |  |  |  |  |  |  |  |  |  |  |  |  |  |  |  |  |
| 413 | 66 | Ecological restoration | Grassland | ＞20 | 35.21 | 107.70 | 0.520 | 0.052 | 0.660 | #### | 10.020 | 1.002 | 35.170 | 3.517 |  |  |  |  |  |  |  |  |  |  |  |  |  |  |  |  |  |  |  |  |  |  |  |  |  |  |  |  |
| 414 | 66 | Ecological restoration | Grassland | ＞20 | 35.21 | 107.70 | 0.530 | 0.053 | 0.665 | #### | 9.670 | 0.967 | 36.280 | 3.628 |  |  |  |  |  |  |  |  |  |  |  |  |  |  |  |  |  |  |  |  |  |  |  |  |  |  |  |  |
| 415 | 66 | Ecological restoration | Grassland | ＞20 | 35.21 | 107.70 |  |  |  |  | 1.730 | 0.173 | 1.240 | 0.124 |  |  |  |  | ##### | 25.04 | ##### | 23.07 | 180.43 | 18.04 | 182.55 | 18.255 |  |  |  |  |  |  |  |  |  |  |  |  |  |  |  |  |
| 416 | 66 | Ecological restoration | Grassland | ＞20 | 35.21 | 107.70 |  |  |  |  | 1.730 | 0.173 | 1.720 | 0.172 |  |  |  |  | ##### | 25.04 | ##### | 25.54 | 180.43 | 18.04 | ##### | ##### |  |  |  |  |  |  |  |  |  |  |  |  |  |  |  |  |
| 417 | 67 | Ecological restoration | Grassland | 0-10 | 39.35 | 100.12 |  |  |  |  | 1.730 | 0.173 | 2.231 | 0.223 |  |  |  |  | ##### | 25.04 | ##### | 38.96 | 180.43 | 18.04 | ##### | ##### |  |  |  |  |  |  |  |  |  |  |  |  |  |  |  |  |
| 418 | 67 | Ecological restoration | Grassland | 0-10 | 39.35 | 100.12 |  |  |  |  | 1.730 | 0.173 | 1.496 | 0.150 |  |  |  |  | ##### | 25.04 | ##### | 34.07 | 180.43 | 18.04 | ##### | ##### |  |  |  |  |  |  |  |  |  |  |  |  |  |  |  |  |
| 419 | 67 | Ecological restoration | Grassland | 0-10 | 39.35 | 100.12 |  |  |  |  | 1.730 | 0.173 | 1.327 | 0.133 |  |  |  |  | ##### | 25.04 | ##### | 38.06 | 180.43 | 18.04 | 610.04 | 61.004 |  |  |  |  |  |  |  |  |  |  |  |  |  |  |  |  |
| 420 | 67 | Ecological restoration | Grassland | 0-10 | 39.35 | 100.12 | 0.460 | 0.020 | 0.520 | #### |  |  |  |  | 4.9 | 0.180 | 7.75 | 0.920 |  |  |  |  |  |  |  |  |  |  |  |  |  |  |  |  |  |  |  |  |  |  |  |  |
| 421 | 67 | Ecological restoration | Grassland | 0-10 | 39.35 | 100.12 | 0.460 | 0.020 | 0.490 | #### |  |  |  |  | 4.9 | 0.180 | 7.93 | 0.490 |  |  |  |  |  |  |  |  |  |  |  |  |  |  |  |  |  |  |  |  |  |  |  |  |
| 422 | 67 | Ecological restoration | Grassland | 0-10 | 39.35 | 100.12 | 0.460 | 0.020 | 0.510 | #### |  |  |  |  | 4.9 | 0.180 | 8.29 | 0.370 |  |  |  |  |  |  |  |  |  |  |  |  |  |  |  |  |  |  |  |  |  |  |  |  |
| 423 | 67 | Ecological restoration | Grassland | 0-10 | 39.35 | 100.12 | 0.460 | 0.020 | 0.520 | #### |  |  |  |  | 4.9 | 0.180 | 9.07 | 0.390 |  |  |  |  |  |  |  |  |  |  |  |  |  |  |  |  |  |  |  |  |  |  |  |  |
| 424 | 67 | Ecological restoration | Grassland | 0-10 | 39.35 | 100.12 | 0.460 | 0.020 | 0.560 | #### |  |  |  |  | 4.9 | 0.180 | 10.64 | 0.720 |  |  |  |  |  |  |  |  |  |  |  |  |  |  |  |  |  |  |  |  |  |  |  |  |
| 425 | 67 | Ecological restoration | Grassland | 0-10 | 39.35 | 100.12 | 1.070 | 0.040 | 0.800 | #### | ##### | 2.210 | 7.130 | 0.500 |  |  |  |  |  |  |  |  |  |  |  |  |  |  |  |  |  |  |  |  |  |  |  |  |  |  |  |  |
| 426 | 67 | Ecological restoration | Grassland | 0-10 | 39.35 | 100.12 | 1.070 | 0.040 | 0.710 | #### | ##### | 2.210 | 14.200 | 0.830 |  |  |  |  |  |  |  |  |  |  |  |  |  |  |  |  |  |  |  |  |  |  |  |  |  |  |  |  |
| 427 | 67 | Ecological restoration | Grassland | 0-10 | 39.35 | 100.12 | 0.682 | 0.068 | 0.684 | #### |  |  |  |  |  |  |  |  |  |  |  |  |  |  |  |  |  |  |  |  |  |  |  |  |  |  |  |  |  |  |  |  |
| 428 | 67 | Ecological restoration | Grassland | 0-10 | 39.35 | 100.12 | 0.682 | 0.068 | 0.673 | #### |  |  |  |  |  |  |  |  |  |  |  |  |  |  |  |  |  |  |  |  |  |  |  |  |  |  |  |  |  |  |  |  |
| 429 | 67 | Ecological restoration | Grassland | 0-10 | 39.35 | 100.12 | 0.682 | 0.068 | 0.654 | #### |  |  |  |  |  |  |  |  |  |  |  |  |  |  |  |  |  |  |  |  |  |  |  |  |  |  |  |  |  |  |  |  |
| 430 | 67 | Ecological restoration | Grassland | 0-10 | 39.35 | 100.12 | 0.682 | 0.068 | 0.697 | #### |  |  |  |  |  |  |  |  |  |  |  |  |  |  |  |  |  |  |  |  |  |  |  |  |  |  |  |  |  |  |  |  |
| 431 | 67 | Ecological restoration | Grassland | 0-10 | 39.35 | 100.12 |  |  |  |  | 3.120 | 0.100 | 7.210 | 0.200 |  |  |  |  |  |  |  |  |  |  |  |  | 10.03 | 3.43 | 17.34 | 3.21 | 9.00 | 0.10 | 11.44 | 0.200 | 0.210 | 0.000 | 1.170 | 0.100 |  |  |  |  |
| 432 | 67 | Ecological restoration | Grassland | 0-10 | 39.35 | 100.12 |  |  |  |  | 3.120 | 0.100 | 15.090 | 0.300 |  |  |  |  |  |  |  |  |  |  |  |  | 10.03 | 3.43 | 22.90 | 0.50 | 9.00 | 0.10 | 18.32 | 0.200 | 0.210 | 0.000 | 2.420 | 0.100 |  |  |  |  |
| 433 | 67 | Ecological restoration | Grassland | 0-10 | 39.35 | 100.12 |  |  |  |  | 3.120 | 0.100 | 18.030 | 0.300 |  |  |  |  |  |  |  |  |  |  |  |  | 10.03 | 3.43 | 31.13 | 0.50 | 9.00 | 0.10 | 21.00 | 0.110 | 0.210 | 0.000 | 3.180 | 0.100 |  |  |  |  |
| 434 | 67 | Ecological restoration | Grassland | 0-10 | 39.35 | 100.12 | 0.60 | 0.03 | 0.620 | #### | 16.310 | 0.970 | 16.790 | 0.980 |  |  |  |  | 62.06 | 5.29 | ##### | 20.17 | ##### | 69.310 | ##### | 71.67 | 12.69 | 0.42 | 168.1 | 5.99 | 8.50 | 0.43 | 13.67 | 0.760 | 1.330 | 0.020 | 0.970 | 0.010 |  |  |  |  |
| 435 | 68 | Ecological restoration | Grassland | 0-10 | 42.34 | 126.38 | 0.60 | 0.03 | 0.590 | 0.010 | 16.310 | 0.970 | 17.380 | 0.762 |  |  |  |  | 62.06 | 5.29 | 619.55 | 35.91 | ##### | 69.310 | ##### | 128.53 | 12.69 | 0.42 | 324.3 | 6.21 | 8.50 | 0.43 | 18.67 | 0.670 | 1.330 | 0.020 | 1.130 | 0.010 |  |  |  |  |
| 436 | 68 | Ecological restoration | Grassland | 0-10 | 42.34 | 126.38 | 0.60 | 0.03 | 0.890 | #### | 16.310 | 0.970 | 22.940 | 0.683 |  |  |  |  | 62.06 | 5.29 | ##### | 9.07 | ##### | 69.310 | ##### | 99.70 | 12.69 | 0.42 | ##### | 5.96 | 8.50 | 0.43 | 9.33 | 0.560 | 1.330 | 0.020 | 0.760 | 0.010 |  |  |  |  |
| 437 | 68 | Ecological restoration | Grassland | 0-10 | 42.34 | 126.38 | 0.60 | 0.03 | 0.650 | #### | 16.310 | 0.970 | 23.150 | 0.873 |  |  |  |  | 62.06 | 5.29 | ##### | 12.55 | ##### | 69.310 | ##### | 79.38 | 12.69 | 0.42 | 447.5 | 6.62 | 8.50 | 0.43 | 9.00 | 0.450 | 1.330 | 0.020 | 0.740 | 0.010 |  |  |  |  |
| 438 | 68 | Ecological restoration | Grassland | 10-20 | 42.34 | 126.38 | 0.100 | 0.000 | 0.050 | 0.100 |  |  |  |  | 0.42 | 0.320 | 0.43 | 0.200 | 162.38 | 6.57 | ##### | 14.25 |  |  |  |  |  |  |  |  | 3.00 | 0.19 | 4.01 | 0.250 | 2.650 | 0.080 | 3.470 | 0.030 |  |  |  |  |
| 439 | 68 | Ecological restoration | Grassland | 10-20 | 42.34 | 126.38 | 0.100 | 0.000 | 0.080 | 0.110 |  |  |  |  | 0.42 | 0.320 | 0.53 | 0.100 | 162.38 | 6.57 | ##### | 27.34 |  |  |  |  |  |  |  |  | 3.00 | 0.19 | 3.12 | 0.060 | 2.650 | 0.080 | 3.060 | 0.220 |  |  |  |  |
| 440 | 68 | Ecological restoration | Grassland | 10-20 | 42.34 | 126.38 | 0.100 | 0.000 | 0.120 | 0.120 |  |  |  |  | 0.42 | 0.320 | 0.60 | 0.051 | 162.38 | 6.57 | 301.68 | 20.56 |  |  |  |  |  |  |  |  | 3.00 | 0.19 | 2.84 | 0.250 | 2.650 | 0.080 | 3.270 | 0.190 |  |  |  |  |
| 441 | 68 | Ecological restoration | Grassland | ＞20 | 42.34 | 126.38 | 0.100 | 0.000 | 0.180 | #### |  |  |  |  | 0.42 | 0.320 | 0.782 | 0.152 | 162.38 | 6.57 | ##### | 23.06 |  |  |  |  |  |  |  |  | 3.00 | 0.19 | 3.45 | 0.200 | 2.650 | 0.080 | 3.720 | 0.020 |  |  |  |  |
| 442 | 68 | Ecological restoration | Grassland | ＞20 | 42.34 | 126.38 | 0.540 | 0.000 | 0.570 | 0.010 | 1.680 | 0.110 | 1.990 | 0.070 |  |  |  |  | 50.38 | 3.68 | ##### | 27.50 | 23.67 | 3.670 | 38.00 | 3.70 |  |  |  |  | 5.00 | 1.00 | 4.00 | 2.000 | 1.590 | 0.110 | 2.020 | 0.150 |  |  |  |  |
| 443 | 68 | Ecological restoration | Grassland | ＞20 | 42.34 | 126.38 | 0.540 | 0.000 | 0.560 | #### | 1.680 | 0.110 | 1.780 | 0.010 |  |  |  |  | 50.38 | 3.68 | 43.10 | 4.30 | 23.67 | 3.670 | 17.30 | 3.80 |  |  |  |  | 5.00 | 1.00 | 9.00 | 1.000 | 1.590 | 0.110 | 2.360 | 0.100 |  |  |  |  |
| 444 | 69 | Ecological restoration | Grassland | 10-20 | 34.60 | 92.58 | 0.540 | 0.000 | 0.540 | 0.010 | 1.680 | 0.110 | 1.160 | 0.120 |  |  |  |  | 50.38 | 3.68 | 88.50 | 17.80 | 23.67 | 3.670 | 83.60 | 11.50 |  |  |  |  | 5.00 | 1.00 | 19.00 | 2.000 | 1.590 | 0.110 | 2.510 | 0.080 |  |  |  |  |
| 445 | 69 | Ecological restoration | Grassland | 10-20 | 34.60 | 92.58 | 0.540 | 0.000 | 0.550 | 0.010 | 1.680 | 0.110 | 0.730 | 0.020 |  |  |  |  | 50.38 | 3.68 | 126.70 | 10.40 | 23.67 | 3.670 | 188.30 | 29.80 |  |  |  |  | 5.00 | 1.00 | 16.00 | 2.000 | 1.590 | 0.110 | 2.420 | 0.140 |  |  |  |  |
| 446 | 69 | Ecological restoration | Grassland | 10-20 | 34.60 | 92.58 | 0.540 | 0.000 | 0.540 | #### | 1.680 | 0.110 | 0.590 | 0.030 |  |  |  |  | 50.38 | 3.68 | ##### | 50.90 | 23.67 | 3.670 | ##### | 130.50 |  |  |  |  | 5.00 | 1.00 | 11.00 | 2.000 | 1.590 | 0.110 | 2.280 | 0.100 |  |  |  |  |
| 447 | 69 | Ecological restoration | Grassland | 10-20 | 34.60 | 92.58 |  |  |  |  | ##### | 0.560 | 16.010 | 3.560 |  |  |  |  |  |  |  |  |  |  |  |  |  |  |  |  |  |  |  |  |  |  |  |  |  |  |  |  |
| 448 | 69 | Ecological restoration | Grassland | 10-20 | 34.60 | 92.58 |  |  |  |  | ##### | 0.560 | 49.780 | 2.480 |  |  |  |  |  |  |  |  |  |  |  |  |  |  |  |  |  |  |  |  |  |  |  |  |  |  |  |  |
| 449 | 69 | Ecological restoration | Grassland | 10-20 | 34.60 | 92.58 |  |  |  |  | ##### | 0.560 | 43.110 | 3.450 |  |  |  |  |  |  |  |  |  |  |  |  |  |  |  |  |  |  |  |  |  |  |  |  |  |  |  |  |
| 450 | 69 | Ecological restoration | Grassland | 10-20 | 34.60 | 92.58 | 0.840 | 0.770 | 0.880 | 0.100 | 82.130 | 5.250 | 77.300 | 9.380 |  |  |  |  |  |  |  |  |  |  |  |  |  |  |  |  |  |  |  |  | 1.166 | 0.155 | 1.061 | 0.105 | 0.346 | 0.086 | 0.429 | 0.046 |
| 451 | 69 | Ecological restoration | Grassland | 10-20 | 34.60 | 92.58 | 0.770 | 0.070 | 0.800 | 0.130 | ##### | 7.390 | 69.380 | 7.660 |  |  |  |  |  |  |  |  |  |  |  |  |  |  |  |  |  |  |  |  | 0.975 | 0.155 | 0.899 | 0.105 | 0.53 | 0.086 | 0.470 | 0.046 |
| 452 | 69 | Ecological restoration | Grassland | 10-20 | 34.60 | 92.58 | 0.720 | 0.040 | 0.700 | #### | ##### | 5.110 | 65.000 | 2.990 |  |  |  |  |  |  |  |  |  |  |  |  |  |  |  |  |  |  |  |  | 0.757 | 0.155 | 0.722 | 0.105 | 0.60 | 0.086 | 0.586 | 0.046 |
| 453 | 69 | Ecological restoration | Grassland | 10-20 | 34.60 | 92.58 | 0.840 | 0.770 | 0.830 | #### | 82.130 | 5.250 | 74.780 | 10.190 |  |  |  |  |  |  |  |  |  |  |  |  |  |  |  |  |  |  |  |  | 1.166 | 0.155 | 1.056 | 0.104 | 0.346 | 0.086 | 0.374 | 0.070 |
| 457 | 71 | Ecological restoration | Grassland | 0-10 | 34.78 | 100.35 | 0.770 | 0.070 | 0.840 | #### | ##### | 7.390 | 83.870 | 9.820 |  |  |  |  |  |  |  |  |  |  |  |  |  |  |  |  |  |  |  |  | 0.975 | 0.155 | 0.962 | 0.104 | 0.53 | 0.086 | 0.454 | 0.070 |
| 458 | 71 | Ecological restoration | Grassland | 10-20 | 34.78 | 100.35 | 0.720 | 0.040 | 0.680 | 0.140 | ##### | 5.110 | 70.180 | 7.360 |  |  |  |  |  |  |  |  |  |  |  |  |  |  |  |  |  |  |  |  | 0.757 | 0.155 | 0.753 | 0.104 | 0.60 | 0.086 | 0.613 | 0.070 |
| 459 | 71 | Ecological restoration | Grassland | 10-20 | 34.78 | 100.35 | 0.010 | 0.001 | 0.550 | #### | 0.010 | 0.001 | 0.020 | 0.002 |  |  |  |  | 133.15 | 48.68 | ##### | 42.07 | 83.24 | ##### | ##### | 46.40 | 60.80 | 21.92 | 57.66 | 11.74 |  |  |  |  |  |  |  |  |  |  |  |  |
| 460 | 71 | Ecological restoration | Grassland | 10-20 | 34.78 | 100.35 | 0.010 | 0.001 | 0.790 | #### | 0.010 | 0.001 | 0.020 | 0.002 |  |  |  |  | 133.15 | 48.68 | 182.88 | 40.11 | 83.24 | ##### | 211.59 | 46.40 | 60.80 | 21.92 | 61.52 | 13.49 |  |  |  |  |  |  |  |  |  |  |  |  |
| 32 | 5 | Ecological rehabilitation | Forest | 0-10 | 36.76 | 109.31 | 0.010 | 0.001 | 0.730 | #### | 0.010 | 0.001 | 0.020 | 0.002 |  |  |  |  | 133.15 | 48.68 | ##### | 49.92 | 83.24 | ##### | ##### | 38.29 | 60.80 | 21.92 | 63.73 | 11.78 |  |  |  |  |  |  |  |  |  |  |  |  |
| 33 | 5 | Ecological rehabilitation | Forest | 10-20 | 36.76 | 109.31 | 0.070 | 0.020 | 0.070 | 0.010 | 1.230 | 0.920 | 1.730 | 0.580 |  |  |  |  |  |  |  |  |  |  |  |  |  |  |  |  |  |  |  |  |  |  |  |  |  |  |  |  |
| 34 | 5 | Ecological rehabilitation | Forest | ＞20 | 36.76 | 109.31 | 0.090 | 0.010 | 0.110 | #### | 0.160 | 0.090 | 0.200 | 0.020 |  |  |  |  |  |  |  |  |  |  |  |  |  |  |  |  |  |  |  |  |  |  |  |  |  |  |  |  |
| 35 | 5 | Ecological rehabilitation | Forest | ＞20 | 36.76 | 109.31 | 0.120 | 0.020 | 0.210 | #### | 2.300 | 0.410 | 7.960 | 0.210 |  |  |  |  |  |  |  |  |  |  |  |  |  |  |  |  |  |  |  |  |  |  |  |  |  |  |  |  |
| 36 | 5 | Ecological rehabilitation | Forest | 0-10 | 36.76 | 109.31 | 0.120 | 0.020 | 0.140 | 0.010 | 2.300 | 0.410 | 3.070 | 0.160 |  |  |  |  |  |  |  |  |  |  |  |  |  |  |  |  |  |  |  |  |  |  |  |  |  |  |  |  |
| 37 | 5 | Ecological rehabilitation | Forest | 10-20 | 36.76 | 109.31 | 0.120 | 0.020 | 0.180 | #### | 2.300 | 0.410 | 2.980 | 0.310 |  |  |  |  |  |  |  |  |  |  |  |  |  |  |  |  |  |  |  |  |  |  |  |  |  |  |  |  |
| 38 | 5 | Ecological rehabilitation | Forest | ＞20 | 36.76 | 109.31 | 0.120 | 0.020 | 0.270 | #### | 2.300 | 0.410 | 6.700 | 0.670 |  |  |  |  |  |  |  |  |  |  |  |  |  |  |  |  |  |  |  |  |  |  |  |  |  |  |  |  |
| 39 | 5 | Ecological rehabilitation | Forest | ＞20 | 36.76 | 109.31 | 0.120 | 0.020 | 0.260 | #### | 2.300 | 0.410 | 13.200 | 1.800 |  |  |  |  |  |  |  |  |  |  |  |  |  |  |  |  |  |  |  |  |  |  |  |  |  |  |  |  |
| 40 | 5 | Ecological rehabilitation | Forest | 0-10 | 36.76 | 109.31 |  |  |  |  | ##### | 5.080 | 44.390 | 2.540 |  |  |  |  |  |  |  |  |  |  |  |  |  |  |  |  |  |  |  |  |  |  |  |  |  |  |  |  |
| 41 | 5 | Ecological rehabilitation | Forest | 10-20 | 36.76 | 109.31 |  |  |  |  | ##### | 5.080 | 47.000 | 3.230 |  |  |  |  |  |  |  |  |  |  |  |  |  |  |  |  |  |  |  |  |  |  |  |  |  |  |  |  |
| 42 | 5 | Ecological rehabilitation | Forest | ＞20 | 36.76 | 109.31 |  |  |  |  | ##### | 5.080 | 66.530 | #### |  |  |  |  |  |  |  |  |  |  |  |  |  |  |  |  |  |  |  |  |  |  |  |  |  |  |  |  |
| 43 | 5 | Ecological rehabilitation | Forest | ＞20 | 36.76 | 109.31 | 0.240 | 0.024 | 0.250 | #### | 1.880 | 0.188 | 2.920 | 0.292 | 0.024 | 0.002 | 0.045 | 0.005 |  |  |  |  |  |  |  |  |  |  |  |  |  |  |  |  | 1.440 | 0.144 | 1.690 | 0.169 |  |  |  |  |
| 53 | 7 | Ecological rehabilitation | Grassland | 0-10 | 40.44 | 116.00 | 0.240 | 0.024 | 0.320 | #### | 1.880 | 0.188 | 2.830 | 0.283 | 0.024 | 0.002 | 0.068 | 0.007 |  |  |  |  |  |  |  |  |  |  |  |  |  |  |  |  | 1.440 | 0.144 | 1.680 | 0.168 |  |  |  |  |
| 54 | 7 | Ecological rehabilitation | Grassland | 0-10 | 40.44 | 116.00 | 0.240 | 0.024 | 0.350 | #### | 1.880 | 0.188 | 5.000 | 0.500 | 0.024 | 0.002 | 0.139 | 0.014 |  |  |  |  |  |  |  |  |  |  |  |  |  |  |  |  | 1.440 | 0.144 | 1.650 | 0.165 |  |  |  |  |
| 75 | 10 | Ecological rehabilitation | Grassland | 0-10 | 35.98 | 106.43 | 0.240 | 0.024 | 0.280 | #### | 1.880 | 0.188 | 2.880 | 0.288 | 0.024 | 0.002 | 0.05 | 0.005 |  |  |  |  |  |  |  |  |  |  |  |  |  |  |  |  | 1.440 | 0.144 | 1.550 | 0.155 |  |  |  |  |
| 76 | 10 | Ecological rehabilitation | Grassland | 0-10 | 35.98 | 106.43 | 0.240 | 0.024 | 0.350 | #### | 1.880 | 0.188 | 4.170 | 0.417 | 0.024 | 0.002 | 0.072 | 0.007 |  |  |  |  |  |  |  |  |  |  |  |  |  |  |  |  | 1.440 | 0.144 | 1.520 | 0.152 |  |  |  |  |
| 77 | 10 | Ecological rehabilitation | Grassland | 0-10 | 35.98 | 106.43 | 0.240 | 0.024 | 0.390 | #### | 1.880 | 0.188 | 5.470 | 0.547 | 0.024 | 0.002 | 0.113 | 0.011 |  |  |  |  |  |  |  |  |  |  |  |  |  |  |  |  | 1.440 | 0.144 | 1.480 | 0.148 |  |  |  |  |
| 78 | 10 | Ecological rehabilitation | Grassland | 10-20 | 35.98 | 106.43 | 0.240 | 0.024 | 0.360 | #### | 1.880 | 0.188 | 2.880 | 0.288 | 0.024 | 0.002 | 0.101 | 0.010 |  |  |  |  |  |  |  |  |  |  |  |  |  |  |  |  | 1.440 | 0.144 | 1.740 | 0.174 |  |  |  |  |
| 79 | 10 | Ecological rehabilitation | Grassland | 10-20 | 35.98 | 106.43 | 0.240 | 0.024 | 0.410 | 0.041 | 1.880 | 0.188 | 4.950 | 0.495 | 0.024 | 0.002 | 0.13 | 0.013 |  |  |  |  |  |  |  |  |  |  |  |  |  |  |  |  | 1.440 | 0.144 | 1.590 | 0.159 |  |  |  |  |
| 80 | 10 | Ecological rehabilitation | Grassland | 10-20 | 35.98 | 106.43 | 0.240 | 0.024 | 0.500 | #### | 1.880 | 0.188 | 6.570 | 0.657 | 0.024 | 0.002 | 0.192 | 0.019 |  |  |  |  |  |  |  |  |  |  |  |  |  |  |  |  | 1.440 | 0.144 | 1.250 | 0.125 |  |  |  |  |
| 81 | 10 | Ecological rehabilitation | Grassland | ＞20 | 35.98 | 106.43 | 0.120 | 0.005 | 0.220 | 0.012 | 1.260 | 0.080 | 1.940 | 0.140 |  |  |  |  |  |  |  |  |  |  |  |  |  |  |  |  |  |  |  |  | 0.680 | 0.068 | 0.770 | 0.077 | 0.39 | 0.039 | 0.430 | 0.043 |
| 82 | 10 | Ecological rehabilitation | Grassland | ＞20 | 35.98 | 106.43 |  |  |  |  | ##### | 1.620 | 24.610 | 2.110 |  |  |  |  |  |  |  |  |  |  |  |  |  |  |  |  |  |  |  |  |  |  |  |  |  |  |  |  |
| 83 | 10 | Ecological rehabilitation | Grassland | ＞20 | 35.98 | 106.43 |  |  |  |  | ##### | 1.620 | 23.080 | 1.800 |  |  |  |  |  |  |  |  |  |  |  |  |  |  |  |  |  |  |  |  |  |  |  |  |  |  |  |  |
| 88 | 12 | Ecological rehabilitation | Grassland | 0-10 | 34.31 | 101.37 |  |  |  |  | ##### | 1.620 | 23.480 | 2.370 |  |  |  |  |  |  |  |  |  |  |  |  |  |  |  |  |  |  |  |  |  |  |  |  |  |  |  |  |
| 89 | 12 | Ecological rehabilitation | Grassland | 0-10 | 34.31 | 101.37 |  |  |  |  | ##### | 1.620 | 15.710 | 1.270 |  |  |  |  |  |  |  |  |  |  |  |  |  |  |  |  |  |  |  |  |  |  |  |  |  |  |  |  |
| 90 | 12 | Ecological rehabilitation | Grassland | 0-10 | 34.31 | 101.37 |  |  |  |  | ##### | 1.620 | 34.820 | 2.550 |  |  |  |  |  |  |  |  |  |  |  |  |  |  |  |  |  |  |  |  |  |  |  |  |  |  |  |  |
| 95 | 15 | Ecological rehabilitation | Forest | 0-10 | 38.43 | 114.09 |  |  |  |  | ##### | 1.620 | 24.660 | 2.470 |  |  |  |  |  |  |  |  |  |  |  |  |  |  |  |  |  |  |  |  |  |  |  |  |  |  |  |  |
| 96 | 15 | Ecological rehabilitation | Forest | 0-10 | 38.43 | 114.09 | 0.523 | 0.052 | 0.501 | #### | ##### | 2.230 | 14.010 | 1.401 | 14.35 | 1.435 | 23.69 | 2.369 |  |  |  |  |  |  |  |  |  |  |  |  |  |  |  |  |  |  |  |  |  |  |  |  |
| 97 | 15 | Ecological rehabilitation | Forest | 0-10 | 38.43 | 114.09 | 0.523 | 0.052 | 0.587 | #### | ##### | 2.230 | 16.070 | 1.607 | 14.35 | 1.435 | 19.21 | 1.921 |  |  |  |  |  |  |  |  |  |  |  |  |  |  |  |  |  |  |  |  |  |  |  |  |
| 98 | 15 | Ecological rehabilitation | Forest | 0-10 | 38.43 | 114.09 | 0.523 | 0.052 | 0.663 | #### | ##### | 2.230 | 21.650 | 2.165 | 14.35 | 1.435 | 18.43 | 1.843 |  |  |  |  |  |  |  |  |  |  |  |  |  |  |  |  |  |  |  |  |  |  |  |  |
| 99 | 15 | Ecological rehabilitation | Forest | 0-10 | 38.43 | 114.09 | 0.320 | 0.020 | 0.310 | #### |  |  |  |  | 19.23 | 2.230 | 18.78 | 3.450 |  |  |  |  |  |  |  |  |  |  |  |  |  |  |  |  |  |  |  |  |  |  |  |  |
| 100 | 16 | Ecological rehabilitation | Forest | 0-10 | 26.80 | 106.75 | 0.320 | 0.020 | 0.290 | #### |  |  |  |  | 19.23 | 2.230 | 18.15 | 2.060 |  |  |  |  |  |  |  |  |  |  |  |  |  |  |  |  |  |  |  |  |  |  |  |  |
| 101 | 16 | Ecological rehabilitation | Forest | 0-10 | 26.80 | 106.75 | 0.320 | 0.020 | 0.280 | #### |  |  |  |  | 19.23 | 2.230 | 17.89 | 3.790 |  |  |  |  |  |  |  |  |  |  |  |  |  |  |  |  |  |  |  |  |  |  |  |  |
| 102 | 16 | Ecological rehabilitation | Forest | 0-10 | 26.80 | 106.75 | 0.320 | 0.020 | 0.240 | 0.010 |  |  |  |  | 19.23 | 2.230 | 17.56 | 2.130 |  |  |  |  |  |  |  |  |  |  |  |  |  |  |  |  |  |  |  |  |  |  |  |  |
| 103 | 16 | Ecological rehabilitation | Forest | 0-10 | 26.80 | 106.75 | 0.320 | 0.020 | 0.210 | #### |  |  |  |  | 19.23 | 2.230 | 17.34 | 2.440 |  |  |  |  |  |  |  |  |  |  |  |  |  |  |  |  |  |  |  |  |  |  |  |  |
| 104 | 16 | Ecological rehabilitation | Forest | 0-10 | 26.80 | 106.75 |  |  |  |  | 6.230 | 0.623 | 6.421 | 0.642 |  |  |  |  | ##### | 23.55 | ##### | 62.35 | ##### | ##### | ##### | 174.64 |  |  |  |  | 10.00 | 1.00 | 17.56 | 1.756 | 2.130 | 0.213 | 2.540 | 0.254 |  |  |  |  |
| 105 | 16 | Ecological rehabilitation | Forest | 0-10 | 26.80 | 106.75 |  |  |  |  | 5.310 | 0.531 | 5.321 | 0.532 |  |  |  |  | ##### | 23.55 | ##### | 63.25 | ##### | ##### | ##### | 170.53 |  |  |  |  | 10.00 | 1.00 | 16.32 | 1.632 | 3.130 | 0.313 | 2.340 | 0.234 |  |  |  |  |
| 106 | 16 | Ecological rehabilitation | Forest | 0-10 | 26.80 | 106.75 |  |  |  |  | 3.190 | 0.319 | 4.250 | 0.425 |  |  |  |  | ##### | 23.55 | ##### | 62.74 | ##### | ##### | ##### | 172.43 |  |  |  |  | 10.00 | 1.00 | 15.20 | 1.520 | 4.130 | 0.413 | 2.560 | 0.256 |  |  |  |  |
| 107 | 16 | Ecological rehabilitation | Forest | 0-10 | 26.80 | 106.75 |  |  |  |  | 1.440 | 0.144 | 2.330 | 0.233 |  |  |  |  | ##### | 23.55 | 631.28 | 63.13 | ##### | ##### | ##### | 178.63 |  |  |  |  | 10.00 | 1.00 | 14.98 | 1.498 | 5.130 | 0.513 | 2.410 | 0.241 |  |  |  |  |
| 108 | 16 | Ecological rehabilitation | Forest | 0-10 | 26.80 | 106.75 |  |  |  |  | 6.230 | 0.623 | 7.430 | 0.743 |  |  |  |  | ##### | 23.55 | ##### | 145.23 | ##### | ##### | ##### | 185.23 |  |  |  |  | 10.00 | 1.00 | 22.31 | 2.231 | 6.130 | 0.613 | 2.890 | 0.289 |  |  |  |  |
| 109 | 16 | Ecological rehabilitation | Forest | 0-10 | 26.80 | 106.75 |  |  |  |  | 5.310 | 0.531 | 5.440 | 0.544 |  |  |  |  | ##### | 23.55 | ##### | 146.33 | ##### | ##### | ##### | 180.02 |  |  |  |  | 10.00 | 1.00 | 20.57 | 2.057 | 7.130 | 0.713 | 2.960 | 0.296 |  |  |  |  |
| 110 | 16 | Ecological rehabilitation | Forest | 0-10 | 26.80 | 106.75 |  |  |  |  | 3.190 | 0.319 | 4.080 | 0.408 |  |  |  |  | ##### | 23.55 | ##### | 130.87 | ##### | ##### | ##### | 187.54 |  |  |  |  | 10.00 | 1.00 | 21.34 | 2.134 | 8.130 | 0.813 | 3.010 | 0.301 |  |  |  |  |
| 111 | 16 | Ecological rehabilitation | Forest | 0-10 | 26.80 | 106.75 |  |  |  |  | 1.440 | 0.144 | 1.040 | 0.104 |  |  |  |  | ##### | 23.55 | ##### | 123.45 | ##### | ##### | ##### | 184.64 |  |  |  |  | 10.00 | 1.00 | 20.96 | 2.096 | 9.130 | 0.913 | 3.060 | 0.306 |  |  |  |  |
| 116 | 18 | Ecological rehabilitation | Forest | 0-10 | 20.25 | 109.66 |  |  |  |  | 6.230 | 0.623 | 7.240 | 0.724 |  |  |  |  | ##### | 23.55 | ##### | 123.56 | ##### | ##### | ##### | ##### |  |  |  |  | 10.00 | 1.00 | 23.47 | 2.347 | 10.130 | 1.013 | 2.210 | 0.221 |  |  |  |  |
| 117 | 18 | Ecological rehabilitation | Forest | 0-10 | 20.25 | 109.66 |  |  |  |  | 5.310 | 0.531 | 5.320 | 0.532 |  |  |  |  | ##### | 23.55 | ##### | 120.05 | ##### | ##### | ##### | 301.57 |  |  |  |  | 10.00 | 1.00 | 22.10 | 2.210 | 11.130 | 1.113 | 2.160 | 0.216 |  |  |  |  |
| 118 | 18 | Ecological rehabilitation | Forest | 0-10 | 20.25 | 109.66 |  |  |  |  | 3.190 | 0.319 | 4.010 | 0.401 |  |  |  |  | ##### | 23.55 | ##### | 120.44 | ##### | ##### | ##### | ##### |  |  |  |  | 10.00 | 1.00 | 23.54 | 2.354 | 12.130 | 1.213 | 2.340 | 0.234 |  |  |  |  |
| 119 | 18 | Ecological rehabilitation | Forest | 0-10 | 20.25 | 109.66 |  |  |  |  | 1.440 | 0.144 | 2.270 | 0.227 |  |  |  |  | ##### | 23.55 | ##### | 112.57 | ##### | ##### | ##### | 301.62 |  |  |  |  | 10.00 | 1.00 | 20.37 | 2.037 | 13.130 | 1.313 | 2.330 | 0.233 |  |  |  |  |
| 120 | 18 | Ecological rehabilitation | Forest | 0-10 | 20.25 | 109.66 |  |  |  |  | 6.230 | 0.623 | 7.060 | 0.706 |  |  |  |  | ##### | 23.55 | ##### | 112.44 | ##### | ##### | ##### | ##### |  |  |  |  | 10.00 | 1.00 | 21.07 | 2.107 | 14.130 | 1.413 | 2.450 | 0.245 |  |  |  |  |
| 121 | 18 | Ecological rehabilitation | Forest | 0-10 | 20.25 | 109.66 |  |  |  |  | 5.310 | 0.531 | 6.130 | 0.613 |  |  |  |  | ##### | 23.55 | ##### | 110.24 | ##### | ##### | ##### | ##### |  |  |  |  | 10.00 | 1.00 | 22.31 | 2.231 | 15.130 | 1.513 | 2.340 | 0.234 |  |  |  |  |
| 122 | 18 | Ecological rehabilitation | Forest | 0-10 | 20.25 | 109.66 |  |  |  |  | 3.190 | 0.319 | 5.090 | 0.509 |  |  |  |  | ##### | 23.55 | ##### | 102.87 | ##### | ##### | ##### | ##### |  |  |  |  | 10.00 | 1.00 | 22.64 | 2.264 | 16.130 | 1.613 | 2.360 | 0.236 |  |  |  |  |
| 123 | 18 | Ecological rehabilitation | Forest | 0-10 | 20.25 | 109.66 |  |  |  |  | 1.440 | 0.144 | 3.160 | 0.316 |  |  |  |  | ##### | 23.55 | ##### | 115.84 | ##### | ##### | ##### | 283.10 |  |  |  |  | 10.00 | 1.00 | 22.30 | 2.230 | 17.130 | 1.713 | 2.310 | 0.231 |  |  |  |  |
| 124 | 19 | Ecological rehabilitation | Grassland | 0-10 | 37.78 | 107.36 |  |  |  |  | 6.230 | 0.623 | 7.330 | 0.733 |  |  |  |  | ##### | 23.55 | ##### | 112.44 | ##### | ##### | ##### | ##### |  |  |  |  | 10.00 | 1.00 | 20.01 | 2.001 | 18.130 | 1.813 | 2.200 | 0.220 |  |  |  |  |
| 125 | 19 | Ecological rehabilitation | Grassland | 0-10 | 37.78 | 107.36 |  |  |  |  | 5.310 | 0.531 | 6.030 | 0.603 |  |  |  |  | ##### | 23.55 | ##### | 113.70 | ##### | ##### | ##### | ##### |  |  |  |  | 10.00 | 1.00 | 20.07 | 2.007 | 19.130 | 1.913 | 2.210 | 0.221 |  |  |  |  |
| 126 | 19 | Ecological rehabilitation | Grassland | 0-10 | 37.78 | 107.36 |  |  |  |  | 3.190 | 0.319 | 5.960 | 0.596 |  |  |  |  | ##### | 23.55 | ##### | 113.32 | ##### | ##### | ##### | ##### |  |  |  |  | 10.00 | 1.00 | 22.30 | 2.230 | 20.130 | 2.013 | 2.230 | 0.223 |  |  |  |  |
| 127 | 19 | Ecological rehabilitation | Grassland | 0-10 | 37.78 | 107.36 |  |  |  |  | 1.440 | 0.144 | 5.080 | 0.508 |  |  |  |  | ##### | 23.55 | ##### | 115.83 | ##### | ##### | ##### | ##### |  |  |  |  | 10.00 | 1.00 | 21.46 | 2.146 | 21.130 | 2.113 | 2.140 | 0.214 |  |  |  |  |
| 128 | 19 | Ecological rehabilitation | Grassland | 0-10 | 37.78 | 107.36 |  |  |  |  | 6.230 | 0.623 | 8.130 | 0.813 |  |  |  |  | ##### | 23.55 | ##### | 120.04 | ##### | ##### | ##### | ##### |  |  |  |  | 10.00 | 1.00 | 22.31 | 2.231 | 22.130 | 2.213 | 2.650 | 0.265 |  |  |  |  |
| 129 | 19 | Ecological rehabilitation | Grassland | 0-10 | 37.78 | 107.36 |  |  |  |  | 5.310 | 0.531 | 6.240 | 0.624 |  |  |  |  | ##### | 23.55 | ##### | 120.64 | ##### | ##### | ##### | ##### |  |  |  |  | 10.00 | 1.00 | 24.17 | 2.417 | 23.130 | 2.313 | 2.640 | 0.264 |  |  |  |  |
| 130 | 19 | Ecological rehabilitation | Grassland | 0-10 | 37.78 | 107.36 |  |  |  |  | 3.190 | 0.319 | 6.070 | 0.607 |  |  |  |  | ##### | 23.55 | ##### | 125.44 | ##### | ##### | ##### | ##### |  |  |  |  | 10.00 | 1.00 | 23.10 | 2.310 | 24.130 | 2.413 | 2.740 | 0.274 |  |  |  |  |
| 131 | 19 | Ecological rehabilitation | Grassland | 0-10 | 37.78 | 107.36 |  |  |  |  | 1.440 | 0.144 | 5.310 | 0.531 |  |  |  |  | ##### | 23.55 | ##### | 121.11 | ##### | ##### | ##### | 237.81 |  |  |  |  | 10.00 | 1.00 | 22.96 | 2.296 | 25.130 | 2.513 | 2.590 | 0.259 |  |  |  |  |
| 132 | 19 | Ecological rehabilitation | Grassland | 0-10 | 37.78 | 107.36 | 0.820 | 0.020 | 0.820 | #### | 30.510 | 2.210 | 24.840 | 2.380 |  |  |  |  |  |  |  |  |  |  |  |  |  |  |  |  |  |  |  |  |  |  |  |  |  |  |  |  |
| 133 | 19 | Ecological rehabilitation | Grassland | 0-10 | 37.78 | 107.36 | 0.820 | 0.020 | 0.840 | #### | 30.510 | 2.210 | 23.940 | 2.840 |  |  |  |  |  |  |  |  |  |  |  |  |  |  |  |  |  |  |  |  |  |  |  |  |  |  |  |  |
| 134 | 20 | Ecological rehabilitation | Forest | 0-10 | 37.46 | 122.10 | 0.820 | 0.020 | 0.860 | #### | 30.510 | 2.210 | 22.550 | 1.300 |  |  |  |  |  |  |  |  |  |  |  |  |  |  |  |  |  |  |  |  |  |  |  |  |  |  |  |  |
| 135 | 20 | Ecological rehabilitation | Forest | 0-10 | 37.46 | 122.10 | 0.820 | 0.020 | 0.750 | #### | 30.510 | 2.210 | 15.750 | 3.970 |  |  |  |  |  |  |  |  |  |  |  |  |  |  |  |  |  |  |  |  |  |  |  |  |  |  |  |  |
| 136 | 20 | Ecological rehabilitation | Forest | 0-10 | 37.46 | 122.10 |  |  |  |  | 13.100 | 0.700 | 15.500 | 1.500 |  |  |  |  | ##### | 29.87 | 371.45 | 37.15 | 48.67 | 4.867 | 57.38 | 5.74 |  |  |  |  |  |  |  |  |  |  |  |  |  |  |  |  |
| 137 | 20 | Ecological rehabilitation | Forest | 0-10 | 37.46 | 122.10 |  |  |  |  | 10.800 | 1.900 | 14.100 | 1.300 |  |  |  |  | ##### | 29.87 | 371.45 | 37.15 | 48.67 | 4.867 | 57.38 | 5.74 |  |  |  |  |  |  |  |  |  |  |  |  |  |  |  |  |
| 138 | 20 | Ecological rehabilitation | Forest | 0-10 | 37.46 | 122.10 |  |  |  |  | 11.900 | 1.800 | 14.800 | 1.400 |  |  |  |  | ##### | 29.87 | 371.45 | 37.15 | 48.67 | 4.867 | 57.38 | 5.74 |  |  |  |  |  |  |  |  |  |  |  |  |  |  |  |  |
| 139 | 20 | Ecological rehabilitation | Forest | 0-10 | 37.46 | 122.10 | 0.820 | 0.082 | 0.620 | #### | 9.220 | 0.922 | 2.160 | 0.216 |  |  |  |  |  |  |  |  |  |  |  |  |  |  |  |  | ##### | ##### | ##### | ##### | 6.420 | 0.642 | 6.330 | 0.633 |  |  |  |  |
| 140 | 20 | Ecological rehabilitation | Forest | 0-10 | 37.46 | 122.10 | 0.681 | 0.068 | 0.610 | 0.061 | 2.670 | 0.267 | 2.240 | 0.224 |  |  |  |  |  |  |  |  |  |  |  |  |  |  |  |  | ##### | 221.31 | ##### | ##### | 6.290 | 0.629 | 5.310 | 0.531 |  |  |  |  |
| 141 | 20 | Ecological rehabilitation | Forest | 0-10 | 37.46 | 122.10 | 0.610 | 0.061 | 0.590 | #### | 2.650 | 0.265 | 2.340 | 0.234 |  |  |  |  |  |  |  |  |  |  |  |  |  |  |  |  | ##### | 174.24 | ##### | ##### | 6.210 | 0.621 | 5.010 | 0.501 |  |  |  |  |
| 183 | 27 | Ecological rehabilitation | Forest | 10-20 | 36.86 | 109.33 | 0.600 | 0.060 | 0.580 | #### | 2.490 | 0.249 | 1.690 | 0.169 |  |  |  |  |  |  |  |  |  |  |  |  |  |  |  |  | ##### | 153.20 | ##### | ##### | 5.960 | 0.596 | 5.230 | 0.523 |  |  |  |  |
| 184 | 27 | Ecological rehabilitation | Forest | ＞20 | 36.86 | 109.33 | 0.605 | 0.061 | 0.631 | #### | 1.590 | 0.159 | 1.870 | 0.187 |  |  |  |  |  |  |  |  |  |  |  |  |  |  |  |  | ##### | 148.72 | ##### | ##### | 5.680 | 0.568 | 5.730 | 0.573 |  |  |  |  |
| 185 | 27 | Ecological rehabilitation | Forest | ＞20 | 36.86 | 109.33 | 0.611 | 0.061 | 0.601 | #### | 1.980 | 0.198 | 1.560 | 0.156 |  |  |  |  |  |  |  |  |  |  |  |  |  |  |  |  | ##### | 146.74 | ##### | ##### | 5.510 | 0.551 | 6.070 | 0.607 |  |  |  |  |
| 189 | 29 | Ecological rehabilitation | Grassland | 0-10 | 43.03 | 119.65 | 0.563 | 0.056 | 0.622 | #### | 3.840 | 0.384 | 2.040 | 0.204 |  |  |  |  |  |  |  |  |  |  |  |  |  |  |  |  | ##### | 135.63 | ##### | ##### | 5.370 | 0.537 | 5.520 | 0.552 |  |  |  |  |
| 190 | 29 | Ecological rehabilitation | Grassland | 10-20 | 43.03 | 119.65 | 0.612 | 0.061 | 0.633 | #### | 4.370 | 0.437 | 2.040 | 0.204 |  |  |  |  |  |  |  |  |  |  |  |  |  |  |  |  | ##### | 124.23 | ##### | ##### | 5.410 | 0.541 | 6.080 | 0.608 |  |  |  |  |
| 191 | 29 | Ecological rehabilitation | Grassland | ＞20 | 43.03 | 119.65 | 0.584 | 0.058 | 0.643 | #### | 9.010 | 0.901 | 2.360 | 0.236 |  |  |  |  |  |  |  |  |  |  |  |  |  |  |  |  | ##### | 100.23 | ##### | ##### | 4.590 | 0.459 | 6.000 | 0.600 |  |  |  |  |
| 207 | 31 | Ecological rehabilitation | Grassland | ＞20 | 33.13 | 102.62 | 0.580 | 0.058 | 0.663 | #### | 8.760 | 0.876 | 2.270 | 0.227 |  |  |  |  |  |  |  |  |  |  |  |  |  |  |  |  | ##### | 89.63 | ##### | ##### | 4.030 | 0.403 | 5.780 | 0.578 |  |  |  |  |
| 208 | 31 | Ecological rehabilitation | Grassland | ＞20 | 33.13 | 102.62 | 0.820 | 0.082 | 0.621 | #### | 9.220 | 0.922 | 2.870 | 0.287 |  |  |  |  |  |  |  |  |  |  |  |  |  |  |  |  | ##### | ##### | ##### | ##### | 6.420 | 0.642 | 6.330 | 0.633 |  |  |  |  |
| 209 | 31 | Ecological rehabilitation | Grassland | ＞20 | 33.13 | 102.62 | 0.681 | 0.068 | 0.553 | #### | 2.670 | 0.267 | 1.680 | 0.168 |  |  |  |  |  |  |  |  |  |  |  |  |  |  |  |  | ##### | 221.31 | ##### | ##### | 6.290 | 0.629 | 6.240 | 0.624 |  |  |  |  |
| 210 | 31 | Ecological rehabilitation | Grassland | ＞20 | 33.13 | 102.62 | 0.610 | 0.061 | 0.552 | #### | 2.650 | 0.265 | 1.790 | 0.179 |  |  |  |  |  |  |  |  |  |  |  |  |  |  |  |  | ##### | 174.24 | ##### | ##### | 6.210 | 0.621 | 6.110 | 0.611 |  |  |  |  |
| 211 | 31 | Ecological rehabilitation | Grassland | ＞20 | 33.13 | 102.62 | 0.600 | 0.060 | 0.551 | #### | 2.490 | 0.249 | 1.560 | 0.156 |  |  |  |  |  |  |  |  |  |  |  |  |  |  |  |  | ##### | 153.20 | ##### | ##### | 5.960 | 0.596 | 6.070 | 0.607 |  |  |  |  |
| 212 | 31 | Ecological rehabilitation | Grassland | ＞20 | 33.13 | 102.62 | 0.605 | 0.061 | 0.554 | #### | 1.590 | 0.159 | 3.160 | 0.316 |  |  |  |  |  |  |  |  |  |  |  |  |  |  |  |  | ##### | 148.72 | ##### | ##### | 5.680 | 0.568 | 5.970 | 0.597 |  |  |  |  |
| 216 | 32 | Ecological rehabilitation | Grassland | 0-10 | 44.25 | 123.57 | 0.611 | 0.061 | 0.552 | #### | 1.980 | 0.198 | 3.310 | 0.331 |  |  |  |  |  |  |  |  |  |  |  |  |  |  |  |  | ##### | 146.74 | ##### | ##### | 5.510 | 0.551 | 5.770 | 0.577 |  |  |  |  |
| 217 | 32 | Ecological rehabilitation | Grassland | 0-10 | 44.25 | 123.57 | 0.563 | 0.056 | 0.504 | #### | 3.840 | 0.384 | 5.070 | 0.507 |  |  |  |  |  |  |  |  |  |  |  |  |  |  |  |  | ##### | 135.63 | ##### | ##### | 5.370 | 0.537 | 5.680 | 0.568 |  |  |  |  |
| 218 | 32 | Ecological rehabilitation | Grassland | 0-10 | 44.25 | 123.57 | 0.612 | 0.061 | 0.531 | #### | 4.370 | 0.437 | 6.010 | 0.601 |  |  |  |  |  |  |  |  |  |  |  |  |  |  |  |  | ##### | 124.23 | ##### | ##### | 5.410 | 0.541 | 5.430 | 0.543 |  |  |  |  |
| 222 | 32 | Ecological rehabilitation | Grassland | 0-10 | 44.25 | 123.57 | 0.584 | 0.058 | 0.507 | 0.051 | 9.010 | 0.901 | 6.230 | 0.623 |  |  |  |  |  |  |  |  |  |  |  |  |  |  |  |  | ##### | 100.23 | ##### | ##### | 4.590 | 0.459 | 5.230 | 0.523 |  |  |  |  |
| 223 | 32 | Ecological rehabilitation | Grassland | 0-10 | 44.25 | 123.57 | 0.580 | 0.058 | 0.504 | #### | 8.760 | 0.876 | 9.380 | 0.938 |  |  |  |  |  |  |  |  |  |  |  |  |  |  |  |  | ##### | 89.63 | ##### | ##### | 4.030 | 0.403 | 5.160 | 0.516 |  |  |  |  |
| 224 | 32 | Ecological rehabilitation | Grassland | 0-10 | 44.25 | 123.57 |  |  |  |  | 2.030 | 0.203 | 3.260 | 0.326 |  |  |  |  |  |  |  |  |  |  |  |  |  |  |  |  |  |  |  |  | 0.420 | 0.042 | 0.650 | 0.065 |  |  |  |  |
| 236 | 35 | Ecological rehabilitation | Grassland | 0-10 | 32.52 | 91.67 |  |  |  |  | 1.690 | 0.169 | 2.740 | 0.274 |  |  |  |  |  |  |  |  |  |  |  |  |  |  |  |  |  |  |  |  | 0.413 | 0.041 | 0.640 | 0.064 |  |  |  |  |
| 322 | 52 | Ecological rehabilitation | Grassland | 10-20 | 47.45 | 126.92 |  |  |  |  | 2.030 | 0.203 | 4.130 | 0.413 |  |  |  |  |  |  |  |  |  |  |  |  |  |  |  |  |  |  |  |  | 0.420 | 0.042 | 0.720 | 0.072 |  |  |  |  |
| 323 | 53 | Ecological rehabilitation | Grassland | 0-10 | 44.07 | 125.70 |  |  |  |  | 1.690 | 0.169 | 2.690 | 0.269 |  |  |  |  |  |  |  |  |  |  |  |  |  |  |  |  |  |  |  |  | 0.413 | 0.041 | 0.710 | 0.071 |  |  |  |  |
| 324 | 53 | Ecological rehabilitation | Grassland | 0-10 | 44.07 | 125.70 |  |  |  |  | 2.030 | 0.203 | 4.080 | 0.408 |  |  |  |  |  |  |  |  |  |  |  |  |  |  |  |  |  |  |  |  | 0.420 | 0.042 | 0.780 | 0.078 |  |  |  |  |
| 325 | 53 | Ecological rehabilitation | Grassland | 0-10 | 44.07 | 125.70 |  |  |  |  | 1.690 | 0.169 | 3.130 | 0.313 |  |  |  |  |  |  |  |  |  |  |  |  |  |  |  |  |  |  |  |  | 0.413 | 0.041 | 0.760 | 0.076 |  |  |  |  |
| 329 | 54 | Ecological rehabilitation | Grassland | 0-10 | 42.67 | 121.52 |  |  |  |  | 2.030 | 0.203 | 5.540 | 0.554 |  |  |  |  |  |  |  |  |  |  |  |  |  |  |  |  |  |  |  |  | 0.420 | 0.042 | 0.750 | 0.075 |  |  |  |  |
| 330 | 54 | Ecological rehabilitation | Forest | 0-10 | 42.67 | 121.52 |  |  |  |  | 1.690 | 0.169 | 4.100 | 0.410 |  |  |  |  |  |  |  |  |  |  |  |  |  |  |  |  |  |  |  |  | 0.413 | 0.041 | 0.690 | 0.069 |  |  |  |  |
| 331 | 54 | Ecological rehabilitation | Forest | 0-10 | 42.67 | 121.52 |  |  |  |  | 2.030 | 0.203 | 4.210 | 0.421 |  |  |  |  |  |  |  |  |  |  |  |  |  |  |  |  |  |  |  |  | 0.420 | 0.042 | 0.660 | 0.066 |  |  |  |  |
| 332 | 55 | Ecological rehabilitation | Forest | 0-10 | 22.54 | 114.55 |  |  |  |  | 1.690 | 0.169 | 3.060 | 0.306 |  |  |  |  |  |  |  |  |  |  |  |  |  |  |  |  |  |  |  |  | 0.413 | 0.041 | 0.830 | 0.083 |  |  |  |  |
| 333 | 55 | Ecological rehabilitation | Forest | 0-10 | 22.54 | 114.55 |  |  |  |  | 2.030 | 0.203 | 5.470 | 0.547 |  |  |  |  |  |  |  |  |  |  |  |  |  |  |  |  |  |  |  |  | 0.420 | 0.042 | 0.850 | 0.085 |  |  |  |  |
| 334 | 56 | Ecological rehabilitation | Grassland | 0-10 | 25.62 | 116.46 |  |  |  |  | 1.690 | 0.169 | 3.012 | 0.301 |  |  |  |  |  |  |  |  |  |  |  |  |  |  |  |  |  |  |  |  | 0.413 | 0.041 | 0.800 | 0.080 |  |  |  |  |
| 335 | 56 | Ecological rehabilitation | Forest | 0-10 | 25.62 | 116.46 |  |  |  |  | 2.030 | 0.203 | 2.840 | 0.284 |  |  |  |  |  |  |  |  |  |  |  |  |  |  |  |  |  |  |  |  | 0.420 | 0.042 | 0.620 | 0.062 |  |  |  |  |
| 336 | 56 | Ecological rehabilitation | Forest | 10-20 | 25.62 | 116.46 |  |  |  |  | 1.690 | 0.169 | 1.950 | 0.195 |  |  |  |  |  |  |  |  |  |  |  |  |  |  |  |  |  |  |  |  | 0.413 | 0.041 | 0.460 | 0.046 |  |  |  |  |
| 337 | 56 | Ecological rehabilitation | Forest | 10-20 | 25.62 | 116.46 |  |  |  |  | 2.030 | 0.203 | 2.990 | 0.299 |  |  |  |  |  |  |  |  |  |  |  |  |  |  |  |  |  |  |  |  | 0.420 | 0.042 | 0.820 | 0.082 |  |  |  |  |
| 338 | 56 | Ecological rehabilitation | Forest | ＞20 | 25.62 | 116.46 |  |  |  |  | 1.690 | 0.169 | 2.570 | 0.257 |  |  |  |  |  |  |  |  |  |  |  |  |  |  |  |  |  |  |  |  | 0.413 | 0.041 | 0.640 | 0.064 |  |  |  |  |
| 339 | 57 | Ecological rehabilitation | Grassland | 0-10 | 45.53 | 126.26 |  |  |  |  | 2.030 | 0.203 | 2.740 | 0.274 |  |  |  |  |  |  |  |  |  |  |  |  |  |  |  |  |  |  |  |  | 0.420 | 0.042 | 0.400 | 0.040 |  |  |  |  |
| 340 | 57 | Ecological rehabilitation | Grassland | 0-10 | 45.53 | 126.26 |  |  |  |  | 1.690 | 0.169 | 2.660 | 0.266 |  |  |  |  |  |  |  |  |  |  |  |  |  |  |  |  |  |  |  |  | 0.413 | 0.041 | 0.630 | 0.063 |  |  |  |  |
| 341 | 57 | Ecological rehabilitation | Grassland | 0-10 | 45.53 | 126.26 | 1.080 | 0.080 | 0.790 | 0.130 |  |  |  |  |  |  |  |  |  |  |  |  |  |  |  |  |  |  |  |  |  |  |  |  |  |  |  |  |  |  |  |  |
| 342 | 58 | Ecological rehabilitation | Grassland | 0-10 | 33.72 | 102.43 | 0.530 | 0.140 | 0.610 | #### |  |  |  |  |  |  |  |  |  |  |  |  |  |  |  |  |  |  |  |  |  |  |  |  |  |  |  |  |  |  |  |  |
| 343 | 58 | Ecological rehabilitation | Grassland | 0-10 | 33.72 | 102.43 | 0.210 | 0.060 | 0.490 | #### |  |  |  |  |  |  |  |  |  |  |  |  |  |  |  |  |  |  |  |  |  |  |  |  |  |  |  |  |  |  |  |  |
| 344 | 58 | Ecological rehabilitation | Grassland | 10-20 | 33.72 | 102.43 | 1.080 | 0.080 | 1.190 | #### |  |  |  |  |  |  |  |  |  |  |  |  |  |  |  |  |  |  |  |  |  |  |  |  |  |  |  |  |  |  |  |  |
| 345 | 58 | Ecological rehabilitation | Grassland | 0-10 | 33.72 | 102.43 | 0.530 | 0.140 | 0.630 | 0.110 |  |  |  |  |  |  |  |  |  |  |  |  |  |  |  |  |  |  |  |  |  |  |  |  |  |  |  |  |  |  |  |  |
| 346 | 58 | Ecological rehabilitation | Grassland | 0-10 | 33.72 | 102.43 | 0.210 | 0.060 | 0.460 | 0.100 |  |  |  |  |  |  |  |  |  |  |  |  |  |  |  |  |  |  |  |  |  |  |  |  |  |  |  |  |  |  |  |  |
| 347 | 58 | Ecological rehabilitation | Grassland | 10-20 | 33.72 | 102.43 | 1.080 | 0.080 | 0.970 | #### |  |  |  |  |  |  |  |  |  |  |  |  |  |  |  |  |  |  |  |  |  |  |  |  |  |  |  |  |  |  |  |  |
| 348 | 58 | Ecological rehabilitation | Grassland | 0-10 | 33.72 | 102.43 | 0.530 | 0.140 | 0.960 | #### |  |  |  |  |  |  |  |  |  |  |  |  |  |  |  |  |  |  |  |  |  |  |  |  |  |  |  |  |  |  |  |  |
| 349 | 58 | Ecological rehabilitation | Grassland | 0-10 | 33.72 | 102.43 | 0.210 | 0.060 | 0.740 | 0.180 |  |  |  |  |  |  |  |  |  |  |  |  |  |  |  |  |  |  |  |  |  |  |  |  |  |  |  |  |  |  |  |  |
| 350 | 58 | Ecological rehabilitation | Grassland | 10-20 | 33.72 | 102.43 | 0.581 | 0.058 | 0.570 | #### | 6.010 | 0.601 | 7.490 | 0.749 |  |  |  |  | ##### | 4.03 | 42.21 | 4.22 |  |  |  |  |  |  |  |  | 5.89 | 0.589 | 5.67 | 0.57 | 1.070 | 0.107 | 1.260 | 0.126 | 0.436 | 0.044 | 0.389 | 0.039 |
| 352 | 60 | Ecological rehabilitation | Forest | 0-10 | 31.03 | 150.27 | 0.562 | 0.056 | 0.565 | #### | 7.640 | 0.764 | 8.220 | 0.822 |  |  |  |  | ##### | 5.87 | 62.23 | 6.22 |  |  |  |  |  |  |  |  | 7.55 | 0.755 | 6.07 | 0.61 | 1.243 | 0.124 | 1.150 | 0.115 | 0.397 | 0.040 | 0.40 | 0.04 |
| 353 | 60 | Ecological rehabilitation | Forest | 0-10 | 31.03 | 150.27 | 0.560 | 0.056 | 0.557 | #### | 10.010 | 1.001 | 8.340 | 0.834 |  |  |  |  | ##### | 8.35 | 125.50 | 12.55 |  |  |  |  |  |  |  |  | 3.27 | 0.327 | 3.30 | 0.33 | 0.860 | 0.086 | 1.230 | 0.123 | 0.411 | 0.041 | 0.396 | 0.04 |
| 354 | 60 | Ecological rehabilitation | Forest | 0-10 | 31.03 | 150.27 | 0.690 | 0.069 | 0.740 | #### | 8.441 | 0.844 | 8.430 | 0.843 |  |  |  |  | ##### | 10.46 | 122.46 | 12.25 |  |  |  |  |  |  |  |  | 6.19 | 0.619 | 7.68 | 0.77 | 1.264 | 0.126 | 1.540 | 0.154 | 0.376 | 0.038 | 0.269 | 0.027 |
| 355 | 60 | Ecological rehabilitation | Forest | 0-10 | 31.03 | 150.27 | 0.512 | 0.051 | 0.501 | #### | 3.070 | 0.307 | 4.220 | 0.422 |  |  |  |  | ##### | 10.32 | 107.68 | 10.77 |  |  |  |  |  |  |  |  | 5.97 | 0.597 | 4.77 | 0.48 | 1.190 | 0.119 | 1.010 | 0.101 | 0.376 | 0.038 | 0.409 | 0.041 |
| 356 | 60 | Ecological rehabilitation | Forest | 0-10 | 31.03 | 150.27 | 0.499 | 0.050 | 0.498 | #### | 4.650 | 0.465 | 4.860 | 0.486 |  |  |  |  | ##### | 4.03 | 42.21 | 4.22 |  |  |  |  |  |  |  |  | 5.89 | 0.589 | 5.67 | 0.57 | 1.070 | 0.107 | 1.260 | 0.126 | 0.436 | 0.044 | 0.389 | 0.039 |
| 357 | 60 | Ecological rehabilitation | Forest | 0-10 | 31.03 | 150.27 | 0.463 | 0.046 | 0.421 | #### | 8.160 | 0.816 | 6.220 | 0.622 |  |  |  |  | ##### | 5.87 | 62.23 | 6.22 |  |  |  |  |  |  |  |  | 7.55 | 0.755 | 6.07 | 0.61 | 1.243 | 0.124 | 1.150 | 0.115 | 0.397 | 0.040 | 0.40 | 0.04 |
| 390 | 64 | Ecological rehabilitation | Forest | 0-10 | 22.65 | 114.02 | 0.473 | 0.047 | 0.452 | #### | 4.680 | 0.468 | 4.090 | 0.409 |  |  |  |  | ##### | 8.35 | 125.50 | 12.55 |  |  |  |  |  |  |  |  | 3.27 | 0.327 | 3.30 | 0.33 | 0.860 | 0.086 | 1.230 | 0.123 | 0.411 | 0.041 | 0.396 | 0.04 |
| 391 | 64 | Ecological rehabilitation | Forest | 10-20 | 22.65 | 114.02 | 0.690 | 0.069 | 0.713 | 0.071 | 4.250 | 0.425 | 4.130 | 0.413 |  |  |  |  | ##### | 10.46 | 122.46 | 12.25 |  |  |  |  |  |  |  |  | 6.19 | 0.619 | 7.68 | 0.77 | 1.264 | 0.126 | 1.540 | 0.154 | 0.376 | 0.038 | 0.269 | 0.027 |
| 392 | 64 | Ecological rehabilitation | Forest | 10-20 | 22.65 | 114.02 | 0.452 | 0.045 | 0.401 | #### | 2.660 | 0.266 | 3.870 | 0.387 |  |  |  |  | ##### | 10.32 | 107.68 | 10.77 |  |  |  |  |  |  |  |  | 5.97 | 0.597 | 4.77 | 0.48 | 1.190 | 0.119 | 1.010 | 0.101 | 0.376 | 0.038 | 0.409 | 0.041 |
| 393 | 64 | Ecological rehabilitation | Forest | ＞20 | 22.65 | 114.02 | 0.530 | 0.020 | 0.600 | #### | 13.100 | 1.430 | 8.590 | 0.390 |  |  |  |  |  |  |  |  |  |  |  |  |  |  |  |  |  |  |  |  |  |  |  |  |  |  |  |  |
| 394 | 65 | Ecological rehabilitation | Forest | 10-20 | 32.37 | 73.87 | 0.530 | 0.020 | 0.590 | #### | 13.100 | 1.430 | 11.300 | 0.670 |  |  |  |  |  |  |  |  |  |  |  |  |  |  |  |  |  |  |  |  |  |  |  |  |  |  |  |  |
| 395 | 65 | Ecological rehabilitation | Forest | 10-20 | 32.37 | 73.87 | 0.530 | 0.020 | 0.690 | #### | 13.100 | 1.430 | 11.700 | 1.780 |  |  |  |  |  |  |  |  |  |  |  |  |  |  |  |  |  |  |  |  |  |  |  |  |  |  |  |  |
| 396 | 65 | Ecological rehabilitation | Forest | 10-20 | 32.37 | 73.87 | 0.400 | 0.010 | 0.430 | 0.010 | 2.650 | 0.070 | 2.940 | 0.030 | 8.63 | 1.850 | 30.20 | 2.330 | ##### | 74.80 | ##### | 29.80 |  |  |  |  |  |  |  |  | 10.30 | 1.33 | 19.70 | 1.200 | 2.060 | 0.050 | 2.610 | 0.050 |  |  |  |  |
| 454 | 70 | Ecological rehabilitation | Forest | 0-10 | 25.67 | 105.65 | 0.400 | 0.010 | 0.390 | 0.010 | 2.650 | 0.070 | 3.830 | 0.070 | 8.63 | 1.850 | 39.5 | 0.840 | ##### | 74.80 | ##### | 52.80 |  |  |  |  |  |  |  |  | 10.30 | 1.33 | 18.30 | 1.330 | 2.060 | 0.050 | 2.770 | 0.050 |  |  |  |  |
| 455 | 70 | Ecological rehabilitation | Forest | 0-10 | 25.67 | 105.65 | 0.400 | 0.010 | 0.380 | #### | 2.650 | 0.070 | 2.560 | 0.180 | 8.63 | 1.850 | 18.9 | 0.780 | ##### | 74.80 | ##### | 16.10 |  |  |  |  |  |  |  |  | 10.30 | 1.33 | 20.00 | 1.150 | 2.060 | 0.050 | 2.550 | 0.070 |  |  |  |  |
| 456 | 70 | Ecological rehabilitation | Forest | 10-20 | 25.67 | 105.65 | 0.400 | 0.010 | 0.440 | #### | 2.650 | 0.070 | 4.240 | 0.140 | 8.63 | 1.850 | 8.85 | 1.880 | ##### | 74.80 | ##### | 76.90 |  |  |  |  |  |  |  |  | 10.30 | 1.33 | 20.30 | 1.200 | 2.060 | 0.050 | 2.690 | 0.090 |  |  |  |  |
| 461 | 72 | Ecological rehabilitation | Forest | 10-20 | 25.64 | 116.46 |  |  |  |  | 0.531 | 0.053 | 1.230 | 0.123 |  |  |  |  |  |  |  |  |  |  |  |  |  |  |  |  |  |  |  |  |  |  |  |  |  |  |  |  |
| 462 | 72 | Ecological rehabilitation | Forest | 10-20 | 25.64 | 116.46 |  |  |  |  | 1.042 | 0.104 | 0.860 | 0.086 |  |  |  |  |  |  |  |  |  |  |  |  |  |  |  |  |  |  |  |  |  |  |  |  |  |  |  |  |
| 463 | 72 | Ecological rehabilitation | Forest | 10-20 | 25.64 | 116.46 |  |  |  |  | 2.369 | 0.237 | 0.870 | 0.087 |  |  |  |  |  |  |  |  |  |  |  |  |  |  |  |  |  |  |  |  |  |  |  |  |  |  |  |  |
|  |  |  |  |  |  |  |  |  |  |  |  |  |  |  |  |  |  |  |  |  |  |  |  |  |  |  |  |  |  |  |  |  |  |  |  |  |  |  |  |  |  |  |
|  |  |  |  |  |  |  |  |  |  |  |  |  |  |  |  |  |  |  |  |  |  |  |  |  |  |  |  |  |  |  |  |  |  |  |  |  |  |  |  |  |  |  |
|  |  |  |  |  |  |  |  |  |  |  |  |  |  |  |  |  |  |  |  |  |  |  |  |  |  |  |  |  |  |  |  |  |  |  |  |  |  |  |  |  |  |  |
|  |  |  |  |  |  |  |  |  |  |  |  |  |  |  |  |  |  |  |  |  |  |  |  |  |  |  |  |  |  |  |  |  |  |  |  |  |  |  |  |  |  |  |
|  |  |  |  |  |  |  |  |  |  |  |  |  |  |  |  |  |  |  |  |  |  |  |  |  |  |  |  |  |  |  |  |  |  |  |  |  |  |  |  |  |  |  |
|  |  |  |  |  |  |  |  |  |  |  |  |  |  |  |  |  |  |  |  |  |  |  |  |  |  |  |  |  |  |  |  |  |  |  |  |  |  |  |  |  |  |  |
|  |  |  |  |  |  |  |  |  |  |  |  |  |  |  |  |  |  |  |  |  |  |  |  |  |  |  |  |  |  |  |  |  |  |  |  |  |  |  |  |  |  |  |
|  |  |  |  |  |  |  |  |  |  |  |  |  |  |  |  |  |  |  |  |  |  |  |  |  |  |  |  |  |  |  |  |  |  |  |  |  |  |  |  |  |  |  |
|  |  |  |  |  |  |  |  |  |  |  |  |  |  |  |  |  |  |  |  |  |  |  |  |  |  |  |  |  |  |  |  |  |  |  |  |  |  |  |  |  |  |  |
|  |  |  |  |  |  |  |  |  |  |  |  |  |  |  |  |  |  |  |  |  |  |  |  |  |  |  |  |  |  |  |  |  |  |  |  |  |  |  |  |  |  |  |
|  |  |  |  |  |  |  |  |  |  |  |  |  |  |  |  |  |  |  |  |  |  |  |  |  |  |  |  |  |  |  |  |  |  |  |  |  |  |  |  |  |  |  |
|  |  |  |  |  |  |  |  |  |  |  |  |  |  |  |  |  |  |  |  |  |  |  |  |  |  |  |  |  |  |  |  |  |  |  |  |  |  |  |  |  |  |  |
|  |  |  |  |  |  |  |  |  |  |  |  |  |  |  |  |  |  |  |  |  |  |  |  |  |  |  |  |  |  |  |  |  |  |  |  |  |  |  |  |  |  |  |
|  |  |  |  |  |  |  |  |  |  |  |  |  |  |  |  |  |  |  |  |  |  |  |  |  |  |  |  |  |  |  |  |  |  |  |  |  |  |  |  |  |  |  |
|  |  |  |  |  |  |  |  |  |  |  |  |  |  |  |  |  |  |  |  |  |  |  |  |  |  |  |  |  |  |  |  |  |  |  |  |  |  |  |  |  |  |  |
|  |  |  |  |  |  |  |  |  |  |  |  |  |  |  |  |  |  |  |  |  |  |  |  |  |  |  |  |  |  |  |  |  |  |  |  |  |  |  |  |  |  |  |
|  |  |  |  |  |  |  |  |  |  |  |  |  |  |  |  |  |  |  |  |  |  |  |  |  |  |  |  |  |  |  |  |  |  |  |  |  |  |  |  |  |  |  |
|  |  |  |  |  |  |  |  |  |  |  |  |  |  |  |  |  |  |  |  |  |  |  |  |  |  |  |  |  |  |  |  |  |  |  |  |  |  |  |  |  |  |  |
|  |  |  |  |  |  |  |  |  |  |  |  |  |  |  |  |  |  |  |  |  |  |  |  |  |  |  |  |  |  |  |  |  |  |  |  |  |  |  |  |  |  |  |
|  |  |  |  |  |  |  |  |  |  |  |  |  |  |  |  |  |  |  |  |  |  |  |  |  |  |  |  |  |  |  |  |  |  |  |  |  |  |  |  |  |  |  |
|  |  |  |  |  |  |  |  |  |  |  |  |  |  |  |  |  |  |  |  |  |  |  |  |  |  |  |  |  |  |  |  |  |  |  |  |  |  |  |  |  |  |  |
|  |  |  |  |  |  |  |  |  |  |  |  |  |  |  |  |  |  |  |  |  |  |  |  |  |  |  |  |  |  |  |  |  |  |  |  |  |  |  |  |  |  |  |
|  |  |  |  |  |  |  |  |  |  |  |  |  |  |  |  |  |  |  |  |  |  |  |  |  |  |  |  |  |  |  |  |  |  |  |  |  |  |  |  |  |  |  |
|  |  |  |  |  |  |  |  |  |  |  |  |  |  |  |  |  |  |  |  |  |  |  |  |  |  |  |  |  |  |  |  |  |  |  |  |  |  |  |  |  |  |  |
|  |  |  |  |  |  |  |  |  |  |  |  |  |  |  |  |  |  |  |  |  |  |  |  |  |  |  |  |  |  |  |  |  |  |  |  |  |  |  |  |  |  |  |
|  |  |  |  |  |  |  |  |  |  |  |  |  |  |  |  |  |  |  |  |  |  |  |  |  |  |  |  |  |  |  |  |  |  |  |  |  |  |  |  |  |  |  |
|  |  |  |  |  |  |  |  |  |  |  |  |  |  |  |  |  |  |  |  |  |  |  |  |  |  |  |  |  |  |  |  |  |  |  |  |  |  |  |  |  |  |  |
|  |  |  |  |  |  |  |  |  |  |  |  |  |  |  |  |  |  |  |  |  |  |  |  |  |  |  |  |  |  |  |  |  |  |  |  |  |  |  |  |  |  |  |
|  |  |  |  |  |  |  |  |  |  |  |  |  |  |  |  |  |  |  |  |  |  |  |  |  |  |  |  |  |  |  |  |  |  |  |  |  |  |  |  |  |  |  |
|  |  |  |  |  |  |  |  |  |  |  |  |  |  |  |  |  |  |  |  |  |  |  |  |  |  |  |  |  |  |  |  |  |  |  |  |  |  |  |  |  |  |  |
|  |  |  |  |  |  |  |  |  |  |  |  |  |  |  |  |  |  |  |  |  |  |  |  |  |  |  |  |  |  |  |  |  |  |  |  |  |  |  |  |  |  |  |
|  |  |  |  |  |  |  |  |  |  |  |  |  |  |  |  |  |  |  |  |  |  |  |  |  |  |  |  |  |  |  |  |  |  |  |  |  |  |  |  |  |  |  |
|  |  |  |  |  |  |  |  |  |  |  |  |  |  |  |  |  |  |  |  |  |  |  |  |  |  |  |  |  |  |  |  |  |  |  |  |  |  |  |  |  |  |  |
|  |  |  |  |  |  |  |  |  |  |  |  |  |  |  |  |  |  |  |  |  |  |  |  |  |  |  |  |  |  |  |  |  |  |  |  |  |  |  |  |  |  |  |
|  |  |  |  |  |  |  |  |  |  |  |  |  |  |  |  |  |  |  |  |  |  |  |  |  |  |  |  |  |  |  |  |  |  |  |  |  |  |  |  |  |  |  |
|  |  |  |  |  |  |  |  |  |  |  |  |  |  |  |  |  |  |  |  |  |  |  |  |  |  |  |  |  |  |  |  |  |  |  |  |  |  |  |  |  |  |  |
|  |  |  |  |  |  |  |  |  |  |  |  |  |  |  |  |  |  |  |  |  |  |  |  |  |  |  |  |  |  |  |  |  |  |  |  |  |  |  |  |  |  |  |
|  |  |  |  |  |  |  |  |  |  |  |  |  |  |  |  |  |  |  |  |  |  |  |  |  |  |  |  |  |  |  |  |  |  |  |  |  |  |  |  |  |  |  |
|  |  |  |  |  |  |  |  |  |  |  |  |  |  |  |  |  |  |  |  |  |  |  |  |  |  |  |  |  |  |  |  |  |  |  |  |  |  |  |  |  |  |  |
|  |  |  |  |  |  |  |  |  |  |  |  |  |  |  |  |  |  |  |  |  |  |  |  |  |  |  |  |  |  |  |  |  |  |  |  |  |  |  |  |  |  |  |
|  |  |  |  |  |  |  |  |  |  |  |  |  |  |  |  |  |  |  |  |  |  |  |  |  |  |  |  |  |  |  |  |  |  |  |  |  |  |  |  |  |  |  |
|  |  |  |  |  |  |  |  |  |  |  |  |  |  |  |  |  |  |  |  |  |  |  |  |  |  |  |  |  |  |  |  |  |  |  |  |  |  |  |  |  |  |  |
|  |  |  |  |  |  |  |  |  |  |  |  |  |  |  |  |  |  |  |  |  |  |  |  |  |  |  |  |  |  |  |  |  |  |  |  |  |  |  |  |  |  |  |
|  |  |  |  |  |  |  |  |  |  |  |  |  |  |  |  |  |  |  |  |  |  |  |  |  |  |  |  |  |  |  |  |  |  |  |  |  |  |  |  |  |  |  |
|  |  |  |  |  |  |  |  |  |  |  |  |  |  |  |  |  |  |  |  |  |  |  |  |  |  |  |  |  |  |  |  |  |  |  |  |  |  |  |  |  |  |  |
|  |  |  |  |  |  |  |  |  |  |  |  |  |  |  |  |  |  |  |  |  |  |  |  |  |  |  |  |  |  |  |  |  |  |  |  |  |  |  |  |  |  |  |
|  |  |  |  |  |  |  |  |  |  |  |  |  |  |  |  |  |  |  |  |  |  |  |  |  |  |  |  |  |  |  |  |  |  |  |  |  |  |  |  |  |  |  |
|  |  |  |  |  |  |  |  |  |  |  |  |  |  |  |  |  |  |  |  |  |  |  |  |  |  |  |  |  |  |  |  |  |  |  |  |  |  |  |  |  |  |  |
|  |  |  |  |  |  |  |  |  |  |  |  |  |  |  |  |  |  |  |  |  |  |  |  |  |  |  |  |  |  |  |  |  |  |  |  |  |  |  |  |  |  |  |
|  |  |  |  |  |  |  |  |  |  |  |  |  |  |  |  |  |  |  |  |  |  |  |  |  |  |  |  |  |  |  |  |  |  |  |  |  |  |  |  |  |  |  |
|  |  |  |  |  |  |  |  |  |  |  |  |  |  |  |  |  |  |  |  |  |  |  |  |  |  |  |  |  |  |  |  |  |  |  |  |  |  |  |  |  |  |  |
|  |  |  |  |  |  |  |  |  |  |  |  |  |  |  |  |  |  |  |  |  |  |  |  |  |  |  |  |  |  |  |  |  |  |  |  |  |  |  |  |  |  |  |
|  |  |  |  |  |  |  |  |  |  |  |  |  |  |  |  |  |  |  |  |  |  |  |  |  |  |  |  |  |  |  |  |  |  |  |  |  |  |  |  |  |  |  |
|  |  |  |  |  |  |  |  |  |  |  |  |  |  |  |  |  |  |  |  |  |  |  |  |  |  |  |  |  |  |  |  |  |  |  |  |  |  |  |  |  |  |  |
|  |  |  |  |  |  |  |  |  |  |  |  |  |  |  |  |  |  |  |  |  |  |  |  |  |  |  |  |  |  |  |  |  |  |  |  |  |  |  |  |  |  |  |
|  |  |  |  |  |  |  |  |  |  |  |  |  |  |  |  |  |  |  |  |  |  |  |  |  |  |  |  |  |  |  |  |  |  |  |  |  |  |  |  |  |  |  |
|  |  |  |  |  |  |  |  |  |  |  |  |  |  |  |  |  |  |  |  |  |  |  |  |  |  |  |  |  |  |  |  |  |  |  |  |  |  |  |  |  |  |  |
|  |  |  |  |  |  |  |  |  |  |  |  |  |  |  |  |  |  |  |  |  |  |  |  |  |  |  |  |  |  |  |  |  |  |  |  |  |  |  |  |  |  |  |
|  |  |  |  |  |  |  |  |  |  |  |  |  |  |  |  |  |  |  |  |  |  |  |  |  |  |  |  |  |  |  |  |  |  |  |  |  |  |  |  |  |  |  |
|  |  |  |  |  |  |  |  |  |  |  |  |  |  |  |  |  |  |  |  |  |  |  |  |  |  |  |  |  |  |  |  |  |  |  |  |  |  |  |  |  |  |  |
|  |  |  |  |  |  |  |  |  |  |  |  |  |  |  |  |  |  |  |  |  |  |  |  |  |  |  |  |  |  |  |  |  |  |  |  |  |  |  |  |  |  |  |
|  |  |  |  |  |  |  |  |  |  |  |  |  |  |  |  |  |  |  |  |  |  |  |  |  |  |  |  |  |  |  |  |  |  |  |  |  |  |  |  |  |  |  |
|  |  |  |  |  |  |  |  |  |  |  |  |  |  |  |  |  |  |  |  |  |  |  |  |  |  |  |  |  |  |  |  |  |  |  |  |  |  |  |  |  |  |  |
|  |  |  |  |  |  |  |  |  |  |  |  |  |  |  |  |  |  |  |  |  |  |  |  |  |  |  |  |  |  |  |  |  |  |  |  |  |  |  |  |  |  |  |
|  |  |  |  |  |  |  |  |  |  |  |  |  |  |  |  |  |  |  |  |  |  |  |  |  |  |  |  |  |  |  |  |  |  |  |  |  |  |  |  |  |  |  |
|  |  |  |  |  |  |  |  |  |  |  |  |  |  |  |  |  |  |  |  |  |  |  |  |  |  |  |  |  |  |  |  |  |  |  |  |  |  |  |  |  |  |  |
|  |  |  |  |  |  |  |  |  |  |  |  |  |  |  |  |  |  |  |  |  |  |  |  |  |  |  |  |  |  |  |  |  |  |  |  |  |  |  |  |  |  |  |
|  |  |  |  |  |  |  |  |  |  |  |  |  |  |  |  |  |  |  |  |  |  |  |  |  |  |  |  |  |  |  |  |  |  |  |  |  |  |  |  |  |  |  |
|  |  |  |  |  |  |  |  |  |  |  |  |  |  |  |  |  |  |  |  |  |  |  |  |  |  |  |  |  |  |  |  |  |  |  |  |  |  |  |  |  |  |  |
|  |  |  |  |  |  |  |  |  |  |  |  |  |  |  |  |  |  |  |  |  |  |  |  |  |  |  |  |  |  |  |  |  |  |  |  |  |  |  |  |  |  |  |
|  |  |  |  |  |  |  |  |  |  |  |  |  |  |  |  |  |  |  |  |  |  |  |  |  |  |  |  |  |  |  |  |  |  |  |  |  |  |  |  |  |  |  |
|  |  |  |  |  |  |  |  |  |  |  |  |  |  |  |  |  |  |  |  |  |  |  |  |  |  |  |  |  |  |  |  |  |  |  |  |  |  |  |  |  |  |  |
|  |  |  |  |  |  |  |  |  |  |  |  |  |  |  |  |  |  |  |  |  |  |  |  |  |  |  |  |  |  |  |  |  |  |  |  |  |  |  |  |  |  |  |
|  |  |  |  |  |  |  |  |  |  |  |  |  |  |  |  |  |  |  |  |  |  |  |  |  |  |  |  |  |  |  |  |  |  |  |  |  |  |  |  |  |  |  |
|  |  |  |  |  |  |  |  |  |  |  |  |  |  |  |  |  |  |  |  |  |  |  |  |  |  |  |  |  |  |  |  |  |  |  |  |  |  |  |  |  |  |  |
|  |  |  |  |  |  |  |  |  |  |  |  |  |  |  |  |  |  |  |  |  |  |  |  |  |  |  |  |  |  |  |  |  |  |  |  |  |  |  |  |  |  |  |
|  |  |  |  |  |  |  |  |  |  |  |  |  |  |  |  |  |  |  |  |  |  |  |  |  |  |  |  |  |  |  |  |  |  |  |  |  |  |  |  |  |  |  |
|  |  |  |  |  |  |  |  |  |  |  |  |  |  |  |  |  |  |  |  |  |  |  |  |  |  |  |  |  |  |  |  |  |  |  |  |  |  |  |  |  |  |  |
|  |  |  |  |  |  |  |  |  |  |  |  |  |  |  |  |  |  |  |  |  |  |  |  |  |  |  |  |  |  |  |  |  |  |  |  |  |  |  |  |  |  |  |
|  |  |  |  |  |  |  |  |  |  |  |  |  |  |  |  |  |  |  |  |  |  |  |  |  |  |  |  |  |  |  |  |  |  |  |  |  |  |  |  |  |  |  |
|  |  |  |  |  |  |  |  |  |  |  |  |  |  |  |  |  |  |  |  |  |  |  |  |  |  |  |  |  |  |  |  |  |  |  |  |  |  |  |  |  |  |  |
|  |  |  |  |  |  |  |  |  |  |  |  |  |  |  |  |  |  |  |  |  |  |  |  |  |  |  |  |  |  |  |  |  |  |  |  |  |  |  |  |  |  |  |
|  |  |  |  |  |  |  |  |  |  |  |  |  |  |  |  |  |  |  |  |  |  |  |  |  |  |  |  |  |  |  |  |  |  |  |  |  |  |  |  |  |  |  |
|  |  |  |  |  |  |  |  |  |  |  |  |  |  |  |  |  |  |  |  |  |  |  |  |  |  |  |  |  |  |  |  |  |  |  |  |  |  |  |  |  |  |  |
|  |  |  |  |  |  |  |  |  |  |  |  |  |  |  |  |  |  |  |  |  |  |  |  |  |  |  |  |  |  |  |  |  |  |  |  |  |  |  |  |  |  |  |
|  |  |  |  |  |  |  |  |  |  |  |  |  |  |  |  |  |  |  |  |  |  |  |  |  |  |  |  |  |  |  |  |  |  |  |  |  |  |  |  |  |  |  |
|  |  |  |  |  |  |  |  |  |  |  |  |  |  |  |  |  |  |  |  |  |  |  |  |  |  |  |  |  |  |  |  |  |  |  |  |  |  |  |  |  |  |  |
|  |  |  |  |  |  |  |  |  |  |  |  |  |  |  |  |  |  |  |  |  |  |  |  |  |  |  |  |  |  |  |  |  |  |  |  |  |  |  |  |  |  |  |
|  |  |  |  |  |  |  |  |  |  |  |  |  |  |  |  |  |  |  |  |  |  |  |  |  |  |  |  |  |  |  |  |  |  |  |  |  |  |  |  |  |  |  |
|  |  |  |  |  |  |  |  |  |  |  |  |  |  |  |  |  |  |  |  |  |  |  |  |  |  |  |  |  |  |  |  |  |  |  |  |  |  |  |  |  |  |  |
|  |  |  |  |  |  |  |  |  |  |  |  |  |  |  |  |  |  |  |  |  |  |  |  |  |  |  |  |  |  |  |  |  |  |  |  |  |  |  |  |  |  |  |
|  |  |  |  |  |  |  |  |  |  |  |  |  |  |  |  |  |  |  |  |  |  |  |  |  |  |  |  |  |  |  |  |  |  |  |  |  |  |  |  |  |  |  |
|  |  |  |  |  |  |  |  |  |  |  |  |  |  |  |  |  |  |  |  |  |  |  |  |  |  |  |  |  |  |  |  |  |  |  |  |  |  |  |  |  |  |  |
|  |  |  |  |  |  |  |  |  |  |  |  |  |  |  |  |  |  |  |  |  |  |  |  |  |  |  |  |  |  |  |  |  |  |  |  |  |  |  |  |  |  |  |
|  |  |  |  |  |  |  |  |  |  |  |  |  |  |  |  |  |  |  |  |  |  |  |  |  |  |  |  |  |  |  |  |  |  |  |  |  |  |  |  |  |  |  |
|  |  |  |  |  |  |  |  |  |  |  |  |  |  |  |  |  |  |  |  |  |  |  |  |  |  |  |  |  |  |  |  |  |  |  |  |  |  |  |  |  |  |  |
|  |  |  |  |  |  |  |  |  |  |  |  |  |  |  |  |  |  |  |  |  |  |  |  |  |  |  |  |  |  |  |  |  |  |  |  |  |  |  |  |  |  |  |
|  |  |  |  |  |  |  |  |  |  |  |  |  |  |  |  |  |  |  |  |  |  |  |  |  |  |  |  |  |  |  |  |  |  |  |  |  |  |  |  |  |  |  |
|  |  |  |  |  |  |  |  |  |  |  |  |  |  |  |  |  |  |  |  |  |  |  |  |  |  |  |  |  |  |  |  |  |  |  |  |  |  |  |  |  |  |  |
|  |  |  |  |  |  |  |  |  |  |  |  |  |  |  |  |  |  |  |  |  |  |  |  |  |  |  |  |  |  |  |  |  |  |  |  |  |  |  |  |  |  |  |
|  |  |  |  |  |  |  |  |  |  |  |  |  |  |  |  |  |  |  |  |  |  |  |  |  |  |  |  |  |  |  |  |  |  |  |  |  |  |  |  |  |  |  |
|  |  |  |  |  |  |  |  |  |  |  |  |  |  |  |  |  |  |  |  |  |  |  |  |  |  |  |  |  |  |  |  |  |  |  |  |  |  |  |  |  |  |  |
|  |  |  |  |  |  |  |  |  |  |  |  |  |  |  |  |  |  |  |  |  |  |  |  |  |  |  |  |  |  |  |  |  |  |  |  |  |  |  |  |  |  |  |
|  |  |  |  |  |  |  |  |  |  |  |  |  |  |  |  |  |  |  |  |  |  |  |  |  |  |  |  |  |  |  |  |  |  |  |  |  |  |  |  |  |  |  |
|  |  |  |  |  |  |  |  |  |  |  |  |  |  |  |  |  |  |  |  |  |  |  |  |  |  |  |  |  |  |  |  |  |  |  |  |  |  |  |  |  |  |  |
|  |  |  |  |  |  |  |  |  |  |  |  |  |  |  |  |  |  |  |  |  |  |  |  |  |  |  |  |  |  |  |  |  |  |  |  |  |  |  |  |  |  |  |
|  |  |  |  |  |  |  |  |  |  |  |  |  |  |  |  |  |  |  |  |  |  |  |  |  |  |  |  |  |  |  |  |  |  |  |  |  |  |  |  |  |  |  |
|  |  |  |  |  |  |  |  |  |  |  |  |  |  |  |  |  |  |  |  |  |  |  |  |  |  |  |  |  |  |  |  |  |  |  |  |  |  |  |  |  |  |  |
|  |  |  |  |  |  |  |  |  |  |  |  |  |  |  |  |  |  |  |  |  |  |  |  |  |  |  |  |  |  |  |  |  |  |  |  |  |  |  |  |  |  |  |
|  |  |  |  |  |  |  |  |  |  |  |  |  |  |  |  |  |  |  |  |  |  |  |  |  |  |  |  |  |  |  |  |  |  |  |  |  |  |  |  |  |  |  |
|  |  |  |  |  |  |  |  |  |  |  |  |  |  |  |  |  |  |  |  |  |  |  |  |  |  |  |  |  |  |  |  |  |  |  |  |  |  |  |  |  |  |  |
|  |  |  |  |  |  |  |  |  |  |  |  |  |  |  |  |  |  |  |  |  |  |  |  |  |  |  |  |  |  |  |  |  |  |  |  |  |  |  |  |  |  |  |
|  |  |  |  |  |  |  |  |  |  |  |  |  |  |  |  |  |  |  |  |  |  |  |  |  |  |  |  |  |  |  |  |  |  |  |  |  |  |  |  |  |  |  |
|  |  |  |  |  |  |  |  |  |  |  |  |  |  |  |  |  |  |  |  |  |  |  |  |  |  |  |  |  |  |  |  |  |  |  |  |  |  |  |  |  |  |  |
|  |  |  |  |  |  |  |  |  |  |  |  |  |  |  |  |  |  |  |  |  |  |  |  |  |  |  |  |  |  |  |  |  |  |  |  |  |  |  |  |  |  |  |
|  |  |  |  |  |  |  |  |  |  |  |  |  |  |  |  |  |  |  |  |  |  |  |  |  |  |  |  |  |  |  |  |  |  |  |  |  |  |  |  |  |  |  |
|  |  |  |  |  |  |  |  |  |  |  |  |  |  |  |  |  |  |  |  |  |  |  |  |  |  |  |  |  |  |  |  |  |  |  |  |  |  |  |  |  |  |  |
|  |  |  |  |  |  |  |  |  |  |  |  |  |  |  |  |  |  |  |  |  |  |  |  |  |  |  |  |  |  |  |  |  |  |  |  |  |  |  |  |  |  |  |
|  |  |  |  |  |  |  |  |  |  |  |  |  |  |  |  |  |  |  |  |  |  |  |  |  |  |  |  |  |  |  |  |  |  |  |  |  |  |  |  |  |  |  |
|  |  |  |  |  |  |  |  |  |  |  |  |  |  |  |  |  |  |  |  |  |  |  |  |  |  |  |  |  |  |  |  |  |  |  |  |  |  |  |  |  |  |  |
|  |  |  |  |  |  |  |  |  |  |  |  |  |  |  |  |  |  |  |  |  |  |  |  |  |  |  |  |  |  |  |  |  |  |  |  |  |  |  |  |  |  |  |
|  |  |  |  |  |  |  |  |  |  |  |  |  |  |  |  |  |  |  |  |  |  |  |  |  |  |  |  |  |  |  |  |  |  |  |  |  |  |  |  |  |  |  |
|  |  |  |  |  |  |  |  |  |  |  |  |  |  |  |  |  |  |  |  |  |  |  |  |  |  |  |  |  |  |  |  |  |  |  |  |  |  |  |  |  |  |  |
|  |  |  |  |  |  |  |  |  |  |  |  |  |  |  |  |  |  |  |  |  |  |  |  |  |  |  |  |  |  |  |  |  |  |  |  |  |  |  |  |  |  |  |
|  |  |  |  |  |  |  |  |  |  |  |  |  |  |  |  |  |  |  |  |  |  |  |  |  |  |  |  |  |  |  |  |  |  |  |  |  |  |  |  |  |  |  |
|  |  |  |  |  |  |  |  |  |  |  |  |  |  |  |  |  |  |  |  |  |  |  |  |  |  |  |  |  |  |  |  |  |  |  |  |  |  |  |  |  |  |  |
|  |  |  |  |  |  |  |  |  |  |  |  |  |  |  |  |  |  |  |  |  |  |  |  |  |  |  |  |  |  |  |  |  |  |  |  |  |  |  |  |  |  |  |
|  |  |  |  |  |  |  |  |  |  |  |  |  |  |  |  |  |  |  |  |  |  |  |  |  |  |  |  |  |  |  |  |  |  |  |  |  |  |  |  |  |  |  |
|  |  |  |  |  |  |  |  |  |  |  |  |  |  |  |  |  |  |  |  |  |  |  |  |  |  |  |  |  |  |  |  |  |  |  |  |  |  |  |  |  |  |  |
|  |  |  |  |  |  |  |  |  |  |  |  |  |  |  |  |  |  |  |  |  |  |  |  |  |  |  |  |  |  |  |  |  |  |  |  |  |  |  |  |  |  |  |
|  |  |  |  |  |  |  |  |  |  |  |  |  |  |  |  |  |  |  |  |  |  |  |  |  |  |  |  |  |  |  |  |  |  |  |  |  |  |  |  |  |  |  |
|  |  |  |  |  |  |  |  |  |  |  |  |  |  |  |  |  |  |  |  |  |  |  |  |  |  |  |  |  |  |  |  |  |  |  |  |  |  |  |  |  |  |  |
|  |  |  |  |  |  |  |  |  |  |  |  |  |  |  |  |  |  |  |  |  |  |  |  |  |  |  |  |  |  |  |  |  |  |  |  |  |  |  |  |  |  |  |
|  |  |  |  |  |  |  |  |  |  |  |  |  |  |  |  |  |  |  |  |  |  |  |  |  |  |  |  |  |  |  |  |  |  |  |  |  |  |  |  |  |  |  |
|  |  |  |  |  |  |  |  |  |  |  |  |  |  |  |  |  |  |  |  |  |  |  |  |  |  |  |  |  |  |  |  |  |  |  |  |  |  |  |  |  |  |  |
|  |  |  |  |  |  |  |  |  |  |  |  |  |  |  |  |  |  |  |  |  |  |  |  |  |  |  |  |  |  |  |  |  |  |  |  |  |  |  |  |  |  |  |
|  |  |  |  |  |  |  |  |  |  |  |  |  |  |  |  |  |  |  |  |  |  |  |  |  |  |  |  |  |  |  |  |  |  |  |  |  |  |  |  |  |  |  |
|  |  |  |  |  |  |  |  |  |  |  |  |  |  |  |  |  |  |  |  |  |  |  |  |  |  |  |  |  |  |  |  |  |  |  |  |  |  |  |  |  |  |  |
|  |  |  |  |  |  |  |  |  |  |  |  |  |  |  |  |  |  |  |  |  |  |  |  |  |  |  |  |  |  |  |  |  |  |  |  |  |  |  |  |  |  |  |
|  |  |  |  |  |  |  |  |  |  |  |  |  |  |  |  |  |  |  |  |  |  |  |  |  |  |  |  |  |  |  |  |  |  |  |  |  |  |  |  |  |  |  |
|  |  |  |  |  |  |  |  |  |  |  |  |  |  |  |  |  |  |  |  |  |  |  |  |  |  |  |  |  |  |  |  |  |  |  |  |  |  |  |  |  |  |  |
|  |  |  |  |  |  |  |  |  |  |  |  |  |  |  |  |  |  |  |  |  |  |  |  |  |  |  |  |  |  |  |  |  |  |  |  |  |  |  |  |  |  |  |
|  |  |  |  |  |  |  |  |  |  |  |  |  |  |  |  |  |  |  |  |  |  |  |  |  |  |  |  |  |  |  |  |  |  |  |  |  |  |  |  |  |  |  |
|  |  |  |  |  |  |  |  |  |  |  |  |  |  |  |  |  |  |  |  |  |  |  |  |  |  |  |  |  |  |  |  |  |  |  |  |  |  |  |  |  |  |  |
|  |  |  |  |  |  |  |  |  |  |  |  |  |  |  |  |  |  |  |  |  |  |  |  |  |  |  |  |  |  |  |  |  |  |  |  |  |  |  |  |  |  |  |
|  |  |  |  |  |  |  |  |  |  |  |  |  |  |  |  |  |  |  |  |  |  |  |  |  |  |  |  |  |  |  |  |  |  |  |  |  |  |  |  |  |  |  |
|  |  |  |  |  |  |  |  |  |  |  |  |  |  |  |  |  |  |  |  |  |  |  |  |  |  |  |  |  |  |  |  |  |  |  |  |  |  |  |  |  |  |  |
|  |  |  |  |  |  |  |  |  |  |  |  |  |  |  |  |  |  |  |  |  |  |  |  |  |  |  |  |  |  |  |  |  |  |  |  |  |  |  |  |  |  |  |
|  |  |  |  |  |  |  |  |  |  |  |  |  |  |  |  |  |  |  |  |  |  |  |  |  |  |  |  |  |  |  |  |  |  |  |  |  |  |  |  |  |  |  |
|  |  |  |  |  |  |  |  |  |  |  |  |  |  |  |  |  |  |  |  |  |  |  |  |  |  |  |  |  |  |  |  |  |  |  |  |  |  |  |  |  |  |  |
|  |  |  |  |  |  |  |  |  |  |  |  |  |  |  |  |  |  |  |  |  |  |  |  |  |  |  |  |  |  |  |  |  |  |  |  |  |  |  |  |  |  |  |
|  |  |  |  |  |  |  |  |  |  |  |  |  |  |  |  |  |  |  |  |  |  |  |  |  |  |  |  |  |  |  |  |  |  |  |  |  |  |  |  |  |  |  |
|  |  |  |  |  |  |  |  |  |  |  |  |  |  |  |  |  |  |  |  |  |  |  |  |  |  |  |  |  |  |  |  |  |  |  |  |  |  |  |  |  |  |  |
|  |  |  |  |  |  |  |  |  |  |  |  |  |  |  |  |  |  |  |  |  |  |  |  |  |  |  |  |  |  |  |  |  |  |  |  |  |  |  |  |  |  |  |
|  |  |  |  |  |  |  |  |  |  |  |  |  |  |  |  |  |  |  |  |  |  |  |  |  |  |  |  |  |  |  |  |  |  |  |  |  |  |  |  |  |  |  |
|  |  |  |  |  |  |  |  |  |  |  |  |  |  |  |  |  |  |  |  |  |  |  |  |  |  |  |  |  |  |  |  |  |  |  |  |  |  |  |  |  |  |  |
|  |  |  |  |  |  |  |  |  |  |  |  |  |  |  |  |  |  |  |  |  |  |  |  |  |  |  |  |  |  |  |  |  |  |  |  |  |  |  |  |  |  |  |
|  |  |  |  |  |  |  |  |  |  |  |  |  |  |  |  |  |  |  |  |  |  |  |  |  |  |  |  |  |  |  |  |  |  |  |  |  |  |  |  |  |  |  |
|  |  |  |  |  |  |  |  |  |  |  |  |  |  |  |  |  |  |  |  |  |  |  |  |  |  |  |  |  |  |  |  |  |  |  |  |  |  |  |  |  |  |  |
|  |  |  |  |  |  |  |  |  |  |  |  |  |  |  |  |  |  |  |  |  |  |  |  |  |  |  |  |  |  |  |  |  |  |  |  |  |  |  |  |  |  |  |
|  |  |  |  |  |  |  |  |  |  |  |  |  |  |  |  |  |  |  |  |  |  |  |  |  |  |  |  |  |  |  |  |  |  |  |  |  |  |  |  |  |  |  |
|  |  |  |  |  |  |  |  |  |  |  |  |  |  |  |  |  |  |  |  |  |  |  |  |  |  |  |  |  |  |  |  |  |  |  |  |  |  |  |  |  |  |  |
|  |  |  |  |  |  |  |  |  |  |  |  |  |  |  |  |  |  |  |  |  |  |  |  |  |  |  |  |  |  |  |  |  |  |  |  |  |  |  |  |  |  |  |
|  |  |  |  |  |  |  |  |  |  |  |  |  |  |  |  |  |  |  |  |  |  |  |  |  |  |  |  |  |  |  |  |  |  |  |  |  |  |  |  |  |  |  |
|  |  |  |  |  |  |  |  |  |  |  |  |  |  |  |  |  |  |  |  |  |  |  |  |  |  |  |  |  |  |  |  |  |  |  |  |  |  |  |  |  |  |  |
|  |  |  |  |  |  |  |  |  |  |  |  |  |  |  |  |  |  |  |  |  |  |  |  |  |  |  |  |  |  |  |  |  |  |  |  |  |  |  |  |  |  |  |
|  |  |  |  |  |  |  |  |  |  |  |  |  |  |  |  |  |  |  |  |  |  |  |  |  |  |  |  |  |  |  |  |  |  |  |  |  |  |  |  |  |  |  |
|  |  |  |  |  |  |  |  |  |  |  |  |  |  |  |  |  |  |  |  |  |  |  |  |  |  |  |  |  |  |  |  |  |  |  |  |  |  |  |  |  |  |  |
|  |  |  |  |  |  |  |  |  |  |  |  |  |  |  |  |  |  |  |  |  |  |  |  |  |  |  |  |  |  |  |  |  |  |  |  |  |  |  |  |  |  |  |
|  |  |  |  |  |  |  |  |  |  |  |  |  |  |  |  |  |  |  |  |  |  |  |  |  |  |  |  |  |  |  |  |  |  |  |  |  |  |  |  |  |  |  |
|  |  |  |  |  |  |  |  |  |  |  |  |  |  |  |  |  |  |  |  |  |  |  |  |  |  |  |  |  |  |  |  |  |  |  |  |  |  |  |  |  |  |  |
|  |  |  |  |  |  |  |  |  |  |  |  |  |  |  |  |  |  |  |  |  |  |  |  |  |  |  |  |  |  |  |  |  |  |  |  |  |  |  |  |  |  |  |
|  |  |  |  |  |  |  |  |  |  |  |  |  |  |  |  |  |  |  |  |  |  |  |  |  |  |  |  |  |  |  |  |  |  |  |  |  |  |  |  |  |  |  |
|  |  |  |  |  |  |  |  |  |  |  |  |  |  |  |  |  |  |  |  |  |  |  |  |  |  |  |  |  |  |  |  |  |  |  |  |  |  |  |  |  |  |  |
|  |  |  |  |  |  |  |  |  |  |  |  |  |  |  |  |  |  |  |  |  |  |  |  |  |  |  |  |  |  |  |  |  |  |  |  |  |  |  |  |  |  |  |
|  |  |  |  |  |  |  |  |  |  |  |  |  |  |  |  |  |  |  |  |  |  |  |  |  |  |  |  |  |  |  |  |  |  |  |  |  |  |  |  |  |  |  |
|  |  |  |  |  |  |  |  |  |  |  |  |  |  |  |  |  |  |  |  |  |  |  |  |  |  |  |  |  |  |  |  |  |  |  |  |  |  |  |  |  |  |  |
|  |  |  |  |  |  |  |  |  |  |  |  |  |  |  |  |  |  |  |  |  |  |  |  |  |  |  |  |  |  |  |  |  |  |  |  |  |  |  |  |  |  |  |
|  |  |  |  |  |  |  |  |  |  |  |  |  |  |  |  |  |  |  |  |  |  |  |  |  |  |  |  |  |  |  |  |  |  |  |  |  |  |  |  |  |  |  |
|  |  |  |  |  |  |  |  |  |  |  |  |  |  |  |  |  |  |  |  |  |  |  |  |  |  |  |  |  |  |  |  |  |  |  |  |  |  |  |  |  |  |  |
|  |  |  |  |  |  |  |  |  |  |  |  |  |  |  |  |  |  |  |  |  |  |  |  |  |  |  |  |  |  |  |  |  |  |  |  |  |  |  |  |  |  |  |
|  |  |  |  |  |  |  |  |  |  |  |  |  |  |  |  |  |  |  |  |  |  |  |  |  |  |  |  |  |  |  |  |  |  |  |  |  |  |  |  |  |  |  |
|  |  |  |  |  |  |  |  |  |  |  |  |  |  |  |  |  |  |  |  |  |  |  |  |  |  |  |  |  |  |  |  |  |  |  |  |  |  |  |  |  |  |  |
|  |  |  |  |  |  |  |  |  |  |  |  |  |  |  |  |  |  |  |  |  |  |  |  |  |  |  |  |  |  |  |  |  |  |  |  |  |  |  |  |  |  |  |
|  |  |  |  |  |  |  |  |  |  |  |  |  |  |  |  |  |  |  |  |  |  |  |  |  |  |  |  |  |  |  |  |  |  |  |  |  |  |  |  |  |  |  |
|  |  |  |  |  |  |  |  |  |  |  |  |  |  |  |  |  |  |  |  |  |  |  |  |  |  |  |  |  |  |  |  |  |  |  |  |  |  |  |  |  |  |  |
|  |  |  |  |  |  |  |  |  |  |  |  |  |  |  |  |  |  |  |  |  |  |  |  |  |  |  |  |  |  |  |  |  |  |  |  |  |  |  |  |  |  |  |
|  |  |  |  |  |  |  |  |  |  |  |  |  |  |  |  |  |  |  |  |  |  |  |  |  |  |  |  |  |  |  |  |  |  |  |  |  |  |  |  |  |  |  |
|  |  |  |  |  |  |  |  |  |  |  |  |  |  |  |  |  |  |  |  |  |  |  |  |  |  |  |  |  |  |  |  |  |  |  |  |  |  |  |  |  |  |  |
|  |  |  |  |  |  |  |  |  |  |  |  |  |  |  |  |  |  |  |  |  |  |  |  |  |  |  |  |  |  |  |  |  |  |  |  |  |  |  |  |  |  |  |
|  |  |  |  |  |  |  |  |  |  |  |  |  |  |  |  |  |  |  |  |  |  |  |  |  |  |  |  |  |  |  |  |  |  |  |  |  |  |  |  |  |  |  |
|  |  |  |  |  |  |  |  |  |  |  |  |  |  |  |  |  |  |  |  |  |  |  |  |  |  |  |  |  |  |  |  |  |  |  |  |  |  |  |  |  |  |  |
|  |  |  |  |  |  |  |  |  |  |  |  |  |  |  |  |  |  |  |  |  |  |  |  |  |  |  |  |  |  |  |  |  |  |  |  |  |  |  |  |  |  |  |
|  |  |  |  |  |  |  |  |  |  |  |  |  |  |  |  |  |  |  |  |  |  |  |  |  |  |  |  |  |  |  |  |  |  |  |  |  |  |  |  |  |  |  |
|  |  |  |  |  |  |  |  |  |  |  |  |  |  |  |  |  |  |  |  |  |  |  |  |  |  |  |  |  |  |  |  |  |  |  |  |  |  |  |  |  |  |  |
|  |  |  |  |  |  |  |  |  |  |  |  |  |  |  |  |  |  |  |  |  |  |  |  |  |  |  |  |  |  |  |  |  |  |  |  |  |  |  |  |  |  |  |
|  |  |  |  |  |  |  |  |  |  |  |  |  |  |  |  |  |  |  |  |  |  |  |  |  |  |  |  |  |  |  |  |  |  |  |  |  |  |  |  |  |  |  |
|  |  |  |  |  |  |  |  |  |  |  |  |  |  |  |  |  |  |  |  |  |  |  |  |  |  |  |  |  |  |  |  |  |  |  |  |  |  |  |  |  |  |  |
|  |  |  |  |  |  |  |  |  |  |  |  |  |  |  |  |  |  |  |  |  |  |  |  |  |  |  |  |  |  |  |  |  |  |  |  |  |  |  |  |  |  |  |
|  |  |  |  |  |  |  |  |  |  |  |  |  |  |  |  |  |  |  |  |  |  |  |  |  |  |  |  |  |  |  |  |  |  |  |  |  |  |  |  |  |  |  |
|  |  |  |  |  |  |  |  |  |  |  |  |  |  |  |  |  |  |  |  |  |  |  |  |  |  |  |  |  |  |  |  |  |  |  |  |  |  |  |  |  |  |  |
|  |  |  |  |  |  |  |  |  |  |  |  |  |  |  |  |  |  |  |  |  |  |  |  |  |  |  |  |  |  |  |  |  |  |  |  |  |  |  |  |  |  |  |
|  |  |  |  |  |  |  |  |  |  |  |  |  |  |  |  |  |  |  |  |  |  |  |  |  |  |  |  |  |  |  |  |  |  |  |  |  |  |  |  |  |  |  |
|  |  |  |  |  |  |  |  |  |  |  |  |  |  |  |  |  |  |  |  |  |  |  |  |  |  |  |  |  |  |  |  |  |  |  |  |  |  |  |  |  |  |  |
|  |  |  |  |  |  |  |  |  |  |  |  |  |  |  |  |  |  |  |  |  |  |  |  |  |  |  |  |  |  |  |  |  |  |  |  |  |  |  |  |  |  |  |
|  |  |  |  |  |  |  |  |  |  |  |  |  |  |  |  |  |  |  |  |  |  |  |  |  |  |  |  |  |  |  |  |  |  |  |  |  |  |  |  |  |  |  |
|  |  |  |  |  |  |  |  |  |  |  |  |  |  |  |  |  |  |  |  |  |  |  |  |  |  |  |  |  |  |  |  |  |  |  |  |  |  |  |  |  |  |  |
|  |  |  |  |  |  |  |  |  |  |  |  |  |  |  |  |  |  |  |  |  |  |  |  |  |  |  |  |  |  |  |  |  |  |  |  |  |  |  |  |  |  |  |
|  |  |  |  |  |  |  |  |  |  |  |  |  |  |  |  |  |  |  |  |  |  |  |  |  |  |  |  |  |  |  |  |  |  |  |  |  |  |  |  |  |  |  |
|  |  |  |  |  |  |  |  |  |  |  |  |  |  |  |  |  |  |  |  |  |  |  |  |  |  |  |  |  |  |  |  |  |  |  |  |  |  |  |  |  |  |  |
|  |  |  |  |  |  |  |  |  |  |  |  |  |  |  |  |  |  |  |  |  |  |  |  |  |  |  |  |  |  |  |  |  |  |  |  |  |  |  |  |  |  |  |
|  |  |  |  |  |  |  |  |  |  |  |  |  |  |  |  |  |  |  |  |  |  |  |  |  |  |  |  |  |  |  |  |  |  |  |  |  |  |  |  |  |  |  |
|  |  |  |  |  |  |  |  |  |  |  |  |  |  |  |  |  |  |  |  |  |  |  |  |  |  |  |  |  |  |  |  |  |  |  |  |  |  |  |  |  |  |  |
|  |  |  |  |  |  |  |  |  |  |  |  |  |  |  |  |  |  |  |  |  |  |  |  |  |  |  |  |  |  |  |  |  |  |  |  |  |  |  |  |  |  |  |
|  |  |  |  |  |  |  |  |  |  |  |  |  |  |  |  |  |  |  |  |  |  |  |  |  |  |  |  |  |  |  |  |  |  |  |  |  |  |  |  |  |  |  |
|  |  |  |  |  |  |  |  |  |  |  |  |  |  |  |  |  |  |  |  |  |  |  |  |  |  |  |  |  |  |  |  |  |  |  |  |  |  |  |  |  |  |  |
|  |  |  |  |  |  |  |  |  |  |  |  |  |  |  |  |  |  |  |  |  |  |  |  |  |  |  |  |  |  |  |  |  |  |  |  |  |  |  |  |  |  |  |
|  |  |  |  |  |  |  |  |  |  |  |  |  |  |  |  |  |  |  |  |  |  |  |  |  |  |  |  |  |  |  |  |  |  |  |  |  |  |  |  |  |  |  |
|  |  |  |  |  |  |  |  |  |  |  |  |  |  |  |  |  |  |  |  |  |  |  |  |  |  |  |  |  |  |  |  |  |  |  |  |  |  |  |  |  |  |  |
|  |  |  |  |  |  |  |  |  |  |  |  |  |  |  |  |  |  |  |  |  |  |  |  |  |  |  |  |  |  |  |  |  |  |  |  |  |  |  |  |  |  |  |
|  |  |  |  |  |  |  |  |  |  |  |  |  |  |  |  |  |  |  |  |  |  |  |  |  |  |  |  |  |  |  |  |  |  |  |  |  |  |  |  |  |  |  |
|  |  |  |  |  |  |  |  |  |  |  |  |  |  |  |  |  |  |  |  |  |  |  |  |  |  |  |  |  |  |  |  |  |  |  |  |  |  |  |  |  |  |  |
|  |  |  |  |  |  |  |  |  |  |  |  |  |  |  |  |  |  |  |  |  |  |  |  |  |  |  |  |  |  |  |  |  |  |  |  |  |  |  |  |  |  |  |
|  |  |  |  |  |  |  |  |  |  |  |  |  |  |  |  |  |  |  |  |  |  |  |  |  |  |  |  |  |  |  |  |  |  |  |  |  |  |  |  |  |  |  |
|  |  |  |  |  |  |  |  |  |  |  |  |  |  |  |  |  |  |  |  |  |  |  |  |  |  |  |  |  |  |  |  |  |  |  |  |  |  |  |  |  |  |  |
|  |  |  |  |  |  |  |  |  |  |  |  |  |  |  |  |  |  |  |  |  |  |  |  |  |  |  |  |  |  |  |  |  |  |  |  |  |  |  |  |  |  |  |
|  |  |  |  |  |  |  |  |  |  |  |  |  |  |  |  |  |  |  |  |  |  |  |  |  |  |  |  |  |  |  |  |  |  |  |  |  |  |  |  |  |  |  |
|  |  |  |  |  |  |  |  |  |  |  |  |  |  |  |  |  |  |  |  |  |  |  |  |  |  |  |  |  |  |  |  |  |  |  |  |  |  |  |  |  |  |  |
|  |  |  |  |  |  |  |  |  |  |  |  |  |  |  |  |  |  |  |  |  |  |  |  |  |  |  |  |  |  |  |  |  |  |  |  |  |  |  |  |  |  |  |
|  |  |  |  |  |  |  |  |  |  |  |  |  |  |  |  |  |  |  |  |  |  |  |  |  |  |  |  |  |  |  |  |  |  |  |  |  |  |  |  |  |  |  |
|  |  |  |  |  |  |  |  |  |  |  |  |  |  |  |  |  |  |  |  |  |  |  |  |  |  |  |  |  |  |  |  |  |  |  |  |  |  |  |  |  |  |  |
|  |  |  |  |  |  |  |  |  |  |  |  |  |  |  |  |  |  |  |  |  |  |  |  |  |  |  |  |  |  |  |  |  |  |  |  |  |  |  |  |  |  |  |
|  |  |  |  |  |  |  |  |  |  |  |  |  |  |  |  |  |  |  |  |  |  |  |  |  |  |  |  |  |  |  |  |  |  |  |  |  |  |  |  |  |  |  |
|  |  |  |  |  |  |  |  |  |  |  |  |  |  |  |  |  |  |  |  |  |  |  |  |  |  |  |  |  |  |  |  |  |  |  |  |  |  |  |  |  |  |  |
|  |  |  |  |  |  |  |  |  |  |  |  |  |  |  |  |  |  |  |  |  |  |  |  |  |  |  |  |  |  |  |  |  |  |  |  |  |  |  |  |  |  |  |
|  |  |  |  |  |  |  |  |  |  |  |  |  |  |  |  |  |  |  |  |  |  |  |  |  |  |  |  |  |  |  |  |  |  |  |  |  |  |  |  |  |  |  |
|  |  |  |  |  |  |  |  |  |  |  |  |  |  |  |  |  |  |  |  |  |  |  |  |  |  |  |  |  |  |  |  |  |  |  |  |  |  |  |  |  |  |  |
|  |  |  |  |  |  |  |  |  |  |  |  |  |  |  |  |  |  |  |  |  |  |  |  |  |  |  |  |  |  |  |  |  |  |  |  |  |  |  |  |  |  |  |
|  |  |  |  |  |  |  |  |  |  |  |  |  |  |  |  |  |  |  |  |  |  |  |  |  |  |  |  |  |  |  |  |  |  |  |  |  |  |  |  |  |  |  |
|  |  |  |  |  |  |  |  |  |  |  |  |  |  |  |  |  |  |  |  |  |  |  |  |  |  |  |  |  |  |  |  |  |  |  |  |  |  |  |  |  |  |  |
|  |  |  |  |  |  |  |  |  |  |  |  |  |  |  |  |  |  |  |  |  |  |  |  |  |  |  |  |  |  |  |  |  |  |  |  |  |  |  |  |  |  |  |
|  |  |  |  |  |  |  |  |  |  |  |  |  |  |  |  |  |  |  |  |  |  |  |  |  |  |  |  |  |  |  |  |  |  |  |  |  |  |  |  |  |  |  |
|  |  |  |  |  |  |  |  |  |  |  |  |  |  |  |  |  |  |  |  |  |  |  |  |  |  |  |  |  |  |  |  |  |  |  |  |  |  |  |  |  |  |  |
|  |  |  |  |  |  |  |  |  |  |  |  |  |  |  |  |  |  |  |  |  |  |  |  |  |  |  |  |  |  |  |  |  |  |  |  |  |  |  |  |  |  |  |
|  |  |  |  |  |  |  |  |  |  |  |  |  |  |  |  |  |  |  |  |  |  |  |  |  |  |  |  |  |  |  |  |  |  |  |  |  |  |  |  |  |  |  |
|  |  |  |  |  |  |  |  |  |  |  |  |  |  |  |  |  |  |  |  |  |  |  |  |  |  |  |  |  |  |  |  |  |  |  |  |  |  |  |  |  |  |  |
|  |  |  |  |  |  |  |  |  |  |  |  |  |  |  |  |  |  |  |  |  |  |  |  |  |  |  |  |  |  |  |  |  |  |  |  |  |  |  |  |  |  |  |
|  |  |  |  |  |  |  |  |  |  |  |  |  |  |  |  |  |  |  |  |  |  |  |  |  |  |  |  |  |  |  |  |  |  |  |  |  |  |  |  |  |  |  |
|  |  |  |  |  |  |  |  |  |  |  |  |  |  |  |  |  |  |  |  |  |  |  |  |  |  |  |  |  |  |  |  |  |  |  |  |  |  |  |  |  |  |  |
|  |  |  |  |  |  |  |  |  |  |  |  |  |  |  |  |  |  |  |  |  |  |  |  |  |  |  |  |  |  |  |  |  |  |  |  |  |  |  |  |  |  |  |
|  |  |  |  |  |  |  |  |  |  |  |  |  |  |  |  |  |  |  |  |  |  |  |  |  |  |  |  |  |  |  |  |  |  |  |  |  |  |  |  |  |  |  |
|  |  |  |  |  |  |  |  |  |  |  |  |  |  |  |  |  |  |  |  |  |  |  |  |  |  |  |  |  |  |  |  |  |  |  |  |  |  |  |  |  |  |  |
|  |  |  |  |  |  |  |  |  |  |  |  |  |  |  |  |  |  |  |  |  |  |  |  |  |  |  |  |  |  |  |  |  |  |  |  |  |  |  |  |  |  |  |
|  |  |  |  |  |  |  |  |  |  |  |  |  |  |  |  |  |  |  |  |  |  |  |  |  |  |  |  |  |  |  |  |  |  |  |  |  |  |  |  |  |  |  |
|  |  |  |  |  |  |  |  |  |  |  |  |  |  |  |  |  |  |  |  |  |  |  |  |  |  |  |  |  |  |  |  |  |  |  |  |  |  |  |  |  |  |  |
|  |  |  |  |  |  |  |  |  |  |  |  |  |  |  |  |  |  |  |  |  |  |  |  |  |  |  |  |  |  |  |  |  |  |  |  |  |  |  |  |  |  |  |
|  |  |  |  |  |  |  |  |  |  |  |  |  |  |  |  |  |  |  |  |  |  |  |  |  |  |  |  |  |  |  |  |  |  |  |  |  |  |  |  |  |  |  |
|  |  |  |  |  |  |  |  |  |  |  |  |  |  |  |  |  |  |  |  |  |  |  |  |  |  |  |  |  |  |  |  |  |  |  |  |  |  |  |  |  |  |  |
|  |  |  |  |  |  |  |  |  |  |  |  |  |  |  |  |  |  |  |  |  |  |  |  |  |  |  |  |  |  |  |  |  |  |  |  |  |  |  |  |  |  |  |
|  |  |  |  |  |  |  |  |  |  |  |  |  |  |  |  |  |  |  |  |  |  |  |  |  |  |  |  |  |  |  |  |  |  |  |  |  |  |  |  |  |  |  |
|  |  |  |  |  |  |  |  |  |  |  |  |  |  |  |  |  |  |  |  |  |  |  |  |  |  |  |  |  |  |  |  |  |  |  |  |  |  |  |  |  |  |  |
|  |  |  |  |  |  |  |  |  |  |  |  |  |  |  |  |  |  |  |  |  |  |  |  |  |  |  |  |  |  |  |  |  |  |  |  |  |  |  |  |  |  |  |
|  |  |  |  |  |  |  |  |  |  |  |  |  |  |  |  |  |  |  |  |  |  |  |  |  |  |  |  |  |  |  |  |  |  |  |  |  |  |  |  |  |  |  |
|  |  |  |  |  |  |  |  |  |  |  |  |  |  |  |  |  |  |  |  |  |  |  |  |  |  |  |  |  |  |  |  |  |  |  |  |  |  |  |  |  |  |  |
|  |  |  |  |  |  |  |  |  |  |  |  |  |  |  |  |  |  |  |  |  |  |  |  |  |  |  |  |  |  |  |  |  |  |  |  |  |  |  |  |  |  |  |
|  |  |  |  |  |  |  |  |  |  |  |  |  |  |  |  |  |  |  |  |  |  |  |  |  |  |  |  |  |  |  |  |  |  |  |  |  |  |  |  |  |  |  |
|  |  |  |  |  |  |  |  |  |  |  |  |  |  |  |  |  |  |  |  |  |  |  |  |  |  |  |  |  |  |  |  |  |  |  |  |  |  |  |  |  |  |  |
|  |  |  |  |  |  |  |  |  |  |  |  |  |  |  |  |  |  |  |  |  |  |  |  |  |  |  |  |  |  |  |  |  |  |  |  |  |  |  |  |  |  |  |
|  |  |  |  |  |  |  |  |  |  |  |  |  |  |  |  |  |  |  |  |  |  |  |  |  |  |  |  |  |  |  |  |  |  |  |  |  |  |  |  |  |  |  |
|  |  |  |  |  |  |  |  |  |  |  |  |  |  |  |  |  |  |  |  |  |  |  |  |  |  |  |  |  |  |  |  |  |  |  |  |  |  |  |  |  |  |  |
|  |  |  |  |  |  |  |  |  |  |  |  |  |  |  |  |  |  |  |  |  |  |  |  |  |  |  |  |  |  |  |  |  |  |  |  |  |  |  |  |  |  |  |
|  |  |  |  |  |  |  |  |  |  |  |  |  |  |  |  |  |  |  |  |  |  |  |  |  |  |  |  |  |  |  |  |  |  |  |  |  |  |  |  |  |  |  |
|  |  |  |  |  |  |  |  |  |  |  |  |  |  |  |  |  |  |  |  |  |  |  |  |  |  |  |  |  |  |  |  |  |  |  |  |  |  |  |  |  |  |  |
|  |  |  |  |  |  |  |  |  |  |  |  |  |  |  |  |  |  |  |  |  |  |  |  |  |  |  |  |  |  |  |  |  |  |  |  |  |  |  |  |  |  |  |
|  |  |  |  |  |  |  |  |  |  |  |  |  |  |  |  |  |  |  |  |  |  |  |  |  |  |  |  |  |  |  |  |  |  |  |  |  |  |  |  |  |  |  |
|  |  |  |  |  |  |  |  |  |  |  |  |  |  |  |  |  |  |  |  |  |  |  |  |  |  |  |  |  |  |  |  |  |  |  |  |  |  |  |  |  |  |  |
|  |  |  |  |  |  |  |  |  |  |  |  |  |  |  |  |  |  |  |  |  |  |  |  |  |  |  |  |  |  |  |  |  |  |  |  |  |  |  |  |  |  |  |
|  |  |  |  |  |  |  |  |  |  |  |  |  |  |  |  |  |  |  |  |  |  |  |  |  |  |  |  |  |  |  |  |  |  |  |  |  |  |  |  |  |  |  |
|  |  |  |  |  |  |  |  |  |  |  |  |  |  |  |  |  |  |  |  |  |  |  |  |  |  |  |  |  |  |  |  |  |  |  |  |  |  |  |  |  |  |  |
|  |  |  |  |  |  |  |  |  |  |  |  |  |  |  |  |  |  |  |  |  |  |  |  |  |  |  |  |  |  |  |  |  |  |  |  |  |  |  |  |  |  |  |
|  |  |  |  |  |  |  |  |  |  |  |  |  |  |  |  |  |  |  |  |  |  |  |  |  |  |  |  |  |  |  |  |  |  |  |  |  |  |  |  |  |  |  |
|  |  |  |  |  |  |  |  |  |  |  |  |  |  |  |  |  |  |  |  |  |  |  |  |  |  |  |  |  |  |  |  |  |  |  |  |  |  |  |  |  |  |  |
|  |  |  |  |  |  |  |  |  |  |  |  |  |  |  |  |  |  |  |  |  |  |  |  |  |  |  |  |  |  |  |  |  |  |  |  |  |  |  |  |  |  |  |
|  |  |  |  |  |  |  |  |  |  |  |  |  |  |  |  |  |  |  |  |  |  |  |  |  |  |  |  |  |  |  |  |  |  |  |  |  |  |  |  |  |  |  |
|  |  |  |  |  |  |  |  |  |  |  |  |  |  |  |  |  |  |  |  |  |  |  |  |  |  |  |  |  |  |  |  |  |  |  |  |  |  |  |  |  |  |  |
|  |  |  |  |  |  |  |  |  |  |  |  |  |  |  |  |  |  |  |  |  |  |  |  |  |  |  |  |  |  |  |  |  |  |  |  |  |  |  |  |  |  |  |
|  |  |  |  |  |  |  |  |  |  |  |  |  |  |  |  |  |  |  |  |  |  |  |  |  |  |  |  |  |  |  |  |  |  |  |  |  |  |  |  |  |  |  |
|  |  |  |  |  |  |  |  |  |  |  |  |  |  |  |  |  |  |  |  |  |  |  |  |  |  |  |  |  |  |  |  |  |  |  |  |  |  |  |  |  |  |  |
|  |  |  |  |  |  |  |  |  |  |  |  |  |  |  |  |  |  |  |  |  |  |  |  |  |  |  |  |  |  |  |  |  |  |  |  |  |  |  |  |  |  |  |
|  |  |  |  |  |  |  |  |  |  |  |  |  |  |  |  |  |  |  |  |  |  |  |  |  |  |  |  |  |  |  |  |  |  |  |  |  |  |  |  |  |  |  |
|  |  |  |  |  |  |  |  |  |  |  |  |  |  |  |  |  |  |  |  |  |  |  |  |  |  |  |  |  |  |  |  |  |  |  |  |  |  |  |  |  |  |  |
|  |  |  |  |  |  |  |  |  |  |  |  |  |  |  |  |  |  |  |  |  |  |  |  |  |  |  |  |  |  |  |  |  |  |  |  |  |  |  |  |  |  |  |
|  |  |  |  |  |  |  |  |  |  |  |  |  |  |  |  |  |  |  |  |  |  |  |  |  |  |  |  |  |  |  |  |  |  |  |  |  |  |  |  |  |  |  |
|  |  |  |  |  |  |  |  |  |  |  |  |  |  |  |  |  |  |  |  |  |  |  |  |  |  |  |  |  |  |  |  |  |  |  |  |  |  |  |  |  |  |  |
|  |  |  |  |  |  |  |  |  |  |  |  |  |  |  |  |  |  |  |  |  |  |  |  |  |  |  |  |  |  |  |  |  |  |  |  |  |  |  |  |  |  |  |
|  |  |  |  |  |  |  |  |  |  |  |  |  |  |  |  |  |  |  |  |  |  |  |  |  |  |  |  |  |  |  |  |  |  |  |  |  |  |  |  |  |  |  |
|  |  |  |  |  |  |  |  |  |  |  |  |  |  |  |  |  |  |  |  |  |  |  |  |  |  |  |  |  |  |  |  |  |  |  |  |  |  |  |  |  |  |  |
|  |  |  |  |  |  |  |  |  |  |  |  |  |  |  |  |  |  |  |  |  |  |  |  |  |  |  |  |  |  |  |  |  |  |  |  |  |  |  |  |  |  |  |
|  |  |  |  |  |  |  |  |  |  |  |  |  |  |  |  |  |  |  |  |  |  |  |  |  |  |  |  |  |  |  |  |  |  |  |  |  |  |  |  |  |  |  |
|  |  |  |  |  |  |  |  |  |  |  |  |  |  |  |  |  |  |  |  |  |  |  |  |  |  |  |  |  |  |  |  |  |  |  |  |  |  |  |  |  |  |  |
|  |  |  |  |  |  |  |  |  |  |  |  |  |  |  |  |  |  |  |  |  |  |  |  |  |  |  |  |  |  |  |  |  |  |  |  |  |  |  |  |  |  |  |
|  |  |  |  |  |  |  |  |  |  |  |  |  |  |  |  |  |  |  |  |  |  |  |  |  |  |  |  |  |  |  |  |  |  |  |  |  |  |  |  |  |  |  |
|  |  |  |  |  |  |  |  |  |  |  |  |  |  |  |  |  |  |  |  |  |  |  |  |  |  |  |  |  |  |  |  |  |  |  |  |  |  |  |  |  |  |  |
|  |  |  |  |  |  |  |  |  |  |  |  |  |  |  |  |  |  |  |  |  |  |  |  |  |  |  |  |  |  |  |  |  |  |  |  |  |  |  |  |  |  |  |
|  |  |  |  |  |  |  |  |  |  |  |  |  |  |  |  |  |  |  |  |  |  |  |  |  |  |  |  |  |  |  |  |  |  |  |  |  |  |  |  |  |  |  |
|  |  |  |  |  |  |  |  |  |  |  |  |  |  |  |  |  |  |  |  |  |  |  |  |  |  |  |  |  |  |  |  |  |  |  |  |  |  |  |  |  |  |  |
|  |  |  |  |  |  |  |  |  |  |  |  |  |  |  |  |  |  |  |  |  |  |  |  |  |  |  |  |  |  |  |  |  |  |  |  |  |  |  |  |  |  |  |
|  |  |  |  |  |  |  |  |  |  |  |  |  |  |  |  |  |  |  |  |  |  |  |  |  |  |  |  |  |  |  |  |  |  |  |  |  |  |  |  |  |  |  |
|  |  |  |  |  |  |  |  |  |  |  |  |  |  |  |  |  |  |  |  |  |  |  |  |  |  |  |  |  |  |  |  |  |  |  |  |  |  |  |  |  |  |  |
|  |  |  |  |  |  |  |  |  |  |  |  |  |  |  |  |  |  |  |  |  |  |  |  |  |  |  |  |  |  |  |  |  |  |  |  |  |  |  |  |  |  |  |
|  |  |  |  |  |  |  |  |  |  |  |  |  |  |  |  |  |  |  |  |  |  |  |  |  |  |  |  |  |  |  |  |  |  |  |  |  |  |  |  |  |  |  |
|  |  |  |  |  |  |  |  |  |  |  |  |  |  |  |  |  |  |  |  |  |  |  |  |  |  |  |  |  |  |  |  |  |  |  |  |  |  |  |  |  |  |  |
|  |  |  |  |  |  |  |  |  |  |  |  |  |  |  |  |  |  |  |  |  |  |  |  |  |  |  |  |  |  |  |  |  |  |  |  |  |  |  |  |  |  |  |
|  |  |  |  |  |  |  |  |  |  |  |  |  |  |  |  |  |  |  |  |  |  |  |  |  |  |  |  |  |  |  |  |  |  |  |  |  |  |  |  |  |  |  |
|  |  |  |  |  |  |  |  |  |  |  |  |  |  |  |  |  |  |  |  |  |  |  |  |  |  |  |  |  |  |  |  |  |  |  |  |  |  |  |  |  |  |  |
|  |  |  |  |  |  |  |  |  |  |  |  |  |  |  |  |  |  |  |  |  |  |  |  |  |  |  |  |  |  |  |  |  |  |  |  |  |  |  |  |  |  |  |
|  |  |  |  |  |  |  |  |  |  |  |  |  |  |  |  |  |  |  |  |  |  |  |  |  |  |  |  |  |  |  |  |  |  |  |  |  |  |  |  |  |  |  |
|  |  |  |  |  |  |  |  |  |  |  |  |  |  |  |  |  |  |  |  |  |  |  |  |  |  |  |  |  |  |  |  |  |  |  |  |  |  |  |  |  |  |  |
|  |  |  |  |  |  |  |  |  |  |  |  |  |  |  |  |  |  |  |  |  |  |  |  |  |  |  |  |  |  |  |  |  |  |  |  |  |  |  |  |  |  |  |
|  |  |  |  |  |  |  |  |  |  |  |  |  |  |  |  |  |  |  |  |  |  |  |  |  |  |  |  |  |  |  |  |  |  |  |  |  |  |  |  |  |  |  |
|  |  |  |  |  |  |  |  |  |  |  |  |  |  |  |  |  |  |  |  |  |  |  |  |  |  |  |  |  |  |  |  |  |  |  |  |  |  |  |  |  |  |  |
|  |  |  |  |  |  |  |  |  |  |  |  |  |  |  |  |  |  |  |  |  |  |  |  |  |  |  |  |  |  |  |  |  |  |  |  |  |  |  |  |  |  |  |
|  |  |  |  |  |  |  |  |  |  |  |  |  |  |  |  |  |  |  |  |  |  |  |  |  |  |  |  |  |  |  |  |  |  |  |  |  |  |  |  |  |  |  |
|  |  |  |  |  |  |  |  |  |  |  |  |  |  |  |  |  |  |  |  |  |  |  |  |  |  |  |  |  |  |  |  |  |  |  |  |  |  |  |  |  |  |  |
|  |  |  |  |  |  |  |  |  |  |  |  |  |  |  |  |  |  |  |  |  |  |  |  |  |  |  |  |  |  |  |  |  |  |  |  |  |  |  |  |  |  |  |
|  |  |  |  |  |  |  |  |  |  |  |  |  |  |  |  |  |  |  |  |  |  |  |  |  |  |  |  |  |  |  |  |  |  |  |  |  |  |  |  |  |  |  |
|  |  |  |  |  |  |  |  |  |  |  |  |  |  |  |  |  |  |  |  |  |  |  |  |  |  |  |  |  |  |  |  |  |  |  |  |  |  |  |  |  |  |  |
|  |  |  |  |  |  |  |  |  |  |  |  |  |  |  |  |  |  |  |  |  |  |  |  |  |  |  |  |  |  |  |  |  |  |  |  |  |  |  |  |  |  |  |
|  |  |  |  |  |  |  |  |  |  |  |  |  |  |  |  |  |  |  |  |  |  |  |  |  |  |  |  |  |  |  |  |  |  |  |  |  |  |  |  |  |  |  |
|  |  |  |  |  |  |  |  |  |  |  |  |  |  |  |  |  |  |  |  |  |  |  |  |  |  |  |  |  |  |  |  |  |  |  |  |  |  |  |  |  |  |  |
|  |  |  |  |  |  |  |  |  |  |  |  |  |  |  |  |  |  |  |  |  |  |  |  |  |  |  |  |  |  |  |  |  |  |  |  |  |  |  |  |  |  |  |
|  |  |  |  |  |  |  |  |  |  |  |  |  |  |  |  |  |  |  |  |  |  |  |  |  |  |  |  |  |  |  |  |  |  |  |  |  |  |  |  |  |  |  |
|  |  |  |  |  |  |  |  |  |  |  |  |  |  |  |  |  |  |  |  |  |  |  |  |  |  |  |  |  |  |  |  |  |  |  |  |  |  |  |  |  |  |  |
|  |  |  |  |  |  |  |  |  |  |  |  |  |  |  |  |  |  |  |  |  |  |  |  |  |  |  |  |  |  |  |  |  |  |  |  |  |  |  |  |  |  |  |
|  |  |  |  |  |  |  |  |  |  |  |  |  |  |  |  |  |  |  |  |  |  |  |  |  |  |  |  |  |  |  |  |  |  |  |  |  |  |  |  |  |  |  |
|  |  |  |  |  |  |  |  |  |  |  |  |  |  |  |  |  |  |  |  |  |  |  |  |  |  |  |  |  |  |  |  |  |  |  |  |  |  |  |  |  |  |  |
|  |  |  |  |  |  |  |  |  |  |  |  |  |  |  |  |  |  |  |  |  |  |  |  |  |  |  |  |  |  |  |  |  |  |  |  |  |  |  |  |  |  |  |
|  |  |  |  |  |  |  |  |  |  |  |  |  |  |  |  |  |  |  |  |  |  |  |  |  |  |  |  |  |  |  |  |  |  |  |  |  |  |  |  |  |  |  |
|  |  |  |  |  |  |  |  |  |  |  |  |  |  |  |  |  |  |  |  |  |  |  |  |  |  |  |  |  |  |  |  |  |  |  |  |  |  |  |  |  |  |  |
|  |  |  |  |  |  |  |  |  |  |  |  |  |  |  |  |  |  |  |  |  |  |  |  |  |  |  |  |  |  |  |  |  |  |  |  |  |  |  |  |  |  |  |
|  |  |  |  |  |  |  |  |  |  |  |  |  |  |  |  |  |  |  |  |  |  |  |  |  |  |  |  |  |  |  |  |  |  |  |  |  |  |  |  |  |  |  |
|  |  |  |  |  |  |  |  |  |  |  |  |  |  |  |  |  |  |  |  |  |  |  |  |  |  |  |  |  |  |  |  |  |  |  |  |  |  |  |  |  |  |  |
|  |  |  |  |  |  |  |  |  |  |  |  |  |  |  |  |  |  |  |  |  |  |  |  |  |  |  |  |  |  |  |  |  |  |  |  |  |  |  |  |  |  |  |
|  |  |  |  |  |  |  |  |  |  |  |  |  |  |  |  |  |  |  |  |  |  |  |  |  |  |  |  |  |  |  |  |  |  |  |  |  |  |  |  |  |  |  |
|  |  |  |  |  |  |  |  |  |  |  |  |  |  |  |  |  |  |  |  |  |  |  |  |  |  |  |  |  |  |  |  |  |  |  |  |  |  |  |  |  |  |  |
|  |  |  |  |  |  |  |  |  |  |  |  |  |  |  |  |  |  |  |  |  |  |  |  |  |  |  |  |  |  |  |  |  |  |  |  |  |  |  |  |  |  |  |
|  |  |  |  |  |  |  |  |  |  |  |  |  |  |  |  |  |  |  |  |  |  |  |  |  |  |  |  |  |  |  |  |  |  |  |  |  |  |  |  |  |  |  |
|  |  |  |  |  |  |  |  |  |  |  |  |  |  |  |  |  |  |  |  |  |  |  |  |  |  |  |  |  |  |  |  |  |  |  |  |  |  |  |  |  |  |  |
|  |  |  |  |  |  |  |  |  |  |  |  |  |  |  |  |  |  |  |  |  |  |  |  |  |  |  |  |  |  |  |  |  |  |  |  |  |  |  |  |  |  |  |
|  |  |  |  |  |  |  |  |  |  |  |  |  |  |  |  |  |  |  |  |  |  |  |  |  |  |  |  |  |  |  |  |  |  |  |  |  |  |  |  |  |  |  |
|  |  |  |  |  |  |  |  |  |  |  |  |  |  |  |  |  |  |  |  |  |  |  |  |  |  |  |  |  |  |  |  |  |  |  |  |  |  |  |  |  |  |  |
|  |  |  |  |  |  |  |  |  |  |  |  |  |  |  |  |  |  |  |  |  |  |  |  |  |  |  |  |  |  |  |  |  |  |  |  |  |  |  |  |  |  |  |
|  |  |  |  |  |  |  |  |  |  |  |  |  |  |  |  |  |  |  |  |  |  |  |  |  |  |  |  |  |  |  |  |  |  |  |  |  |  |  |  |  |  |  |
|  |  |  |  |  |  |  |  |  |  |  |  |  |  |  |  |  |  |  |  |  |  |  |  |  |  |  |  |  |  |  |  |  |  |  |  |  |  |  |  |  |  |  |
|  |  |  |  |  |  |  |  |  |  |  |  |  |  |  |  |  |  |  |  |  |  |  |  |  |  |  |  |  |  |  |  |  |  |  |  |  |  |  |  |  |  |  |
|  |  |  |  |  |  |  |  |  |  |  |  |  |  |  |  |  |  |  |  |  |  |  |  |  |  |  |  |  |  |  |  |  |  |  |  |  |  |  |  |  |  |  |
|  |  |  |  |  |  |  |  |  |  |  |  |  |  |  |  |  |  |  |  |  |  |  |  |  |  |  |  |  |  |  |  |  |  |  |  |  |  |  |  |  |  |  |
|  |  |  |  |  |  |  |  |  |  |  |  |  |  |  |  |  |  |  |  |  |  |  |  |  |  |  |  |  |  |  |  |  |  |  |  |  |  |  |  |  |  |  |
|  |  |  |  |  |  |  |  |  |  |  |  |  |  |  |  |  |  |  |  |  |  |  |  |  |  |  |  |  |  |  |  |  |  |  |  |  |  |  |  |  |  |  |
|  |  |  |  |  |  |  |  |  |  |  |  |  |  |  |  |  |  |  |  |  |  |  |  |  |  |  |  |  |  |  |  |  |  |  |  |  |  |  |  |  |  |  |
|  |  |  |  |  |  |  |  |  |  |  |  |  |  |  |  |  |  |  |  |  |  |  |  |  |  |  |  |  |  |  |  |  |  |  |  |  |  |  |  |  |  |  |
|  |  |  |  |  |  |  |  |  |  |  |  |  |  |  |  |  |  |  |  |  |  |  |  |  |  |  |  |  |  |  |  |  |  |  |  |  |  |  |  |  |  |  |
|  |  |  |  |  |  |  |  |  |  |  |  |  |  |  |  |  |  |  |  |  |  |  |  |  |  |  |  |  |  |  |  |  |  |  |  |  |  |  |  |  |  |  |
|  |  |  |  |  |  |  |  |  |  |  |  |  |  |  |  |  |  |  |  |  |  |  |  |  |  |  |  |  |  |  |  |  |  |  |  |  |  |  |  |  |  |  |
|  |  |  |  |  |  |  |  |  |  |  |  |  |  |  |  |  |  |  |  |  |  |  |  |  |  |  |  |  |  |  |  |  |  |  |  |  |  |  |  |  |  |  |
|  |  |  |  |  |  |  |  |  |  |  |  |  |  |  |  |  |  |  |  |  |  |  |  |  |  |  |  |  |  |  |  |  |  |  |  |  |  |  |  |  |  |  |
|  |  |  |  |  |  |  |  |  |  |  |  |  |  |  |  |  |  |  |  |  |  |  |  |  |  |  |  |  |  |  |  |  |  |  |  |  |  |  |  |  |  |  |
|  |  |  |  |  |  |  |  |  |  |  |  |  |  |  |  |  |  |  |  |  |  |  |  |  |  |  |  |  |  |  |  |  |  |  |  |  |  |  |  |  |  |  |
|  |  |  |  |  |  |  |  |  |  |  |  |  |  |  |  |  |  |  |  |  |  |  |  |  |  |  |  |  |  |  |  |  |  |  |  |  |  |  |  |  |  |  |
|  |  |  |  |  |  |  |  |  |  |  |  |  |  |  |  |  |  |  |  |  |  |  |  |  |  |  |  |  |  |  |  |  |  |  |  |  |  |  |  |  |  |  |
|  |  |  |  |  |  |  |  |  |  |  |  |  |  |  |  |  |  |  |  |  |  |  |  |  |  |  |  |  |  |  |  |  |  |  |  |  |  |  |  |  |  |  |
|  |  |  |  |  |  |  |  |  |  |  |  |  |  |  |  |  |  |  |  |  |  |  |  |  |  |  |  |  |  |  |  |  |  |  |  |  |  |  |  |  |  |  |
|  |  |  |  |  |  |  |  |  |  |  |  |  |  |  |  |  |  |  |  |  |  |  |  |  |  |  |  |  |  |  |  |  |  |  |  |  |  |  |  |  |  |  |
|  |  |  |  |  |  |  |  |  |  |  |  |  |  |  |  |  |  |  |  |  |  |  |  |  |  |  |  |  |  |  |  |  |  |  |  |  |  |  |  |  |  |  |
|  |  |  |  |  |  |  |  |  |  |  |  |  |  |  |  |  |  |  |  |  |  |  |  |  |  |  |  |  |  |  |  |  |  |  |  |  |  |  |  |  |  |  |
|  |  |  |  |  |  |  |  |  |  |  |  |  |  |  |  |  |  |  |  |  |  |  |  |  |  |  |  |  |  |  |  |  |  |  |  |  |  |  |  |  |  |  |
|  |  |  |  |  |  |  |  |  |  |  |  |  |  |  |  |  |  |  |  |  |  |  |  |  |  |  |  |  |  |  |  |  |  |  |  |  |  |  |  |  |  |  |
|  |  |  |  |  |  |  |  |  |  |  |  |  |  |  |  |  |  |  |  |  |  |  |  |  |  |  |  |  |  |  |  |  |  |  |  |  |  |  |  |  |  |  |
|  |  |  |  |  |  |  |  |  |  |  |  |  |  |  |  |  |  |  |  |  |  |  |  |  |  |  |  |  |  |  |  |  |  |  |  |  |  |  |  |  |  |  |
|  |  |  |  |  |  |  |  |  |  |  |  |  |  |  |  |  |  |  |  |  |  |  |  |  |  |  |  |  |  |  |  |  |  |  |  |  |  |  |  |  |  |  |
|  |  |  |  |  |  |  |  |  |  |  |  |  |  |  |  |  |  |  |  |  |  |  |  |  |  |  |  |  |  |  |  |  |  |  |  |  |  |  |  |  |  |  |
|  |  |  |  |  |  |  |  |  |  |  |  |  |  |  |  |  |  |  |  |  |  |  |  |  |  |  |  |  |  |  |  |  |  |  |  |  |  |  |  |  |  |  |
|  |  |  |  |  |  |  |  |  |  |  |  |  |  |  |  |  |  |  |  |  |  |  |  |  |  |  |  |  |  |  |  |  |  |  |  |  |  |  |  |  |  |  |
|  |  |  |  |  |  |  |  |  |  |  |  |  |  |  |  |  |  |  |  |  |  |  |  |  |  |  |  |  |  |  |  |  |  |  |  |  |  |  |  |  |  |  |
|  |  |  |  |  |  |  |  |  |  |  |  |  |  |  |  |  |  |  |  |  |  |  |  |  |  |  |  |  |  |  |  |  |  |  |  |  |  |  |  |  |  |  |
|  |  |  |  |  |  |  |  |  |  |  |  |  |  |  |  |  |  |  |  |  |  |  |  |  |  |  |  |  |  |  |  |  |  |  |  |  |  |  |  |  |  |  |
|  |  |  |  |  |  |  |  |  |  |  |  |  |  |  |  |  |  |  |  |  |  |  |  |  |  |  |  |  |  |  |  |  |  |  |  |  |  |  |  |  |  |  |
|  |  |  |  |  |  |  |  |  |  |  |  |  |  |  |  |  |  |  |  |  |  |  |  |  |  |  |  |  |  |  |  |  |  |  |  |  |  |  |  |  |  |  |
|  |  |  |  |  |  |  |  |  |  |  |  |  |  |  |  |  |  |  |  |  |  |  |  |  |  |  |  |  |  |  |  |  |  |  |  |  |  |  |  |  |  |  |
|  |  |  |  |  |  |  |  |  |  |  |  |  |  |  |  |  |  |  |  |  |  |  |  |  |  |  |  |  |  |  |  |  |  |  |  |  |  |  |  |  |  |  |
|  |  |  |  |  |  |  |  |  |  |  |  |  |  |  |  |  |  |  |  |  |  |  |  |  |  |  |  |  |  |  |  |  |  |  |  |  |  |  |  |  |  |  |
|  |  |  |  |  |  |  |  |  |  |  |  |  |  |  |  |  |  |  |  |  |  |  |  |  |  |  |  |  |  |  |  |  |  |  |  |  |  |  |  |  |  |  |
|  |  |  |  |  |  |  |  |  |  |  |  |  |  |  |  |  |  |  |  |  |  |  |  |  |  |  |  |  |  |  |  |  |  |  |  |  |  |  |  |  |  |  |
|  |  |  |  |  |  |  |  |  |  |  |  |  |  |  |  |  |  |  |  |  |  |  |  |  |  |  |  |  |  |  |  |  |  |  |  |  |  |  |  |  |  |  |
|  |  |  |  |  |  |  |  |  |  |  |  |  |  |  |  |  |  |  |  |  |  |  |  |  |  |  |  |  |  |  |  |  |  |  |  |  |  |  |  |  |  |  |
|  |  |  |  |  |  |  |  |  |  |  |  |  |  |  |  |  |  |  |  |  |  |  |  |  |  |  |  |  |  |  |  |  |  |  |  |  |  |  |  |  |  |  |
|  |  |  |  |  |  |  |  |  |  |  |  |  |  |  |  |  |  |  |  |  |  |  |  |  |  |  |  |  |  |  |  |  |  |  |  |  |  |  |  |  |  |  |
|  |  |  |  |  |  |  |  |  |  |  |  |  |  |  |  |  |  |  |  |  |  |  |  |  |  |  |  |  |  |  |  |  |  |  |  |  |  |  |  |  |  |  |
|  |  |  |  |  |  |  |  |  |  |  |  |  |  |  |  |  |  |  |  |  |  |  |  |  |  |  |  |  |  |  |  |  |  |  |  |  |  |  |  |  |  |  |
|  |  |  |  |  |  |  |  |  |  |  |  |  |  |  |  |  |  |  |  |  |  |  |  |  |  |  |  |  |  |  |  |  |  |  |  |  |  |  |  |  |  |  |
|  |  |  |  |  |  |  |  |  |  |  |  |  |  |  |  |  |  |  |  |  |  |  |  |  |  |  |  |  |  |  |  |  |  |  |  |  |  |  |  |  |  |  |
|  |  |  |  |  |  |  |  |  |  |  |  |  |  |  |  |  |  |  |  |  |  |  |  |  |  |  |  |  |  |  |  |  |  |  |  |  |  |  |  |  |  |  |
|  |  |  |  |  |  |  |  |  |  |  |  |  |  |  |  |  |  |  |  |  |  |  |  |  |  |  |  |  |  |  |  |  |  |  |  |  |  |  |  |  |  |  |
|  |  |  |  |  |  |  |  |  |  |  |  |  |  |  |  |  |  |  |  |  |  |  |  |  |  |  |  |  |  |  |  |  |  |  |  |  |  |  |  |  |  |  |
|  |  |  |  |  |  |  |  |  |  |  |  |  |  |  |  |  |  |  |  |  |  |  |  |  |  |  |  |  |  |  |  |  |  |  |  |  |  |  |  |  |  |  |
|  |  |  |  |  |  |  |  |  |  |  |  |  |  |  |  |  |  |  |  |  |  |  |  |  |  |  |  |  |  |  |  |  |  |  |  |  |  |  |  |  |  |  |
|  |  |  |  |  |  |  |  |  |  |  |  |  |  |  |  |  |  |  |  |  |  |  |  |  |  |  |  |  |  |  |  |  |  |  |  |  |  |  |  |  |  |  |
|  |  |  |  |  |  |  |  |  |  |  |  |  |  |  |  |  |  |  |  |  |  |  |  |  |  |  |  |  |  |  |  |  |  |  |  |  |  |  |  |  |  |  |
|  |  |  |  |  |  |  |  |  |  |  |  |  |  |  |  |  |  |  |  |  |  |  |  |  |  |  |  |  |  |  |  |  |  |  |  |  |  |  |  |  |  |  |
|  |  |  |  |  |  |  |  |  |  |  |  |  |  |  |  |  |  |  |  |  |  |  |  |  |  |  |  |  |  |  |  |  |  |  |  |  |  |  |  |  |  |  |
|  |  |  |  |  |  |  |  |  |  |  |  |  |  |  |  |  |  |  |  |  |  |  |  |  |  |  |  |  |  |  |  |  |  |  |  |  |  |  |  |  |  |  |
|  |  |  |  |  |  |  |  |  |  |  |  |  |  |  |  |  |  |  |  |  |  |  |  |  |  |  |  |  |  |  |  |  |  |  |  |  |  |  |  |  |  |  |
|  |  |  |  |  |  |  |  |  |  |  |  |  |  |  |  |  |  |  |  |  |  |  |  |  |  |  |  |  |  |  |  |  |  |  |  |  |  |  |  |  |  |  |
|  |  |  |  |  |  |  |  |  |  |  |  |  |  |  |  |  |  |  |  |  |  |  |  |  |  |  |  |  |  |  |  |  |  |  |  |  |  |  |  |  |  |  |
|  |  |  |  |  |  |  |  |  |  |  |  |  |  |  |  |  |  |  |  |  |  |  |  |  |  |  |  |  |  |  |  |  |  |  |  |  |  |  |  |  |  |  |
|  |  |  |  |  |  |  |  |  |  |  |  |  |  |  |  |  |  |  |  |  |  |  |  |  |  |  |  |  |  |  |  |  |  |  |  |  |  |  |  |  |  |  |
|  |  |  |  |  |  |  |  |  |  |  |  |  |  |  |  |  |  |  |  |  |  |  |  |  |  |  |  |  |  |  |  |  |  |  |  |  |  |  |  |  |  |  |
|  |  |  |  |  |  |  |  |  |  |  |  |  |  |  |  |  |  |  |  |  |  |  |  |  |  |  |  |  |  |  |  |  |  |  |  |  |  |  |  |  |  |  |
|  |  |  |  |  |  |  |  |  |  |  |  |  |  |  |  |  |  |  |  |  |  |  |  |  |  |  |  |  |  |  |  |  |  |  |  |  |  |  |  |  |  |  |
|  |  |  |  |  |  |  |  |  |  |  |  |  |  |  |  |  |  |  |  |  |  |  |  |  |  |  |  |  |  |  |  |  |  |  |  |  |  |  |  |  |  |  |
|  |  |  |  |  |  |  |  |  |  |  |  |  |  |  |  |  |  |  |  |  |  |  |  |  |  |  |  |  |  |  |  |  |  |  |  |  |  |  |  |  |  |  |
|  |  |  |  |  |  |  |  |  |  |  |  |  |  |  |  |  |  |  |  |  |  |  |  |  |  |  |  |  |  |  |  |  |  |  |  |  |  |  |  |  |  |  |
|  |  |  |  |  |  |  |  |  |  |  |  |  |  |  |  |  |  |  |  |  |  |  |  |  |  |  |  |  |  |  |  |  |  |  |  |  |  |  |  |  |  |  |
|  |  |  |  |  |  |  |  |  |  |  |  |  |  |  |  |  |  |  |  |  |  |  |  |  |  |  |  |  |  |  |  |  |  |  |  |  |  |  |  |  |  |  |
|  |  |  |  |  |  |  |  |  |  |  |  |  |  |  |  |  |  |  |  |  |  |  |  |  |  |  |  |  |  |  |  |  |  |  |  |  |  |  |  |  |  |  |
|  |  |  |  |  |  |  |  |  |  |  |  |  |  |  |  |  |  |  |  |  |  |  |  |  |  |  |  |  |  |  |  |  |  |  |  |  |  |  |  |  |  |  |
|  |  |  |  |  |  |  |  |  |  |  |  |  |  |  |  |  |  |  |  |  |  |  |  |  |  |  |  |  |  |  |  |  |  |  |  |  |  |  |  |  |  |  |
|  |  |  |  |  |  |  |  |  |  |  |  |  |  |  |  |  |  |  |  |  |  |  |  |  |  |  |  |  |  |  |  |  |  |  |  |  |  |  |  |  |  |  |
|  |  |  |  |  |  |  |  |  |  |  |  |  |  |  |  |  |  |  |  |  |  |  |  |  |  |  |  |  |  |  |  |  |  |  |  |  |  |  |  |  |  |  |
|  |  |  |  |  |  |  |  |  |  |  |  |  |  |  |  |  |  |  |  |  |  |  |  |  |  |  |  |  |  |  |  |  |  |  |  |  |  |  |  |  |  |  |
|  |  |  |  |  |  |  |  |  |  |  |  |  |  |  |  |  |  |  |  |  |  |  |  |  |  |  |  |  |  |  |  |  |  |  |  |  |  |  |  |  |  |  |
|  |  |  |  |  |  |  |  |  |  |  |  |  |  |  |  |  |  |  |  |  |  |  |  |  |  |  |  |  |  |  |  |  |  |  |  |  |  |  |  |  |  |  |
|  |  |  |  |  |  |  |  |  |  |  |  |  |  |  |  |  |  |  |  |  |  |  |  |  |  |  |  |  |  |  |  |  |  |  |  |  |  |  |  |  |  |  |
|  |  |  |  |  |  |  |  |  |  |  |  |  |  |  |  |  |  |  |  |  |  |  |  |  |  |  |  |  |  |  |  |  |  |  |  |  |  |  |  |  |  |  |
|  |  |  |  |  |  |  |  |  |  |  |  |  |  |  |  |  |  |  |  |  |  |  |  |  |  |  |  |  |  |  |  |  |  |  |  |  |  |  |  |  |  |  |
|  |  |  |  |  |  |  |  |  |  |  |  |  |  |  |  |  |  |  |  |  |  |  |  |  |  |  |  |  |  |  |  |  |  |  |  |  |  |  |  |  |  |  |
|  |  |  |  |  |  |  |  |  |  |  |  |  |  |  |  |  |  |  |  |  |  |  |  |  |  |  |  |  |  |  |  |  |  |  |  |  |  |  |  |  |  |  |
|  |  |  |  |  |  |  |  |  |  |  |  |  |  |  |  |  |  |  |  |  |  |  |  |  |  |  |  |  |  |  |  |  |  |  |  |  |  |  |  |  |  |  |
|  |  |  |  |  |  |  |  |  |  |  |  |  |  |  |  |  |  |  |  |  |  |  |  |  |  |  |  |  |  |  |  |  |  |  |  |  |  |  |  |  |  |  |
|  |  |  |  |  |  |  |  |  |  |  |  |  |  |  |  |  |  |  |  |  |  |  |  |  |  |  |  |  |  |  |  |  |  |  |  |  |  |  |  |  |  |  |
|  |  |  |  |  |  |  |  |  |  |  |  |  |  |  |  |  |  |  |  |  |  |  |  |  |  |  |  |  |  |  |  |  |  |  |  |  |  |  |  |  |  |  |
|  |  |  |  |  |  |  |  |  |  |  |  |  |  |  |  |  |  |  |  |  |  |  |  |  |  |  |  |  |  |  |  |  |  |  |  |  |  |  |  |  |  |  |
|  |  |  |  |  |  |  |  |  |  |  |  |  |  |  |  |  |  |  |  |  |  |  |  |  |  |  |  |  |  |  |  |  |  |  |  |  |  |  |  |  |  |  |
|  |  |  |  |  |  |  |  |  |  |  |  |  |  |  |  |  |  |  |  |  |  |  |  |  |  |  |  |  |  |  |  |  |  |  |  |  |  |  |  |  |  |  |
|  |  |  |  |  |  |  |  |  |  |  |  |  |  |  |  |  |  |  |  |  |  |  |  |  |  |  |  |  |  |  |  |  |  |  |  |  |  |  |  |  |  |  |
|  |  |  |  |  |  |  |  |  |  |  |  |  |  |  |  |  |  |  |  |  |  |  |  |  |  |  |  |  |  |  |  |  |  |  |  |  |  |  |  |  |  |  |
|  |  |  |  |  |  |  |  |  |  |  |  |  |  |  |  |  |  |  |  |  |  |  |  |  |  |  |  |  |  |  |  |  |  |  |  |  |  |  |  |  |  |  |
|  |  |  |  |  |  |  |  |  |  |  |  |  |  |  |  |  |  |  |  |  |  |  |  |  |  |  |  |  |  |  |  |  |  |  |  |  |  |  |  |  |  |  |
|  |  |  |  |  |  |  |  |  |  |  |  |  |  |  |  |  |  |  |  |  |  |  |  |  |  |  |  |  |  |  |  |  |  |  |  |  |  |  |  |  |  |  |
|  |  |  |  |  |  |  |  |  |  |  |  |  |  |  |  |  |  |  |  |  |  |  |  |  |  |  |  |  |  |  |  |  |  |  |  |  |  |  |  |  |  |  |
|  |  |  |  |  |  |  |  |  |  |  |  |  |  |  |  |  |  |  |  |  |  |  |  |  |  |  |  |  |  |  |  |  |  |  |  |  |  |  |  |  |  |  |
|  |  |  |  |  |  |  |  |  |  |  |  |  |  |  |  |  |  |  |  |  |  |  |  |  |  |  |  |  |  |  |  |  |  |  |  |  |  |  |  |  |  |  |
|  |  |  |  |  |  |  |  |  |  |  |  |  |  |  |  |  |  |  |  |  |  |  |  |  |  |  |  |  |  |  |  |  |  |  |  |  |  |  |  |  |  |  |
|  |  |  |  |  |  |  |  |  |  |  |  |  |  |  |  |  |  |  |  |  |  |  |  |  |  |  |  |  |  |  |  |  |  |  |  |  |  |  |  |  |  |  |
|  |  |  |  |  |  |  |  |  |  |  |  |  |  |  |  |  |  |  |  |  |  |  |  |  |  |  |  |  |  |  |  |  |  |  |  |  |  |  |  |  |  |  |
|  |  |  |  |  |  |  |  |  |  |  |  |  |  |  |  |  |  |  |  |  |  |  |  |  |  |  |  |  |  |  |  |  |  |  |  |  |  |  |  |  |  |  |
|  |  |  |  |  |  |  |  |  |  |  |  |  |  |  |  |  |  |  |  |  |  |  |  |  |  |  |  |  |  |  |  |  |  |  |  |  |  |  |  |  |  |  |
|  |  |  |  |  |  |  |  |  |  |  |  |  |  |  |  |  |  |  |  |  |  |  |  |  |  |  |  |  |  |  |  |  |  |  |  |  |  |  |  |  |  |  |
|  |  |  |  |  |  |  |  |  |  |  |  |  |  |  |  |  |  |  |  |  |  |  |  |  |  |  |  |  |  |  |  |  |  |  |  |  |  |  |  |  |  |  |
|  |  |  |  |  |  |  |  |  |  |  |  |  |  |  |  |  |  |  |  |  |  |  |  |  |  |  |  |  |  |  |  |  |  |  |  |  |  |  |  |  |  |  |
|  |  |  |  |  |  |  |  |  |  |  |  |  |  |  |  |  |  |  |  |  |  |  |  |  |  |  |  |  |  |  |  |  |  |  |  |  |  |  |  |  |  |  |
|  |  |  |  |  |  |  |  |  |  |  |  |  |  |  |  |  |  |  |  |  |  |  |  |  |  |  |  |  |  |  |  |  |  |  |  |  |  |  |  |  |  |  |
|  |  |  |  |  |  |  |  |  |  |  |  |  |  |  |  |  |  |  |  |  |  |  |  |  |  |  |  |  |  |  |  |  |  |  |  |  |  |  |  |  |  |  |
|  |  |  |  |  |  |  |  |  |  |  |  |  |  |  |  |  |  |  |  |  |  |  |  |  |  |  |  |  |  |  |  |  |  |  |  |  |  |  |  |  |  |  |
|  |  |  |  |  |  |  |  |  |  |  |  |  |  |  |  |  |  |  |  |  |  |  |  |  |  |  |  |  |  |  |  |  |  |  |  |  |  |  |  |  |  |  |
|  |  |  |  |  |  |  |  |  |  |  |  |  |  |  |  |  |  |  |  |  |  |  |  |  |  |  |  |  |  |  |  |  |  |  |  |  |  |  |  |  |  |  |
|  |  |  |  |  |  |  |  |  |  |  |  |  |  |  |  |  |  |  |  |  |  |  |  |  |  |  |  |  |  |  |  |  |  |  |  |  |  |  |  |  |  |  |
|  |  |  |  |  |  |  |  |  |  |  |  |  |  |  |  |  |  |  |  |  |  |  |  |  |  |  |  |  |  |  |  |  |  |  |  |  |  |  |  |  |  |  |
|  |  |  |  |  |  |  |  |  |  |  |  |  |  |  |  |  |  |  |  |  |  |  |  |  |  |  |  |  |  |  |  |  |  |  |  |  |  |  |  |  |  |  |
|  |  |  |  |  |  |  |  |  |  |  |  |  |  |  |  |  |  |  |  |  |  |  |  |  |  |  |  |  |  |  |  |  |  |  |  |  |  |  |  |  |  |  |
|  |  |  |  |  |  |  |  |  |  |  |  |  |  |  |  |  |  |  |  |  |  |  |  |  |  |  |  |  |  |  |  |  |  |  |  |  |  |  |  |  |  |  |
|  |  |  |  |  |  |  |  |  |  |  |  |  |  |  |  |  |  |  |  |  |  |  |  |  |  |  |  |  |  |  |  |  |  |  |  |  |  |  |  |  |  |  |
|  |  |  |  |  |  |  |  |  |  |  |  |  |  |  |  |  |  |  |  |  |  |  |  |  |  |  |  |  |  |  |  |  |  |  |  |  |  |  |  |  |  |  |
|  |  |  |  |  |  |  |  |  |  |  |  |  |  |  |  |  |  |  |  |  |  |  |  |  |  |  |  |  |  |  |  |  |  |  |  |  |  |  |  |  |  |  |
|  |  |  |  |  |  |  |  |  |  |  |  |  |  |  |  |  |  |  |  |  |  |  |  |  |  |  |  |  |  |  |  |  |  |  |  |  |  |  |  |  |  |  |
|  |  |  |  |  |  |  |  |  |  |  |  |  |  |  |  |  |  |  |  |  |  |  |  |  |  |  |  |  |  |  |  |  |  |  |  |  |  |  |  |  |  |  |
|  |  |  |  |  |  |  |  |  |  |  |  |  |  |  |  |  |  |  |  |  |  |  |  |  |  |  |  |  |  |  |  |  |  |  |  |  |  |  |  |  |  |  |
|  |  |  |  |  |  |  |  |  |  |  |  |  |  |  |  |  |  |  |  |  |  |  |  |  |  |  |  |  |  |  |  |  |  |  |  |  |  |  |  |  |  |  |
|  |  |  |  |  |  |  |  |  |  |  |  |  |  |  |  |  |  |  |  |  |  |  |  |  |  |  |  |  |  |  |  |  |  |  |  |  |  |  |  |  |  |  |
|  |  |  |  |  |  |  |  |  |  |  |  |  |  |  |  |  |  |  |  |  |  |  |  |  |  |  |  |  |  |  |  |  |  |  |  |  |  |  |  |  |  |  |
|  |  |  |  |  |  |  |  |  |  |  |  |  |  |  |  |  |  |  |  |  |  |  |  |  |  |  |  |  |  |  |  |  |  |  |  |  |  |  |  |  |  |  |
|  |  |  |  |  |  |  |  |  |  |  |  |  |  |  |  |  |  |  |  |  |  |  |  |  |  |  |  |  |  |  |  |  |  |  |  |  |  |  |  |  |  |  |
|  |  |  |  |  |  |  |  |  |  |  |  |  |  |  |  |  |  |  |  |  |  |  |  |  |  |  |  |  |  |  |  |  |  |  |  |  |  |  |  |  |  |  |
|  |  |  |  |  |  |  |  |  |  |  |  |  |  |  |  |  |  |  |  |  |  |  |  |  |  |  |  |  |  |  |  |  |  |  |  |  |  |  |  |  |  |  |
|  |  |  |  |  |  |  |  |  |  |  |  |  |  |  |  |  |  |  |  |  |  |  |  |  |  |  |  |  |  |  |  |  |  |  |  |  |  |  |  |  |  |  |
|  |  |  |  |  |  |  |  |  |  |  |  |  |  |  |  |  |  |  |  |  |  |  |  |  |  |  |  |  |  |  |  |  |  |  |  |  |  |  |  |  |  |  |
|  |  |  |  |  |  |  |  |  |  |  |  |  |  |  |  |  |  |  |  |  |  |  |  |  |  |  |  |  |  |  |  |  |  |  |  |  |  |  |  |  |  |  |
|  |  |  |  |  |  |  |  |  |  |  |  |  |  |  |  |  |  |  |  |  |  |  |  |  |  |  |  |  |  |  |  |  |  |  |  |  |  |  |  |  |  |  |
|  |  |  |  |  |  |  |  |  |  |  |  |  |  |  |  |  |  |  |  |  |  |  |  |  |  |  |  |  |  |  |  |  |  |  |  |  |  |  |  |  |  |  |
|  |  |  |  |  |  |  |  |  |  |  |  |  |  |  |  |  |  |  |  |  |  |  |  |  |  |  |  |  |  |  |  |  |  |  |  |  |  |  |  |  |  |  |
|  |  |  |  |  |  |  |  |  |  |  |  |  |  |  |  |  |  |  |  |  |  |  |  |  |  |  |  |  |  |  |  |  |  |  |  |  |  |  |  |  |  |  |
|  |  |  |  |  |  |  |  |  |  |  |  |  |  |  |  |  |  |  |  |  |  |  |  |  |  |  |  |  |  |  |  |  |  |  |  |  |  |  |  |  |  |  |
|  |  |  |  |  |  |  |  |  |  |  |  |  |  |  |  |  |  |  |  |  |  |  |  |  |  |  |  |  |  |  |  |  |  |  |  |  |  |  |  |  |  |  |
|  |  |  |  |  |  |  |  |  |  |  |  |  |  |  |  |  |  |  |  |  |  |  |  |  |  |  |  |  |  |  |  |  |  |  |  |  |  |  |  |  |  |  |
|  |  |  |  |  |  |  |  |  |  |  |  |  |  |  |  |  |  |  |  |  |  |  |  |  |  |  |  |  |  |  |  |  |  |  |  |  |  |  |  |  |  |  |
|  |  |  |  |  |  |  |  |  |  |  |  |  |  |  |  |  |  |  |  |  |  |  |  |  |  |  |  |  |  |  |  |  |  |  |  |  |  |  |  |  |  |  |
|  |  |  |  |  |  |  |  |  |  |  |  |  |  |  |  |  |  |  |  |  |  |  |  |  |  |  |  |  |  |  |  |  |  |  |  |  |  |  |  |  |  |  |
|  |  |  |  |  |  |  |  |  |  |  |  |  |  |  |  |  |  |  |  |  |  |  |  |  |  |  |  |  |  |  |  |  |  |  |  |  |  |  |  |  |  |  |
|  |  |  |  |  |  |  |  |  |  |  |  |  |  |  |  |  |  |  |  |  |  |  |  |  |  |  |  |  |  |  |  |  |  |  |  |  |  |  |  |  |  |  |
|  |  |  |  |  |  |  |  |  |  |  |  |  |  |  |  |  |  |  |  |  |  |  |  |  |  |  |  |  |  |  |  |  |  |  |  |  |  |  |  |  |  |  |
|  |  |  |  |  |  |  |  |  |  |  |  |  |  |  |  |  |  |  |  |  |  |  |  |  |  |  |  |  |  |  |  |  |  |  |  |  |  |  |  |  |  |  |
|  |  |  |  |  |  |  |  |  |  |  |  |  |  |  |  |  |  |  |  |  |  |  |  |  |  |  |  |  |  |  |  |  |  |  |  |  |  |  |  |  |  |  |
|  |  |  |  |  |  |  |  |  |  |  |  |  |  |  |  |  |  |  |  |  |  |  |  |  |  |  |  |  |  |  |  |  |  |  |  |  |  |  |  |  |  |  |
|  |  |  |  |  |  |  |  |  |  |  |  |  |  |  |  |  |  |  |  |  |  |  |  |  |  |  |  |  |  |  |  |  |  |  |  |  |  |  |  |  |  |  |
|  |  |  |  |  |  |  |  |  |  |  |  |  |  |  |  |  |  |  |  |  |  |  |  |  |  |  |  |  |  |  |  |  |  |  |  |  |  |  |  |  |  |  |
|  |  |  |  |  |  |  |  |  |  |  |  |  |  |  |  |  |  |  |  |  |  |  |  |  |  |  |  |  |  |  |  |  |  |  |  |  |  |  |  |  |  |  |
|  |  |  |  |  |  |  |  |  |  |  |  |  |  |  |  |  |  |  |  |  |  |  |  |  |  |  |  |  |  |  |  |  |  |  |  |  |  |  |  |  |  |  |
|  |  |  |  |  |  |  |  |  |  |  |  |  |  |  |  |  |  |  |  |  |  |  |  |  |  |  |  |  |  |  |  |  |  |  |  |  |  |  |  |  |  |  |
|  |  |  |  |  |  |  |  |  |  |  |  |  |  |  |  |  |  |  |  |  |  |  |  |  |  |  |  |  |  |  |  |  |  |  |  |  |  |  |  |  |  |  |
|  |  |  |  |  |  |  |  |  |  |  |  |  |  |  |  |  |  |  |  |  |  |  |  |  |  |  |  |  |  |  |  |  |  |  |  |  |  |  |  |  |  |  |
|  |  |  |  |  |  |  |  |  |  |  |  |  |  |  |  |  |  |  |  |  |  |  |  |  |  |  |  |  |  |  |  |  |  |  |  |  |  |  |  |  |  |  |
|  |  |  |  |  |  |  |  |  |  |  |  |  |  |  |  |  |  |  |  |  |  |  |  |  |  |  |  |  |  |  |  |  |  |  |  |  |  |  |  |  |  |  |
|  |  |  |  |  |  |  |  |  |  |  |  |  |  |  |  |  |  |  |  |  |  |  |  |  |  |  |  |  |  |  |  |  |  |  |  |  |  |  |  |  |  |  |
|  |  |  |  |  |  |  |  |  |  |  |  |  |  |  |  |  |  |  |  |  |  |  |  |  |  |  |  |  |  |  |  |  |  |  |  |  |  |  |  |  |  |  |
|  |  |  |  |  |  |  |  |  |  |  |  |  |  |  |  |  |  |  |  |  |  |  |  |  |  |  |  |  |  |  |  |  |  |  |  |  |  |  |  |  |  |  |
|  |  |  |  |  |  |  |  |  |  |  |  |  |  |  |  |  |  |  |  |  |  |  |  |  |  |  |  |  |  |  |  |  |  |  |  |  |  |  |  |  |  |  |
|  |  |  |  |  |  |  |  |  |  |  |  |  |  |  |  |  |  |  |  |  |  |  |  |  |  |  |  |  |  |  |  |  |  |  |  |  |  |  |  |  |  |  |
|  |  |  |  |  |  |  |  |  |  |  |  |  |  |  |  |  |  |  |  |  |  |  |  |  |  |  |  |  |  |  |  |  |  |  |  |  |  |  |  |  |  |  |
|  |  |  |  |  |  |  |  |  |  |  |  |  |  |  |  |  |  |  |  |  |  |  |  |  |  |  |  |  |  |  |  |  |  |  |  |  |  |  |  |  |  |  |
|  |  |  |  |  |  |  |  |  |  |  |  |  |  |  |  |  |  |  |  |  |  |  |  |  |  |  |  |  |  |  |  |  |  |  |  |  |  |  |  |  |  |  |
|  |  |  |  |  |  |  |  |  |  |  |  |  |  |  |  |  |  |  |  |  |  |  |  |  |  |  |  |  |  |  |  |  |  |  |  |  |  |  |  |  |  |  |
|  |  |  |  |  |  |  |  |  |  |  |  |  |  |  |  |  |  |  |  |  |  |  |  |  |  |  |  |  |  |  |  |  |  |  |  |  |  |  |  |  |  |  |
|  |  |  |  |  |  |  |  |  |  |  |  |  |  |  |  |  |  |  |  |  |  |  |  |  |  |  |  |  |  |  |  |  |  |  |  |  |  |  |  |  |  |  |
|  |  |  |  |  |  |  |  |  |  |  |  |  |  |  |  |  |  |  |  |  |  |  |  |  |  |  |  |  |  |  |  |  |  |  |  |  |  |  |  |  |  |  |
|  |  |  |  |  |  |  |  |  |  |  |  |  |  |  |  |  |  |  |  |  |  |  |  |  |  |  |  |  |  |  |  |  |  |  |  |  |  |  |  |  |  |  |
|  |  |  |  |  |  |  |  |  |  |  |  |  |  |  |  |  |  |  |  |  |  |  |  |  |  |  |  |  |  |  |  |  |  |  |  |  |  |  |  |  |  |  |
|  |  |  |  |  |  |  |  |  |  |  |  |  |  |  |  |  |  |  |  |  |  |  |  |  |  |  |  |  |  |  |  |  |  |  |  |  |  |  |  |  |  |  |
|  |  |  |  |  |  |  |  |  |  |  |  |  |  |  |  |  |  |  |  |  |  |  |  |  |  |  |  |  |  |  |  |  |  |  |  |  |  |  |  |  |  |  |
|  |  |  |  |  |  |  |  |  |  |  |  |  |  |  |  |  |  |  |  |  |  |  |  |  |  |  |  |  |  |  |  |  |  |  |  |  |  |  |  |  |  |  |
|  |  |  |  |  |  |  |  |  |  |  |  |  |  |  |  |  |  |  |  |  |  |  |  |  |  |  |  |  |  |  |  |  |  |  |  |  |  |  |  |  |  |  |
|  |  |  |  |  |  |  |  |  |  |  |  |  |  |  |  |  |  |  |  |  |  |  |  |  |  |  |  |  |  |  |  |  |  |  |  |  |  |  |  |  |  |  |
|  |  |  |  |  |  |  |  |  |  |  |  |  |  |  |  |  |  |  |  |  |  |  |  |  |  |  |  |  |  |  |  |  |  |  |  |  |  |  |  |  |  |  |
|  |  |  |  |  |  |  |  |  |  |  |  |  |  |  |  |  |  |  |  |  |  |  |  |  |  |  |  |  |  |  |  |  |  |  |  |  |  |  |  |  |  |  |
|  |  |  |  |  |  |  |  |  |  |  |  |  |  |  |  |  |  |  |  |  |  |  |  |  |  |  |  |  |  |  |  |  |  |  |  |  |  |  |  |  |  |  |
|  |  |  |  |  |  |  |  |  |  |  |  |  |  |  |  |  |  |  |  |  |  |  |  |  |  |  |  |  |  |  |  |  |  |  |  |  |  |  |  |  |  |  |
|  |  |  |  |  |  |  |  |  |  |  |  |  |  |  |  |  |  |  |  |  |  |  |  |  |  |  |  |  |  |  |  |  |  |  |  |  |  |  |  |  |  |  |
|  |  |  |  |  |  |  |  |  |  |  |  |  |  |  |  |  |  |  |  |  |  |  |  |  |  |  |  |  |  |  |  |  |  |  |  |  |  |  |  |  |  |  |
|  |  |  |  |  |  |  |  |  |  |  |  |  |  |  |  |  |  |  |  |  |  |  |  |  |  |  |  |  |  |  |  |  |  |  |  |  |  |  |  |  |  |  |
|  |  |  |  |  |  |  |  |  |  |  |  |  |  |  |  |  |  |  |  |  |  |  |  |  |  |  |  |  |  |  |  |  |  |  |  |  |  |  |  |  |  |  |
|  |  |  |  |  |  |  |  |  |  |  |  |  |  |  |  |  |  |  |  |  |  |  |  |  |  |  |  |  |  |  |  |  |  |  |  |  |  |  |  |  |  |  |
|  |  |  |  |  |  |  |  |  |  |  |  |  |  |  |  |  |  |  |  |  |  |  |  |  |  |  |  |  |  |  |  |  |  |  |  |  |  |  |  |  |  |  |
|  |  |  |  |  |  |  |  |  |  |  |  |  |  |  |  |  |  |  |  |  |  |  |  |  |  |  |  |  |  |  |  |  |  |  |  |  |  |  |  |  |  |  |
|  |  |  |  |  |  |  |  |  |  |  |  |  |  |  |  |  |  |  |  |  |  |  |  |  |  |  |  |  |  |  |  |  |  |  |  |  |  |  |  |  |  |  |
|  |  |  |  |  |  |  |  |  |  |  |  |  |  |  |  |  |  |  |  |  |  |  |  |  |  |  |  |  |  |  |  |  |  |  |  |  |  |  |  |  |  |  |
|  |  |  |  |  |  |  |  |  |  |  |  |  |  |  |  |  |  |  |  |  |  |  |  |  |  |  |  |  |  |  |  |  |  |  |  |  |  |  |  |  |  |  |
|  |  |  |  |  |  |  |  |  |  |  |  |  |  |  |  |  |  |  |  |  |  |  |  |  |  |  |  |  |  |  |  |  |  |  |  |  |  |  |  |  |  |  |
|  |  |  |  |  |  |  |  |  |  |  |  |  |  |  |  |  |  |  |  |  |  |  |  |  |  |  |  |  |  |  |  |  |  |  |  |  |  |  |  |  |  |  |
|  |  |  |  |  |  |  |  |  |  |  |  |  |  |  |  |  |  |  |  |  |  |  |  |  |  |  |  |  |  |  |  |  |  |  |  |  |  |  |  |  |  |  |
|  |  |  |  |  |  |  |  |  |  |  |  |  |  |  |  |  |  |  |  |  |  |  |  |  |  |  |  |  |  |  |  |  |  |  |  |  |  |  |  |  |  |  |
|  |  |  |  |  |  |  |  |  |  |  |  |  |  |  |  |  |  |  |  |  |  |  |  |  |  |  |  |  |  |  |  |  |  |  |  |  |  |  |  |  |  |  |
|  |  |  |  |  |  |  |  |  |  |  |  |  |  |  |  |  |  |  |  |  |  |  |  |  |  |  |  |  |  |  |  |  |  |  |  |  |  |  |  |  |  |  |
|  |  |  |  |  |  |  |  |  |  |  |  |  |  |  |  |  |  |  |  |  |  |  |  |  |  |  |  |  |  |  |  |  |  |  |  |  |  |  |  |  |  |  |
|  |  |  |  |  |  |  |  |  |  |  |  |  |  |  |  |  |  |  |  |  |  |  |  |  |  |  |  |  |  |  |  |  |  |  |  |  |  |  |  |  |  |  |
|  |  |  |  |  |  |  |  |  |  |  |  |  |  |  |  |  |  |  |  |  |  |  |  |  |  |  |  |  |  |  |  |  |  |  |  |  |  |  |  |  |  |  |
|  |  |  |  |  |  |  |  |  |  |  |  |  |  |  |  |  |  |  |  |  |  |  |  |  |  |  |  |  |  |  |  |  |  |  |  |  |  |  |  |  |  |  |
|  |  |  |  |  |  |  |  |  |  |  |  |  |  |  |  |  |  |  |  |  |  |  |  |  |  |  |  |  |  |  |  |  |  |  |  |  |  |  |  |  |  |  |
|  |  |  |  |  |  |  |  |  |  |  |  |  |  |  |  |  |  |  |  |  |  |  |  |  |  |  |  |  |  |  |  |  |  |  |  |  |  |  |  |  |  |  |
|  |  |  |  |  |  |  |  |  |  |  |  |  |  |  |  |  |  |  |  |  |  |  |  |  |  |  |  |  |  |  |  |  |  |  |  |  |  |  |  |  |  |  |
|  |  |  |  |  |  |  |  |  |  |  |  |  |  |  |  |  |  |  |  |  |  |  |  |  |  |  |  |  |  |  |  |  |  |  |  |  |  |  |  |  |  |  |
|  |  |  |  |  |  |  |  |  |  |  |  |  |  |  |  |  |  |  |  |  |  |  |  |  |  |  |  |  |  |  |  |  |  |  |  |  |  |  |  |  |  |  |
|  |  |  |  |  |  |  |  |  |  |  |  |  |  |  |  |  |  |  |  |  |  |  |  |  |  |  |  |  |  |  |  |  |  |  |  |  |  |  |  |  |  |  |
|  |  |  |  |  |  |  |  |  |  |  |  |  |  |  |  |  |  |  |  |  |  |  |  |  |  |  |  |  |  |  |  |  |  |  |  |  |  |  |  |  |  |  |
|  |  |  |  |  |  |  |  |  |  |  |  |  |  |  |  |  |  |  |  |  |  |  |  |  |  |  |  |  |  |  |  |  |  |  |  |  |  |  |  |  |  |  |
|  |  |  |  |  |  |  |  |  |  |  |  |  |  |  |  |  |  |  |  |  |  |  |  |  |  |  |  |  |  |  |  |  |  |  |  |  |  |  |  |  |  |  |
|  |  |  |  |  |  |  |  |  |  |  |  |  |  |  |  |  |  |  |  |  |  |  |  |  |  |  |  |  |  |  |  |  |  |  |  |  |  |  |  |  |  |  |
|  |  |  |  |  |  |  |  |  |  |  |  |  |  |  |  |  |  |  |  |  |  |  |  |  |  |  |  |  |  |  |  |  |  |  |  |  |  |  |  |  |  |  |
|  |  |  |  |  |  |  |  |  |  |  |  |  |  |  |  |  |  |  |  |  |  |  |  |  |  |  |  |  |  |  |  |  |  |  |  |  |  |  |  |  |  |  |
|  |  |  |  |  |  |  |  |  |  |  |  |  |  |  |  |  |  |  |  |  |  |  |  |  |  |  |  |  |  |  |  |  |  |  |  |  |  |  |  |  |  |  |
|  |  |  |  |  |  |  |  |  |  |  |  |  |  |  |  |  |  |  |  |  |  |  |  |  |  |  |  |  |  |  |  |  |  |  |  |  |  |  |  |  |  |  |
|  |  |  |  |  |  |  |  |  |  |  |  |  |  |  |  |  |  |  |  |  |  |  |  |  |  |  |  |  |  |  |  |  |  |  |  |  |  |  |  |  |  |  |
|  |  |  |  |  |  |  |  |  |  |  |  |  |  |  |  |  |  |  |  |  |  |  |  |  |  |  |  |  |  |  |  |  |  |  |  |  |  |  |  |  |  |  |
|  |  |  |  |  |  |  |  |  |  |  |  |  |  |  |  |  |  |  |  |  |  |  |  |  |  |  |  |  |  |  |  |  |  |  |  |  |  |  |  |  |  |  |
|  |  |  |  |  |  |  |  |  |  |  |  |  |  |  |  |  |  |  |  |  |  |  |  |  |  |  |  |  |  |  |  |  |  |  |  |  |  |  |  |  |  |  |
|  |  |  |  |  |  |  |  |  |  |  |  |  |  |  |  |  |  |  |  |  |  |  |  |  |  |  |  |  |  |  |  |  |  |  |  |  |  |  |  |  |  |  |
|  |  |  |  |  |  |  |  |  |  |  |  |  |  |  |  |  |  |  |  |  |  |  |  |  |  |  |  |  |  |  |  |  |  |  |  |  |  |  |  |  |  |  |
|  |  |  |  |  |  |  |  |  |  |  |  |  |  |  |  |  |  |  |  |  |  |  |  |  |  |  |  |  |  |  |  |  |  |  |  |  |  |  |  |  |  |  |
|  |  |  |  |  |  |  |  |  |  |  |  |  |  |  |  |  |  |  |  |  |  |  |  |  |  |  |  |  |  |  |  |  |  |  |  |  |  |  |  |  |  |  |
|  |  |  |  |  |  |  |  |  |  |  |  |  |  |  |  |  |  |  |  |  |  |  |  |  |  |  |  |  |  |  |  |  |  |  |  |  |  |  |  |  |  |  |
|  |  |  |  |  |  |  |  |  |  |  |  |  |  |  |  |  |  |  |  |  |  |  |  |  |  |  |  |  |  |  |  |  |  |  |  |  |  |  |  |  |  |  |
|  |  |  |  |  |  |  |  |  |  |  |  |  |  |  |  |  |  |  |  |  |  |  |  |  |  |  |  |  |  |  |  |  |  |  |  |  |  |  |  |  |  |  |
|  |  |  |  |  |  |  |  |  |  |  |  |  |  |  |  |  |  |  |  |  |  |  |  |  |  |  |  |  |  |  |  |  |  |  |  |  |  |  |  |  |  |  |
|  |  |  |  |  |  |  |  |  |  |  |  |  |  |  |  |  |  |  |  |  |  |  |  |  |  |  |  |  |  |  |  |  |  |  |  |  |  |  |  |  |  |  |
|  |  |  |  |  |  |  |  |  |  |  |  |  |  |  |  |  |  |  |  |  |  |  |  |  |  |  |  |  |  |  |  |  |  |  |  |  |  |  |  |  |  |  |
|  |  |  |  |  |  |  |  |  |  |  |  |  |  |  |  |  |  |  |  |  |  |  |  |  |  |  |  |  |  |  |  |  |  |  |  |  |  |  |  |  |  |  |
|  |  |  |  |  |  |  |  |  |  |  |  |  |  |  |  |  |  |  |  |  |  |  |  |  |  |  |  |  |  |  |  |  |  |  |  |  |  |  |  |  |  |  |
|  |  |  |  |  |  |  |  |  |  |  |  |  |  |  |  |  |  |  |  |  |  |  |  |  |  |  |  |  |  |  |  |  |  |  |  |  |  |  |  |  |  |  |
|  |  |  |  |  |  |  |  |  |  |  |  |  |  |  |  |  |  |  |  |  |  |  |  |  |  |  |  |  |  |  |  |  |  |  |  |  |  |  |  |  |  |  |
|  |  |  |  |  |  |  |  |  |  |  |  |  |  |  |  |  |  |  |  |  |  |  |  |  |  |  |  |  |  |  |  |  |  |  |  |  |  |  |  |  |  |  |
|  |  |  |  |  |  |  |  |  |  |  |  |  |  |  |  |  |  |  |  |  |  |  |  |  |  |  |  |  |  |  |  |  |  |  |  |  |  |  |  |  |  |  |
|  |  |  |  |  |  |  |  |  |  |  |  |  |  |  |  |  |  |  |  |  |  |  |  |  |  |  |  |  |  |  |  |  |  |  |  |  |  |  |  |  |  |  |
|  |  |  |  |  |  |  |  |  |  |  |  |  |  |  |  |  |  |  |  |  |  |  |  |  |  |  |  |  |  |  |  |  |  |  |  |  |  |  |  |  |  |  |
|  |  |  |  |  |  |  |  |  |  |  |  |  |  |  |  |  |  |  |  |  |  |  |  |  |  |  |  |  |  |  |  |  |  |  |  |  |  |  |  |  |  |  |
|  |  |  |  |  |  |  |  |  |  |  |  |  |  |  |  |  |  |  |  |  |  |  |  |  |  |  |  |  |  |  |  |  |  |  |  |  |  |  |  |  |  |  |
|  |  |  |  |  |  |  |  |  |  |  |  |  |  |  |  |  |  |  |  |  |  |  |  |  |  |  |  |  |  |  |  |  |  |  |  |  |  |  |  |  |  |  |
|  |  |  |  |  |  |  |  |  |  |  |  |  |  |  |  |  |  |  |  |  |  |  |  |  |  |  |  |  |  |  |  |  |  |  |  |  |  |  |  |  |  |  |
|  |  |  |  |  |  |  |  |  |  |  |  |  |  |  |  |  |  |  |  |  |  |  |  |  |  |  |  |  |  |  |  |  |  |  |  |  |  |  |  |  |  |  |
|  |  |  |  |  |  |  |  |  |  |  |  |  |  |  |  |  |  |  |  |  |  |  |  |  |  |  |  |  |  |  |  |  |  |  |  |  |  |  |  |  |  |  |
|  |  |  |  |  |  |  |  |  |  |  |  |  |  |  |  |  |  |  |  |  |  |  |  |  |  |  |  |  |  |  |  |  |  |  |  |  |  |  |  |  |  |  |
|  |  |  |  |  |  |  |  |  |  |  |  |  |  |  |  |  |  |  |  |  |  |  |  |  |  |  |  |  |  |  |  |  |  |  |  |  |  |  |  |  |  |  |
|  |  |  |  |  |  |  |  |  |  |  |  |  |  |  |  |  |  |  |  |  |  |  |  |  |  |  |  |  |  |  |  |  |  |  |  |  |  |  |  |  |  |  |
|  |  |  |  |  |  |  |  |  |  |  |  |  |  |  |  |  |  |  |  |  |  |  |  |  |  |  |  |  |  |  |  |  |  |  |  |  |  |  |  |  |  |  |
|  |  |  |  |  |  |  |  |  |  |  |  |  |  |  |  |  |  |  |  |  |  |  |  |  |  |  |  |  |  |  |  |  |  |  |  |  |  |  |  |  |  |  |
|  |  |  |  |  |  |  |  |  |  |  |  |  |  |  |  |  |  |  |  |  |  |  |  |  |  |  |  |  |  |  |  |  |  |  |  |  |  |  |  |  |  |  |
|  |  |  |  |  |  |  |  |  |  |  |  |  |  |  |  |  |  |  |  |  |  |  |  |  |  |  |  |  |  |  |  |  |  |  |  |  |  |  |  |  |  |  |
|  |  |  |  |  |  |  |  |  |  |  |  |  |  |  |  |  |  |  |  |  |  |  |  |  |  |  |  |  |  |  |  |  |  |  |  |  |  |  |  |  |  |  |
|  |  |  |  |  |  |  |  |  |  |  |  |  |  |  |  |  |  |  |  |  |  |  |  |  |  |  |  |  |  |  |  |  |  |  |  |  |  |  |  |  |  |  |
|  |  |  |  |  |  |  |  |  |  |  |  |  |  |  |  |  |  |  |  |  |  |  |  |  |  |  |  |  |  |  |  |  |  |  |  |  |  |  |  |  |  |  |
|  |  |  |  |  |  |  |  |  |  |  |  |  |  |  |  |  |  |  |  |  |  |  |  |  |  |  |  |  |  |  |  |  |  |  |  |  |  |  |  |  |  |  |
|  |  |  |  |  |  |  |  |  |  |  |  |  |  |  |  |  |  |  |  |  |  |  |  |  |  |  |  |  |  |  |  |  |  |  |  |  |  |  |  |  |  |  |
|  |  |  |  |  |  |  |  |  |  |  |  |  |  |  |  |  |  |  |  |  |  |  |  |  |  |  |  |  |  |  |  |  |  |  |  |  |  |  |  |  |  |  |
|  |  |  |  |  |  |  |  |  |  |  |  |  |  |  |  |  |  |  |  |  |  |  |  |  |  |  |  |  |  |  |  |  |  |  |  |  |  |  |  |  |  |  |
|  |  |  |  |  |  |  |  |  |  |  |  |  |  |  |  |  |  |  |  |  |  |  |  |  |  |  |  |  |  |  |  |  |  |  |  |  |  |  |  |  |  |  |
|  |  |  |  |  |  |  |  |  |  |  |  |  |  |  |  |  |  |  |  |  |  |  |  |  |  |  |  |  |  |  |  |  |  |  |  |  |  |  |  |  |  |  |
|  |  |  |  |  |  |  |  |  |  |  |  |  |  |  |  |  |  |  |  |  |  |  |  |  |  |  |  |  |  |  |  |  |  |  |  |  |  |  |  |  |  |  |
|  |  |  |  |  |  |  |  |  |  |  |  |  |  |  |  |  |  |  |  |  |  |  |  |  |  |  |  |  |  |  |  |  |  |  |  |  |  |  |  |  |  |  |
|  |  |  |  |  |  |  |  |  |  |  |  |  |  |  |  |  |  |  |  |  |  |  |  |  |  |  |  |  |  |  |  |  |  |  |  |  |  |  |  |  |  |  |
|  |  |  |  |  |  |  |  |  |  |  |  |  |  |  |  |  |  |  |  |  |  |  |  |  |  |  |  |  |  |  |  |  |  |  |  |  |  |  |  |  |  |  |
|  |  |  |  |  |  |  |  |  |  |  |  |  |  |  |  |  |  |  |  |  |  |  |  |  |  |  |  |  |  |  |  |  |  |  |  |  |  |  |  |  |  |  |
|  |  |  |  |  |  |  |  |  |  |  |  |  |  |  |  |  |  |  |  |  |  |  |  |  |  |  |  |  |  |  |  |  |  |  |  |  |  |  |  |  |  |  |
|  |  |  |  |  |  |  |  |  |  |  |  |  |  |  |  |  |  |  |  |  |  |  |  |  |  |  |  |  |  |  |  |  |  |  |  |  |  |  |  |  |  |  |
|  |  |  |  |  |  |  |  |  |  |  |  |  |  |  |  |  |  |  |  |  |  |  |  |  |  |  |  |  |  |  |  |  |  |  |  |  |  |  |  |  |  |  |
|  |  |  |  |  |  |  |  |  |  |  |  |  |  |  |  |  |  |  |  |  |  |  |  |  |  |  |  |  |  |  |  |  |  |  |  |  |  |  |  |  |  |  |
|  |  |  |  |  |  |  |  |  |  |  |  |  |  |  |  |  |  |  |  |  |  |  |  |  |  |  |  |  |  |  |  |  |  |  |  |  |  |  |  |  |  |  |
|  |  |  |  |  |  |  |  |  |  |  |  |  |  |  |  |  |  |  |  |  |  |  |  |  |  |  |  |  |  |  |  |  |  |  |  |  |  |  |  |  |  |  |
|  |  |  |  |  |  |  |  |  |  |  |  |  |  |  |  |  |  |  |  |  |  |  |  |  |  |  |  |  |  |  |  |  |  |  |  |  |  |  |  |  |  |  |
|  |  |  |  |  |  |  |  |  |  |  |  |  |  |  |  |  |  |  |  |  |  |  |  |  |  |  |  |  |  |  |  |  |  |  |  |  |  |  |  |  |  |  |
|  |  |  |  |  |  |  |  |  |  |  |  |  |  |  |  |  |  |  |  |  |  |  |  |  |  |  |  |  |  |  |  |  |  |  |  |  |  |  |  |  |  |  |
|  |  |  |  |  |  |  |  |  |  |  |  |  |  |  |  |  |  |  |  |  |  |  |  |  |  |  |  |  |  |  |  |  |  |  |  |  |  |  |  |  |  |  |
|  |  |  |  |  |  |  |  |  |  |  |  |  |  |  |  |  |  |  |  |  |  |  |  |  |  |  |  |  |  |  |  |  |  |  |  |  |  |  |  |  |  |  |
|  |  |  |  |  |  |  |  |  |  |  |  |  |  |  |  |  |  |  |  |  |  |  |  |  |  |  |  |  |  |  |  |  |  |  |  |  |  |  |  |  |  |  |
|  |  |  |  |  |  |  |  |  |  |  |  |  |  |  |  |  |  |  |  |  |  |  |  |  |  |  |  |  |  |  |  |  |  |  |  |  |  |  |  |  |  |  |
|  |  |  |  |  |  |  |  |  |  |  |  |  |  |  |  |  |  |  |  |  |  |  |  |  |  |  |  |  |  |  |  |  |  |  |  |  |  |  |  |  |  |  |
|  |  |  |  |  |  |  |  |  |  |  |  |  |  |  |  |  |  |  |  |  |  |  |  |  |  |  |  |  |  |  |  |  |  |  |  |  |  |  |  |  |  |  |
|  |  |  |  |  |  |  |  |  |  |  |  |  |  |  |  |  |  |  |  |  |  |  |  |  |  |  |  |  |  |  |  |  |  |  |  |  |  |  |  |  |  |  |
|  |  |  |  |  |  |  |  |  |  |  |  |  |  |  |  |  |  |  |  |  |  |  |  |  |  |  |  |  |  |  |  |  |  |  |  |  |  |  |  |  |  |  |
|  |  |  |  |  |  |  |  |  |  |  |  |  |  |  |  |  |  |  |  |  |  |  |  |  |  |  |  |  |  |  |  |  |  |  |  |  |  |  |  |  |  |  |
|  |  |  |  |  |  |  |  |  |  |  |  |  |  |  |  |  |  |  |  |  |  |  |  |  |  |  |  |  |  |  |  |  |  |  |  |  |  |  |  |  |  |  |
|  |  |  |  |  |  |  |  |  |  |  |  |  |  |  |  |  |  |  |  |  |  |  |  |  |  |  |  |  |  |  |  |  |  |  |  |  |  |  |  |  |  |  |
|  |  |  |  |  |  |  |  |  |  |  |  |  |  |  |  |  |  |  |  |  |  |  |  |  |  |  |  |  |  |  |  |  |  |  |  |  |  |  |  |  |  |  |
|  |  |  |  |  |  |  |  |  |  |  |  |  |  |  |  |  |  |  |  |  |  |  |  |  |  |  |  |  |  |  |  |  |  |  |  |  |  |  |  |  |  |  |
|  |  |  |  |  |  |  |  |  |  |  |  |  |  |  |  |  |  |  |  |  |  |  |  |  |  |  |  |  |  |  |  |  |  |  |  |  |  |  |  |  |  |  |
|  |  |  |  |  |  |  |  |  |  |  |  |  |  |  |  |  |  |  |  |  |  |  |  |  |  |  |  |  |  |  |  |  |  |  |  |  |  |  |  |  |  |  |
|  |  |  |  |  |  |  |  |  |  |  |  |  |  |  |  |  |  |  |  |  |  |  |  |  |  |  |  |  |  |  |  |  |  |  |  |  |  |  |  |  |  |  |
|  |  |  |  |  |  |  |  |  |  |  |  |  |  |  |  |  |  |  |  |  |  |  |  |  |  |  |  |  |  |  |  |  |  |  |  |  |  |  |  |  |  |  |
|  |  |  |  |  |  |  |  |  |  |  |  |  |  |  |  |  |  |  |  |  |  |  |  |  |  |  |  |  |  |  |  |  |  |  |  |  |  |  |  |  |  |  |
|  |  |  |  |  |  |  |  |  |  |  |  |  |  |  |  |  |  |  |  |  |  |  |  |  |  |  |  |  |  |  |  |  |  |  |  |  |  |  |  |  |  |  |
|  |  |  |  |  |  |  |  |  |  |  |  |  |  |  |  |  |  |  |  |  |  |  |  |  |  |  |  |  |  |  |  |  |  |  |  |  |  |  |  |  |  |  |
|  |  |  |  |  |  |  |  |  |  |  |  |  |  |  |  |  |  |  |  |  |  |  |  |  |  |  |  |  |  |  |  |  |  |  |  |  |  |  |  |  |  |  |
|  |  |  |  |  |  |  |  |  |  |  |  |  |  |  |  |  |  |  |  |  |  |  |  |  |  |  |  |  |  |  |  |  |  |  |  |  |  |  |  |  |  |  |
|  |  |  |  |  |  |  |  |  |  |  |  |  |  |  |  |  |  |  |  |  |  |  |  |  |  |  |  |  |  |  |  |  |  |  |  |  |  |  |  |  |  |  |
|  |  |  |  |  |  |  |  |  |  |  |  |  |  |  |  |  |  |  |  |  |  |  |  |  |  |  |  |  |  |  |  |  |  |  |  |  |  |  |  |  |  |  |
|  |  |  |  |  |  |  |  |  |  |  |  |  |  |  |  |  |  |  |  |  |  |  |  |  |  |  |  |  |  |  |  |  |  |  |  |  |  |  |  |  |  |  |
|  |  |  |  |  |  |  |  |  |  |  |  |  |  |  |  |  |  |  |  |  |  |  |  |  |  |  |  |  |  |  |  |  |  |  |  |  |  |  |  |  |  |  |
|  |  |  |  |  |  |  |  |  |  |  |  |  |  |  |  |  |  |  |  |  |  |  |  |  |  |  |  |  |  |  |  |  |  |  |  |  |  |  |  |  |  |  |
|  |  |  |  |  |  |  |  |  |  |  |  |  |  |  |  |  |  |  |  |  |  |  |  |  |  |  |  |  |  |  |  |  |  |  |  |  |  |  |  |  |  |  |
|  |  |  |  |  |  |  |  |  |  |  |  |  |  |  |  |  |  |  |  |  |  |  |  |  |  |  |  |  |  |  |  |  |  |  |  |  |  |  |  |  |  |  |
|  |  |  |  |  |  |  |  |  |  |  |  |  |  |  |  |  |  |  |  |  |  |  |  |  |  |  |  |  |  |  |  |  |  |  |  |  |  |  |  |  |  |  |
|  |  |  |  |  |  |  |  |  |  |  |  |  |  |  |  |  |  |  |  |  |  |  |  |  |  |  |  |  |  |  |  |  |  |  |  |  |  |  |  |  |  |  |
|  |  |  |  |  |  |  |  |  |  |  |  |  |  |  |  |  |  |  |  |  |  |  |  |  |  |  |  |  |  |  |  |  |  |  |  |  |  |  |  |  |  |  |
|  |  |  |  |  |  |  |  |  |  |  |  |  |  |  |  |  |  |  |  |  |  |  |  |  |  |  |  |  |  |  |  |  |  |  |  |  |  |  |  |  |  |  |
|  |  |  |  |  |  |  |  |  |  |  |  |  |  |  |  |  |  |  |  |  |  |  |  |  |  |  |  |  |  |  |  |  |  |  |  |  |  |  |  |  |  |  |
|  |  |  |  |  |  |  |  |  |  |  |  |  |  |  |  |  |  |  |  |  |  |  |  |  |  |  |  |  |  |  |  |  |  |  |  |  |  |  |  |  |  |  |
|  |  |  |  |  |  |  |  |  |  |  |  |  |  |  |  |  |  |  |  |  |  |  |  |  |  |  |  |  |  |  |  |  |  |  |  |  |  |  |  |  |  |  |
|  |  |  |  |  |  |  |  |  |  |  |  |  |  |  |  |  |  |  |  |  |  |  |  |  |  |  |  |  |  |  |  |  |  |  |  |  |  |  |  |  |  |  |
|  |  |  |  |  |  |  |  |  |  |  |  |  |  |  |  |  |  |  |  |  |  |  |  |  |  |  |  |  |  |  |  |  |  |  |  |  |  |  |  |  |  |  |
|  |  |  |  |  |  |  |  |  |  |  |  |  |  |  |  |  |  |  |  |  |  |  |  |  |  |  |  |  |  |  |  |  |  |  |  |  |  |  |  |  |  |  |
|  |  |  |  |  |  |  |  |  |  |  |  |  |  |  |  |  |  |  |  |  |  |  |  |  |  |  |  |  |  |  |  |  |  |  |  |  |  |  |  |  |  |  |
|  |  |  |  |  |  |  |  |  |  |  |  |  |  |  |  |  |  |  |  |  |  |  |  |  |  |  |  |  |  |  |  |  |  |  |  |  |  |  |  |  |  |  |
|  |  |  |  |  |  |  |  |  |  |  |  |  |  |  |  |  |  |  |  |  |  |  |  |  |  |  |  |  |  |  |  |  |  |  |  |  |  |  |  |  |  |  |
|  |  |  |  |  |  |  |  |  |  |  |  |  |  |  |  |  |  |  |  |  |  |  |  |  |  |  |  |  |  |  |  |  |  |  |  |  |  |  |  |  |  |  |
|  |  |  |  |  |  |  |  |  |  |  |  |  |  |  |  |  |  |  |  |  |  |  |  |  |  |  |  |  |  |  |  |  |  |  |  |  |  |  |  |  |  |  |
|  |  |  |  |  |  |  |  |  |  |  |  |  |  |  |  |  |  |  |  |  |  |  |  |  |  |  |  |  |  |  |  |  |  |  |  |  |  |  |  |  |  |  |
|  |  |  |  |  |  |  |  |  |  |  |  |  |  |  |  |  |  |  |  |  |  |  |  |  |  |  |  |  |  |  |  |  |  |  |  |  |  |  |  |  |  |  |
|  |  |  |  |  |  |  |  |  |  |  |  |  |  |  |  |  |  |  |  |  |  |  |  |  |  |  |  |  |  |  |  |  |  |  |  |  |  |  |  |  |  |  |
|  |  |  |  |  |  |  |  |  |  |  |  |  |  |  |  |  |  |  |  |  |  |  |  |  |  |  |  |  |  |  |  |  |  |  |  |  |  |  |  |  |  |  |
|  |  |  |  |  |  |  |  |  |  |  |  |  |  |  |  |  |  |  |  |  |  |  |  |  |  |  |  |  |  |  |  |  |  |  |  |  |  |  |  |  |  |  |
|  |  |  |  |  |  |  |  |  |  |  |  |  |  |  |  |  |  |  |  |  |  |  |  |  |  |  |  |  |  |  |  |  |  |  |  |  |  |  |  |  |  |  |
|  |  |  |  |  |  |  |  |  |  |  |  |  |  |  |  |  |  |  |  |  |  |  |  |  |  |  |  |  |  |  |  |  |  |  |  |  |  |  |  |  |  |  |
|  |  |  |  |  |  |  |  |  |  |  |  |  |  |  |  |  |  |  |  |  |  |  |  |  |  |  |  |  |  |  |  |  |  |  |  |  |  |  |  |  |  |  |
|  |  |  |  |  |  |  |  |  |  |  |  |  |  |  |  |  |  |  |  |  |  |  |  |  |  |  |  |  |  |  |  |  |  |  |  |  |  |  |  |  |  |  |
|  |  |  |  |  |  |  |  |  |  |  |  |  |  |  |  |  |  |  |  |  |  |  |  |  |  |  |  |  |  |  |  |  |  |  |  |  |  |  |  |  |  |  |
|  |  |  |  |  |  |  |  |  |  |  |  |  |  |  |  |  |  |  |  |  |  |  |  |  |  |  |  |  |  |  |  |  |  |  |  |  |  |  |  |  |  |  |
|  |  |  |  |  |  |  |  |  |  |  |  |  |  |  |  |  |  |  |  |  |  |  |  |  |  |  |  |  |  |  |  |  |  |  |  |  |  |  |  |  |  |  |
|  |  |  |  |  |  |  |  |  |  |  |  |  |  |  |  |  |  |  |  |  |  |  |  |  |  |  |  |  |  |  |  |  |  |  |  |  |  |  |  |  |  |  |
|  |  |  |  |  |  |  |  |  |  |  |  |  |  |  |  |  |  |  |  |  |  |  |  |  |  |  |  |  |  |  |  |  |  |  |  |  |  |  |  |  |  |  |
|  |  |  |  |  |  |  |  |  |  |  |  |  |  |  |  |  |  |  |  |  |  |  |  |  |  |  |  |  |  |  |  |  |  |  |  |  |  |  |  |  |  |  |
|  |  |  |  |  |  |  |  |  |  |  |  |  |  |  |  |  |  |  |  |  |  |  |  |  |  |  |  |  |  |  |  |  |  |  |  |  |  |  |  |  |  |  |
|  |  |  |  |  |  |  |  |  |  |  |  |  |  |  |  |  |  |  |  |  |  |  |  |  |  |  |  |  |  |  |  |  |  |  |  |  |  |  |  |  |  |  |
|  |  |  |  |  |  |  |  |  |  |  |  |  |  |  |  |  |  |  |  |  |  |  |  |  |  |  |  |  |  |  |  |  |  |  |  |  |  |  |  |  |  |  |
|  |  |  |  |  |  |  |  |  |  |  |  |  |  |  |  |  |  |  |  |  |  |  |  |  |  |  |  |  |  |  |  |  |  |  |  |  |  |  |  |  |  |  |
|  |  |  |  |  |  |  |  |  |  |  |  |  |  |  |  |  |  |  |  |  |  |  |  |  |  |  |  |  |  |  |  |  |  |  |  |  |  |  |  |  |  |  |
|  |  |  |  |  |  |  |  |  |  |  |  |  |  |  |  |  |  |  |  |  |  |  |  |  |  |  |  |  |  |  |  |  |  |  |  |  |  |  |  |  |  |  |
|  |  |  |  |  |  |  |  |  |  |  |  |  |  |  |  |  |  |  |  |  |  |  |  |  |  |  |  |  |  |  |  |  |  |  |  |  |  |  |  |  |  |  |
|  |  |  |  |  |  |  |  |  |  |  |  |  |  |  |  |  |  |  |  |  |  |  |  |  |  |  |  |  |  |  |  |  |  |  |  |  |  |  |  |  |  |  |
|  |  |  |  |  |  |  |  |  |  |  |  |  |  |  |  |  |  |  |  |  |  |  |  |  |  |  |  |  |  |  |  |  |  |  |  |  |  |  |  |  |  |  |
|  |  |  |  |  |  |  |  |  |  |  |  |  |  |  |  |  |  |  |  |  |  |  |  |  |  |  |  |  |  |  |  |  |  |  |  |  |  |  |  |  |  |  |
|  |  |  |  |  |  |  |  |  |  |  |  |  |  |  |  |  |  |  |  |  |  |  |  |  |  |  |  |  |  |  |  |  |  |  |  |  |  |  |  |  |  |  |
|  |  |  |  |  |  |  |  |  |  |  |  |  |  |  |  |  |  |  |  |  |  |  |  |  |  |  |  |  |  |  |  |  |  |  |  |  |  |  |  |  |  |  |
|  |  |  |  |  |  |  |  |  |  |  |  |  |  |  |  |  |  |  |  |  |  |  |  |  |  |  |  |  |  |  |  |  |  |  |  |  |  |  |  |  |  |  |
|  |  |  |  |  |  |  |  |  |  |  |  |  |  |  |  |  |  |  |  |  |  |  |  |  |  |  |  |  |  |  |  |  |  |  |  |  |  |  |  |  |  |  |
|  |  |  |  |  |  |  |  |  |  |  |  |  |  |  |  |  |  |  |  |  |  |  |  |  |  |  |  |  |  |  |  |  |  |  |  |  |  |  |  |  |  |  |
|  |  |  |  |  |  |  |  |  |  |  |  |  |  |  |  |  |  |  |  |  |  |  |  |  |  |  |  |  |  |  |  |  |  |  |  |  |  |  |  |  |  |  |
|  |  |  |  |  |  |  |  |  |  |  |  |  |  |  |  |  |  |  |  |  |  |  |  |  |  |  |  |  |  |  |  |  |  |  |  |  |  |  |  |  |  |  |
|  |  |  |  |  |  |  |  |  |  |  |  |  |  |  |  |  |  |  |  |  |  |  |  |  |  |  |  |  |  |  |  |  |  |  |  |  |  |  |  |  |  |  |
|  |  |  |  |  |  |  |  |  |  |  |  |  |  |  |  |  |  |  |  |  |  |  |  |  |  |  |  |  |  |  |  |  |  |  |  |  |  |  |  |  |  |  |
|  |  |  |  |  |  |  |  |  |  |  |  |  |  |  |  |  |  |  |  |  |  |  |  |  |  |  |  |  |  |  |  |  |  |  |  |  |  |  |  |  |  |  |
|  |  |  |  |  |  |  |  |  |  |  |  |  |  |  |  |  |  |  |  |  |  |  |  |  |  |  |  |  |  |  |  |  |  |  |  |  |  |  |  |  |  |  |
|  |  |  |  |  |  |  |  |  |  |  |  |  |  |  |  |  |  |  |  |  |  |  |  |  |  |  |  |  |  |  |  |  |  |  |  |  |  |  |  |  |  |  |
|  |  |  |  |  |  |  |  |  |  |  |  |  |  |  |  |  |  |  |  |  |  |  |  |  |  |  |  |  |  |  |  |  |  |  |  |  |  |  |  |  |  |  |
|  |  |  |  |  |  |  |  |  |  |  |  |  |  |  |  |  |  |  |  |  |  |  |  |  |  |  |  |  |  |  |  |  |  |  |  |  |  |  |  |  |  |  |
|  |  |  |  |  |  |  |  |  |  |  |  |  |  |  |  |  |  |  |  |  |  |  |  |  |  |  |  |  |  |  |  |  |  |  |  |  |  |  |  |  |  |  |
|  |  |  |  |  |  |  |  |  |  |  |  |  |  |  |  |  |  |  |  |  |  |  |  |  |  |  |  |  |  |  |  |  |  |  |  |  |  |  |  |  |  |  |
|  |  |  |  |  |  |  |  |  |  |  |  |  |  |  |  |  |  |  |  |  |  |  |  |  |  |  |  |  |  |  |  |  |  |  |  |  |  |  |  |  |  |  |
|  |  |  |  |  |  |  |  |  |  |  |  |  |  |  |  |  |  |  |  |  |  |  |  |  |  |  |  |  |  |  |  |  |  |  |  |  |  |  |  |  |  |  |
|  |  |  |  |  |  |  |  |  |  |  |  |  |  |  |  |  |  |  |  |  |  |  |  |  |  |  |  |  |  |  |  |  |  |  |  |  |  |  |  |  |  |  |
|  |  |  |  |  |  |  |  |  |  |  |  |  |  |  |  |  |  |  |  |  |  |  |  |  |  |  |  |  |  |  |  |  |  |  |  |  |  |  |  |  |  |  |
|  |  |  |  |  |  |  |  |  |  |  |  |  |  |  |  |  |  |  |  |  |  |  |  |  |  |  |  |  |  |  |  |  |  |  |  |  |  |  |  |  |  |  |
|  |  |  |  |  |  |  |  |  |  |  |  |  |  |  |  |  |  |  |  |  |  |  |  |  |  |  |  |  |  |  |  |  |  |  |  |  |  |  |  |  |  |  |
|  |  |  |  |  |  |  |  |  |  |  |  |  |  |  |  |  |  |  |  |  |  |  |  |  |  |  |  |  |  |  |  |  |  |  |  |  |  |  |  |  |  |  |
|  |  |  |  |  |  |  |  |  |  |  |  |  |  |  |  |  |  |  |  |  |  |  |  |  |  |  |  |  |  |  |  |  |  |  |  |  |  |  |  |  |  |  |
|  |  |  |  |  |  |  |  |  |  |  |  |  |  |  |  |  |  |  |  |  |  |  |  |  |  |  |  |  |  |  |  |  |  |  |  |  |  |  |  |  |  |  |
|  |  |  |  |  |  |  |  |  |  |  |  |  |  |  |  |  |  |  |  |  |  |  |  |  |  |  |  |  |  |  |  |  |  |  |  |  |  |  |  |  |  |  |
|  |  |  |  |  |  |  |  |  |  |  |  |  |  |  |  |  |  |  |  |  |  |  |  |  |  |  |  |  |  |  |  |  |  |  |  |  |  |  |  |  |  |  |
|  |  |  |  |  |  |  |  |  |  |  |  |  |  |  |  |  |  |  |  |  |  |  |  |  |  |  |  |  |  |  |  |  |  |  |  |  |  |  |  |  |  |  |
|  |  |  |  |  |  |  |  |  |  |  |  |  |  |  |  |  |  |  |  |  |  |  |  |  |  |  |  |  |  |  |  |  |  |  |  |  |  |  |  |  |  |  |
|  |  |  |  |  |  |  |  |  |  |  |  |  |  |  |  |  |  |  |  |  |  |  |  |  |  |  |  |  |  |  |  |  |  |  |  |  |  |  |  |  |  |  |
|  |  |  |  |  |  |  |  |  |  |  |  |  |  |  |  |  |  |  |  |  |  |  |  |  |  |  |  |  |  |  |  |  |  |  |  |  |  |  |  |  |  |  |
|  |  |  |  |  |  |  |  |  |  |  |  |  |  |  |  |  |  |  |  |  |  |  |  |  |  |  |  |  |  |  |  |  |  |  |  |  |  |  |  |  |  |  |
|  |  |  |  |  |  |  |  |  |  |  |  |  |  |  |  |  |  |  |  |  |  |  |  |  |  |  |  |  |  |  |  |  |  |  |  |  |  |  |  |  |  |  |
|  |  |  |  |  |  |  |  |  |  |  |  |  |  |  |  |  |  |  |  |  |  |  |  |  |  |  |  |  |  |  |  |  |  |  |  |  |  |  |  |  |  |  |
|  |  |  |  |  |  |  |  |  |  |  |  |  |  |  |  |  |  |  |  |  |  |  |  |  |  |  |  |  |  |  |  |  |  |  |  |  |  |  |  |  |  |  |
|  |  |  |  |  |  |  |  |  |  |  |  |  |  |  |  |  |  |  |  |  |  |  |  |  |  |  |  |  |  |  |  |  |  |  |  |  |  |  |  |  |  |  |
|  |  |  |  |  |  |  |  |  |  |  |  |  |  |  |  |  |  |  |  |  |  |  |  |  |  |  |  |  |  |  |  |  |  |  |  |  |  |  |  |  |  |  |
|  |  |  |  |  |  |  |  |  |  |  |  |  |  |  |  |  |  |  |  |  |  |  |  |  |  |  |  |  |  |  |  |  |  |  |  |  |  |  |  |  |  |  |
|  |  |  |  |  |  |  |  |  |  |  |  |  |  |  |  |  |  |  |  |  |  |  |  |  |  |  |  |  |  |  |  |  |  |  |  |  |  |  |  |  |  |  |
|  |  |  |  |  |  |  |  |  |  |  |  |  |  |  |  |  |  |  |  |  |  |  |  |  |  |  |  |  |  |  |  |  |  |  |  |  |  |  |  |  |  |  |
|  |  |  |  |  |  |  |  |  |  |  |  |  |  |  |  |  |  |  |  |  |  |  |  |  |  |  |  |  |  |  |  |  |  |  |  |  |  |  |  |  |  |  |
|  |  |  |  |  |  |  |  |  |  |  |  |  |  |  |  |  |  |  |  |  |  |  |  |  |  |  |  |  |  |  |  |  |  |  |  |  |  |  |  |  |  |  |
|  |  |  |  |  |  |  |  |  |  |  |  |  |  |  |  |  |  |  |  |  |  |  |  |  |  |  |  |  |  |  |  |  |  |  |  |  |  |  |  |  |  |  |
|  |  |  |  |  |  |  |  |  |  |  |  |  |  |  |  |  |  |  |  |  |  |  |  |  |  |  |  |  |  |  |  |  |  |  |  |  |  |  |  |  |  |  |
|  |  |  |  |  |  |  |  |  |  |  |  |  |  |  |  |  |  |  |  |  |  |  |  |  |  |  |  |  |  |  |  |  |  |  |  |  |  |  |  |  |  |  |
|  |  |  |  |  |  |  |  |  |  |  |  |  |  |  |  |  |  |  |  |  |  |  |  |  |  |  |  |  |  |  |  |  |  |  |  |  |  |  |  |  |  |  |
|  |  |  |  |  |  |  |  |  |  |  |  |  |  |  |  |  |  |  |  |  |  |  |  |  |  |  |  |  |  |  |  |  |  |  |  |  |  |  |  |  |  |  |
|  |  |  |  |  |  |  |  |  |  |  |  |  |  |  |  |  |  |  |  |  |  |  |  |  |  |  |  |  |  |  |  |  |  |  |  |  |  |  |  |  |  |  |
|  |  |  |  |  |  |  |  |  |  |  |  |  |  |  |  |  |  |  |  |  |  |  |  |  |  |  |  |  |  |  |  |  |  |  |  |  |  |  |  |  |  |  |
|  |  |  |  |  |  |  |  |  |  |  |  |  |  |  |  |  |  |  |  |  |  |  |  |  |  |  |  |  |  |  |  |  |  |  |  |  |  |  |  |  |  |  |
|  |  |  |  |  |  |  |  |  |  |  |  |  |  |  |  |  |  |  |  |  |  |  |  |  |  |  |  |  |  |  |  |  |  |  |  |  |  |  |  |  |  |  |
|  |  |  |  |  |  |  |  |  |  |  |  |  |  |  |  |  |  |  |  |  |  |  |  |  |  |  |  |  |  |  |  |  |  |  |  |  |  |  |  |  |  |  |
|  |  |  |  |  |  |  |  |  |  |  |  |  |  |  |  |  |  |  |  |  |  |  |  |  |  |  |  |  |  |  |  |  |  |  |  |  |  |  |  |  |  |  |
|  |  |  |  |  |  |  |  |  |  |  |  |  |  |  |  |  |  |  |  |  |  |  |  |  |  |  |  |  |  |  |  |  |  |  |  |  |  |  |  |  |  |  |
|  |  |  |  |  |  |  |  |  |  |  |  |  |  |  |  |  |  |  |  |  |  |  |  |  |  |  |  |  |  |  |  |  |  |  |  |  |  |  |  |  |  |  |
|  |  |  |  |  |  |  |  |  |  |  |  |  |  |  |  |  |  |  |  |  |  |  |  |  |  |  |  |  |  |  |  |  |  |  |  |  |  |  |  |  |  |  |
|  |  |  |  |  |  |  |  |  |  |  |  |  |  |  |  |  |  |  |  |  |  |  |  |  |  |  |  |  |  |  |  |  |  |  |  |  |  |  |  |  |  |  |
|  |  |  |  |  |  |  |  |  |  |  |  |  |  |  |  |  |  |  |  |  |  |  |  |  |  |  |  |  |  |  |  |  |  |  |  |  |  |  |  |  |  |  |
|  |  |  |  |  |  |  |  |  |  |  |  |  |  |  |  |  |  |  |  |  |  |  |  |  |  |  |  |  |  |  |  |  |  |  |  |  |  |  |  |  |  |  |
|  |  |  |  |  |  |  |  |  |  |  |  |  |  |  |  |  |  |  |  |  |  |  |  |  |  |  |  |  |  |  |  |  |  |  |  |  |  |  |  |  |  |  |
|  |  |  |  |  |  |  |  |  |  |  |  |  |  |  |  |  |  |  |  |  |  |  |  |  |  |  |  |  |  |  |  |  |  |  |  |  |  |  |  |  |  |  |
|  |  |  |  |  |  |  |  |  |  |  |  |  |  |  |  |  |  |  |  |  |  |  |  |  |  |  |  |  |  |  |  |  |  |  |  |  |  |  |  |  |  |  |
|  |  |  |  |  |  |  |  |  |  |  |  |  |  |  |  |  |  |  |  |  |  |  |  |  |  |  |  |  |  |  |  |  |  |  |  |  |  |  |  |  |  |  |
|  |  |  |  |  |  |  |  |  |  |  |  |  |  |  |  |  |  |  |  |  |  |  |  |  |  |  |  |  |  |  |  |  |  |  |  |  |  |  |  |  |  |  |
|  |  |  |  |  |  |  |  |  |  |  |  |  |  |  |  |  |  |  |  |  |  |  |  |  |  |  |  |  |  |  |  |  |  |  |  |  |  |  |  |  |  |  |
|  |  |  |  |  |  |  |  |  |  |  |  |  |  |  |  |  |  |  |  |  |  |  |  |  |  |  |  |  |  |  |  |  |  |  |  |  |  |  |  |  |  |  |
|  |  |  |  |  |  |  |  |  |  |  |  |  |  |  |  |  |  |  |  |  |  |  |  |  |  |  |  |  |  |  |  |  |  |  |  |  |  |  |  |  |  |  |
|  |  |  |  |  |  |  |  |  |  |  |  |  |  |  |  |  |  |  |  |  |  |  |  |  |  |  |  |  |  |  |  |  |  |  |  |  |  |  |  |  |  |  |
|  |  |  |  |  |  |  |  |  |  |  |  |  |  |  |  |  |  |  |  |  |  |  |  |  |  |  |  |  |  |  |  |  |  |  |  |  |  |  |  |  |  |  |
|  |  |  |  |  |  |  |  |  |  |  |  |  |  |  |  |  |  |  |  |  |  |  |  |  |  |  |  |  |  |  |  |  |  |  |  |  |  |  |  |  |  |  |
|  |  |  |  |  |  |  |  |  |  |  |  |  |  |  |  |  |  |  |  |  |  |  |  |  |  |  |  |  |  |  |  |  |  |  |  |  |  |  |  |  |  |  |
|  |  |  |  |  |  |  |  |  |  |  |  |  |  |  |  |  |  |  |  |  |  |  |  |  |  |  |  |  |  |  |  |  |  |  |  |  |  |  |  |  |  |  |
|  |  |  |  |  |  |  |  |  |  |  |  |  |  |  |  |  |  |  |  |  |  |  |  |  |  |  |  |  |  |  |  |  |  |  |  |  |  |  |  |  |  |  |
|  |  |  |  |  |  |  |  |  |  |  |  |  |  |  |  |  |  |  |  |  |  |  |  |  |  |  |  |  |  |  |  |  |  |  |  |  |  |  |  |  |  |  |
|  |  |  |  |  |  |  |  |  |  |  |  |  |  |  |  |  |  |  |  |  |  |  |  |  |  |  |  |  |  |  |  |  |  |  |  |  |  |  |  |  |  |  |
|  |  |  |  |  |  |  |  |  |  |  |  |  |  |  |  |  |  |  |  |  |  |  |  |  |  |  |  |  |  |  |  |  |  |  |  |  |  |  |  |  |  |  |
|  |  |  |  |  |  |  |  |  |  |  |  |  |  |  |  |  |  |  |  |  |  |  |  |  |  |  |  |  |  |  |  |  |  |  |  |  |  |  |  |  |  |  |
|  |  |  |  |  |  |  |  |  |  |  |  |  |  |  |  |  |  |  |  |  |  |  |  |  |  |  |  |  |  |  |  |  |  |  |  |  |  |  |  |  |  |  |
|  |  |  |  |  |  |  |  |  |  |  |  |  |  |  |  |  |  |  |  |  |  |  |  |  |  |  |  |  |  |  |  |  |  |  |  |  |  |  |  |  |  |  |
|  |  |  |  |  |  |  |  |  |  |  |  |  |  |  |  |  |  |  |  |  |  |  |  |  |  |  |  |  |  |  |  |  |  |  |  |  |  |  |  |  |  |  |
|  |  |  |  |  |  |  |  |  |  |  |  |  |  |  |  |  |  |  |  |  |  |  |  |  |  |  |  |  |  |  |  |  |  |  |  |  |  |  |  |  |  |  |
|  |  |  |  |  |  |  |  |  |  |  |  |  |  |  |  |  |  |  |  |  |  |  |  |  |  |  |  |  |  |  |  |  |  |  |  |  |  |  |  |  |  |  |
|  |  |  |  |  |  |  |  |  |  |  |  |  |  |  |  |  |  |  |  |  |  |  |  |  |  |  |  |  |  |  |  |  |  |  |  |  |  |  |  |  |  |  |
|  |  |  |  |  |  |  |  |  |  |  |  |  |  |  |  |  |  |  |  |  |  |  |  |  |  |  |  |  |  |  |  |  |  |  |  |  |  |  |  |  |  |  |
|  |  |  |  |  |  |  |  |  |  |  |  |  |  |  |  |  |  |  |  |  |  |  |  |  |  |  |  |  |  |  |  |  |  |  |  |  |  |  |  |  |  |  |
|  |  |  |  |  |  |  |  |  |  |  |  |  |  |  |  |  |  |  |  |  |  |  |  |  |  |  |  |  |  |  |  |  |  |  |  |  |  |  |  |  |  |  |
|  |  |  |  |  |  |  |  |  |  |  |  |  |  |  |  |  |  |  |  |  |  |  |  |  |  |  |  |  |  |  |  |  |  |  |  |  |  |  |  |  |  |  |
|  |  |  |  |  |  |  |  |  |  |  |  |  |  |  |  |  |  |  |  |  |  |  |  |  |  |  |  |  |  |  |  |  |  |  |  |  |  |  |  |  |  |  |
|  |  |  |  |  |  |  |  |  |  |  |  |  |  |  |  |  |  |  |  |  |  |  |  |  |  |  |  |  |  |  |  |  |  |  |  |  |  |  |  |  |  |  |
|  |  |  |  |  |  |  |  |  |  |  |  |  |  |  |  |  |  |  |  |  |  |  |  |  |  |  |  |  |  |  |  |  |  |  |  |  |  |  |  |  |  |  |
|  |  |  |  |  |  |  |  |  |  |  |  |  |  |  |  |  |  |  |  |  |  |  |  |  |  |  |  |  |  |  |  |  |  |  |  |  |  |  |  |  |  |  |
|  |  |  |  |  |  |  |  |  |  |  |  |  |  |  |  |  |  |  |  |  |  |  |  |  |  |  |  |  |  |  |  |  |  |  |  |  |  |  |  |  |  |  |
|  |  |  |  |  |  |  |  |  |  |  |  |  |  |  |  |  |  |  |  |  |  |  |  |  |  |  |  |  |  |  |  |  |  |  |  |  |  |  |  |  |  |  |
|  |  |  |  |  |  |  |  |  |  |  |  |  |  |  |  |  |  |  |  |  |  |  |  |  |  |  |  |  |  |  |  |  |  |  |  |  |  |  |  |  |  |  |
|  |  |  |  |  |  |  |  |  |  |  |  |  |  |  |  |  |  |  |  |  |  |  |  |  |  |  |  |  |  |  |  |  |  |  |  |  |  |  |  |  |  |  |
|  |  |  |  |  |  |  |  |  |  |  |  |  |  |  |  |  |  |  |  |  |  |  |  |  |  |  |  |  |  |  |  |  |  |  |  |  |  |  |  |  |  |  |
|  |  |  |  |  |  |  |  |  |  |  |  |  |  |  |  |  |  |  |  |  |  |  |  |  |  |  |  |  |  |  |  |  |  |  |  |  |  |  |  |  |  |  |
|  |  |  |  |  |  |  |  |  |  |  |  |  |  |  |  |  |  |  |  |  |  |  |  |  |  |  |  |  |  |  |  |  |  |  |  |  |  |  |  |  |  |  |
|  |  |  |  |  |  |  |  |  |  |  |  |  |  |  |  |  |  |  |  |  |  |  |  |  |  |  |  |  |  |  |  |  |  |  |  |  |  |  |  |  |  |  |
|  |  |  |  |  |  |  |  |  |  |  |  |  |  |  |  |  |  |  |  |  |  |  |  |  |  |  |  |  |  |  |  |  |  |  |  |  |  |  |  |  |  |  |
|  |  |  |  |  |  |  |  |  |  |  |  |  |  |  |  |  |  |  |  |  |  |  |  |  |  |  |  |  |  |  |  |  |  |  |  |  |  |  |  |  |  |  |
|  |  |  |  |  |  |  |  |  |  |  |  |  |  |  |  |  |  |  |  |  |  |  |  |  |  |  |  |  |  |  |  |  |  |  |  |  |  |  |  |  |  |  |
|  |  |  |  |  |  |  |  |  |  |  |  |  |  |  |  |  |  |  |  |  |  |  |  |  |  |  |  |  |  |  |  |  |  |  |  |  |  |  |  |  |  |  |
|  |  |  |  |  |  |  |  |  |  |  |  |  |  |  |  |  |  |  |  |  |  |  |  |  |  |  |  |  |  |  |  |  |  |  |  |  |  |  |  |  |  |  |
|  |  |  |  |  |  |  |  |  |  |  |  |  |  |  |  |  |  |  |  |  |  |  |  |  |  |  |  |  |  |  |  |  |  |  |  |  |  |  |  |  |  |  |
|  |  |  |  |  |  |  |  |  |  |  |  |  |  |  |  |  |  |  |  |  |  |  |  |  |  |  |  |  |  |  |  |  |  |  |  |  |  |  |  |  |  |  |
|  |  |  |  |  |  |  |  |  |  |  |  |  |  |  |  |  |  |  |  |  |  |  |  |  |  |  |  |  |  |  |  |  |  |  |  |  |  |  |  |  |  |  |
|  |  |  |  |  |  |  |  |  |  |  |  |  |  |  |  |  |  |  |  |  |  |  |  |  |  |  |  |  |  |  |  |  |  |  |  |  |  |  |  |  |  |  |
|  |  |  |  |  |  |  |  |  |  |  |  |  |  |  |  |  |  |  |  |  |  |  |  |  |  |  |  |  |  |  |  |  |  |  |  |  |  |  |  |  |  |  |
|  |  |  |  |  |  |  |  |  |  |  |  |  |  |  |  |  |  |  |  |  |  |  |  |  |  |  |  |  |  |  |  |  |  |  |  |  |  |  |  |  |  |  |
|  |  |  |  |  |  |  |  |  |  |  |  |  |  |  |  |  |  |  |  |  |  |  |  |  |  |  |  |  |  |  |  |  |  |  |  |  |  |  |  |  |  |  |
|  |  |  |  |  |  |  |  |  |  |  |  |  |  |  |  |  |  |  |  |  |  |  |  |  |  |  |  |  |  |  |  |  |  |  |  |  |  |  |  |  |  |  |
|  |  |  |  |  |  |  |  |  |  |  |  |  |  |  |  |  |  |  |  |  |  |  |  |  |  |  |  |  |  |  |  |  |  |  |  |  |  |  |  |  |  |  |
|  |  |  |  |  |  |  |  |  |  |  |  |  |  |  |  |  |  |  |  |  |  |  |  |  |  |  |  |  |  |  |  |  |  |  |  |  |  |  |  |  |  |  |
|  |  |  |  |  |  |  |  |  |  |  |  |  |  |  |  |  |  |  |  |  |  |  |  |  |  |  |  |  |  |  |  |  |  |  |  |  |  |  |  |  |  |  |
|  |  |  |  |  |  |  |  |  |  |  |  |  |  |  |  |  |  |  |  |  |  |  |  |  |  |  |  |  |  |  |  |  |  |  |  |  |  |  |  |  |  |  |
|  |  |  |  |  |  |  |  |  |  |  |  |  |  |  |  |  |  |  |  |  |  |  |  |  |  |  |  |  |  |  |  |  |  |  |  |  |  |  |  |  |  |  |
|  |  |  |  |  |  |  |  |  |  |  |  |  |  |  |  |  |  |  |  |  |  |  |  |  |  |  |  |  |  |  |  |  |  |  |  |  |  |  |  |  |  |  |
|  |  |  |  |  |  |  |  |  |  |  |  |  |  |  |  |  |  |  |  |  |  |  |  |  |  |  |  |  |  |  |  |  |  |  |  |  |  |  |  |  |  |  |
|  |  |  |  |  |  |  |  |  |  |  |  |  |  |  |  |  |  |  |  |  |  |  |  |  |  |  |  |  |  |  |  |  |  |  |  |  |  |  |  |  |  |  |
|  |  |  |  |  |  |  |  |  |  |  |  |  |  |  |  |  |  |  |  |  |  |  |  |  |  |  |  |  |  |  |  |  |  |  |  |  |  |  |  |  |  |  |
|  |  |  |  |  |  |  |  |  |  |  |  |  |  |  |  |  |  |  |  |  |  |  |  |  |  |  |  |  |  |  |  |  |  |  |  |  |  |  |  |  |  |  |
|  |  |  |  |  |  |  |  |  |  |  |  |  |  |  |  |  |  |  |  |  |  |  |  |  |  |  |  |  |  |  |  |  |  |  |  |  |  |  |  |  |  |  |
|  |  |  |  |  |  |  |  |  |  |  |  |  |  |  |  |  |  |  |  |  |  |  |  |  |  |  |  |  |  |  |  |  |  |  |  |  |  |  |  |  |  |  |
|  |  |  |  |  |  |  |  |  |  |  |  |  |  |  |  |  |  |  |  |  |  |  |  |  |  |  |  |  |  |  |  |  |  |  |  |  |  |  |  |  |  |  |
|  |  |  |  |  |  |  |  |  |  |  |  |  |  |  |  |  |  |  |  |  |  |  |  |  |  |  |  |  |  |  |  |  |  |  |  |  |  |  |  |  |  |  |
|  |  |  |  |  |  |  |  |  |  |  |  |  |  |  |  |  |  |  |  |  |  |  |  |  |  |  |  |  |  |  |  |  |  |  |  |  |  |  |  |  |  |  |
|  |  |  |  |  |  |  |  |  |  |  |  |  |  |  |  |  |  |  |  |  |  |  |  |  |  |  |  |  |  |  |  |  |  |  |  |  |  |  |  |  |  |  |
|  |  |  |  |  |  |  |  |  |  |  |  |  |  |  |  |  |  |  |  |  |  |  |  |  |  |  |  |  |  |  |  |  |  |  |  |  |  |  |  |  |  |  |
|  |  |  |  |  |  |  |  |  |  |  |  |  |  |  |  |  |  |  |  |  |  |  |  |  |  |  |  |  |  |  |  |  |  |  |  |  |  |  |  |  |  |  |
|  |  |  |  |  |  |  |  |  |  |  |  |  |  |  |  |  |  |  |  |  |  |  |  |  |  |  |  |  |  |  |  |  |  |  |  |  |  |  |  |  |  |  |
|  |  |  |  |  |  |  |  |  |  |  |  |  |  |  |  |  |  |  |  |  |  |  |  |  |  |  |  |  |  |  |  |  |  |  |  |  |  |  |  |  |  |  |
|  |  |  |  |  |  |  |  |  |  |  |  |  |  |  |  |  |  |  |  |  |  |  |  |  |  |  |  |  |  |  |  |  |  |  |  |  |  |  |  |  |  |  |
|  |  |  |  |  |  |  |  |  |  |  |  |  |  |  |  |  |  |  |  |  |  |  |  |  |  |  |  |  |  |  |  |  |  |  |  |  |  |  |  |  |  |  |
|  |  |  |  |  |  |  |  |  |  |  |  |  |  |  |  |  |  |  |  |  |  |  |  |  |  |  |  |  |  |  |  |  |  |  |  |  |  |  |  |  |  |  |
|  |  |  |  |  |  |  |  |  |  |  |  |  |  |  |  |  |  |  |  |  |  |  |  |  |  |  |  |  |  |  |  |  |  |  |  |  |  |  |  |  |  |  |
|  |  |  |  |  |  |  |  |  |  |  |  |  |  |  |  |  |  |  |  |  |  |  |  |  |  |  |  |  |  |  |  |  |  |  |  |  |  |  |  |  |  |  |
|  |  |  |  |  |  |  |  |  |  |  |  |  |  |  |  |  |  |  |  |  |  |  |  |  |  |  |  |  |  |  |  |  |  |  |  |  |  |  |  |  |  |  |
|  |  |  |  |  |  |  |  |  |  |  |  |  |  |  |  |  |  |  |  |  |  |  |  |  |  |  |  |  |  |  |  |  |  |  |  |  |  |  |  |  |  |  |
|  |  |  |  |  |  |  |  |  |  |  |  |  |  |  |  |  |  |  |  |  |  |  |  |  |  |  |  |  |  |  |  |  |  |  |  |  |  |  |  |  |  |  |
|  |  |  |  |  |  |  |  |  |  |  |  |  |  |  |  |  |  |  |  |  |  |  |  |  |  |  |  |  |  |  |  |  |  |  |  |  |  |  |  |  |  |  |
|  |  |  |  |  |  |  |  |  |  |  |  |  |  |  |  |  |  |  |  |  |  |  |  |  |  |  |  |  |  |  |  |  |  |  |  |  |  |  |  |  |  |  |
|  |  |  |  |  |  |  |  |  |  |  |  |  |  |  |  |  |  |  |  |  |  |  |  |  |  |  |  |  |  |  |  |  |  |  |  |  |  |  |  |  |  |  |
|  |  |  |  |  |  |  |  |  |  |  |  |  |  |  |  |  |  |  |  |  |  |  |  |  |  |  |  |  |  |  |  |  |  |  |  |  |  |  |  |  |  |  |
|  |  |  |  |  |  |  |  |  |  |  |  |  |  |  |  |  |  |  |  |  |  |  |  |  |  |  |  |  |  |  |  |  |  |  |  |  |  |  |  |  |  |  |
|  |  |  |  |  |  |  |  |  |  |  |  |  |  |  |  |  |  |  |  |  |  |  |  |  |  |  |  |  |  |  |  |  |  |  |  |  |  |  |  |  |  |  |
|  |  |  |  |  |  |  |  |  |  |  |  |  |  |  |  |  |  |  |  |  |  |  |  |  |  |  |  |  |  |  |  |  |  |  |  |  |  |  |  |  |  |  |
|  |  |  |  |  |  |  |  |  |  |  |  |  |  |  |  |  |  |  |  |  |  |  |  |  |  |  |  |  |  |  |  |  |  |  |  |  |  |  |  |  |  |  |
|  |  |  |  |  |  |  |  |  |  |  |  |  |  |  |  |  |  |  |  |  |  |  |  |  |  |  |  |  |  |  |  |  |  |  |  |  |  |  |  |  |  |  |
|  |  |  |  |  |  |  |  |  |  |  |  |  |  |  |  |  |  |  |  |  |  |  |  |  |  |  |  |  |  |  |  |  |  |  |  |  |  |  |  |  |  |  |
|  |  |  |  |  |  |  |  |  |  |  |  |  |  |  |  |  |  |  |  |  |  |  |  |  |  |  |  |  |  |  |  |  |  |  |  |  |  |  |  |  |  |  |
|  |  |  |  |  |  |  |  |  |  |  |  |  |  |  |  |  |  |  |  |  |  |  |  |  |  |  |  |  |  |  |  |  |  |  |  |  |  |  |  |  |  |  |
|  |  |  |  |  |  |  |  |  |  |  |  |  |  |  |  |  |  |  |  |  |  |  |  |  |  |  |  |  |  |  |  |  |  |  |  |  |  |  |  |  |  |  |
|  |  |  |  |  |  |  |  |  |  |  |  |  |  |  |  |  |  |  |  |  |  |  |  |  |  |  |  |  |  |  |  |  |  |  |  |  |  |  |  |  |  |  |
|  |  |  |  |  |  |  |  |  |  |  |  |  |  |  |  |  |  |  |  |  |  |  |  |  |  |  |  |  |  |  |  |  |  |  |  |  |  |  |  |  |  |  |
|  |  |  |  |  |  |  |  |  |  |  |  |  |  |  |  |  |  |  |  |  |  |  |  |  |  |  |  |  |  |  |  |  |  |  |  |  |  |  |  |  |  |  |
|  |  |  |  |  |  |  |  |  |  |  |  |  |  |  |  |  |  |  |  |  |  |  |  |  |  |  |  |  |  |  |  |  |  |  |  |  |  |  |  |  |  |  |
|  |  |  |  |  |  |  |  |  |  |  |  |  |  |  |  |  |  |  |  |  |  |  |  |  |  |  |  |  |  |  |  |  |  |  |  |  |  |  |  |  |  |  |
|  |  |  |  |  |  |  |  |  |  |  |  |  |  |  |  |  |  |  |  |  |  |  |  |  |  |  |  |  |  |  |  |  |  |  |  |  |  |  |  |  |  |  |
|  |  |  |  |  |  |  |  |  |  |  |  |  |  |  |  |  |  |  |  |  |  |  |  |  |  |  |  |  |  |  |  |  |  |  |  |  |  |  |  |  |  |  |
|  |  |  |  |  |  |  |  |  |  |  |  |  |  |  |  |  |  |  |  |  |  |  |  |  |  |  |  |  |  |  |  |  |  |  |  |  |  |  |  |  |  |  |
|  |  |  |  |  |  |  |  |  |  |  |  |  |  |  |  |  |  |  |  |  |  |  |  |  |  |  |  |  |  |  |  |  |  |  |  |  |  |  |  |  |  |  |
|  |  |  |  |  |  |  |  |  |  |  |  |  |  |  |  |  |  |  |  |  |  |  |  |  |  |  |  |  |  |  |  |  |  |  |  |  |  |  |  |  |  |  |
|  |  |  |  |  |  |  |  |  |  |  |  |  |  |  |  |  |  |  |  |  |  |  |  |  |  |  |  |  |  |  |  |  |  |  |  |  |  |  |  |  |  |  |
|  |  |  |  |  |  |  |  |  |  |  |  |  |  |  |  |  |  |  |  |  |  |  |  |  |  |  |  |  |  |  |  |  |  |  |  |  |  |  |  |  |  |  |
|  |  |  |  |  |  |  |  |  |  |  |  |  |  |  |  |  |  |  |  |  |  |  |  |  |  |  |  |  |  |  |  |  |  |  |  |  |  |  |  |  |  |  |
|  |  |  |  |  |  |  |  |  |  |  |  |  |  |  |  |  |  |  |  |  |  |  |  |  |  |  |  |  |  |  |  |  |  |  |  |  |  |  |  |  |  |  |
|  |  |  |  |  |  |  |  |  |  |  |  |  |  |  |  |  |  |  |  |  |  |  |  |  |  |  |  |  |  |  |  |  |  |  |  |  |  |  |  |  |  |  |
|  |  |  |  |  |  |  |  |  |  |  |  |  |  |  |  |  |  |  |  |  |  |  |  |  |  |  |  |  |  |  |  |  |  |  |  |  |  |  |  |  |  |  |
|  |  |  |  |  |  |  |  |  |  |  |  |  |  |  |  |  |  |  |  |  |  |  |  |  |  |  |  |  |  |  |  |  |  |  |  |  |  |  |  |  |  |  |
|  |  |  |  |  |  |  |  |  |  |  |  |  |  |  |  |  |  |  |  |  |  |  |  |  |  |  |  |  |  |  |  |  |  |  |  |  |  |  |  |  |  |  |
|  |  |  |  |  |  |  |  |  |  |  |  |  |  |  |  |  |  |  |  |  |  |  |  |  |  |  |  |  |  |  |  |  |  |  |  |  |  |  |  |  |  |  |
|  |  |  |  |  |  |  |  |  |  |  |  |  |  |  |  |  |  |  |  |  |  |  |  |  |  |  |  |  |  |  |  |  |  |  |  |  |  |  |  |  |  |  |
|  |  |  |  |  |  |  |  |  |  |  |  |  |  |  |  |  |  |  |  |  |  |  |  |  |  |  |  |  |  |  |  |  |  |  |  |  |  |  |  |  |  |  |
|  |  |  |  |  |  |  |  |  |  |  |  |  |  |  |  |  |  |  |  |  |  |  |  |  |  |  |  |  |  |  |  |  |  |  |  |  |  |  |  |  |  |  |
|  |  |  |  |  |  |  |  |  |  |  |  |  |  |  |  |  |  |  |  |  |  |  |  |  |  |  |  |  |  |  |  |  |  |  |  |  |  |  |  |  |  |  |
|  |  |  |  |  |  |  |  |  |  |  |  |  |  |  |  |  |  |  |  |  |  |  |  |  |  |  |  |  |  |  |  |  |  |  |  |  |  |  |  |  |  |  |
|  |  |  |  |  |  |  |  |  |  |  |  |  |  |  |  |  |  |  |  |  |  |  |  |  |  |  |  |  |  |  |  |  |  |  |  |  |  |  |  |  |  |  |
|  |  |  |  |  |  |  |  |  |  |  |  |  |  |  |  |  |  |  |  |  |  |  |  |  |  |  |  |  |  |  |  |  |  |  |  |  |  |  |  |  |  |  |
|  |  |  |  |  |  |  |  |  |  |  |  |  |  |  |  |  |  |  |  |  |  |  |  |  |  |  |  |  |  |  |  |  |  |  |  |  |  |  |  |  |  |  |
|  |  |  |  |  |  |  |  |  |  |  |  |  |  |  |  |  |  |  |  |  |  |  |  |  |  |  |  |  |  |  |  |  |  |  |  |  |  |  |  |  |  |  |
|  |  |  |  |  |  |  |  |  |  |  |  |  |  |  |  |  |  |  |  |  |  |  |  |  |  |  |  |  |  |  |  |  |  |  |  |  |  |  |  |  |  |  |
|  |  |  |  |  |  |  |  |  |  |  |  |  |  |  |  |  |  |  |  |  |  |  |  |  |  |  |  |  |  |  |  |  |  |  |  |  |  |  |  |  |  |  |
|  |  |  |  |  |  |  |  |  |  |  |  |  |  |  |  |  |  |  |  |  |  |  |  |  |  |  |  |  |  |  |  |  |  |  |  |  |  |  |  |  |  |  |
|  |  |  |  |  |  |  |  |  |  |  |  |  |  |  |  |  |  |  |  |  |  |  |  |  |  |  |  |  |  |  |  |  |  |  |  |  |  |  |  |  |  |  |
|  |  |  |  |  |  |  |  |  |  |  |  |  |  |  |  |  |  |  |  |  |  |  |  |  |  |  |  |  |  |  |  |  |  |  |  |  |  |  |  |  |  |  |
|  |  |  |  |  |  |  |  |  |  |  |  |  |  |  |  |  |  |  |  |  |  |  |  |  |  |  |  |  |  |  |  |  |  |  |  |  |  |  |  |  |  |  |
|  |  |  |  |  |  |  |  |  |  |  |  |  |  |  |  |  |  |  |  |  |  |  |  |  |  |  |  |  |  |  |  |  |  |  |  |  |  |  |  |  |  |  |
|  |  |  |  |  |  |  |  |  |  |  |  |  |  |  |  |  |  |  |  |  |  |  |  |  |  |  |  |  |  |  |  |  |  |  |  |  |  |  |  |  |  |  |
|  |  |  |  |  |  |  |  |  |  |  |  |  |  |  |  |  |  |  |  |  |  |  |  |  |  |  |  |  |  |  |  |  |  |  |  |  |  |  |  |  |  |  |
|  |  |  |  |  |  |  |  |  |  |  |  |  |  |  |  |  |  |  |  |  |  |  |  |  |  |  |  |  |  |  |  |  |  |  |  |  |  |  |  |  |  |  |
|  |  |  |  |  |  |  |  |  |  |  |  |  |  |  |  |  |  |  |  |  |  |  |  |  |  |  |  |  |  |  |  |  |  |  |  |  |  |  |  |  |  |  |
|  |  |  |  |  |  |  |  |  |  |  |  |  |  |  |  |  |  |  |  |  |  |  |  |  |  |  |  |  |  |  |  |  |  |  |  |  |  |  |  |  |  |  |
|  |  |  |  |  |  |  |  |  |  |  |  |  |  |  |  |  |  |  |  |  |  |  |  |  |  |  |  |  |  |  |  |  |  |  |  |  |  |  |  |  |  |  |
|  |  |  |  |  |  |  |  |  |  |  |  |  |  |  |  |  |  |  |  |  |  |  |  |  |  |  |  |  |  |  |  |  |  |  |  |  |  |  |  |  |  |  |
|  |  |  |  |  |  |  |  |  |  |  |  |  |  |  |  |  |  |  |  |  |  |  |  |  |  |  |  |  |  |  |  |  |  |  |  |  |  |  |  |  |  |  |
|  |  |  |  |  |  |  |  |  |  |  |  |  |  |  |  |  |  |  |  |  |  |  |  |  |  |  |  |  |  |  |  |  |  |  |  |  |  |  |  |  |  |  |
|  |  |  |  |  |  |  |  |  |  |  |  |  |  |  |  |  |  |  |  |  |  |  |  |  |  |  |  |  |  |  |  |  |  |  |  |  |  |  |  |  |  |  |
|  |  |  |  |  |  |  |  |  |  |  |  |  |  |  |  |  |  |  |  |  |  |  |  |  |  |  |  |  |  |  |  |  |  |  |  |  |  |  |  |  |  |  |
|  |  |  |  |  |  |  |  |  |  |  |  |  |  |  |  |  |  |  |  |  |  |  |  |  |  |  |  |  |  |  |  |  |  |  |  |  |  |  |  |  |  |  |
|  |  |  |  |  |  |  |  |  |  |  |  |  |  |  |  |  |  |  |  |  |  |  |  |  |  |  |  |  |  |  |  |  |  |  |  |  |  |  |  |  |  |  |
|  |  |  |  |  |  |  |  |  |  |  |  |  |  |  |  |  |  |  |  |  |  |  |  |  |  |  |  |  |  |  |  |  |  |  |  |  |  |  |  |  |  |  |
|  |  |  |  |  |  |  |  |  |  |  |  |  |  |  |  |  |  |  |  |  |  |  |  |  |  |  |  |  |  |  |  |  |  |  |  |  |  |  |  |  |  |  |
|  |  |  |  |  |  |  |  |  |  |  |  |  |  |  |  |  |  |  |  |  |  |  |  |  |  |  |  |  |  |  |  |  |  |  |  |  |  |  |  |  |  |  |
|  |  |  |  |  |  |  |  |  |  |  |  |  |  |  |  |  |  |  |  |  |  |  |  |  |  |  |  |  |  |  |  |  |  |  |  |  |  |  |  |  |  |  |
|  |  |  |  |  |  |  |  |  |  |  |  |  |  |  |  |  |  |  |  |  |  |  |  |  |  |  |  |  |  |  |  |  |  |  |  |  |  |  |  |  |  |  |
|  |  |  |  |  |  |  |  |  |  |  |  |  |  |  |  |  |  |  |  |  |  |  |  |  |  |  |  |  |  |  |  |  |  |  |  |  |  |  |  |  |  |  |
|  |  |  |  |  |  |  |  |  |  |  |  |  |  |  |  |  |  |  |  |  |  |  |  |  |  |  |  |  |  |  |  |  |  |  |  |  |  |  |  |  |  |  |
|  |  |  |  |  |  |  |  |  |  |  |  |  |  |  |  |  |  |  |  |  |  |  |  |  |  |  |  |  |  |  |  |  |  |  |  |  |  |  |  |  |  |  |
|  |  |  |  |  |  |  |  |  |  |  |  |  |  |  |  |  |  |  |  |  |  |  |  |  |  |  |  |  |  |  |  |  |  |  |  |  |  |  |  |  |  |  |
|  |  |  |  |  |  |  |  |  |  |  |  |  |  |  |  |  |  |  |  |  |  |  |  |  |  |  |  |  |  |  |  |  |  |  |  |  |  |  |  |  |  |  |
|  |  |  |  |  |  |  |  |  |  |  |  |  |  |  |  |  |  |  |  |  |  |  |  |  |  |  |  |  |  |  |  |  |  |  |  |  |  |  |  |  |  |  |
|  |  |  |  |  |  |  |  |  |  |  |  |  |  |  |  |  |  |  |  |  |  |  |  |  |  |  |  |  |  |  |  |  |  |  |  |  |  |  |  |  |  |  |
|  |  |  |  |  |  |  |  |  |  |  |  |  |  |  |  |  |  |  |  |  |  |  |  |  |  |  |  |  |  |  |  |  |  |  |  |  |  |  |  |  |  |  |
|  |  |  |  |  |  |  |  |  |  |  |  |  |  |  |  |  |  |  |  |  |  |  |  |  |  |  |  |  |  |  |  |  |  |  |  |  |  |  |  |  |  |  |
|  |  |  |  |  |  |  |  |  |  |  |  |  |  |  |  |  |  |  |  |  |  |  |  |  |  |  |  |  |  |  |  |  |  |  |  |  |  |  |  |  |  |  |
|  |  |  |  |  |  |  |  |  |  |  |  |  |  |  |  |  |  |  |  |  |  |  |  |  |  |  |  |  |  |  |  |  |  |  |  |  |  |  |  |  |  |  |
|  |  |  |  |  |  |  |  |  |  |  |  |  |  |  |  |  |  |  |  |  |  |  |  |  |  |  |  |  |  |  |  |  |  |  |  |  |  |  |  |  |  |  |
|  |  |  |  |  |  |  |  |  |  |  |  |  |  |  |  |  |  |  |  |  |  |  |  |  |  |  |  |  |  |  |  |  |  |  |  |  |  |  |  |  |  |  |
|  |  |  |  |  |  |  |  |  |  |  |  |  |  |  |  |  |  |  |  |  |  |  |  |  |  |  |  |  |  |  |  |  |  |  |  |  |  |  |  |  |  |  |
|  |  |  |  |  |  |  |  |  |  |  |  |  |  |  |  |  |  |  |  |  |  |  |  |  |  |  |  |  |  |  |  |  |  |  |  |  |  |  |  |  |  |  |
|  |  |  |  |  |  |  |  |  |  |  |  |  |  |  |  |  |  |  |  |  |  |  |  |  |  |  |  |  |  |  |  |  |  |  |  |  |  |  |  |  |  |  |
|  |  |  |  |  |  |  |  |  |  |  |  |  |  |  |  |  |  |  |  |  |  |  |  |  |  |  |  |  |  |  |  |  |  |  |  |  |  |  |  |  |  |  |
|  |  |  |  |  |  |  |  |  |  |  |  |  |  |  |  |  |  |  |  |  |  |  |  |  |  |  |  |  |  |  |  |  |  |  |  |  |  |  |  |  |  |  |
|  |  |  |  |  |  |  |  |  |  |  |  |  |  |  |  |  |  |  |  |  |  |  |  |  |  |  |  |  |  |  |  |  |  |  |  |  |  |  |  |  |  |  |
|  |  |  |  |  |  |  |  |  |  |  |  |  |  |  |  |  |  |  |  |  |  |  |  |  |  |  |  |  |  |  |  |  |  |  |  |  |  |  |  |  |  |  |
|  |  |  |  |  |  |  |  |  |  |  |  |  |  |  |  |  |  |  |  |  |  |  |  |  |  |  |  |  |  |  |  |  |  |  |  |  |  |  |  |  |  |  |
|  |  |  |  |  |  |  |  |  |  |  |  |  |  |  |  |  |  |  |  |  |  |  |  |  |  |  |  |  |  |  |  |  |  |  |  |  |  |  |  |  |  |  |
|  |  |  |  |  |  |  |  |  |  |  |  |  |  |  |  |  |  |  |  |  |  |  |  |  |  |  |  |  |  |  |  |  |  |  |  |  |  |  |  |  |  |  |
|  |  |  |  |  |  |  |  |  |  |  |  |  |  |  |  |  |  |  |  |  |  |  |  |  |  |  |  |  |  |  |  |  |  |  |  |  |  |  |  |  |  |  |
|  |  |  |  |  |  |  |  |  |  |  |  |  |  |  |  |  |  |  |  |  |  |  |  |  |  |  |  |  |  |  |  |  |  |  |  |  |  |  |  |  |  |  |
|  |  |  |  |  |  |  |  |  |  |  |  |  |  |  |  |  |  |  |  |  |  |  |  |  |  |  |  |  |  |  |  |  |  |  |  |  |  |  |  |  |  |  |
|  |  |  |  |  |  |  |  |  |  |  |  |  |  |  |  |  |  |  |  |  |  |  |  |  |  |  |  |  |  |  |  |  |  |  |  |  |  |  |  |  |  |  |
|  |  |  |  |  |  |  |  |  |  |  |  |  |  |  |  |  |  |  |  |  |  |  |  |  |  |  |  |  |  |  |  |  |  |  |  |  |  |  |  |  |  |  |
|  |  |  |  |  |  |  |  |  |  |  |  |  |  |  |  |  |  |  |  |  |  |  |  |  |  |  |  |  |  |  |  |  |  |  |  |  |  |  |  |  |  |  |
|  |  |  |  |  |  |  |  |  |  |  |  |  |  |  |  |  |  |  |  |  |  |  |  |  |  |  |  |  |  |  |  |  |  |  |  |  |  |  |  |  |  |  |
|  |  |  |  |  |  |  |  |  |  |  |  |  |  |  |  |  |  |  |  |  |  |  |  |  |  |  |  |  |  |  |  |  |  |  |  |  |  |  |  |  |  |  |
|  |  |  |  |  |  |  |  |  |  |  |  |  |  |  |  |  |  |  |  |  |  |  |  |  |  |  |  |  |  |  |  |  |  |  |  |  |  |  |  |  |  |  |
|  |  |  |  |  |  |  |  |  |  |  |  |  |  |  |  |  |  |  |  |  |  |  |  |  |  |  |  |  |  |  |  |  |  |  |  |  |  |  |  |  |  |  |
|  |  |  |  |  |  |  |  |  |  |  |  |  |  |  |  |  |  |  |  |  |  |  |  |  |  |  |  |  |  |  |  |  |  |  |  |  |  |  |  |  |  |  |
|  |  |  |  |  |  |  |  |  |  |  |  |  |  |  |  |  |  |  |  |  |  |  |  |  |  |  |  |  |  |  |  |  |  |  |  |  |  |  |  |  |  |  |
|  |  |  |  |  |  |  |  |  |  |  |  |  |  |  |  |  |  |  |  |  |  |  |  |  |  |  |  |  |  |  |  |  |  |  |  |  |  |  |  |  |  |  |
|  |  |  |  |  |  |  |  |  |  |  |  |  |  |  |  |  |  |  |  |  |  |  |  |  |  |  |  |  |  |  |  |  |  |  |  |  |  |  |  |  |  |  |
|  |  |  |  |  |  |  |  |  |  |  |  |  |  |  |  |  |  |  |  |  |  |  |  |  |  |  |  |  |  |  |  |  |  |  |  |  |  |  |  |  |  |  |
|  |  |  |  |  |  |  |  |  |  |  |  |  |  |  |  |  |  |  |  |  |  |  |  |  |  |  |  |  |  |  |  |  |  |  |  |  |  |  |  |  |  |  |
|  |  |  |  |  |  |  |  |  |  |  |  |  |  |  |  |  |  |  |  |  |  |  |  |  |  |  |  |  |  |  |  |  |  |  |  |  |  |  |  |  |  |  |
|  |  |  |  |  |  |  |  |  |  |  |  |  |  |  |  |  |  |  |  |  |  |  |  |  |  |  |  |  |  |  |  |  |  |  |  |  |  |  |  |  |  |  |
|  |  |  |  |  |  |  |  |  |  |  |  |  |  |  |  |  |  |  |  |  |  |  |  |  |  |  |  |  |  |  |  |  |  |  |  |  |  |  |  |  |  |  |
|  |  |  |  |  |  |  |  |  |  |  |  |  |  |  |  |  |  |  |  |  |  |  |  |  |  |  |  |  |  |  |  |  |  |  |  |  |  |  |  |  |  |  |
|  |  |  |  |  |  |  |  |  |  |  |  |  |  |  |  |  |  |  |  |  |  |  |  |  |  |  |  |  |  |  |  |  |  |  |  |  |  |  |  |  |  |  |
|  |  |  |  |  |  |  |  |  |  |  |  |  |  |  |  |  |  |  |  |  |  |  |  |  |  |  |  |  |  |  |  |  |  |  |  |  |  |  |  |  |  |  |
|  |  |  |  |  |  |  |  |  |  |  |  |  |  |  |  |  |  |  |  |  |  |  |  |  |  |  |  |  |  |  |  |  |  |  |  |  |  |  |  |  |  |  |
|  |  |  |  |  |  |  |  |  |  |  |  |  |  |  |  |  |  |  |  |  |  |  |  |  |  |  |  |  |  |  |  |  |  |  |  |  |  |  |  |  |  |  |
|  |  |  |  |  |  |  |  |  |  |  |  |  |  |  |  |  |  |  |  |  |  |  |  |  |  |  |  |  |  |  |  |  |  |  |  |  |  |  |  |  |  |  |
|  |  |  |  |  |  |  |  |  |  |  |  |  |  |  |  |  |  |  |  |  |  |  |  |  |  |  |  |  |  |  |  |  |  |  |  |  |  |  |  |  |  |  |
|  |  |  |  |  |  |  |  |  |  |  |  |  |  |  |  |  |  |  |  |  |  |  |  |  |  |  |  |  |  |  |  |  |  |  |  |  |  |  |  |  |  |  |
|  |  |  |  |  |  |  |  |  |  |  |  |  |  |  |  |  |  |  |  |  |  |  |  |  |  |  |  |  |  |  |  |  |  |  |  |  |  |  |  |  |  |  |
|  |  |  |  |  |  |  |  |  |  |  |  |  |  |  |  |  |  |  |  |  |  |  |  |  |  |  |  |  |  |  |  |  |  |  |  |  |  |  |  |  |  |  |
|  |  |  |  |  |  |  |  |  |  |  |  |  |  |  |  |  |  |  |  |  |  |  |  |  |  |  |  |  |  |  |  |  |  |  |  |  |  |  |  |  |  |  |
|  |  |  |  |  |  |  |  |  |  |  |  |  |  |  |  |  |  |  |  |  |  |  |  |  |  |  |  |  |  |  |  |  |  |  |  |  |  |  |  |  |  |  |
|  |  |  |  |  |  |  |  |  |  |  |  |  |  |  |  |  |  |  |  |  |  |  |  |  |  |  |  |  |  |  |  |  |  |  |  |  |  |  |  |  |  |  |
