# Supplementary material for: Effects of Ecosystem Recovery Types on Soil Phosphorus Bioavailability, Roles of Plant and Microbial Diversity: A Meta‐Analysis
Source: Ecol Evol. 2025 Apr 25;15(4):e71172. doi: 10.1002/ece3.71172 (PMC12032196; doi:10.1002/ece3.71172)
Supplement: Supplementary file 2 — Appendix S2. [file ECE3-15-e71172-s004.docx]

**Supplementary information1**

**Effects of ecosystem recovery types on soil phosphorus bioavailability, roles of plant and microbial diversity: a meta-analysis**

Jinguo Hua ^1^, Wenyue Wang ^2^, Jinyu Huo ^1^, Lin Wu ^1^, Lingfeng Huang ^1*^, Hongtao Zhong ^1,3*^

^1^ Key Laboratory of the Ministry of Education for Coastal and Wetland Ecosystems, College of the Environment and Ecology, Xiamen University, Xiamen, Fujian 361102, China

^2^ College of Horticulture and Forestry, Huazhong Agricultural University, Wuhan, Hubei 430070, China

^3^ School of Biological Sciences, The University of Western Australia, Perth, WA 6009, Australia

*Authors for correspondence:

Hongtao Zhong (zhonght@xmu.edu.cn), Lingfeng Huang (huanglf@xmu.edu.cn)

**List of Contents**

**Fig. S1.** The PRISMA flow diagram.

**Fig. S2.** Distribution of experiments included in this meta-analysis in relation to effect of restoration types on soil phosphorus bioavailability, plant and microbial diversity.

**Fig. S3.** Funnel charts of data sensitivity analysis results.

**Fig. S4.** Effects of recovery type on phosphorus levels between forest and grassland ecosystems.

**Fig. S5.** Effects of recovery types on phosphorus levels between different restoration durations.

**Notes S1.** Studies included in the meta-analysis.


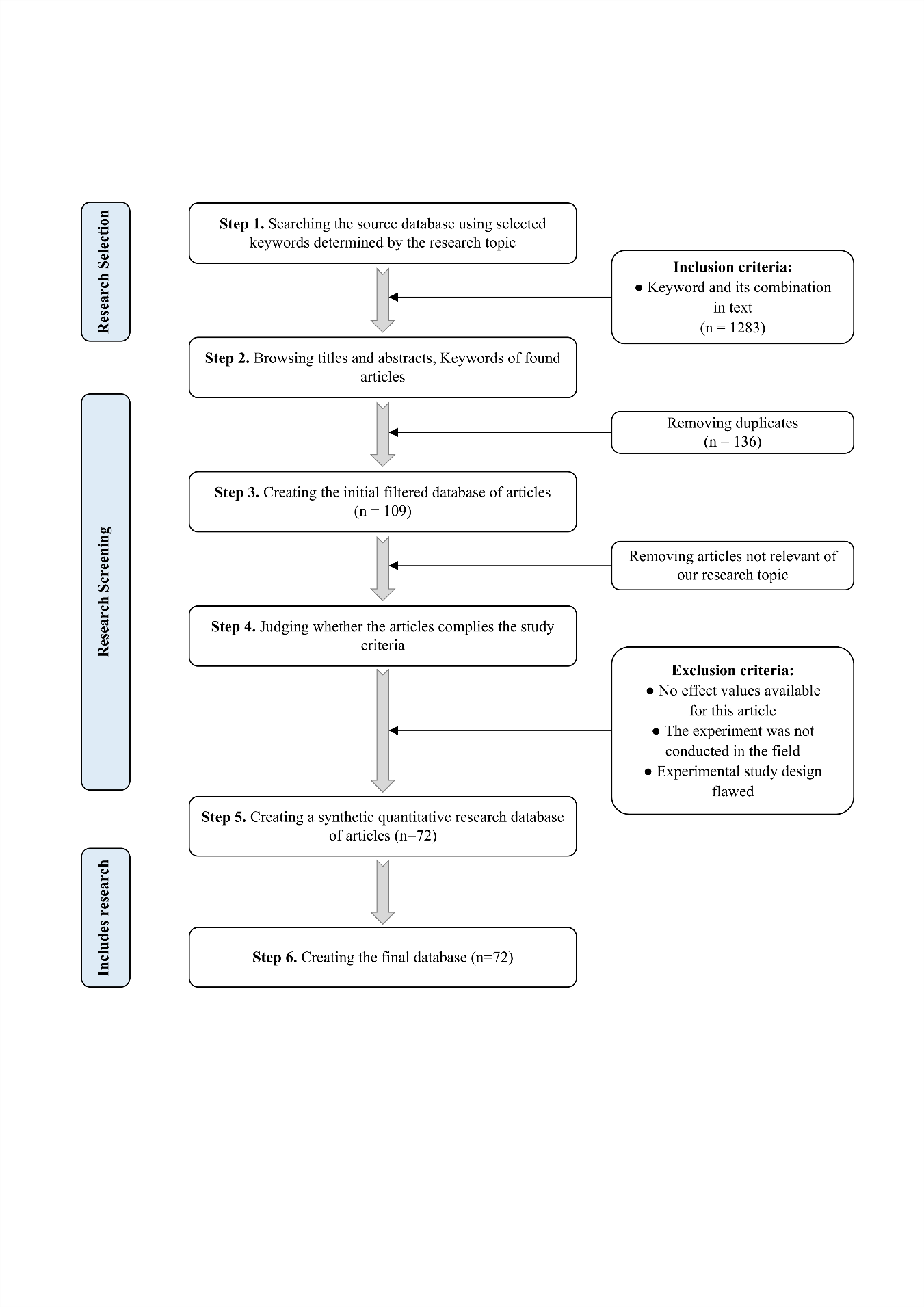


**Fig. S1.** The PRISMA flow diagram.

**
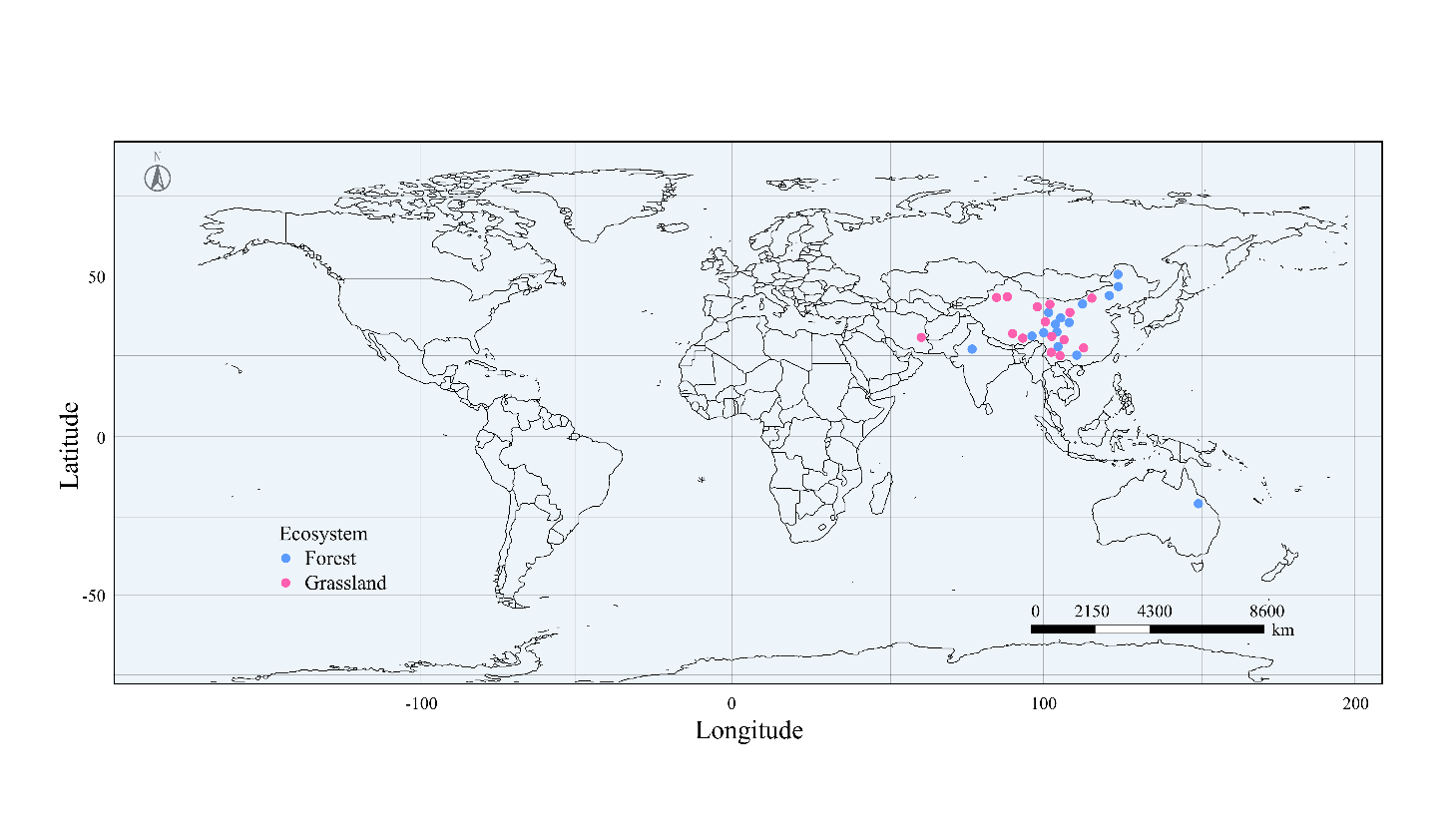
**

**Fig. S2.** Distribution of experiments included in this meta-analysis in relation to effect of recovery types on soil phosphorus bioavailability, plant and microbial diversity. Note that in some cases there are overlapped data points.


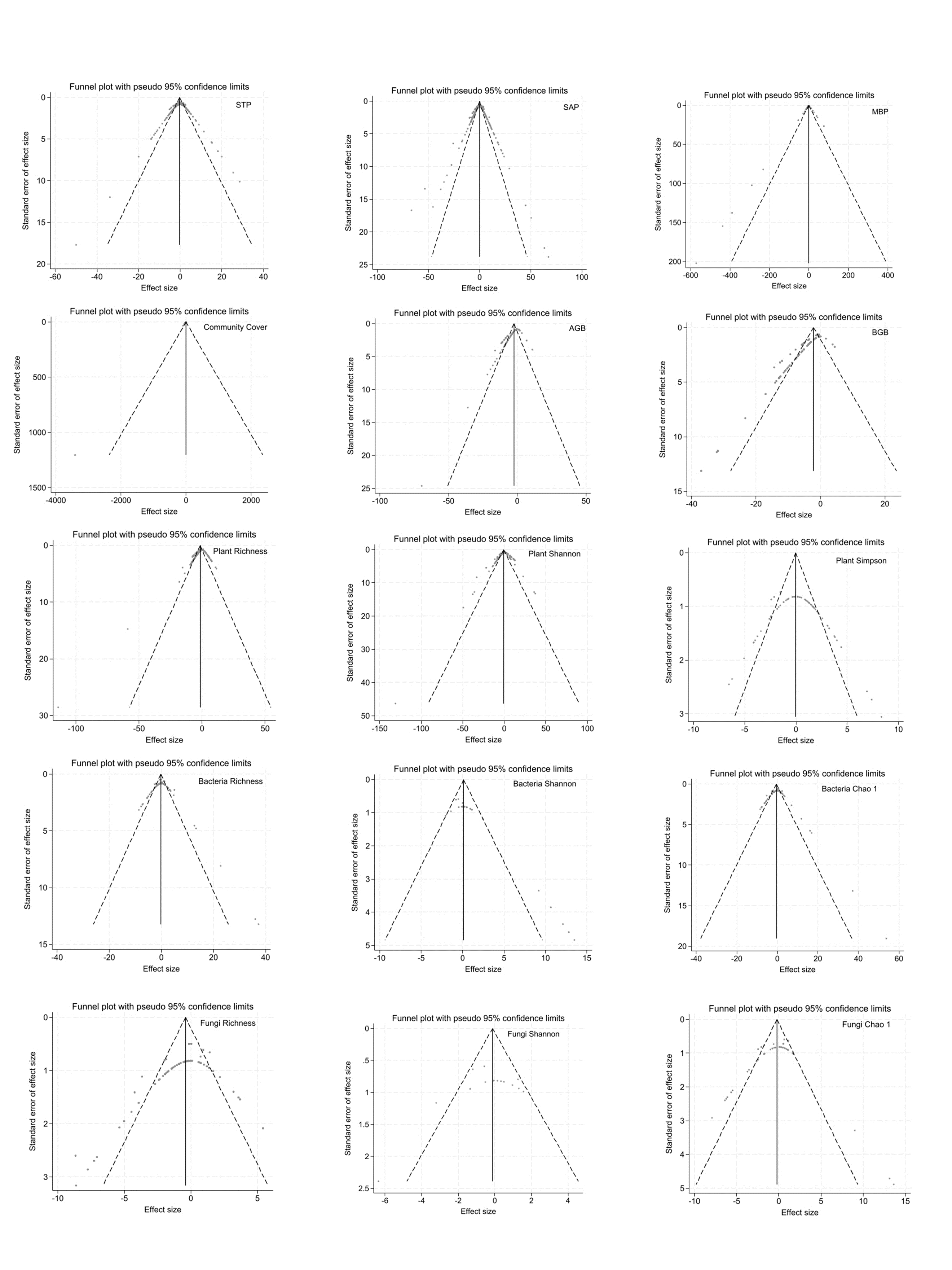


**Fig. S3.** Funnel charts of data sensitivity analysis results. STP, soil total phosphorus; SAP, soil available phosphorus; MBP, microbial biomass phosphorus; AGB, aboveground biomass; BGB, belowground biomass.


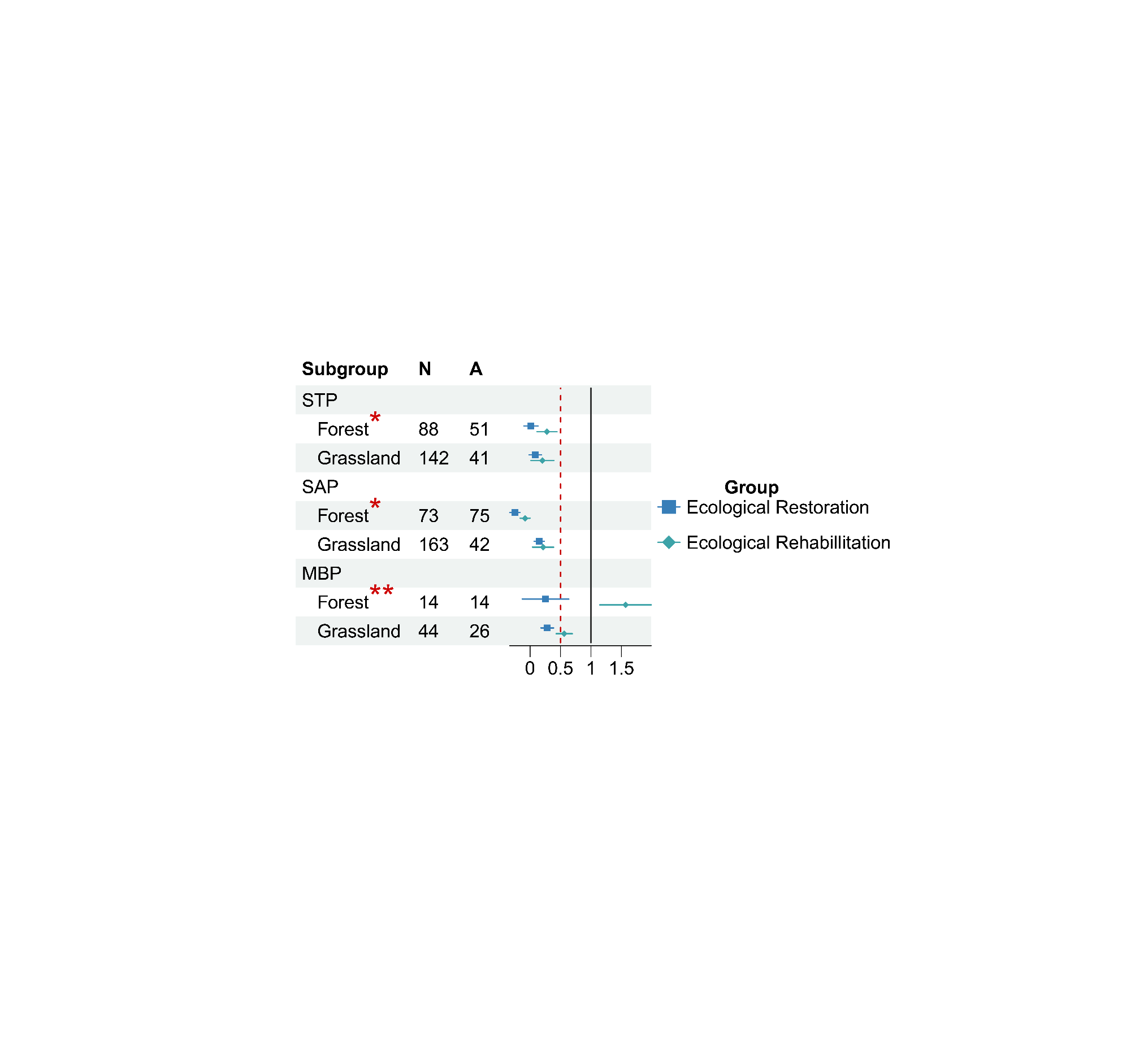


**Fig. S4.** Effects of recovery types on phosphorus levels between forest and grassland ecosystems. N, ecological restoration; A, ecological rehabilitation. Second and third columns are the sample size for the variables. Points with error bars represent weighted means and their 95% RR confidence intervals (CI). Red asterisks indicate that responses between different recovery indicators were considered significant, while * indicates *P* < 0.05 and ** indicate *P* < 0.01. STP, soil total phosphorus; SAP, soil available phosphorus; MBP, microbial biomass phosphorus.


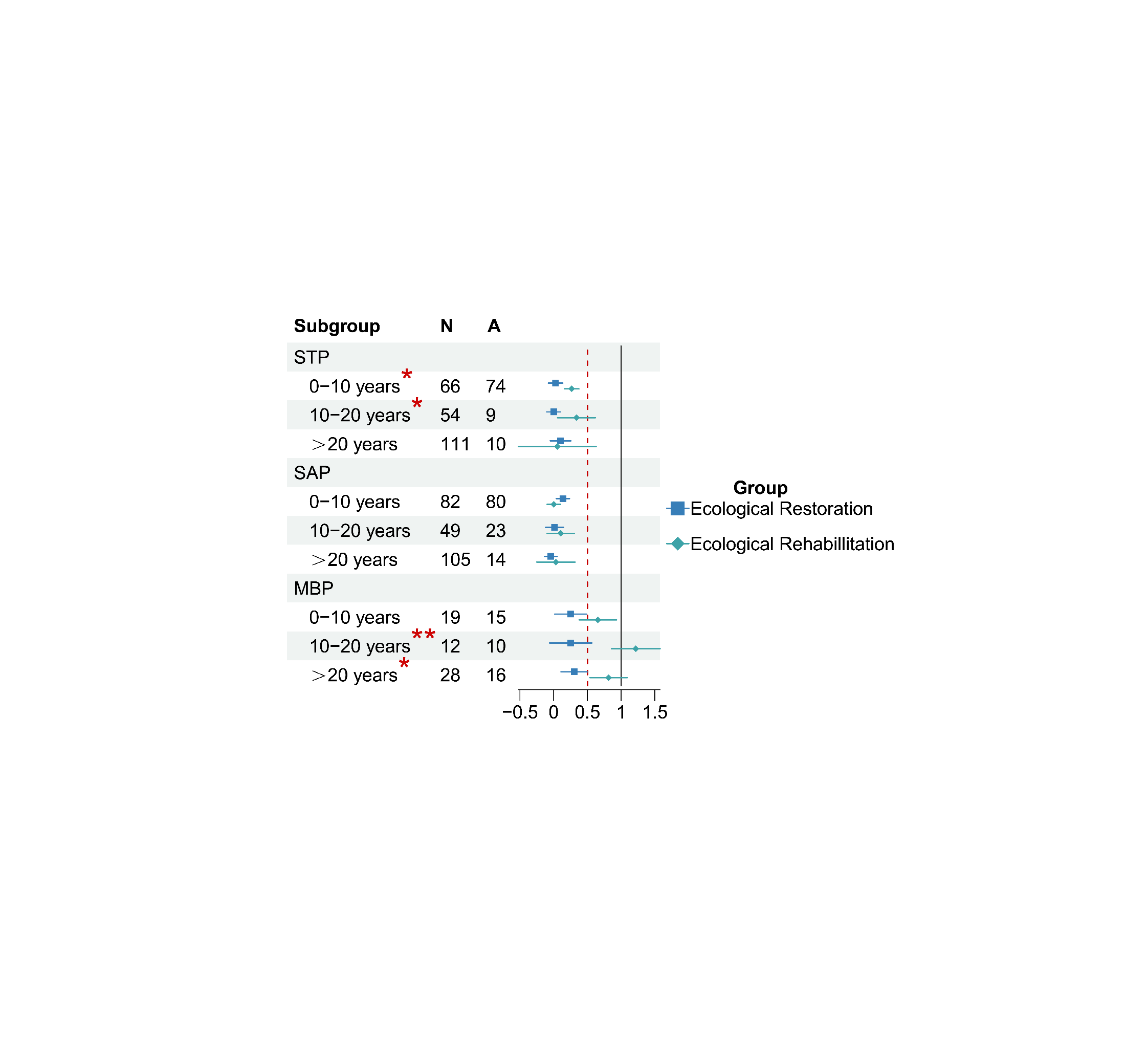


**Fig. S5.** Effects of recovery types on phosphorus levels between different restoration durations. N, ecological restoration; A, ecological rehabilitation. Second and third columns are the sample size for the variables. Points with error bars represent weighted means and their 95% RR confidence intervals (CI). Red asterisks indicate that responses between different recovery indicators were considered significant, while * indicates *P* < 0.05 and ** indicate *P* < 0.01. STP, soil total phosphorus; SAP, soil available phosphorus; MBP, microbial biomass phosphorus.

**Notes S1.** Studies included in the meta-analysis.

Amarasinghe, A., Knox, O.G.G., Fyfe, C., Lobry de Bruyn, L.A, Wilson, B.R., 2021. Response of soil microbial functionality and soil properties to environmental plantings across a chronosequence in south eastern Australia. Appl. Soil Ecol. 168, 104100. https://doi.org/10.1016/j.apsoil.2021.104100.

Chen, X., Zhang, Z., Han, X., Hao, X., Lu, X., Yan, J., Biswas, A., Dunfield, K., Zou, W., 2021. Impacts of land-use changes on the variability of microbiomes in soil profiles. J. Sci. Food Agric. 101, 5056–5066. https://doi.org/10.1002/jsfa.11150.

Chen, X.X, Li, H.L., Dong, Z., Chen, P., Qiu, S.Q., Gao, Y., 2019. Effects of soil remediation and changes of plant diversity in burned areas under different afforestation models (in China). Journal of Soil and Water Conservation 03, 332-339. https://doi.org/10.13870/j.cnki.stbcxb.2019.03.049.

Cheng, J., Jing, G., Wei, L., Jing, Z., 2016. Long-term grazing exclusion effects on vegetation characteristics, soil properties and bacterial communities in the semi-arid grasslands of China. Ecological engineering: The Journal of Ecotechnology 97, 170–178. https://doi.org/10.1016/j.ecoleng.2016.09.003.

Cheng, Y., An, S. S., Li, G.H., Li, D.H., 2010. Effects of vegetation restoration on soil nutrients and microbial biomass in loess hilly region of Ningxia (in China). Journal of China Ecological Agriculture 18(02), 261-266. <https://doi.org/10.3724/SP.J.1011.2010.00261>.

Cui, Y., Fang, L., Guo, X., Wang, X., Wang, Y., Zhang, Y., Zhang, X., 2018. Responses of soil bacterial communities, enzyme activities, and nutrients to agricultural-to-natural ecosystem conversion in the Loess Plateau, China. Journal of Soils & Sediments 19, 1–14. https://doi.org/10.1007/s11368-018-2110-4.

Deng, L., Peng, C., Huang, C., Wang, K., Shangguan, Z., 2019. Drivers of soil microbial metabolic limitation changes along a vegetation restoration gradient on the Loess Plateau, China. Geoderma 353, 188–200. https://doi.org/10.1016/j.geoderma.2019.06.037.

Deng, L., Zhang, Z.N., Shangguan, Z.P., 2014. Long-term fencing effects on plant diversity and soil properties in China. Soil Till Res 137, 7–15. https://doi.org/10.1016/j.still.2013.11.002.

Duan, C.W., Li, X.L., Chai, Y., Xu, W.Y., Su, L.L., Yang, X.G., Ma, P.P., 2022. Eco-stoichiometric characteristics of carbon, nitrogen and phosphorus in degraded alpine meadow under artificial restoration (in China). Journal of Grassland of China 07, 23-32. https://doi.org/10.16742/j.zgcdxb.20210298.

Ebrahimi, M., Khosravi, H., Rigi, M., 2016. Short-term grazing exclusion from heavy livestock rangelands affects vegetation cover and soil properties in natural ecosystems of southeastern Iran. Ecological Engineering 95, 10–18. https://doi.org/10.1016/j.ecoleng.2016.06.069.

Gao, X., Dong, S., Xu, Y., Li, Y., Li, S., Wu, S., Shen, H., Liu, S., Fry, E.L., 2021. Revegetation significantly increased the bacterial-fungal interactions in different successional stages of alpine grasslands on the Qinghai-Tibetan Plateau. Catena 205, 105385. https://doi.org/10.1016/j.catena.2021.105385.

Ge, P., Cai, T., Da. L.J., Cui, Y.Y., Wang, Y.H., Yu, F.D., 2014. Evolution of soil nutrients and microorganisms in *Pinus massoniana* secondary forest at different restoration stages (in China). Chinese agricultural science bulletin 22, 22-27. http://www.casb.org.cn.

Guo, J., Jiang, H., Bian, H., Sheng, L., He, C., Gao, Y., 2017. Natural succession is a feasible approach for cultivated peatland restoration in Northeast China. Ecological Engineering 104, 39–44. https://doi.org/10.1016/j.ecoleng.2017.04.001.

Guo, N., Degen, A.A., Deng, B., Shi, F., Shang, Z., 2019. Changes in vegetation parameters and soil nutrients along degradation and recovery successions on alpine grasslands of the Tibetan plateau. Agriculture, Ecosystems & Environment 284, 106593. https://doi.org/10.1016/j.agee.2019.106593.

Han, C., Liang, D., Zhou, W., Xu, Q., Xiang, M., Gu, Y., Siddique, K.H.M., 2024. Soil, Plant, and Microorganism Interactions Drive Secondary Succession in Alpine Grassland Restoration. Plants-Basel 13, 780. https://doi.org/10.3390/plants13060780.

Hou, X., Han, H., Tigabu, M., Cai, L., Ma, X., 2019. Changes in soil physico-chemical properties following vegetation restoration mediate bacterial community composition and diversity in Changting, China. Ecological Engineering 138, 171–179. https://doi.org/10.1016/j.ecoleng.2019.07.031.

Hu, F., Du, H., Zeng, F.P., Song, T.Q., Peng, W.X., Zhang, F., 2018. Effects of different vegetation restoration on soil nutrient content and microbial diversity in typical karst peak-cluster depressions (in China). Acta Ecologica Sinica 06, 2170-2179. https://doi.org/10.5846 /stxb201703180458.

Hu, H.Y., Zhang, Y.L., Chu, Z.P., Huang, C.F., Wang, S.Z., Zhou, C.F., 2021. Changes of soil enzyme activities and microbial diversity in different vegetation restoration stages in red soil erosion areas (in China). Journal of Applied and Environmental Biology 03, 734-741. https://doi.org/10.19675/j.cnki.1006-687x.2020.

Hu, J.J., Zhou, Q., Cao, Q., Hu, J, 2022. Effects of ecological restoration measures on vegetation and soil properties in semi-humid sandy land on the southeast Qinghai-Tibetan Plateau, China. Glob. Ecol. Conserv. 33, e02000. https://doi.org/10.1016/j.gecco.2022.e02000.

Hu, M., Sardans, J., Sun, D., Yan, R., Wu, H., Ni, R., Peuelas, J., 2024. Microbial diversity and keystone species drive soil nutrient cycling and multifunctionality following mangrove restoration. Environmental Research 251, 118715. https://doi.org/10.1016/j.envres.2024.118715.

Jing, Z., Cheng, J., Su, J., Bai, Y., Jin, J., 2014. Changes in plant community composition and soil properties under 3-decade grazing exclusion in semiarid grassland. Ecological Engineering 64, 171–178. https://doi.org/10.1016/j.ecoleng.2013.12.023.

Li, Q., Zhou, D., Jin, Y., Wang, M., Song, Y., Li, G., 2014. Effects of fencing on vegetation and soil restoration in a degraded alkaline grassland in northeast China. Journal of Arid Land 6, 478-487. https://doi.org/10.1007/s40333-013-0207-6.

Li, W., Liu, Y., Wang, J., Shi, S., Cao, W., 2018. Six years of grazing exclusion is the optimum duration in the alpine meadow-steppe of the north-eastern Qinghai-Tibetan Plateau. Sci Rep 8, 17269. https://doi.org/10.1038/s41598-018-35273-y.

Li, X.N., Wang, C., Zhang, W.W., Zhao, C.Q., Shi, R.S., Xue, R.B., Fan, R., 2019. Effects of short-term vegetation restoration on soil physical and chemical properties and microbial community structure in wasteland of Beijing suburbs (in China). Journal of Soil and Water Conservation 05, 343-348+357. https://doi.org/10.13870/j.cnki.stbcxb.2019.05.050.

Li, Y., Shi, C., Wei, D., Ding, J., Xu, N., Jin, L., Wang, L., 2023. Associations of soil bacterial diversity and function with plant diversity in *Carex* tussock wetland. Front. Microbiol. 14, 1142052. https://doi.org/10.3389/fmicb.2023.1142052.

Li, Y.R., Hu, S., Lang, S., Pu, Y., Zhang, S., Li, T., Xu, X., Jia, Y., Wang, G., Yuan, D., Li, Yun, 2023. Soil quality and ecological benefits assessment of alpine desertified grassland following different ecological restoration measures. Front. Plant Sci. 14, 1283457. https://doi.org/10.3389/fpls.2023.1283457.

Liao, H., Zheng, C., Li, J., Long, J., 2018. Dynamics of soil microbial recovery from cropland to orchard along a 20-year chronosequence in a degraded karst ecosystem. Sci. Total Environ. 639, 1051–1059. https://doi.org/10.1016/j.scitotenv.2018.05.246.

Liu, D., Huang, Y.M., An, S.S., 2012. Changes of soil nitrogen and microbial activity during the restoration of artificial *Robinia pseudoacacia* forest in loess hilly region (in China). Journal of China Ecological Agriculture 20 (03), 322-329. https://doi.org/10.3724/SP.J.1011.2012.00322.

Liu, G., Bai, Z., Shah, F., Cui, G., Xiao, Z., Gong, H., Li, D., Lin, Y., Li, B., Ji, G., Shah, S., 2021. Compositional and structural changes in soil microbial communities in response to straw mulching and plant revegetation in an abandoned artificial pasture in Northeast China. Glob. Ecol. Conserv. 31, 01871. https://doi.org/10.1016/j.gecco.2021.e01871.

Liu, H.F., Liang C.T., Ai, Z., Zhang, J.Y., Wu, Y., Xue, S., Liu, G.B., 2019. Plant‐mycorrhizae association affects plant diversity, biomass, and soil nutrients along temporal gradients of natural restoration after farmland abandonment in the Loess Plateau, China. Land Degradation and Development 30, 1677-1690. https://doi.org/10.1002/ldr.3372.

Liu, J., Bian, Z., Zhang, K., Ahmad, B., Khan, A., 2019. Effects of different fencing regimes on community structure of degraded desert grasslands on Mu Us desert, China. Ecol. Evol. 9, 3367–3377. https://doi.org/10.1002/ece3.4958.

Liu, W., Fu, S., Yan, S., Ren, C., Wu, S., Deng, J., Li, B., Han, X., Yang, G., 2020. Responses of plant community to the linkages in plant-soil C: N: P stoichiometry during secondary succession of abandoned farmlands, China. J. Arid Land 12, 215–226. https://doi.org/10.1007/s40333-020-0009-6.

Liu, Y., Hou, Z.S., Zhao, S., Qi, G.H., Zhang, X.M., Guo, S.P., 2018. Evolution of plant diversity and soil characteristics in the process of vegetation restoration in gneiss mountain area of Taihang Mountain (in China). Journal of Ecology 38 (15), 5331-5339. https://doi.org/10.5846 /stxb201709271749.

Miao, R., Jiang, D., Musa, A., Zhou, Q., Guo, M., Wang, Y., 2015. Effectiveness of shrub planting and grazing exclusion on degraded sandy grassland restoration in Horqin sandy land in Inner Mongolia. Ecol. Eng. 74, 164–173. https://doi.org/10.1016/j.ecoleng.2014.10.004.

Mir, A.H., Dad, J.M., Singh, B., Kamili, A.N., 2022. Passive restoration considerably improved the community structure, soil health and carbon stock in the Pine forests of Kashmir Himalaya, India. Ecol. Eng. 176, 106535. https://doi.org/10.1016/j.ecoleng.2021.106535.

Pan, Y., Kang, P., Qu, X., Ran, Y., Li, X., 2024. Effects of long-term fencing on soil microbial community structure and function in the desert steppe, China. Journal of Arid LandIssue 16 (3), 431-446. https://doi.org/10.1007/s40333-024-0009-z.

Rong, Y., Yuan, F., Ma, L., 2014. Effectiveness of exclosures for restoring soils and vegetation degraded by overgrazing in the Junggar Basin, China. Grassland Science 60, 118–124. https://doi.org/10.1111/grs.12048.

Song, D.C., Wu, H., Wang, L.D., He, F.L., Zhao, X.R., Han, S.H., Xu, B.Y., 2021. Variation characteristics of soil microorganisms and soil enzyme activities in secondary grassland in Minqin area (in China). Journal of Grassland of China 43 (06), 85-93. https://doi.org/06,85-93.10.16742/j.zgcdxb.20200224.

Song, X.C., Wang, H.L., Qin, W.D., Deng, X.J., Tian, H.D., Tan, Y.B., Cao, J.Z., 2019. Effects of different restoration types of degraded plantations on functional diversity of soil microbial communities (in China). Acta Applied Ecology 03, 841-848. <https://doi.org/10.13287/j.1001-9332.201903.036>.

Su, J., Jing, G., Jin, J., Wei, L., Liu, J., Cheng, J., 2017. Identifying drivers of root community compositional changes in semiarid grassland on the Loess plateau after long-term grazing exclusion. Ecological engineering: The Journal of Ecotechnology 99, 13–21. https://doi.org/10.1016/j.ecoleng.2016.11.050.

Sun, D., Huang, Y., Wang, Z., Tang, X., Ye, W., Cao, H., Shen, H., 2024. Soil microbial community structure, function and network along a mangrove forest restoration chronosequence. Sci. Total Environ. 913, 169704. https://doi.org/10.1016/j.scitotenv.2023.169704.

Tao, J.Y., Tan, J.L., Zheng, F.L., Wang, X.N., Zhang, W.J., 2022. Effects of vegetation restoration model on soil main enzyme activities, microbial diversity and soil nutrients in southern Ningxia (in China). Agricultural Research in Arid Areas 03, 207-217. https://doi.org/10.7606/j.issn.1000-7601.2022.03.25.

Wang, A.N., Huang, Q.X., Li, X.G., Xu, X.H., Li, Y.L., 2019. Bacterial community structure and diversity in rhizosphere soil of different vegetation restoration types in northern Hebei Province (in China). Forestry Science 09, 130-141. https://doi.org/10.11707/j.1001-7488.20190914.

Wang, J., Wang, X., Liu, G., Wang, G., Zhang, C., 2020. Fencing as an effective approach for restoration of alpine meadows: Evidence from nutrient limitation of soil microbes. Geoderma 363, 114148. https://doi.org/10.1016/j.geoderma.2019.114148.

Wang, J., Wang, X., Liu, G., Wang, G., Zhang, C., 2021. Grazing-to-fencing conversion affects soil microbial composition, functional profiles by altering plant functional groups in a Tibetan alpine meadow. Applied Soil Ecology 166, 104008. https://doi.org/10.1016/j.apsoil.2021.104008.

Wang, X., Song, N.P., Yang, X.G., Yang, M.X., Xiao, X.P., 2013. Response of grassland plant diversity to soil factors under grazing disturbance (in China). Acta prataculture 05, 27-36. https://doi.org/10. 11686/ cyxb20130504.

Wang, Z., Li, X., Ji, B., Struik, P.C., Tang, S., 2021. Coupling Between the Responses of Plants, Soil, and Microorganisms Following Grazing Exclusion in an Overgrazed Grassland. Frontiers in Plant Science 12, 640789. https://doi.org/10.3389/fpls.2021.640789.

Wang, Z., Zhang, Q., Staley, C., Gao, H., Ishii, S., Wei, X., Liu, J., Cheng, J., Hao, M., Sadowsky, M.J., 2019. Impact of long-term grazing exclusion on soil microbial community composition and nutrient availability. Biology and Fertility of Soils 55, 121–134. https://doi.org/10.1007/s00374-018-01336-5.

Wu, C.D., Meng, Z.J., Liu, M.H., Meng, X.Z., Zhou, C.L., 2024. Plant diversity and its relationship with soil chemical factors in the wetland of Naoli River Nature Reserve (in China). Journal of Jiangxi Agricultural University 04, 969-979. https://doi.org/10.3724/aauj.2024086.

Wu, X.Y., Lu, H., Wang, X.J., 2007. Changes of plant diversity, biomass and soil nutrient content after natural restoration of cut-off land of sand-fixing forest (in China). Journal of Ecology 07, 978-982. https://doi.org/10.13292/j.1000-4890.2007.0176.

Xiang, K.X., Zhang, X., Liu, J.M., Xue, J.H., Cui, Y.C., Wu, Y.B., 2019. Correlation between plant diversity index of cypress forest and soil physical and chemical indexes in Shimo District (in China). Journal of south China agriculture 08,1771-1778. https://doi.org/10.3969/j.issn.2095-1191.2019.08.17.

Xu, X., Cui, F., Fu, L., Fan, P., Wang, M., Cai, Z., 2024. A pioneer tree species rapidly facilitating ecosystem restoration in coastal regions depends on soil traits. Catena 238, 107825. https://doi.org/10.1016/j.catena.2024.107825.

Yan, B., Sun, L., Li, J., Liang, C., Wei, F., Xue, S., Wang, G., 2020. Change in composition and potential functional genes of soil bacterial and fungal communities with secondary succession in Quercus liaotungensis forests of the Loess Plateau, western China. Geoderma 364, 114199. https://doi.org/10.1016/j.geoderma.2020.114199.

Yan, C.L., Xue, Y., Wang, Y.F., Kang, H.B., Wang, D.X., 2024. Changes of plant diversity and its driving factors in abandoned farmland at different restoration stages in the middle Qinling Mountains (in China). Environmental Science 02, 992-1003. <https://doi.org/10.13227/j.hjkx.202303002>.

Yang, F., Huang, M., Li, C., Wu, X., Fang, L., 2022. Vegetation restoration increases the diversity of bacterial communities in deep soils. Appl. Soil Ecol. 180, 104631. https://doi.org/10.1016/j.apsoil.2022.104631.

Yang, S., Feng, C., Ma, Y., Wang, W., Huang, C., Qi, C., Fu, S., Chen, H.Y.H., 2021. Transition from N to P limited soil nutrients over time since restoration in degraded subtropical broadleaved mixed forests. For. Ecol. Manage. 494, 119298. https://doi.org/10.1016/j.foreco.2021.119298.

Yang, Y., Liu, H., Yang, X., Yao, H., Deng, X., Wang, Y., An, S., Kuzyakov, Y., Chang, S.X., 2022. Plant and soil elemental C: N: P ratios are linked to soil microbial diversity during grassland restoration on the Loess Plateau, China. Sci. Total Environ. 806, 150557. https://doi.org/10.1016/j.scitotenv.2021.150557.

Yang, Z., Xiong, W., Xu, Y., Jiang, L., Chen, H., 2016. Soil properties and species composition under different grazing intensity in an alpine meadow on the eastern Tibetan Plateau, China. Environmental Monitoring and Assessment 188, 678. https://doi.org/10.1007/s10661-016-5663-y.

Yin, Y.L., Wang, Y.Q., Li, S.X., Liu, Y., Zhao, W., Ma, Y.S., Bao, G.S., 2019. Effects of enclosure on soil microbial community diversity and soil stoichiometry characteristics in degraded alpine meadow (in China). Journal of Applied Ecology 01, 127-136. https://doi.org/10.13287/j.1001-9332.201901.009.

Yuan, J., Ouyang, Z., Zheng, H., Xu, W., 2012. Effects of different grassland restoration approaches on soil properties in the southeastern Horqin sandy land, northern China. Applied Soil Ecology 61, 34–39. https://doi.org/10.1016/j.apsoil.2012.04.003.

Yuan, Z., Epstein, H., Li, G., 2020. Grazing exclusion did not affect soil properties in alpine meadows in the Tibetan permafrost region. Ecological Engineering 147, 105657. https://doi.org/10.1016/j.ecoleng.2019.105657.

Zhang, C., Li, J., Wang, J., Liu, G., Wang, G., Guo, L., Peng, S., 2019. Decreased temporary turnover of bacterial communities along soil depth gradient during a 35-year grazing exclusion period in a semiarid grassland. Geoderma 351, 49–58. https://doi.org/10.1016/j.geoderma.2019.05.010.

Zhang, C., Liu, G., Xue, S., Wang, G., 2016. Soil bacterial community dynamics reflect changes in plant community and soil properties during the secondary succession of abandoned farmland in the Loess Plateau. Soil Biol. Biochem. 97, 40–49. https://doi.org/10.1016/j.soilbio.2016.02.013.

Zhang, P.J., Xu, J.M., Lu, W.H., Pan, S.H., Chen, M.X., Li, K.S., Shang, X.H., 2021. Analysis of Plant Diversity and Soil Physical and Chemical Properties of *Eucalyptus urophylla* Plantation in Leizhou Peninsula (in China). Journal of Central South University of Forestry and Technology 09, 96-105. https://doi.org/10.14067/j.cnki.1673-923x.2021.09.011.

Zhang, W., Xu, Y., Gao, D., Wang, X., Liu, W., Deng, J., Han, X., Yang, G., Feng, Y., Ren, G., 2019. Ecoenzymatic stoichiometry and nutrient dynamics along a revegetation chronosequence in the soils of abandoned land and Robinia pseudoacacia plantation on the Loess Plateau, China. Soil Biology and Biochemistry 134, 1–14. https://doi.org/10.1016/j.soilbio.2019.03.017.

Zhang, X.Y., Du, W.B., Zhang, X.P., Wang, J.X., Li, D.Y., Cai, X.H., 2007. Evaluation of soil microorganisms in the process of vegetation restoration in western Sichuan and its relationship with soil factors (in China). Ecological environment 05, 1470-1474. https://doi.org/10.16258/j.cnki.1674-5906.2007.05.

Zhang, Y., Wang, G., Gou, Q., Zhang, Y., Liu, J., Gao, M., 2023. Succession of a natural desert vegetation community after long-term fencing at the edge of a desert oasis in northwest China. Front. Plant Sci. 14, 1091446. https://doi.org/10.3389/fpls.2023.1091446.

Zhang, Y., Zhao, W., 2015. Vegetation and soil property response of short-time fencing in temperate desert of the Hexi Corridor, northwestern China. Catena 133, 43–51. https://doi.org/10.1016/j.catena.2015.04.019.

Zhang, Z., Han, X., Yan, J., Zou, W., Wang, E., Lu, X., Chen, X., 2020. Keystone Microbiomes Revealed by 14 Years of Field Restoration of the Degraded Agricultural Soil Under Distinct Vegetation Scenarios. Front. Microbiol. 11, 1915. https://doi.org/10.3389/fmicb.2020.01915.

Zheng, X., Yan, M., Lin, C., Guo, B., Ding, H., Yu, J., Peng, S., Sveen, T.R., Zhang, Y., 2022. Vegetation restoration types affect soil bacterial community composition and diversity in degraded lands in subtropical of China. Restor. Ecol. 30, e13494. https://doi.org/10.1111/rec.13494.

Zhong, Z., Zhang, X., Wang, X., Fu, S., Wu, S., Lu, X., Ren, C., Han, X., Yang, G., 2020. Soil bacteria and fungi respond differently to plant diversity and plant family composition during the secondary succession of abandoned farmland on the Loess Plateau, China. Plant and Soil 448, 183–200. https://doi.org/10.1007/s11104-019-04415-0.

Zhu, G.Y., Deng, L., Zhang, X.B., Shangguan, Z.P., 2016. Effects of grazing exclusion on plant community and soil physicochemical properties in a desert steppe on the Loess Plateau, China. Ecological engineering: The Journal of Ecotechnology 90, 372–381. https://doi.org/10.1016/j.ecoleng.2016.02.001.
